# Supplementary material for: Human granulocytotropic anaplasmosis—A systematic review and analysis of the literature
Source: PLoS Negl Trop Dis. 2024 Aug 5;18(8):e0012313. doi: 10.1371/journal.pntd.0012313 (PMC11326711; doi:10.1371/journal.pntd.0012313)
Supplement: S1 Text — (DOCX) [file pntd.0012313.s001.docx]

Systematic Review Protocol

Inhaltsverzeichnis

[Review Title 2](#_Toc170647970)

[Timescale 2](#_Toc170647971)

[Review team details 2](#_Toc170647972)

[Review methods 4](#_Toc170647973)

[Review questions: 4](#_Toc170647974)

[Searches 4](#_Toc170647975)

[Condition/Domain being studies 4](#_Toc170647976)

[Participants/Population 4](#_Toc170647977)

[Intervention(s), Exposure(s) 5](#_Toc170647978)

[Comparators/Control 5](#_Toc170647979)

[Types of study to be included initially 5](#_Toc170647980)

[Primary outcome 5](#_Toc170647981)

[Measures of effect 5](#_Toc170647982)

[Secondary outcomes 5](#_Toc170647983)

[Selection strategy 5](#_Toc170647984)

[Data extraction 5](#_Toc170647985)

[Risk of bias (quality) assessment 8](#_Toc170647986)

[Strategy for data synthesis 8](#_Toc170647987)

[Analysis of subgroups or subsets 9](#_Toc170647988)

[Additional Information 9](#_Toc170647989)

[References 9](#_Toc170647990)

# Review Title

Human Anaplasmosis: A systematic review of the literature

# Timescale

Anticipated Start date: 14 November 2022

Anticipated Completion date: 27 October 2023

Stage of review at time of submission to PROSPERO:

- Preliminary searches: completed
- Piloting of study selection process: completed

# Review team details

Named Contact: Sophie Schudel

Named contact email: [Sophie.schudel@swisstph.ch](mailto:Sophie.schudel@swisstph.ch)

Organisational affiliation of the review:

- Swiss Tropical and Public Health Institute, Basel, Switzerland.
- University of Basel, Basel, Switzerland.

Team Members and their Organisational affiliation

- Sophie Schudel:
- Swiss Tropical and Public Health Institute, Basel, Switzerland.
- University of Basel, Basel, Switzerland.
- Larissa Gygax:
- Swiss Tropical and Public Health Institute, Basel, Switzerland.
- University of Basel, Basel, Switzerland.
- Esther Künzli:
- Swiss Tropical and Public Health Institute, Basel, Switzerland.
- University of Basel, Basel, Switzerland.
- Christian Kositz
- Swiss Tropical and Public Health Institute, Basel, Switzerland.
- Clinical Research Department, Faculty of Infectious and Tropical Diseases, London School of Hygiene & Tropical Medicine, London, United Kingdom.
- Andreas Neumayr:
- Swiss Tropical and Public Health Institute, Basel, Switzerland.
- University of Basel, Basel, Switzerland.
- Department of Public Health and Tropical Medicine, College of Public Health, Medical and Veterinary Sciences, James Cook University, Queensland, Australia.

Reviewer roles

- Primary reviewers: Sophie Schudel
  Secondary reviewers: Andreas Neumayr, Larissa Gygax, Christian Kositz, Esther Künzli
  Quality assessors: Esther Künzli, Christian Kositz, Andreas Neumayr

Funding: Self financed, no external funding

Conflicts of interest/ Competing interest: The authors declare that they have no known conflicts of interest.

Type and method of review: Systematic Review, Health area: Infections and infestations

Language: English

Country: Switzerland

Keywords: systematic review, anaplasma, human anaplasmosis, anaplasma phagocytophilum, human granulocytotropic ehrlichiosis

Registration information: PROSPERO 2022 CRD42022384354

Current review status: completed

# Review methods

## Review questions:

- What is the epidemiology and geographical range of human anaplasmosis?
- What are risk factors associated with acquiring anaplasmosis and for sequelae and mortality?
- What is the clinical spectrum (signs and symptoms) and the laboratory findings of human anaplasmosis?
- Are there regional differences in clinical spectrum and laboratory findings of human anaplasmosis?
- What antibiotic treatment regimens are used for human anaplasmosis?
- What is the outcome of treated and untreated human anaplasmosis and does outcome differ according to the antibiotic regimen used?

## Searches

We will search the following bibliographic databases: PubMed, Embase Elsevier, CINAHL, Scopus, Web of Science Core Collection, BIOSIS Citation Index, BIOSIS Previews, Current Contents Connect, Data Citation Index, Derwent Innovations Index, KCI-Korean Journal Database, SciELO Citation Index.

The search strategy will include terms relating to Human Anaplasmosis. The search strategy for PubMed will be the following:

(Anaplasma[Mesh:NoExp] OR "Anaplasma phagocytophilum"[Mesh] OR "Anaplasma ovis"[Mesh] OR Anaplasmosis[Mesh] OR (Ehrlichiosis[Mesh] AND Granulocytes[Mesh])) OR (Anaplasma[tiab] OR Anaplasmas[tiab] OR "HGE Agent"[tiab] OR phagocytophil*[tiab] OR "E equi"[tiab] OR "Ehrlichia equi"[tiab] OR (Ehrlichia[tiab] AND granulocytotropic[tiab]) OR Anaplasmosis[tiab] OR Anaplasmoses[tiab] OR ((Ehrlichiosis[tiab] OR Ehrlichioses[tiab]) AND granulocyt*[tiab])) NOT (Animals[Mesh] NOT Humans[Mesh])

The search term will be adapted for use with other bibliographic databases.

The search will be restricted to papers in English, German, French, Spanish or Italian. There will be no geographical restriction. There will be no date limitation set but as the first case of veterinary Anaplasmosis has been described in 1932 and the first case of Human Anaplasmosis has been described in 1994, we don’t expect to find relevant studies earlier.

The searches will be re-run just before the final analyses and further studies retrieved for inclusion.

## Condition/Domain being studies

Anaplasmosis is a zoonotic bacterial infection transmitted to humans by hard ticks (Ixo*des spp*) causing primarily an undifferentiated febrile illness.

## Participants/Population

Human anaplasmosis cases

## Intervention(s), Exposure(s)

Not applicable

## Comparators/Control

Not applicable

## Types of study to be included initially

There are no restrictions on the types of study to be included. Since the search strategy is rather broad and the estimated amount of high quality studies rather low, this review will consider all types of published study designs, not only limited to RCTs. Quality assessment will be crucial before definite inclusion. In doubt, two independent reviewers will be consulted for further assessment. What types of studies eventually will be included, will become apparent after completing the search and quality assessment

## Primary outcome

Of interest to the review are epidemiological, clinical and diagnostic findings as well as data on therapy regimens and clinical outcome of human anaplasmosis, as specified in the data extraction list.

## Measures of effect

The frequency of the signs/symptoms, laboratory abnormalities, complications/sequelae and the outcome of human anaplasmosis cases will be descriptively summarized (using percentages, medians, ranges). The obtained geographic data will be reported as maps.

## Secondary outcomes

Not applicable

## Selection strategy

Identified material will be de-duplicated both by automatic search for duplicates by Endnote software and manual search for duplicates, following this review. Titles and abstracts of material identified via searches will be screened and reviewed manually. Screening will be conducted by two reviewers. Secondly, the reference lists of identified relevant articles will be manually searched for additional studies or articles. Further identified material will be again screened and reference lists will be searched. Full text papers of potentially eligible articles will be obtained.
Inclusion criteria will be applied and full text papers selected for the review. Studies that didn’t fulfill the criteria for inclusion will be excluded and their bibliographic details will be listed in an Appendix.
Results will be reported using a PRIMSA diagram. The bibliographic software ENDNOTE will be used for storage and processing.

## Data extraction

A standardized, pre-piloted form will be used to extract data from included studies for assessment of study quality and evidence synthesis. Extracted information will include: study characteristics, patient characteristics, epidemiological, clinical and laboratory findings, diagnostic measures, therapy regimen and outcome data. A detailed list of extracted parameters is available below:

Study characteristics:

- Reference No. according to our search list
- Year
- First author
- Title
- Journal
- Country of study
- Type of study
- Study Period
- Inclusion / exclusion of article; If exclusion, reason for exclusion
- Number of HA cases reported in the reference
  - If cohort study: population characteristics
  - If cohort study: patient specificity of data

Epidemiology

- Patient's age (years)
- Patient's sex
- Most likely country of acquisition
- Most likely province of acquisition
- If imported: time between end of trip and symptoms
- Country of diagnosis
- Autochthonous or imported case
- Occupational/ recreational risk factor for tick bite
- Year of Acquisition
- Pre-existing conditions
- Immunocompromised yes/ no
- Pregnancy or breast feeding

Clinical Presentation

- Symptomatic / asymptomatic
- Hospital admission
- Duration of symptoms (fever)
- Fever
  - Highest temperature measured
- Chills
- Malaise/ fatigue
- Rash
- Presence of eschar, erythema migrans
- Headache
- Nausea
- Vomiting
- Myalgia
- Arthralgia
- Lymphadenopathy
- Other gastrointestinal symptoms
- Hepatosplenomegaly
- Pulmonary symptoms
- Other neurological symptoms
- Hypotension
- Cardiovascular symptoms
- Other symptoms
- Duration between begin of symptoms and presentation to Doctor
- Specify vector of disease
  - Tick bite remembered
    - Duration between bite and symptoms

Diagnostics

- Serology
- PCR
- Blood smear or buffy coat microscopy
- Culture
- Biopsy
- Level of diagnostic certainty
- Time of first specific diagnostic test
- Additional diagnostics
- Anaplasma species
- Coinfections
- Diagnosis given
- Leukopenia acc. Author
  - Leucopenia: exact value
  - Specify leucopenia
- Thrombocytopenia acc. Author
  - Thrombocytopenia: exact value
- Anemia acc. Author
  - Red blood cell count
- Elevated liver enzymes (at least one)
  - (AST) aspartate aminotransferase elevated
    - AST: exact value
  - (ALT) alanine aminotransferase elevated
    - ALT: exact value
  - (AP) alkaline ahosphatase elevated
    - AP: Exact Value
  - Bilirubin elevated
    - Bilirubin: exact value
- CRP elevated
  - CRP highest
- D-Dimer elevated
  - D-Dimer: exact value
- BSR elevated
  - BSR: exact value
- Procalcitonin
- Creatinine elevated
  - Creatinine: exact value
- Other laboratory findings

Treatment

- Any antibiotic treatment
  - If no antibiotics specify why
- Antibiotic used for HA
- Empirical antibiotic therapy
- Duration of antibiotic treatment
- Dosage of antibiotic treatment
- Second-line therapy
- Side effects of treatment
- Symptomatic treatment

Outcome

- Complications
- Outcome
  - Death: cause
  - Sequelae

## Risk of bias (quality) assessment

The selected full text papers will be assessed by two reviewers for methodological validity prior to inclusion into the review. Any disagreements will be resolved through discussion between the reviewers, further disagreement will be intended to be resolved by a third independent reviewer. Due to the low expected number of high-quality study designs especially questions about completeness of data and selective reporting are thought to be relevant as the great majority of studies will not be randomised or blinded.

It will be considered and discussed how the quality assessment results might have an impact on the conclusions and recommendations of the review. Discussion will be presented in the “Discussion” section of the review.

The review itself will be checked using a systematic review quality assessment tool, the PRISMA checklist.

## Strategy for data synthesis

The data will be visually presented in summary tables and synthesized narratively in a sense of an observational analysis. As far as the available data allows, associations and conclusions will be drawn. Where we can, we will attempt to group similar data.

At this stage, the decision will be made whether the data will only be synthesized narratively or if the data is sufficient for a meta-analysis. Four aspects will be assessed whether it is appropriate to combine the results in a meta-analysis.
1: Studies should be similar in terms of the patients (inclusion criteria, patient characteristics)
2: Interventions/Exposures and Comparators should be the same
3: The same outcomes should be reported (primary or secondary, as well as time frames)
4: The results should show that the effects/impacts are generally going into the same direction (visualized by forest plot using a statistical software)

If all four criteria are sufficiently fulfilled by the data from reviewed studies, a meta-analysis will be performed – due to rather expectable lack of sufficient homogeneous data, further planning in this direction is not appropriate now. In case of only some studies meeting all the criteria, it may be considered to perform a meta-analysis only using those studies. In this case, a sensitivity analysis will be carried out, using the remaining studies to test the robustness of the results. Any decisions will be justified in the text of the review, clearly setting out the reasons of why a meta-analysis was performed or not.
As the scoping search showed a relatively limited number of published studies, we may expect limitations due to a lack of data. It is likely in this review, that we will have to deal with a variety of different study designs with different study aims, resulting in a rather large heterogeneity. Furthermore, the review question isn’t just comparing two different interventions on the outcome, we want to get a further picture of human anaplasmosis. Examining the epidemiology, the clinical impact and laboratory findings, evaluating different therapy plans and outcomes, we will most likely have to deal with a large variety of data or possibly a lack of data. Since one of the aims of our review is to show the current state of knowledge, discovering possible knowledge gaps, this will be considered and discussed in the “discussion” and “conclusions” section.

## Analysis of subgroups or subsets

If the necessary data are available, subgroup analyses will be done for cases from different world regions, age groups, *Anaplasma* species as well as possible risk factors for poor outcome. As the scope and quality of data is still unclear at this point it is not possible to specify the groups in advance.

## Additional Information

This systematic review will be conducted in line and with the same intention as the below listed systematic reviews previously conducted by our team [1–3].

## References

1. Kahlig P, Neumayr A, Paris DH. Louse-borne relapsing fever-A systematic review and analysis of the literature: Part 2-Mortality, Jarisch-Herxheimer reaction, impact on pregnancy. PLoS Negl Trop Dis. 2021;15: e0008656. doi:10.1371/journal.pntd.0008656

2. Kahlig P, Paris DH, Neumayr A. Louse-borne relapsing fever-A systematic review and analysis of the literature: Part 1-Epidemiology and diagnostic aspects. PLoS Negl Trop Dis. 2021;15: e0008564. doi:10.1371/journal.pntd.0008564

3. Jakab Á, Kahlig P, Kuenzli E, Neumayr A. Tick borne relapsing fever - a systematic review and analysis of the literature. PLoS Negl Trop Dis. 2022;16: e0010212. doi:10.1371/journal.pntd.0010212
