# Supplementary material for: Human granulocytotropic anaplasmosis—A systematic review and analysis of the literature
Source: PLoS Negl Trop Dis. 2024 Aug 5;18(8):e0012313. doi: 10.1371/journal.pntd.0012313 (PMC11326711; doi:10.1371/journal.pntd.0012313)
Supplement: S2 Text — (DOCX) [file pntd.0012313.s002.docx]

**List of databases with search term used**

- - **PubMed**

(Anaplasma[Mesh:NoExp] OR "Anaplasma phagocytophilum"[Mesh] OR "Anaplasma ovis"[Mesh] OR Anaplasmosis[Mesh] OR (Ehrlichiosis[Mesh] AND Granulocytes[Mesh])) OR (Anaplasma[tiab] OR Anaplasmas[tiab] OR "HGE Agent"[tiab] OR phagocytophil*[tiab] OR "E equi"[tiab] OR "Ehrlichia equi"[tiab] OR (Ehrlichia[tiab] AND granulocytotropic[tiab]) OR Anaplasmosis[tiab] OR Anaplasmoses[tiab] OR ((Ehrlichiosis[tiab] OR Ehrlichioses[tiab]) AND granulocyt*[tiab])) NOT (Animals[Mesh] NOT Humans[Mesh])

- **Cochrane Library**

Anaplasma:ti,ab OR Anaplasmas:ti,ab OR "HGE Agent":ti,ab OR phagocytophil*:ti,ab OR "E equi":ti,ab OR "Ehrlichia equi":ti,ab OR (Ehrlichia:ti,ab AND granulocytotropic:ti,ab) OR Anaplasmosis:ti,ab OR Anaplasmoses:ti,ab OR ((Ehrlichiosis:ti,ab OR Ehrlichioses:ti,ab) AND granulocyt*:ti,ab)

- **Embase (Elsevier)**

(Anaplasma/de OR 'Anaplasma phagocytophilum'/exp OR 'Anaplasma ovis'/exp OR 'Anaplasma platys'/exp OR Anaplasmosis/de OR 'human granulocytic anaplasmosis'/exp OR (Ehrlichiosis/exp AND Granulocytes/exp) OR 'human granulocytic anaplasmosis'/exp) OR (Anaplasma:ti,ab OR Anaplasmas:ti,ab OR 'HGE Agent':ti,ab OR phagocytophil*:ti,ab OR 'E equi':ti,ab OR 'Ehrlichia equi':ti,ab OR (Ehrlichia:ti,ab AND granulocytotropic:ti,ab) OR Anaplasmosis:ti,ab OR Anaplasmoses:ti,ab OR ((Ehrlichiosis:ti,ab OR Ehrlichioses:ti,ab) AND granulocyt*:ti,ab)) NOT (Animal/exp NOT Human/exp)

- **CINAHL (Ebsco)**

(TI Anaplasma OR AB Anaplasma) OR (TI Anaplasmas OR AB Anaplasmas) OR (TI "HGE Agent" OR AB "HGE Agent") OR (TI phagocytophil* OR AB phagocytophil*) OR (TI "E equi" OR AB "E equi") OR (TI "Ehrlichia equi" OR AB "Ehrlichia equi") OR ((TI Ehrlichia OR AB Ehrlichia) AND (TI granulocytotropic OR AB granulocytotropic)) OR (TI Anaplasmosis OR AB Anaplasmosis) OR (TI Anaplasmoses OR AB Anaplasmoses) OR (((TI Ehrlichiosis OR AB Ehrlichiosis) OR (TI Ehrlichioses OR AB Ehrlichioses)) AND (TI granulocyt* OR AB granulocyt*))

- **Web of Science of Science Collection**

TS= (Anaplasma OR Anaplasmas OR "HGE Agent" OR phagocytophil* OR "E equi" OR "Ehrlichia equi" OR (Ehrlichia AND granulocytotropic) OR Anaplasmosis OR Anaplasmoses OR ((Ehrlichiosis OR Ehrlichioses) AND granulocyt*)) AND (TS=Human OR TS=Humans)

- **Scopus**

( ( TITLE-ABS ( anaplasma ) OR TITLE-ABS ( anaplasmas ) OR TITLE-ABS ( "HGE Agent" ) OR TITLE-ABS ( phagocytophil* ) OR TITLE-ABS ( "E equi" ) OR TITLE-ABS ( "Ehrlichia equi" ) OR ( TITLE-ABS ( ehrlichia ) AND TITLE-ABS ( granulocytotropic ) ) OR TITLE-ABS ( anaplasmosis ) OR TITLE-ABS ( anaplasmoses ) OR ( ( TITLE-ABS ( ehrlichiosis ) OR TITLE-ABS ( ehrlichioses ) ) AND TITLE-ABS ( granulocyt* ) ) ) OR ( AUTHKEY ( anaplasma ) OR AUTHKEY ( anaplasmas ) OR AUTHKEY ( "HGE Agent" ) OR AUTHKEY ( phagocytophil* ) OR AUTHKEY ( "E equi" ) OR AUTHKEY ( "Ehrlichia equi" ) OR ( AUTHKEY ( ehrlichia ) AND AUTHKEY ( granulocytotropic ) ) OR AUTHKEY ( anaplasmosis ) OR AUTHKEY ( anaplasmoses ) OR ( ( AUTHKEY ( ehrlichiosis ) OR AUTHKEY ( ehrlichioses ) ) AND AUTHKEY ( granulocyt* ) ) ) ) AND ( TITLE-ABS ( human ) OR TITLE-ABS ( humans ) OR AUTHKEY (human) OR AUTHKEY (humans) )
