# Supplementary material for: Human granulocytotropic anaplasmosis—A systematic review and analysis of the literature
Source: PLoS Negl Trop Dis. 2024 Aug 5;18(8):e0012313. doi: 10.1371/journal.pntd.0012313 (PMC11326711; doi:10.1371/journal.pntd.0012313)
Supplement: S3 Text — (DOCX) [file pntd.0012313.s003.docx]

Included

1. Bakken JS, Dumler JS, Chen SM, Eckman MR, Van Etta LL, Walker DH. Human granulocytic ehrlichiosis in the upper Midwest United States. A new species emerging? Jama. 1994;272(3):212-8. Epub 1994/07/20. PubMed PMID: 8022040.

2. Hardalo CJ, Quagliarello V, Dumler JS. HUMAN GRANULOCYTIC EHRLICHIOSIS IN CONNECTICUT - REPORT OF A FATAL CASE. Clinical Infectious Diseases. 1995;21(4):910-4. doi: 10.1093/clinids/21.4.910. PubMed PMID: WOS:A1995RZ13900012.

3. Reed KD, Mitchell PD, Persing DH, Kolbert CP, Cameron V. Transmission of human granulocytic ehrlichiosis. Jama. 1995;273(1):23. Epub 1995/01/04. PubMed PMID: 7864987.

4. Shea KW, Calio AJ, Klein NC, Cunha BA. Rhabdomyolysis associated with Ehrlichia chaffeensis infection. Clin Infect Dis. 1995;21(4):1056-7. Epub 1995/10/01. doi: 10.1093/clinids/21.4.1056. PubMed PMID: 8645815.

5. Sumption KJ, Wright DJ, Cutler SJ, Dale BA. Human ehrlichiosis in the UK. Lancet. 1995;346(8988):1487-8. Epub 1995/12/02. doi: 10.1016/s0140-6736(95)92502-3. PubMed PMID: 7491006.

6. Telford SR, 3rd, Lepore TJ, Snow P, Warner CK, Dawson JE. Human granulocytic ehrlichiosis in Massachusetts. Ann Intern Med. 1995;123(4):277-9. Epub 1995/08/15. doi: 10.7326/0003-4819-123-4-199508150-00006. PubMed PMID: 7611594.

7. Aguero-Rosenfeld ME, Horowitz HW, Wormser GP, McKenna DF, Nowakowski J, Muñoz J, et al. Human granulocytic ehrlichiosis: a case series from a medical center in New York State. Ann Intern Med. 1996;125(11):904-8. Epub 1996/12/01. doi: 10.7326/0003-4819-125-11-199612010-00006. PubMed PMID: 8967671.

8. Ahkee S, Ramirez J. A case of concurrent Lyme meningitis with ehrlichiosis. Scand J Infect Dis. 1996;28(5):527-8. Epub 1996/01/01. doi: 10.3109/00365549609037953. PubMed PMID: 8953687.

9. Bakken JS, Krueth J, Wilson-Nordskog C, Tilden RL, Asanovich K, Dumler JS. Clinical and laboratory characteristics of human granulocytic ehrlichiosis. Jama. 1996;275(3):199-205. Epub 1996/01/17. PubMed PMID: 8604172.

10. Dumler JS, Bakken JS. Human granulocytic ehrlichiosis in Wisconsin and Minnesota: a frequent infection with the potential for persistence. J Infect Dis. 1996;173(4):1027-30. Epub 1996/04/01. doi: 10.1093/infdis/173.4.1027. PubMed PMID: 8603945.

11. Gewirtz AS, Cornbleet PJ, Vugia DJ, Traver C, Niederhuber J, Kolbert CP, et al. Human granulocytic ehrlichiosis: report of a case in Northern California. Clin Infect Dis. 1996;23(3):653-4. Epub 1996/09/01. doi: 10.1093/clinids/23.3.653. PubMed PMID: 8879806.

12. Goodman JL, Nelson C, Vitale B, Madigan JE, Dumler JS, Kurtti TJ, et al. Direct cultivation of the causative agent of human granulocytic ehrlichiosis. N Engl J Med. 1996;334(4):209-15. Epub 1996/01/25. doi: 10.1056/nejm199601253340401. PubMed PMID: 8531996.

13. Günthard HF, Péter O, Gubler J. Leukopenia and thrombocytopenia in a patient with early Lyme borreliosis. Clin Infect Dis. 1996;22(6):1119-20. Epub 1996/06/01. doi: 10.1093/clinids/22.6.1119. PubMed PMID: 8783732.

14. Horowitz HW, Marks SJ, Weintraub M, Dumler JS. Brachial plexopathy associated with human granulocytic ehrlichiosis. Neurology. 1996;46(4):1026-9. Epub 1996/04/01. doi: 10.1212/wnl.46.4.1026. PubMed PMID: 8780084.

15. Mazzella FM, Roman A, Perez A. A case of concurrent presentation of human ehrlichiosis and Lyme disease in Connecticut. Conn Med. 1996;60(9):515-9. Epub 1996/09/01. PubMed PMID: 8908776.

16. Shea KW, Calio AJ, Klein NC, Cunha BA. Ehrlichia equi infection associated with rhabdomyolysis. Clin Infect Dis. 1996;22(3):605. Epub 1996/03/01. doi: 10.1093/clinids/22.3.605-a. PubMed PMID: 8853010.

17. Adachi JA, Grimm EM, Johnson P, Uthman M, Kaplan B, Rakita RM. Human granulocytic ehrlichiosis in a renal transplant patient - Case report and review of the literature. Transplantation. 1997;64(8):1139-42. doi: 10.1097/00007890-199710270-00010. PubMed PMID: WOS:A1997YE23300010.

18. Arraga de Alvarado CM, Parra, O., Palmar, M., Chango, R., & Alvarado, M. C. Ehrlichia platys: Preparación del antígeno y uso de la técnica de inmunofluorescencia indirecta (IFI) en caninos y humanos. Revista Científica De La Facultad De Ciencias Veterinarias De La Universidad Del Zulia. 1997;7 (2).

19. Duffy J, Pittlekow MR, Kolbert CP, Rutledge BJ, Persing DH. Coinfection with Borrelia burgdorferi and the agent of human granulocytic ehrlichiosis. Lancet. 1997;349(9049):399. Epub 1997/02/08. doi: 10.1016/s0140-6736(97)80017-7. PubMed PMID: 9033471.

20. Nadelman RB, Horowitz HW, Hsieh TC, Wu JM, Aguero-Rosenfeld ME, Schwartz I, et al. Simultaneous human granulocytic ehrlichiosis and Lyme borreliosis. N Engl J Med. 1997;337(1):27-30. Epub 1997/07/03. doi: 10.1056/nejm199707033370105. PubMed PMID: 9203428.

21. Petrovec M, Lotric Furlan S, Zupanc TA, Strle F, Brouqui P, Roux V, et al. Human disease in Europe caused by a granulocytic Ehrlichia species. J Clin Microbiol. 1997;35(6):1556-9. Epub 1997/06/01. doi: 10.1128/jcm.35.6.1556-1559.1997. PubMed PMID: 9163481; PubMed Central PMCID: PMCPMC229786.

22. Wong SJ, Brady GS, Dumler JS. Serological responses to Ehrlichia equi, Ehrlichia chaffeensis, and Borrelia burgdorferi in patients from New York State. J Clin Microbiol. 1997;35(9):2198-205. Epub 1997/09/01. doi: 10.1128/jcm.35.9.2198-2205.1997. PubMed PMID: 9276387; PubMed Central PMCID: PMCPMC229939.

23. Wormser GP, Horowitz HW, Nowakowski J, McKenna D, Dumler JS, Varde S, et al. Positive Lyme disease serology in patients with clinical and laboratory evidence of human granulocytic ehrlichiosis. Am J Clin Pathol. 1997;107(2):142-7. Epub 1997/02/01. doi: 10.1093/ajcp/107.2.142. PubMed PMID: 9024062.

24. Bakken JS, Erlemeyer SA, Kanoff RJ, Silvestrini TC, 2nd, Goodwin DD, Dumler JS. Demyelinating polyneuropathy associated with human granulocytic ehrlichiosis. Clin Infect Dis. 1998;27(5):1323-4. Epub 1998/11/25. PubMed PMID: 9827293.

25. Buitrago MI, Ijdo JW, Rinaudo P, Simon H, Copel J, Gadbaw J, et al. Human granulocytic ehrlichiosis during pregnancy treated successfully with rifampin. Clin Infect Dis. 1998;27(1):213-5. Epub 1998/07/24. doi: 10.1086/517678. PubMed PMID: 9675481.

26. Horowitz HW, Aguero-Rosenfeld M, Horowitz HW, Aguero-Rosenfeld M, Dumler JS, McKenna DF, et al. Reinfection with the agent of human granulocytic ehrlichiosis. Annals of Internal Medicine. 1998;129(6):461-3. doi: 10.7326/0003-4819-129-6-199809150-00007. PubMed PMID: 1125855. Language: English. Entry Date: 19981001. Revision Date: 20191111. Publication Type: journal article.

27. Horowitz HW, Aguero-Rosenfeld ME, McKenna DF, Holmgren D, Hsieh TC, Varde SA, et al. Clinical and laboratory spectrum of culture-proven human granulocytic ehrlichiosis: comparison with culture-negative cases. Clin Infect Dis. 1998;27(5):1314-7. Epub 1998/11/25. doi: 10.1086/515000. PubMed PMID: 9827289.

28. Horowitz HW, Kilchevsky E, Haber S, Aguero-Rosenfeld M, Kranwinkel R, James EK, et al. Perinatal transmission of the agent of human granulocytic ehrlichiosis. N Engl J Med. 1998;339(6):375-8. Epub 1998/08/06. doi: 10.1056/nejm199808063390604. PubMed PMID: 9691104.

29. Horowitz HW, Raffalli J, Nadelman RB, Wu J, Wormser GP. Saddleback fever due to human granulocytic ehrlichiosis. Lancet. 1998;351(9103):650. Epub 1998/03/21. doi: 10.1016/s0140-6736(05)78434-8. PubMed PMID: 9500331.

30. Jahangir A, Kolbert C, Edwards W, Mitchell P, Dumler JS, Persing DH. Fatal pancarditis associated with human granulocytic Ehrlichiosis in a 44-year-old man. Clin Infect Dis. 1998;27(6):1424-7. Epub 1998/12/30. doi: 10.1086/515014. PubMed PMID: 9868655.

31. Lotric-Furlan S, Petrovec M, Avsic-Zupanc T, Nicholson WL, Sumner JW, Childs JE, et al. Human ehrlichiosis in central Europe. Wien Klin Wochenschr. 1998;110(24):894-7. Epub 1999/02/27. PubMed PMID: 10048172.

32. Lotric-Furlan S, Petrovec M, Zupanc TA, Nicholson WL, Sumner JW, Childs JE, et al. Human granulocytic ehrlichiosis in Europe: clinical and laboratory findings for four patients from Slovenia. Clin Infect Dis. 1998;27(3):424-8. Epub 1998/10/14. doi: 10.1086/514683. PubMed PMID: 9770134.

33. Weber R, Pusterla N, Loy M, Lutz H. Fever, leukopenia, and thrombocytopenia in a patient with acute Lyme borreliosis were due to human granulocytic ehrlichiosis. Clin Infect Dis. 1998;26(1):253-4. Epub 1998/02/10. doi: 10.1086/517052. PubMed PMID: 9455582.

34. Belongia EA, Reed KD, Mitchell PD, Chyou PH, Mueller-Rizner N, Finkel MF, et al. Clinical and epidemiological features of early Lyme disease and human granulocytic ehrlichiosis in Wisconsin. Clin Infect Dis. 1999;29(6):1472-7. Epub 1999/12/10. doi: 10.1086/313532. PubMed PMID: 10585798.

35. Carpenter CF, Gandhi TK, Kong LK, Corey GR, Chen SM, Walker DH, et al. The incidence of ehrlichial and rickettsial infection in patients with unexplained fever and recent history of tick bite in central North Carolina. J Infect Dis. 1999;180(3):900-3. Epub 1999/08/07. doi: 10.1086/314954. PubMed PMID: 10438390.

36. Comer JA, Nicholson WL, Olson JG, Childs JE. Serologic testing for human granulocytic ehrlichiosis at a national referral center. J Clin Microbiol. 1999;37(3):558-64. Epub 1999/02/13. doi: 10.1128/jcm.37.3.558-564.1999. PubMed PMID: 9986812; PubMed Central PMCID: PMCPMC84468.

37. Eastlund T, Persing D, Mathiesen D, Kim D, Bieging J, McCann P, et al. Human granulocytic ehrlichiosis after red cell transfusion. Transfusion. 1999;39(10):117S-S. PubMed PMID: WOS:000083207500555.

38. Foley JE, Crawford-Miksza L, Dumler JS, Glaser C, Chae JS, Yeh E, et al. Human granulocytic ehrlichiosis in Northern California: two case descriptions with genetic analysis of the Ehrlichiae. Clin Infect Dis. 1999;29(2):388-92. Epub 1999/09/07. doi: 10.1086/520220. PubMed PMID: 10476747.

39. George JC. About a first case of human granulocytic ehrlichiosis in France revealed by a summer flu-like syndrom. Revue du Praticien - Medecine Generale. 1999;13(475):1715-7.

40. Hossain D, Aguero-Rosenfeld ME, Horowitz HW, Wu JM, Hsieh TC, Sachdeva N, et al. Clinical and laboratory evolution of a culture-confirmed case of human granulocytic ehrlichiosis. Conn Med. 1999;63(5):265-70. Epub 1999/06/11. PubMed PMID: 10363404.

41. Keysary A, Amram L, Keren G, Sthoeger Z, Potasman I, Jacob A, et al. Serologic evidence of human monocytic and granulocytic ehrlichiosis in Israel. Emerg Infect Dis. 1999;5(6):775-8. Epub 1999/12/22. doi: 10.3201/eid0506.990605. PubMed PMID: 10603210; PubMed Central PMCID: PMCPMC2640796.

42. Laferl H, Hogrefe W, Köck T, Pichler H. A further case of acute human granulocytic ehrlichiosis in Slovenia. Eur J Clin Microbiol Infect Dis. 1999;18(5):385-6. Epub 1999/07/27. doi: 10.1007/pl00015026. PubMed PMID: 10421051.

43. Modi KS, Dahl DC, Berkseth RO, Schut R, Greeno E. Human granulocytic ehrlichiosis presenting with acute renal failure and mimicking thrombotic thrombocytopenic purpura. American Journal of Nephrology. 1999;19(6):677-81. doi: 10.1159/000013541.

44. van Dobbenburgh A, van Dam AP, Fikrig E, van Dobbenburgh A, van Dam AP, Fikrig E. Human granulocytic ehrlichiosis in western Europe. New England Journal of Medicine. 1999;340:1214-6. doi: 10.1056/NEJM199904153401517.

45. Gershel JC. Human granulocytic ehrlichiosis presenting as abdominal pain. Pediatrics. 2000;106(3):602-4. Epub 2000/09/02. doi: 10.1542/peds.106.3.602. PubMed PMID: 10969110.

46. Lee FS, Chu FK, Tackley M, Wu AD, Atri A, Wessels MR. Human granulocytic ehrlichiosis presenting as facial diplegia in a 42-year-old woman. Clin Infect Dis. 2000;31(5):1288-91. Epub 2000/11/14. doi: 10.1086/317466. PubMed PMID: 11073767.

47. Lepidi H, Bunnell JE, Martin ME, Madigan JE, Stuen S, Dumler JS. Comparative pathology, and immunohistology associated with clinical illness after Ehrlichia phagocytophila-group infections. Am J Trop Med Hyg. 2000;62(1):29-37. Epub 2000/04/13. doi: 10.4269/ajtmh.2000.62.29. PubMed PMID: 10761721.

48. Oteo J. First report of human granulocytic ehrlichiosis from southern Europe (Spain) (vol 6, pg 431, 2000). Emerging Infectious Diseases. 2000;6(6):663-. PubMed PMID: WOS:000165815700023.

49. Rabinstein A, Tikhomirov V, Kaluta A, Gelfmann N, Iannini P, Edwards L. Recurrent and prolonged fever in asplenic patients with human granulocytic ehrlichiosis. Qjm. 2000;93(3):198-201. Epub 2000/04/06. doi: 10.1093/qjmed/93.3.198-a. PubMed PMID: 10751242.

50. Weber R, Pusterla N, Loy M, Leutenegger CM, Schär G, Baumann D, et al. [Serologic and clinical evidence for endemic occurrences of human granulocytic ehrlichiosis in North-Eastern Switzerland]. Schweiz Med Wochenschr. 2000;130(41):1462-70. Epub 2000/11/15. PubMed PMID: 11075410.

51. Case records of the Massachusetts General Hospital. Weekly clinicopathological exercises. Case 37-2001. A 76-year-old man with fever, dyspnea, pulmonary infiltrates, pleural effusions, and confusion. N Engl J Med. 2001;345(22):1627-34. Epub 2002/01/05. doi: 10.1056/NEJMcpc3756515. PubMed PMID: 11757511.

52. Arnez M, Petrovec M, Lotric-Furlan S, Zupanc TA, Strle F. First European pediatric case of human granulocytic ehrlichiosis. J Clin Microbiol. 2001;39(12):4591-2. Epub 2001/11/29. doi: 10.1128/jcm.39.12.4591-4592.2001. PubMed PMID: 11724894; PubMed Central PMCID: PMCPMC88598.

53. Belongia EA, Gale CM, Reed KD, Mitchell PD, Vandermause M, Finkel MF, et al. Population-based incidence of human granulocytic ehrlichiosis in northwestern Wisconsin, 1997-1999. J Infect Dis. 2001;184(11):1470-4. Epub 2001/12/26. doi: 10.1086/324517. PubMed PMID: 11709792.

54. Karlsson U, Bjöersdorff A, Massung RF, Christensson B. Human granulocytic ehrlichiosis--a clinical case in Scandinavia. Scand J Infect Dis. 2001;33(1):73-4. Epub 2001/03/10. doi: 10.1080/003655401750064130. PubMed PMID: 11234985.

55. Lotric-Furlan S, Avsic-Zupanc T, Petrovec M, Nicholson WL, Sumner JW, Childs JE, et al. Clinical and serological follow-up of patients with human granulocytic ehrlichiosis in Slovenia. Clin Diagn Lab Immunol. 2001;8(5):899-903. Epub 2001/08/31. doi: 10.1128/cdli.8.5.899-903.2001. PubMed PMID: 11527800; PubMed Central PMCID: PMCPMC96168.

56. Trofe J, Reddy KS, Stratta RJ, Flax SD, Somerville KT, Alloway RR, et al. Human granulocytic ehrlichiosis in pancreas transplant recipients. Transpl Infect Dis. 2001;3(1):34-9. Epub 2001/06/29. doi: 10.1034/j.1399-3062.2001.003001034.x. PubMed PMID: 11429038.

57. Tylewska-Wierzbanowska S, Chmielewski T, Kondrusik M, Hermanowska-Szpakowicz T, Sawicki W, Sułek K. First cases of acute human granulocytic ehrlichiosis in Poland. Eur J Clin Microbiol Infect Dis. 2001;20(3):196-8. Epub 2001/05/12. doi: 10.1007/s100960100464. PubMed PMID: 11347671.

58. Bjöersdorff A, Wittesjö B, Berglun J, Massung RF, Eliasson I. Human granulocytic ehrlichiosis as a common cause of tick-associated fever in Southeast Sweden: report from a prospective clinical study. Scand J Infect Dis. 2002;34(3):187-91. Epub 2002/05/28. doi: 10.1080/00365540110080061. PubMed PMID: 12030391.

59. Casau NC, Hewins ME, Zaleznik DF. Treatment of human granulocytic ehrlichiosis during pregnancy and risk of perinatal transmission. Scand J Infect Dis. 2002;34(11):853-5. Epub 2003/02/13. doi: 10.1080/0036554021000026937. PubMed PMID: 12578161.

60. Hulinska D, Votypka J, Plch J, Vlcek E, Valesová M, Bojar M, et al. Molecular and microscopical evidence of Ehrlichia spp. and Borrelia burgdorferi sensu lato in patients, animals and ticks in the Czech Republic. New Microbiol. 2002;25(4):437-48. Epub 2002/11/20. PubMed PMID: 12437223.

61. Krause PJ, McKay K, Thompson CA, Sikand VK, Lentz R, Lepore T, et al. Disease-specific diagnosis of coinfecting tickborne zoonoses: babesiosis, human granulocytic ehrlichiosis, and Lyme disease. Clin Infect Dis. 2002;34(9):1184-91. Epub 2002/04/10. doi: 10.1086/339813. PubMed PMID: 11941544.

62. Manickaratnam S. Pigment induced renal failure in human granulocytic ehrlichiosis. Journal of General Internal Medicine. 2002;17:55-6. PubMed PMID: WOS:000175158200139.

63. Patel P, Mileno M. Granulocytic Ehrlichiosis in an HIV-positive patient. Infections in Medicine. 2002;19(1):39-42.

64. Vannorsdall MD, Thomas S, Smith RP, Zimmerman R, Christman R, Vella JP. Human granulocytic ehrlichiosis in a renal allograft recipient: review of the clinical spectrum of disease in solid organ transplant patients. Transpl Infect Dis. 2002;4(2):97-101. Epub 2002/09/11. doi: 10.1034/j.1399-3062.2002.01015.x. PubMed PMID: 12220247.

65. Anliker MD, Wüthrich B. Acute urticaria and angioedema due to ehrlichiosis. Dermatology. 2003;207(4):417-8. Epub 2003/12/06. doi: 10.1159/000074130. PubMed PMID: 14657642.

66. Baumann D, Pusterla N, Péter O, Grimm F, Fournier PE, Schär G, et al. [Fever after a tick bite: clinical manifestations and diagnosis of acute tick bite-associated infections in northeastern Switzerland]. Dtsch Med Wochenschr. 2003;128(19):1042-7. Epub 2003/05/09. doi: 10.1055/s-2003-39103. PubMed PMID: 12736854.

67. Homann CN, Feichtinger M, Santner B, Crevenna R, Homann B, Wenzel K, et al. Neuro-Ehrlichiosis: A new tick-borne disease with nervous system involvement? Ceska a Slovenska Neurologie a Neurochirurgie. 2003;66(6):420-6.

68. Krause PJ, Corrow CL, Bakken JS. Successful treatment of human granulocytic ehrlichiosis in children using rifampin. Pediatrics. 2003;112(3 Pt 1):e252-3. Epub 2003/09/02. doi: 10.1542/peds.112.3.e252. PubMed PMID: 12949322.

69. Lee MS, Goslee TE, Lessell S. Ehrlichiosis optic neuritis. American Journal of Ophthalmology. 2003;135(3):412-3. doi: 10.1016/s0002-9394(02)01945-1. PubMed PMID: WOS:000181258100034.

70. Low A, Turett G. Human granulocytic ehrlichiosis presenting as acute abdomen in an adult. Clin Infect Dis. 2003;37(10):1397-8. Epub 2003/10/30. doi: 10.1086/379129. PubMed PMID: 14583879.

71. Moss WJ, Dumler JS. Simultaneous infection with Borrelia burgdorferi and human granulocytic ehrlichiosis. Pediatr Infect Dis J. 2003;22(1):91-2. Epub 2003/01/30. doi: 10.1097/00006454-200301000-00023. PubMed PMID: 12553302.

72. Prükk T, Ainsalu K, Laja E, Aigro A. Human granulocytic ehrlichiosis in Estonia. Emerg Infect Dis. 2003;9(11):1499-500. Epub 2004/01/17. doi: 10.3201/eid0911.030480. PubMed PMID: 14725264; PubMed Central PMCID: PMCPMC3035530.

73. Remy V, Hansmann Y, De Martino S, Christmann D, Brouqui P. Human anaplasmosis presenting as atypical pneumonitis in France. Clin Infect Dis. 2003;37(6):846-8. Epub 2003/09/05. doi: 10.1086/377502. PubMed PMID: 12955649.

74. Ruscio M, Cinco M. Human granulocytic ehrlichiosis in Italy - First report on two confirmed cases. In: Hechemy KE, AvsicZupanc T, Childs JE, Raoult DA, editors. Rickettsiology: Present and Future Directions. Annals of the New York Academy of Sciences. 9902003. p. 350-2.

75. Springer SA, Altice FL. Human immunodeficiency virus infection with human granulocytic ehrlichiosis complicated by symptomatic lactic acidosis. Clin Infect Dis. 2003;36(12):e162-4. Epub 2003/06/13. doi: 10.1086/374930. PubMed PMID: 12802782.

76. Walder G, Falkensammer B, Aigner J, Tiwald G, Dierich MP, Würzner R, et al. First documented case of human granulocytic ehrlichiosis in Austria. Wien Klin Wochenschr. 2003;115(7-8):263-6. Epub 2003/06/05. doi: 10.1007/bf03040326. PubMed PMID: 12778780.

77. Walder G, Tiwald G, Dierich MP, Würzner R. Serological evidence for human granulocytic ehrlichiosis in Western Austria. Eur J Clin Microbiol Infect Dis. 2003;22(9):543-7. Epub 2003/08/26. doi: 10.1007/s10096-003-0986-3. PubMed PMID: 12938008.

78. Bayard-Mc Neeley M, Bansal A, Chowdhury I, Girao G, Small CB, Seiter K, et al. In vivo and in vitro studies on Anaplasma phagocytophilum infection of the myeloid cells of a patient with chronic myelogenous leukaemia and human granulocytic ehrlichiosis. J Clin Pathol. 2004;57(5):499-503. Epub 2004/04/29. doi: 10.1136/jcp.2003.011775. PubMed PMID: 15113857; PubMed Central PMCID: PMCPMC1770287.

79. Grzeszczuk A, Puzanowska B, Miegoć H, Prokopowicz D. Incidence and prevalence of infection with Anaplasma phagocytophilum. Prospective study in healthy individuals exposed to ticks. Ann Agric Environ Med. 2004;11(1):155-7. Epub 2004/07/09. PubMed PMID: 15236514.

80. Hermanowska-Szpakowicz T, Skotarczak B, Kondrusik M, Rymaszewska A, Sawczuk M, Maciejewska A, et al. Detecting DNAs of Anaplasma phagocytophilum and Babesia in the blood of patients suspected of Lyme disease. Ann Agric Environ Med. 2004;11(2):351-4. Epub 2005/01/04. PubMed PMID: 15627349.

81. Rosenberg R. Human granulocytic ehrlichiosis: "out of the Lyme light". Conn Med. 2004;68(4):195-7. Epub 2004/04/21. PubMed PMID: 15095825.

82. Batsis JA, Uslan DZ, Baddour LM. 70-year-old man with fever, shaking chills, and weakness. Mayo Clin Proc. 2005;80(9):1209-12. Epub 2005/09/24. doi: 10.4065/80.9.1209. PubMed PMID: 16178501.

83. de la Fuente J, Torina A, Naranjo V, Caracappa S, Di Marco V, Alongi A, et al. Infection with Anaplasma phagocytophilum in a seronegative patient in Sicily, Italy: case report. Ann Clin Microbiol Antimicrob. 2005;4:15. Epub 2005/10/06. doi: 10.1186/1476-0711-4-15. PubMed PMID: 16202127; PubMed Central PMCID: PMCPMC1253508.

84. Fritz CL, Bronson LR, Smith CR, Crawford-Miksza L, Yeh E, Schnurr D. Clinical, epidemiologic, and environmental surveillance for ehrlichiosis and anaplasmosis in an endemic area of northern California. J Vector Ecol. 2005;30(1):4-10. Epub 2005/07/13. PubMed PMID: 16007950.

85. Halasz CL, Niedt GW, Kurtz CP, Scorpio DG, Bakken JS, Dumler JS. A case of Sweet syndrome associated with human granulocytic anaplasmosis. Arch Dermatol. 2005;141(7):887-9. Epub 2005/07/20. doi: 10.1001/archderm.141.7.887. PubMed PMID: 16027306.

86. Heller HM, Telford SR, Branda JA, Harris NL, Goodson JD, Ryan ET, et al. A Man with weakness and pain in the legs - Human granulocytic ehrlichiosis (A-phagocytophilum infection). New England Journal of Medicine. 2005;352(13):1358-64. doi: 10.1056/NEJMcpc059004. PubMed PMID: WOS:000227998300014.

87. Lotric-Furlan S, Petrovec M, Avsic-Zupanc T, Strle F. Concomitant tickborne encephalitis and human granulocytic ehrlichiosis. Emerg Infect Dis. 2005;11(3):485-8. Epub 2005/03/11. doi: 10.3201/eid1103.040776. PubMed PMID: 15757574; PubMed Central PMCID: PMCPMC3298262.

88. Malik A, Jameel MN, Ali SS, Mir S. Human granulocytic anaplasmosis affecting the myocardium. J Gen Intern Med. 2005;20(10):C8-10. Epub 2005/09/30. doi: 10.1111/j.1525-1497.2005.0218_4.x. PubMed PMID: 16191146; PubMed Central PMCID: PMCPMC1490240.

89. Afanasieva MV, Vorobyeva NN, Korenberg EI, Frizen VI. Human granulocytic anaplasmosis: Risk in the Cisural region, Russia. International Journal of Medical Microbiology. 2006;296:167-8. doi: 10.1016/j.ijmm.2006.01.047. PubMed PMID: WOS:000238550200032.

90. Beltrame A, Ruscio M, Arzese A, Rorato G, Negri C, Londero A, et al. Human granulocytic anaplasmosis in Northeastern Italy. In: Hechemy KE, Oteo JA, Raoult DA, Silverman DJ, Blanco JR, editors. Century of Rickettsiology: Emerging, Reemerging Rickettsioses, Molecular Diagnostics, and Emerging Veterinary Rickettsioses. 1. 10782006. p. 106-9.

91. Garcia JC, Nunez MJ, Castro B, Fraile FJ, Lopez A, Mella MC, et al. Human anaplasmosis - The first Spanish case confirmed by PCR. In: Hechemy KE, Oteo JA, Raoult DA, Silverman DJ, Blanco JR, editors. Century of Rickettsiology: Emerging, Reemerging Rickettsioses, Molecular Diagnostics, and Emerging Veterinary Rickettsioses. Annals of the New York Academy of Sciences. 10782006. p. 545-7.

92. Grzeszczuk A, Ziarko S, Kovalchuk O, Stańczak J. Etiology of tick-borne febrile illnesses in adult residents of North-Eastern Poland: report from a prospective clinical study. Int J Med Microbiol. 2006;296 Suppl 40:242-9. Epub 2006/03/15. doi: 10.1016/j.ijmm.2006.01.007. PubMed PMID: 16530481.

93. Kowalski J, Hopfenmüller W, Fingerle V, Malberg H, Eisenblätter M, Wagner J, et al. Seroprevalence of human granulocytic anaplasmosis in Berlin/Brandenburg, Germany: an 8-year survey. Clin Microbiol Infect. 2006;12(9):924-7. Epub 2006/08/03. doi: 10.1111/j.1469-0691.2006.01490.x. PubMed PMID: 16882301.

94. Loebermann M, Fingerle V, Lademann M, Fritzsche C, Reisinger EC. Borrelia burgdorferi and Anaplasma phagocytophilum coinfection. Emerg Infect Dis. 2006;12(2):353-5. Epub 2006/11/04. doi: 10.3201/eid1202.050765. PubMed PMID: 17080581; PubMed Central PMCID: PMCPMC3373081.

95. Mastrandrea S, Mura MS, Tola S, Patta C, Tanda A, Porcu R, et al. Two cases of human granulocytic ehrlichiosis in Sardinia, Italy confirmed by PCR. Ann N Y Acad Sci. 2006;1078:548-51. Epub 2006/11/23. doi: 10.1196/annals.1374.107. PubMed PMID: 17114774.

96. Santos AS, Bacellar F, Dumler JS. Human exposure to Anaplasma phagocytophilum in Portugal. Ann N Y Acad Sci. 2006;1078:100-5. Epub 2006/11/23. doi: 10.1196/annals.1374.014. PubMed PMID: 17114687.

97. Walder G, Fuchs D, Sarcletti M, Berek K, Falkensammer B, Huber K, et al. Human granulocytic anaplasmosis in Austria: epidemiological, clinical, and laboratory findings in five consecutive patients from Tyrol, Austria. Int J Med Microbiol. 2006;296 Suppl 40:297-301. Epub 2006/03/15. doi: 10.1016/j.ijmm.2005.12.001. PubMed PMID: 16531117.

98. Walder G, Lkhamsuren E, Shagdar A, Bataa J, Batmunkh T, Orth D, et al. Serological evidence for tick-borne encephalitis, borreliosis, and human granulocytic anaplasmosis in Mongolia. Int J Med Microbiol. 2006;296 Suppl 40:69-75. Epub 2006/03/10. doi: 10.1016/j.ijmm.2006.01.031. PubMed PMID: 16524782.

99. Wormser GP, Filozov A, Telford SR, 3rd, Utpat S, Kamer RS, Liveris D, et al. Dissociation between inhibition and killing by levofloxacin in human granulocytic anaplasmosis. Vector Borne Zoonotic Dis. 2006;6(4):388-94. Epub 2006/12/26. doi: 10.1089/vbz.2006.6.388. PubMed PMID: 17187574.

100. Assi MA, Yao JD, Walker RC. Lyme disease followed by human granulocytic anaplasmosis in a kidney transplant recipient. Transpl Infect Dis. 2007;9(1):66-72. Epub 2007/02/23. doi: 10.1111/j.1399-3062.2006.00177.x. PubMed PMID: 17313478.

101. Boateng F, Ohene-Adjei R, Amoateng-Adjepong Y. Rhabdomyolysis and acute renal failure associated with human granulocytic amaplasmosis [1]. Mayo Clinic Proceedings. 2007;82(2):250. doi: 10.4065/82.2.250.

102. Byrnes V, Chopra S, Koziel MJ. Resolution of chronic hepatitis C following parasitosis. World J Gastroenterol. 2007;13(31):4268-9. Epub 2007/08/19. doi: 10.3748/wjg.v13.i31.4268. PubMed PMID: 17696260; PubMed Central PMCID: PMCPMC4250630.

103. De Raedt S, Antoine S, Ebinger G. Lymphocytic meningitis in human granulocytic anaplasmosis: A case report. European Journal of Neurology. 2007;14:113-. PubMed PMID: WOS:000250519300369.

104. Dhand A, Nadelman RB, Aguero-Rosenfeld M, Haddad FA, Stokes DP, Horowitz HW. Human granulocytic anaplasmosis during pregnancy: case series and literature review. Clin Infect Dis. 2007;45(5):589-93. Epub 2007/08/09. doi: 10.1086/520659. PubMed PMID: 17682993.

105. Dumler JS, Barat NC, Barat CE, Bakken JS. Human granulocytic anaplasmosis and macrophage activation. Clin Infect Dis. 2007;45(2):199-204. Epub 2007/06/21. doi: 10.1086/518834. PubMed PMID: 17578779.

106. Narasimhamurthy MA, Peart M, Hart P. Human granulocytic anaplasmosis (Ehrlichiosis) masquerading as thrombotic thrombocytopenic purpura. Journal of General Internal Medicine. 2007;22:243-. PubMed PMID: WOS:000251610700847.

107. Peris-Garcia J, Cuadrado-Pastor JM, Jover-Diaz F, Batas-Velasco M. Probable case of imported human anaplasmosis. Enfermedades Infecciosas Y Microbiologia Clinica. 2007;25(10):656-7. doi: 10.1157/13112945. PubMed PMID: WOS:000252553800011.

108. Young NP, Klein CJ. Encephalopathy with seizures having PCR-positive Anaplasma phagocytophilum and Ehrlichia chaffeensis. Eur J Neurol. 2007;14(2):e3-4. Epub 2007/01/26. doi: 10.1111/j.1468-1331.2006.01582.x. PubMed PMID: 17250712.

109. Anaplasma phagocytophilum transmitted through blood transfusion--Minnesota, 2007. MMWR Morb Mortal Wkly Rep. 2008;57(42):1145-8. Epub 2008/10/24. PubMed PMID: 18946461.

110. Florescu D, Sordillo PP, Glyptis A, Zlatanic E, Smith B, Polsky B, et al. Splenic infarction in human babesiosis: Two cases and discussion. Clinical Infectious Diseases. 2008;46(1):e8-e11. doi: 10.1086/524081.

111. Kocianová E, Kost'anová Z, Stefanidesová K, Spitalská E, Boldis V, Hucková D, et al. Serologic evidence of Anaplasma phagocytophilum infections in patients with a history of tick bite in central Slovakia. Wien Klin Wochenschr. 2008;120(13-14):427-31. Epub 2008/08/30. doi: 10.1007/s00508-008-1000-y. PubMed PMID: 18726669.

112. Muffly T, McCormick TC, Cook C, Wall J. Human granulocytic ehrlichiosis complicating early pregnancy. Infect Dis Obstet Gynecol. 2008;2008:359172. Epub 2008/05/30. doi: 10.1155/2008/359172. PubMed PMID: 18509484; PubMed Central PMCID: PMCPMC2396214.

113. Psaroulaki A, Koliou M, Chochlakis D, Ioannou I, Mazeri S, Tselentis Y. Anaplasma phagocytophilum infection in a child. Pediatr Infect Dis J. 2008;27(7):664-6. Epub 2008/06/10. doi: 10.1097/INF.0b013e31816a0606. PubMed PMID: 18536621.

114. Torina A, Alongi A, Naranjo V, Scimeca S, Nicosia S, Di Marco V, et al. Characterization of anaplasma infections in Sicily, Italy. Ann N Y Acad Sci. 2008;1149:90-3. Epub 2009/01/06. doi: 10.1196/annals.1428.065. PubMed PMID: 19120181.

115. Zhang L, Liu Y, Ni D, Li Q, Yu Y, Yu XJ, et al. Nosocomial transmission of human granulocytic anaplasmosis in China. Jama. 2008;300(19):2263-70. Epub 2008/11/20. doi: 10.1001/jama.2008.626. PubMed PMID: 19017912.

116. Anaplasmosis and ehrlichiosis - Maine, 2008. MMWR Morb Mortal Wkly Rep. 2009;58(37):1033-6. Epub 2009/09/26. PubMed PMID: 19779398.

117. Chochlakis D, Koliou M, Ioannou I, Tselentis Y, Psaroulaki A. Kawasaki disease and Anaplasma sp. infection of an infant in Cyprus. Int J Infect Dis. 2009;13(2):e71-3. Epub 2008/10/14. doi: 10.1016/j.ijid.2008.08.001. PubMed PMID: 18848483.

118. Chochlakis D, Psaroulaki A, Kokkini S, Kostanatis S, Arkalati E, Karagrannaki E, et al. First evidence of Anaplasma infection in Crete, Greece. Report of six human cases. Clin Microbiol Infect. 2009;15 Suppl 2:8-9. Epub 2010/07/10. doi: 10.1111/j.1469-0691.2008.02695.x. PubMed PMID: 20584160.

119. Donato AA, Chaudhary A. A 78-year-old man with the "summer flu" and cytopenias. Clin Infect Dis. 2009;48(10):1433, 79-80. Epub 2009/04/21. doi: 10.1086/598325. PubMed PMID: 19374559.

120. Hulínská D, Votýpka J, Vanousová D, Hercogová J, Hulínský V, Drevová H, et al. Identification of Anaplasma phagocytophilum and Borrelia burgdorferi sensu lato in patients with erythema migrans. Folia Microbiol (Praha). 2009;54(3):246-56. Epub 2009/08/04. doi: 10.1007/s12223-009-0039-0. PubMed PMID: 19649743.

121. Jensenius M, Davis X, von Sonnenburg F, Schwartz E, Keystone JS, Leder K, et al. Multicenter GeoSentinel analysis of rickettsial diseases in international travelers, 1996-2008. Emerg Infect Dis. 2009;15(11):1791-8. Epub 2009/11/07. doi: 10.3201/eid1511.090677. PubMed PMID: 19891867; PubMed Central PMCID: PMCPMC2857242.

122. Kim BB, Carey JM, Yancovitz SR, Fische HD. Development of human granulocytic anaplasmosis infection despite prophylaxis with a one-time dose of oral doxycycline after recent tick exposure. Infectious Diseases in Clinical Practice. 2009;17(3):184-6. doi: 10.1097/IPC.0b013e31817e5c03.

123. Lotric-Furlan S, Ruzic-Sabljic E, Strle F. Concomitant human granulocytic anaplasmosis and Lyme neuroborreliosis. Clin Microbiol Infect. 2009;15 Suppl 2:28-9. Epub 2009/05/07. doi: 10.1111/j.1469-0691.2008.02145.x. PubMed PMID: 19416290.

124. Ouyang J, Zhang Q, Xu J, Chen B, Zhou RF. Human granulocytic anaplasmosis: A case report and review of literature. Blood. 2009;114(22).

125. Parkins MD, Church DL, Jiang XY, Gregson DB. Human granulocytic anaplasmosis: First reported case in Canada. Can J Infect Dis Med Microbiol. 2009;20(3):e100-2. Epub 2010/09/03. doi: 10.1155/2009/124173. PubMed PMID: 20808448; PubMed Central PMCID: PMCPMC2770309.

126. Patnaik MM. Inclusion bodies in tick-borne diseases diagnosed in patients from Northern Wisconsin. Clinical Medicine and Research. 2009;7(1-2):45-7. doi: 10.3121/cmr.2009.807.818.

127. Dimosthenis Chochlakis II, Yannis Tselentis, and Anna Psaroulaki. Human anaplasmosis and Anaplasma ovis variant. Emerging Infectious Diseases. 2010;16:1031-2. doi: 10.3201/eid1606.090175.

128. Lagac Wiens P. Neutrophilic inclusions in a hunter. Clin Infect Dis. 2010;51(9):1073, 102-3. Epub 2010/10/12. doi: 10.1086/656621. PubMed PMID: 20925504.

129. Novakova M, Vichova B, Majlathova V, Lesnakova A, Pochybova M, Pet'ko B. FIRST CASE OF HUMAN GRANULOCYTIC ANAPLASMOSIS FROM SLOVAKIA. Annals of Agricultural and Environmental Medicine. 2010;17(1):173-5. PubMed PMID: WOS:000279504800023.

130. Vogl UM, Presterl E, Stanek G, Ramharter M, Gattringer KB, Graninger W. First described case of human granulocytic anaplasmosis in a patient in Eastern Austria. Wien Med Wochenschr. 2010;160(3-4):91-3. Epub 2010/03/20. doi: 10.1007/s10354-009-0733-1. PubMed PMID: 20300926.

131. Welc-Falęciak R, Hildebrandt A, Siński E. Co-infection with Borrelia species and other tick-borne pathogens in humans: two cases from Poland. Ann Agric Environ Med. 2010;17(2):309-13. Epub 2010/12/29. PubMed PMID: 21186774.

132. Cochez C, Ducoffre G, Vandenvelde C, Luyasu V, Heyman P. Human anaplasmosis in Belgium: a 10-year seroepidemiological study. Ticks Tick Borne Dis. 2011;2(3):156-9. Epub 2011/09/06. doi: 10.1016/j.ttbdis.2011.06.004. PubMed PMID: 21890069.

133. Dahlgren FS, Mandel EJ, Krebs JW, Massung RF, McQuiston JH. Increasing incidence of Ehrlichia chaffeensis and Anaplasma phagocytophilum in the United States, 2000-2007. Am J Trop Med Hyg. 2011;85(1):124-31. Epub 2011/07/08. doi: 10.4269/ajtmh.2011.10-0613. PubMed PMID: 21734137; PubMed Central PMCID: PMCPMC3122356.

134. Ghafar MW, Eltablawy NA. Molecular survey of five tick-borne pathogens (Ehrlichia chaffeensis, Ehrlichia ewingii, Anaplasma phagocytophilum, Borrelia burgdorferi sensu lato and Babesia microti) in Egyptian farmers. Global Veterinaria. 2011;7(3):249-55.

135. Ghera P, Kasirye Y, Choudhry MW, Shaw GR, Ejercito VS. Acute transient sensorineural hearing loss due to Anaplasma phagocytophilum. Wmj. 2011;110(6):288-90. Epub 2012/02/14. PubMed PMID: 22324206.

136. Kallick CA. Ehrlichia and bone marrow cells: could Ehrlichial infection explain the unsuspected etiology of some diseases of the immune system? Med Hypotheses. 2011;77(3):374-9. Epub 2011/06/15. doi: 10.1016/j.mehy.2011.05.019. PubMed PMID: 21669495.

137. Kanjilal S, Brutsaert E, Markoff B. Anaplasmosis: A case report and literature review. Journal of Hospital Medicine. 2011;6(4):S197. doi: 10.1002/jhm.920.

138. Levicnik Stezinar S, Rahne Potokar U, Avsic Zupanc T, Jereb M. Transfusion transmitted human granulocytic ehrlichiosis. Vox Sanguinis. 2011;101:234. doi: 10.1111/j.1423-0410.2011.01498-2.x.

139. Li H, Zhou Y, Wang W, Guo D, Huang S, Jie S. The clinical characteristics and outcomes of patients with human granulocytic anaplasmosis in China. Int J Infect Dis. 2011;15(12):e859-66. Epub 2011/10/22. doi: 10.1016/j.ijid.2011.09.008. PubMed PMID: 22015246.

140. Liu QH. Pay attention to differential diagnosis of anaplasmosis with thrombocytopenic syndrome. International Journal of Infectious Diseases. 2011;15:S112. doi: 10.1016/S1201-9712(11)60391-3.

141. Lovrich SD, Jobe DA, Kowalski TJ, Policepatil SM, Callister SM. Expansion of the Midwestern focus for human granulocytic anaplasmosis into the region surrounding La Crosse, Wisconsin. J Clin Microbiol. 2011;49(11):3855-9. Epub 2011/09/16. doi: 10.1128/jcm.05025-11. PubMed PMID: 21918026; PubMed Central PMCID: PMCPMC3209085.

142. Qasba N, Shamshirsaz AA, Feder HM, Campbell WA, Egan JF, Shamshirsaz AA. A case report of human granulocytic anaplasmosis (ehrlichiosis) in pregnancy and a literature review of tick-borne diseases in the United States during pregnancy. Obstet Gynecol Surv. 2011;66(12):788-96. Epub 2011/12/24. doi: 10.1097/OGX.0b013e31823e0d0e. PubMed PMID: 22192463.

143. Singh A, Kim K, Reilly R, Saraiya N. A case of ehrlichiosis presenting as neutropenic fever with typhilitis. Journal of Hospital Medicine. 2011;6(4):S251. doi: 10.1002/jhm.920.

144. Stanojevic D, De Nazareth AG, Wichman T. A man with generalized fatigue and malaise. Journal of General Internal Medicine. 2011;26:S551. doi: 10.1007/s11606-011-1730-9.

145. Talsness SR, Shukla SK, Mazza JJ, Yale SH. Rhabdomyolysis-induced acute kidney injury secondary to Anaplasma phagocytophilum and concomitant statin use. Wmj. 2011;110(2):82-4. Epub 2011/05/13. PubMed PMID: 21560563.

146. Tsibris AMN, Shepard JAO, Zukerberg LR. Case 6-2011: A 77-year-old man with dyspnea, weakness, and diaphoresis. New England Journal of Medicine. 2011;364(8):759-67. doi: 10.1056/NEJMcpc1011320.

147. Zhang L, Cui F, Wang L, Zhang L, Zhang J, Wang S, et al. Investigation of anaplasmosis in Yiyuan County, Shandong Province, China. Asian Pac J Trop Med. 2011;4(7):568-72. Epub 2011/08/02. doi: 10.1016/s1995-7645(11)60148-x. PubMed PMID: 21803311.

148. Annen K, Friedman K, Eshoa C, Horowitz M, Gottschall J, Straus T. Two cases of transfusion-transmitted Anaplasma phagocytophilum. Am J Clin Pathol. 2012;137(4):562-5. Epub 2012/03/21. doi: 10.1309/ajcp4e4vqqqoziaq. PubMed PMID: 22431531.

149. Bellone M, Chiang J, Ahmed T, Galanakis D, Senzel L. Thrombotic thrombocytopenic purpura and its look-alikes: a single institution experience. Transfus Apher Sci. 2012;46(1):59-64. Epub 2011/12/14. doi: 10.1016/j.transci.2011.11.003. PubMed PMID: 22154043.

150. Dorn HF, Dickinson B, Agarwal A, Brayman KL. Human ehrlichiosis after treatment of acute cellular rejection in a kidney transplant patient. Transplantation. 2012;94:531.

151. Jereb M, Pecaver B, Tomazic J, Muzlovic I, Avsic-Zupanc T, Premru-Srsen T, et al. Severe human granulocytic anaplasmosis transmitted by blood transfusion. Emerg Infect Dis. 2012;18(8):1354-7. Epub 2012/07/31. doi: 10.3201/eid1808.120180. PubMed PMID: 22841007; PubMed Central PMCID: PMCPMC3414041.

152. Koebel C, Kern A, Edouard S, Hoang AT, Celestin N, Hansmann Y, et al. Human granulocytic anaplasmosis in eastern France: clinical presentation and laboratory diagnosis. Diagn Microbiol Infect Dis. 2012;72(3):214-8. Epub 2012/02/11. doi: 10.1016/j.diagmicrobio.2011.12.005. PubMed PMID: 22321996.

153. Liu Y, Li Q, Hu W, Wu J, Wang Y, Mei L, et al. Person-to-person transmission of severe fever with thrombocytopenia syndrome virus. Vector Borne Zoonotic Dis. 2012;12(2):156-60. Epub 2011/10/01. doi: 10.1089/vbz.2011.0758. PubMed PMID: 21955213.

154. Pozdnyakova O, Dorfman DM. Human granulocytic anaplasmosis. Blood. 2012;120(25):4911. Epub 2013/02/01. doi: 10.1182/blood-2012-07-445817. PubMed PMID: 23367517.

155. Temtanakitpaisan Y, Methachittiphan N, Barbosa FB, Treadwell TL, Love DG, editors. Cardiac involvement in human granulocytic anaplasmosis. 17th World Congress on Heart Disease / Annual Scientific Sessions of the International-Academy-of-Cardiology

https://cardiologyonlinecom/wchd12/indexhtml; 2012 Jul 27-30; Toronto, CANADA2012.

156. Weil AA, Baron EL, Brown CM, Drapkin MS. Clinical findings and diagnosis in human granulocytic anaplasmosis: a case series from Massachusetts. Mayo Clin Proc. 2012;87(3):233-9. Epub 2012/03/06. doi: 10.1016/j.mayocp.2011.09.008. PubMed PMID: 22386178; PubMed Central PMCID: PMCPMC3498394.

157. Alhumaidan H, Westley B, Esteva C, Berardi V, Young C, Sweeney J. Transfusion-transmitted anaplasmosis from leukoreduced red blood cells. Transfusion. 2013;53(1):181-6. Epub 2012/05/09. doi: 10.1111/j.1537-2995.2012.03685.x. PubMed PMID: 22563784.

158. Bautista MT, Sharma R, Orenstein A, Circeo L. Myocardial dysfunction and shock from human granulocytic anaplasmosis (HGA): An unusual presentation. Critical Care Medicine. 2013;41(12):A295-A6. doi: 10.1097/01.ccm.0000440398.71873.ea.

159. George S, Sandal S. Images in clinical medicine. A ticking time bomb. N Engl J Med. 2013;368(19):1826. Epub 2013/05/10. doi: 10.1056/NEJMicm1214469. PubMed PMID: 23656648.

160. Horowitz HW, Aguero-Rosenfeld ME, Holmgren D, McKenna D, Schwartz I, Cox ME, et al. Lyme disease and human granulocytic anaplasmosis coinfection: impact of case definition on coinfection rates and illness severity. Clin Infect Dis. 2013;56(1):93-9. Epub 2012/10/09. doi: 10.1093/cid/cis852. PubMed PMID: 23042964.

161. Joshi M, Suresh SC, Shaikh IA, Rana Z, Scholand SJ. Tick-borne trauma: an unusual presentation of anaplasmosis. Conn Med. 2013;77(7):417-20. Epub 2013/11/08. PubMed PMID: 24195180.

162. Koff G, Sellers J, Oxman D. Anaplasmosis and ARDS. Critical Care Medicine. 2013;41(12):A316. doi: 10.1097/01.ccm.0000440463.93851.02.

163. Lindblom A, Wallménius K, Nordberg M, Forsberg P, Eliasson I, Påhlson C, et al. Seroreactivity for spotted fever rickettsiae and co-infections with other tick-borne agents among habitants in central and southern Sweden. Eur J Clin Microbiol Infect Dis. 2013;32(3):317-23. Epub 2012/09/11. doi: 10.1007/s10096-012-1742-3. PubMed PMID: 22961007; PubMed Central PMCID: PMCPMC3569577.

164. Maczka I, Roguska U, Tylewska-Wierzbanowska S. Prevalence of rickettsioses in Poland in 2006-2012. Przegl Epidemiol. 2013;67(4):633-6, 721-3. Epub 2013/01/01. PubMed PMID: 24741909.

165. Maggi RG, Mascarelli PE, Havenga LN, Naidoo V, Breitschwerdt EB. Co-infection with Anaplasma platys, Bartonella henselae and Candidatus Mycoplasma haematoparvum in a veterinarian. Parasit Vectors. 2013;6:103. Epub 2013/04/17. doi: 10.1186/1756-3305-6-103. PubMed PMID: 23587235; PubMed Central PMCID: PMCPMC3637287.

166. Mir MA, Grant J. Dysarthria and thrombocytopenia after tick bite. Blood. 2013;122(15):2538. Epub 2013/11/23. doi: 10.1182/blood-2013-06-506410. PubMed PMID: 24266032.

167. Nall R, Comstock P. Fatigue and second degree AV block in a young healthy male. Journal of General Internal Medicine. 2013;28:S326.

168. Ohashi N, Gaowa, Wuritu, Kawamori F, Wu D, Yoshikawa Y, et al. Human granulocytic Anaplasmosis, Japan. Emerg Infect Dis. 2013;19(2):289-92. Epub 2013/03/07. doi: 10.3201/eid1902.120855. PubMed PMID: 23460988; PubMed Central PMCID: PMCPMC3559047.

169. Vanicek J, Stastnik M, Kianicka B, Bares M, Bulik M. Rare neurological presentation of human granulocytic anaplasmosis. Eur J Neurol. 2013;20(5):e70-2. Epub 2013/04/13. doi: 10.1111/ene.12110. PubMed PMID: 23577610.

170. Wormser GP, Aguero-Rosenfeld ME, Cox ME, Nowakowski J, Nadelman RB, Holmgren D, et al. Differences and similarities between culture-confirmed human granulocytic anaplasmosis and early lyme disease. J Clin Microbiol. 2013;51(3):954-8. Epub 2013/01/11. doi: 10.1128/jcm.02929-12. PubMed PMID: 23303504; PubMed Central PMCID: PMCPMC3592036.

171. Zhang L, Wang G, Liu Q, Chen C, Li J, Long B, et al. Molecular analysis of Anaplasma phagocytophilum isolated from patients with febrile diseases of unknown etiology in China. PLoS One. 2013;8(2):e57155. Epub 2013/03/02. doi: 10.1371/journal.pone.0057155. PubMed PMID: 23451170; PubMed Central PMCID: PMCPMC3579781.

172. Aggarwal S, Bannon S. Anaplasmosis. J Glob Infect Dis. 2014;6(1):47-8. Epub 2014/04/18. doi: 10.4103/0974-777x.127958. PubMed PMID: 24741236; PubMed Central PMCID: PMCPMC3982361.

173. Arraga-Alvarado CM, Qurollo BA, Parra OC, Berrueta MA, Hegarty BC, Breitschwerdt EB. Case report: Molecular evidence of Anaplasma platys infection in two women from Venezuela. Am J Trop Med Hyg. 2014;91(6):1161-5. Epub 2014/10/01. doi: 10.4269/ajtmh.14-0372. PubMed PMID: 25266347; PubMed Central PMCID: PMCPMC4257640.

174. Gaowa, Yoshikawa Y, Ohashi N, Wu D, Kawamori F, Ikegaya A, et al. Anaplasma phagocytophilum antibodies in humans, Japan, 2010-2011. Emerging Infectious Diseases. 2014;20(3):508-9. doi: 10.3201/eid2003.131337.

175. Hagedorn P, Imhoff M, Fischer C, Domingo C, Niedrig M. Human granulocytic anaplasmosis acquired in Scotland, 2013. Emerg Infect Dis. 2014;20(6):1079-81. Epub 2014/05/27. doi: 10.3201/eid2006.131849. PubMed PMID: 24857681; PubMed Central PMCID: PMCPMC4036789.

176. Hing M, Woestyn S, Van Bosterhaut B, Desbonnet Y, Heyman P, Cochez C, et al. Diagnosis of human granulocytic anaplasmosis in Belgium by combining molecular and serological methods. New Microbes New Infect. 2014;2(6):177-8. Epub 2015/01/08. doi: 10.1002/nmi2.65. PubMed PMID: 25566398; PubMed Central PMCID: PMCPMC4265051.

177. Hosseini-Vasoukolaei N, Ali Oshaghi M, Shayan P, Vatandoost H, Babamahmoudi F, Yaghoobi-Ershadi MR, et al. Anaplasma infection in ticks, livestock and human in Ghaemshahr, Mazandaran Province, Iran. Journal of Arthropod-Borne Diseases. 2014;8(2):204-11.

178. Karlen N, Peters MN, Roussel J. Give me doxycycline or give me death. Journal of General Internal Medicine. 2014;29:S359.

179. Kim K-H, Yi J, Oh WS, Kim N-H, Choi SJ, Choe PG, et al. Human granulocytic anaplasmosis, South Korea, 2013. Emerging Infectious Diseases. 2014;20(10):1708-11. doi: 10.3201/eid2010.131680. PubMed PMID: 109757736. Language: English. Entry Date: 20150814. Revision Date: 20220203. Publication Type: journal article.

180. Mayne PJ. Clinical determinants of Lyme borreliosis, babesiosis, bartonellosis, anaplasmosis, and ehrlichiosis in an Australian cohort. International Journal of General Medicine. 2014;8:15-26. doi: 10.2147/IJGM.S75825.

181. Moniuszko A, Dunaj J, Swięcicka I, Zambrowski G, Chmielewska-Badora J, Zukiewicz-Sobczak W, et al. Co-infections with Borrelia species, Anaplasma phagocytophilum and Babesia spp. in patients with tick-borne encephalitis. Eur J Clin Microbiol Infect Dis. 2014;33(10):1835-41. Epub 2014/05/23. doi: 10.1007/s10096-014-2134-7. PubMed PMID: 24848130; PubMed Central PMCID: PMCPMC4182641.

182. Mutneja R, Shah M, Shivamurthy P, Gowani S, Delgado-Colon D, Lawlor M. Acute respiratory distress syndrome in human granulocytic ehrlichiosis. Conn Med. 2014;78(6):345-7. Epub 2015/02/13. PubMed PMID: 25672061.

183. Picco P, Naselli A, Pala G, Rizzo F, Damasio B, Buoncompagni A, et al. Whole-body MRI as an unconventional diagnostic tool in a pediatric patient with systemic infection. Acta Radiol Short Rep. 2014;3(11):2047981614549571. Epub 2014/12/24. doi: 10.1177/2047981614549571. PubMed PMID: 25535572; PubMed Central PMCID: PMCPMC4271715.

184. Rand JV, Tarasen AJ, Kumar J, Homan SM, Tobin E. Intracytoplasmic granulocytic morulae counts on confirmed cases of ehrlichiosis/anaplasmosis in the Northeast. Am J Clin Pathol. 2014;141(5):683-6. Epub 2014/04/10. doi: 10.1309/ajcp6q2bokyaldyz. PubMed PMID: 24713739.

185. Selvaraj V, Leyse J, Magauran C. Deceptively simple or simply deceptive! Journal of the American Geriatrics Society. 2014;62:S289. doi: 10.1111/jgs.12870.

186. Townsend RL, Moritz ED, Fialkow LB, Berardi V, Stramer SL. Probable transfusion-transmission of Anaplasma phagocytophilum by leukoreduced platelets. Transfusion. 2014;54(11):2828-32. Epub 2014/04/18. doi: 10.1111/trf.12675. PubMed PMID: 24738852.

187. Arcila VG, Arroyo Salgado BJ, Bello Espinosa AA, Escobar ZR, Polo Andrade ER. Microbiological diagnosis compatible with anaplasma sp. in a patient suffering from febrile syndrome. Revista Argentina de Microbiologia. 2015;47(1):78-9. doi: 10.1016/j.ram.2015.01.001.

188. Broussard JR, Johansen K. Autoimmune primary adrenal insufficiency associated with aseptic meningitis from tick-borne infection. Endocrine Reviews. 2015;36.

189. Christmann D. [Lyme borreliosis and co-infections. Place of Anaplasma phagocytophilum and Bartonella henselae]. Bull Acad Natl Med. 2015;199(4-5):617-26; discussion 26-8. Epub 2015/04/01. PubMed PMID: 27509682.

190. Cooper JD, Dometita D, Hasan A, Dorion P, Wolk DM, Martinez RM. "Orange" You Glad You Checked the Buffy Coat? Clinical Microbiology Newsletter. 2015;37(2):9-13. doi: 10.1016/j.clinmicnews.2015.01.001.

191. Dahlgren FS, Heitman KN, Drexler NA, Massung RF, Behravesh CB. Human granulocytic anaplasmosis in the United States from 2008 to 2012: a summary of national surveillance data. Am J Trop Med Hyg. 2015;93(1):66-72. Epub 2015/04/15. doi: 10.4269/ajtmh.15-0122. PubMed PMID: 25870428; PubMed Central PMCID: PMCPMC4497906.

192. DeRose J. Human granulocytic anaplasmosis in the setting of a hunting injury. International Journal of Antimicrobial Agents. 2015;45:S106-S7.

193. García JC, Núñez MJ, Portillo A, Oteo JA. [Human anaplasmosis: two case-reports]. Enferm Infecc Microbiol Clin. 2015;33(1):68-9. Epub 2014/07/31. doi: 10.1016/j.eimc.2014.05.009. PubMed PMID: 25073813.

194. Jiao X-y, Fan Z-c, Li Y-z, Tang Y-t, Ke C-w. Clinical and laboratory features parameters of human granulocytic anaplasmosis (HGA) in patients admitted to hospital in Guangdong Province, China. Tropical Doctor. 2015;45(4):209-13. doi: 10.1177/0049475515579772. PubMed PMID: 110506611. Language: English. Entry Date: 20160615. Revision Date: 20180622. Publication Type: journal article.

195. Kaphle U, Kheir F, Thammasitboon S. A Rare Case of ARDS From Human Anaplasmosis. Respir Care. 2015;60(7):e125-7. Epub 2015/02/12. doi: 10.4187/respcare.03714. PubMed PMID: 25669216.

196. Li H, Zheng Y-C, Ma L, Jia N, Jiang B-G, Jiang R-R, et al. Human infection with a novel tick-borne Anaplasma species in China: a surveillance study. Lancet Infectious Diseases. 2015;15(6):663-70. doi: 10.1016/S1473-3099(15)70051-4. PubMed PMID: 109788568. Language: English. Entry Date: 20150923. Revision Date: 20200708. Publication Type: journal article.

197. Lotrič-Furlan S, Rojko T, Jelovšek M, Petrovec M, Avšič-Županc T, Lusa L, et al. Comparison of clinical and laboratory characteristics of patients fulfilling criteria for proven and probable human granulocytic anaplasmosis. Microbes Infect. 2015;17(11-12):829-33. Epub 2015/10/04. doi: 10.1016/j.micinf.2015.09.017. PubMed PMID: 26432519.

198. Marijo Aguileraa c, Anne Marie Furusethb, Lauren Giacobbea, Katherine Jacobsa, Kirk Ramina. A Case of Severe Human Granulocytic Anaplasmosis in an Immunocompromised Pregnant Patient. Journal of Medical cases. 2015;6:282-4.

199. Shields K, Cumming M, Rios J, Wong MT, Zwicker JI, Stramer SL, et al. Transfusion-associated Anaplasma phagocytophilum infection in a pregnant patient with thalassemia trait: a case report. Transfusion. 2015;55(4):719-25. Epub 2014/11/12. doi: 10.1111/trf.12908. PubMed PMID: 25385549.

200. Solar VR, Mendoza De La Garza M, Treadwell T. Heart failure and atrial fibrillation triggered by anaplasmosis in an elderly female. Journal of the American Geriatrics Society. 2015;63:S244. doi: 10.1111/jgs.13439.

201. Tan EM, Marcelin JR, Tande AJ, Rizza SA, Cummins NW. Fever and Cardiac Arrest in a Patient With a Left Ventricular Assist Device. Open Forum Infect Dis. 2015;2(2):ofv033. Epub 2015/09/18. doi: 10.1093/ofid/ofv033. PubMed PMID: 26380334; PubMed Central PMCID: PMCPMC4567092.

202. Welc-Falęciak R, Kowalec M, Zajkowska J, Pancewicz SA, Siński E. Clinical and molecular features of one case of human infection with Anaplasma phagocytophilum from Podlaskie Province in eastern Poland. Ann Agric Environ Med. 2015;22(3):414-7. Epub 2015/09/26. doi: 10.5604/12321966.1167704. PubMed PMID: 26403105.

203. Alyemni D, Uppal G, Nwaoduah N, Ly V. Blood film findings in anaplasmosis. Br J Haematol. 2016;175(3):365. Epub 2016/10/28. doi: 10.1111/bjh.14281. PubMed PMID: 27489126.

204. Fernandes A. A case of acute transient flaccid paralysis with anaplasmosis. Journal of General Internal Medicine. 2016;31(2):S487.

205. Fine AB, Sweeney JD, Nixon CP, Knoll BM. Transfusion-transmitted anaplasmosis from a leukoreduced platelet pool. Transfusion. 2016;56(3):699-704. Epub 2015/12/10. doi: 10.1111/trf.13392. PubMed PMID: 26645855.

206. Galloo X, Wiels W, Du Four S, Surmont M, Mertens R. Beyond lyme: Tick-borne illness in Europe. Acta Clinica Belgica: International Journal of Clinical and Laboratory Medicine. 2016;71:40. doi: 10.1080/17843286.2016.1250435.

207. Jahfari S, Hofhuis A, Fonville M, van der Giessen J, van Pelt W, Sprong H. Molecular Detection of Tick-Borne Pathogens in Humans with Tick Bites and Erythema Migrans, in the Netherlands. PLoS Negl Trop Dis. 2016;10(10):e0005042. Epub 2016/10/06. doi: 10.1371/journal.pntd.0005042. PubMed PMID: 27706159; PubMed Central PMCID: PMCPMC5051699.

208. Markowicz M, Schötta AM, Wijnveld M, Stanek G. Human granulocytic anaplasmosis acquired in Connecticut, USA, diagnosed in Vienna, Austria, 2015. Diagn Microbiol Infect Dis. 2016;84(4):347-9. Epub 2016/01/19. doi: 10.1016/j.diagmicrobio.2015.12.004. PubMed PMID: 26778486.

209. Mujahid R, Colon-cartagena W. A heart-breaking tick bite. Case of a patient with Human Granulocytic Anaplasmosis Cardiomyopathy. Journal of the American Geriatrics Society. 2016;64:S93-S. PubMed PMID: WOS:000374763800259.

210. Pañczuk A, Tokarska-Rodak M, Kozioł-Montewka M, Plewik D. The incidence of Borrelia burgdorferi, Anaplasma phagocytophilum and Babesia microti coinfections among foresters and farmers in eastern Poland. J Vector Borne Dis. 2016;53(4):348-54. Epub 2016/12/31. PubMed PMID: 28035112.

211. Pokorn M, Županc TA, Strle F. Pediatric Human Granulocytic Anaplasmosis is Rare in Europe. Pediatr Infect Dis J. 2016;35(3):358-9. Epub 2016/02/13. doi: 10.1097/inf.0000000000001004. PubMed PMID: 26866855.

212. Tintel Astigarraga MJ, Paola AS, Nara EM. Ehrlichiosis, tick-borne disease: A potential zoonosis in Paraguay. Revista Electronica de Veterinaria. 2016;17(9).

213. Alarcon SV, Murati J, Ciofoaia GA, Sundar PR. Renal involvement in anaplasmosis. Journal of General Internal Medicine. 2017;32(2):S582.

214. Caranfa JT, Kohn CG, Coleman CI. Case studies (August 2017). 2017.

215. Fleischner Z, Koo T, Abdul MH, Marcos LA. Clinical features of PCR confirmed human ehrlichiosis and anaplasmosis in an endemic area of the Northeast United States. Open Forum Infectious Diseases. 2017;4:S121-S2. doi: 10.1093/ofid/ofx163.156.

216. Johnson TM, Brown MS, Rabbat M, Slim J. Hemophagocytic Lymphohistiocytosis Associated with Anaplasmosis. J Glob Infect Dis. 2017;9(2):76-8. Epub 2017/06/07. doi: 10.4103/jgid.jgid_116_16. PubMed PMID: 28584460; PubMed Central PMCID: PMCPMC5452556.

217. Kim CM, Kim SW, Kim DM, Yoon NR, Jha P, Jang SJ, et al. Case Report: Polymerase Chain Reaction Testing of Tick Bite Site Samples for the Diagnosis of Human Granulocytic Anaplasmosis. American Journal of Tropical Medicine and Hygiene. 2017;97(2):403-6. doi: 10.4269/ajtmh.16-0570. PubMed PMID: WOS:000423197200016.

218. Lagler H, Harrison N, Kussmann M, Obermüller M, Burgmann H, Makristathis A, et al. Direct detection of Anaplasma phagocytophilum by polymerase chain reaction followed by electrospray ionization mass spectrometry from human blood. Int J Infect Dis. 2017;60:61-3. Epub 2017/05/21. doi: 10.1016/j.ijid.2017.05.006. PubMed PMID: 28526564.

219. Lee SH, Park SY, Jang MJ, Choi KJ, Lee HK, Cho YU, et al. Clinical Isolation of Anaplasma phagocytophilum in South Korea. Am J Trop Med Hyg. 2017;97(6):1686-90. Epub 2017/11/17. doi: 10.4269/ajtmh.16-0529. PubMed PMID: 29141715; PubMed Central PMCID: PMCPMC5805025.

220. Marko D, Perry AM, Ponnampalam A, Nasr MR. Cytopenias and clonal expansion of gamma/delta T-cells in a patient with anaplasmosis: a potential diagnostic pitfall. J Clin Exp Hematop. 2017;56(3):160-4. Epub 2017/03/24. doi: 10.3960/jslrt.56.160. PubMed PMID: 28331130; PubMed Central PMCID: PMCPMC6144175.

221. Moniuszko-Malinowska A, Czupryna P, Dunaj J, Swierzbinska R, Guziejko K, Rutkowski R, et al. Evaluation of NF-kappa B concentration in patients with tick-borne encephalitis, neuroborreliosis, anaplasmosis and Anaplasma phagocythophilum with tick-borne encephalitis virus co-infection. Cytokine. 2017;90:155-60. doi: 10.1016/j.cyto.2016.10.014. PubMed PMID: WOS:000394396100022.

222. Nitzan O, Blum A, Marva E, Katz A, Tzadok BS, Nachum-Biala Y, et al. Case Report: Infectious Diseases in Pilgrims Visiting the Holy Land. Am J Trop Med Hyg. 2017;97(2):611-4. Epub 2017/07/20. doi: 10.4269/ajtmh.17-0097. PubMed PMID: 28722620; PubMed Central PMCID: PMCPMC5544103.

223. Schotthoefer AM, Schrodi SJ, Meece JK, Fritsche TR, Shukla SK. Pro-inflammatory immune responses are associated with clinical signs and symptoms of human anaplasmosis. PLoS One. 2017;12(6):e0179655. Epub 2017/06/20. doi: 10.1371/journal.pone.0179655. PubMed PMID: 28628633; PubMed Central PMCID: PMCPMC5476275.

224. Sigurjonsdottir VK, Feder HM, Jr., Wormser GP. Anaplasmosis in pediatric patients: Case report and review. Diagn Microbiol Infect Dis. 2017;89(3):230-4. Epub 2017/10/21. doi: 10.1016/j.diagmicrobio.2017.08.003. PubMed PMID: 29050793.

225. Tsiodras S, Spanakis N, Spanakos G, Pervanidou D, Georgakopoulou T, Campos E, et al. Fatal human anaplasmosis associated with macrophage activation syndrome in Greece and the Public Health response. J Infect Public Health. 2017;10(6):819-23. Epub 2017/02/13. doi: 10.1016/j.jiph.2017.01.002. PubMed PMID: 28189511.

226. Vikse J, Klos J, Berg A. A travelling camper with a spiking fever, headache, myalgia, hepatitis, and intracellular inclusions. Lancet Infect Dis. 2017;17(12):1318. Epub 2017/11/28. doi: 10.1016/s1473-3099(17)30305-5. PubMed PMID: 29173891.

227. Yi J, Kim KH, Ko MK, Lee EY, Choi SJ, Oh MD. Human Granulocytic Anaplasmosis as a Cause of Febrile Illness in Korea Since at Least 2006. Am J Trop Med Hyg. 2017;96(4):777-82. Epub 2017/01/18. doi: 10.4269/ajtmh.16-0309. PubMed PMID: 28093540; PubMed Central PMCID: PMCPMC5392619.

228. Ahn J, Swanenberg I, Schmidt N, Janjigian M. Ticked off: A case of anaplasmosis and lyme co-infection. Journal of General Internal Medicine. 2018;33(2):645-6.

229. Camacci ML, Panganiban RP, Pattison Z, Haghayeghi K, Daly A, Ojevwe C, et al. Severe Human Granulocytic Anaplasmosis With Significantly Elevated Ferritin Levels in an Immunocompetent Host in Pennsylvania: A Case Report. J Investig Med High Impact Case Rep. 2018;6:2324709618758350. Epub 2018/02/23. doi: 10.1177/2324709618758350. PubMed PMID: 29468169; PubMed Central PMCID: PMCPMC5815407.

230. Chase B, Bonnar P. A walk through the tall grass: A case of transaminitis, thrombocytopenia, and leukopenia resulting from an emerging zoonotic infection in nova scotia. JAMMI. 2018;3(4):247-50. doi: 10.3138/jammi.2018-0023.

231. Duca N, Chapman T, Yenebere P. Ticks and tricks: Empiric anaplasmosis meningitis coverage in endemic areas. Journal of General Internal Medicine. 2018;33(2):646.

232. Dunaj J, Moniuszko-Malinowska A, Swiecicka I, Andersson M, Czupryna P, Rutkowski K, et al. Tick-borne infections and co-infections in patients with non-specific symptoms in Poland. Adv Med Sci. 2018;63(1):167-72. Epub 2017/11/10. doi: 10.1016/j.advms.2017.09.004. PubMed PMID: 29120859.

233. Edginton S, Guan TH, Evans G, Srivastava S. Human granulocytic anaplasmosis acquired from a blacklegged tick in Ontario. Cmaj. 2018;190(12):E363-e6. Epub 2018/03/28. doi: 10.1503/cmaj.171243. PubMed PMID: 29581163; PubMed Central PMCID: PMCPMC5871440.

234. Epstein R, Ristau J, Ellner JJ. An Unsuspected Zoonotic Infection Presenting as Sepsis. Am J Med. 2018;131(1):e17-e8. Epub 2017/09/02. doi: 10.1016/j.amjmed.2017.07.040. PubMed PMID: 28860034.

235. Gaowa, Wulantuya, Yin X, Cao M, Guo S, Ding C, et al. Case of Human Infection with Anaplasma phagocytophilum in Inner Mongolia, China. Jpn J Infect Dis. 2018;71(2):155-7. Epub 2018/03/02. doi: 10.7883/yoken.JJID.2017.450. PubMed PMID: 29491236.

236. Goel R, Westblade LF, Kessler DA, Sfeir M, Slavinski S, Backenson B, et al. Death from Transfusion-Transmitted Anaplasmosis, New York, USA, 2017. Emerg Infect Dis. 2018;24(8):1548-50. Epub 2018/07/18. doi: 10.3201/eid2408.172048. PubMed PMID: 30016241; PubMed Central PMCID: PMCPMC6056119.

237. Hall LT, Beyea A. Bitten and bedbound: Human granulocytic anaplasmosis in a patient with frontotemporal dementia. Journal of the American Geriatrics Society. 2018;66:S101. doi: 10.1111/jgs.15376.

238. Hirsh BA, Greenspon LW. Anaplasmosis-associated pneumonitis. American Journal of Respiratory and Critical Care Medicine. 2018;197(MeetingAbstracts).

239. Khan NR, Hasan A, Bijol V, Jhaveri KD, Uppal NN. Anaplasmosis induced acute interstitial nephritis (AIN) following tick bite: A rare association. Journal of the American Society of Nephrology. 2018;29:263.

240. Khan R, Ali A. Non-traumatic splenic rupture in a patient with human granulocytic anaplasmosis and focused review of the literature. Ticks Tick Borne Dis. 2018;9(3):735-7. Epub 2018/03/04. doi: 10.1016/j.ttbdis.2018.02.017. PubMed PMID: 29500125.

241. Kim SW, Kim C-M, Kim D-M, Yun NR. Manifestation of anaplasmosis as cerebral infarction: a case report. BMC Infectious Diseases. 2018;18(1):N.PAG-N.PAG. doi: 10.1186/s12879-018-3321-4. PubMed PMID: 131287139. Language: English. Entry Date: In Process. Revision Date: 20180908. Publication Type: journal article.

242. Kobayashi KJ, Weil AA, Branda JA. Case 16-2018: A 45-Year-Old Man with Fever, Thrombocytopenia, and Elevated Aminotransferase Levels. N Engl J Med. 2018;378(21):2023-9. Epub 2018/05/24. doi: 10.1056/NEJMcpc1712227. PubMed PMID: 29791814.

243. Lavoignet CE, Le Borgne P, Slimani H. Anaplasmosis: A diagnosis to evoke after a tick bite. Annales Francaises de Medecine d'Urgence. 2018;8(1):43-5. doi: 10.1007/s13341-017-0797-2.

244. Lee SH, Park S, Lee YS, Lee HK, Hwang SD. Diagnosis and molecular characteristics of human infections caused by Anaplasma phagocytophilum in South Korea. J Microbiol. 2018;56(11):847-53. Epub 2018/10/26. doi: 10.1007/s12275-018-8385-8. PubMed PMID: 30353471.

245. Mascarenhas TR, Silibovsky RS, Singh P, Belden KA. Tick-borne illness after transplantation: Case and review. Transplant Infectious Disease. 2018;20(2). doi: 10.1111/tid.12830.

246. Mei C, Shah R, Fisher MJ, Marcos LA, Mansour M. A peculiar case of sepsis: The role of procalcitonin in diagnosing human granulocytic anaplasmosis. American Journal of Respiratory and Critical Care Medicine. 2018;197(MeetingAbstracts).

247. Ray AS, Cain HC. A patient with anaplasmosis and subsequent spontaneous splenic rupture. American Journal of Respiratory and Critical Care Medicine. 2018;197(MeetingAbstracts).

248. Samanani S, Kandalaft O, Joshi K, Colon-Cartagena W. A stroke of good luck. Journal of the American Geriatrics Society. 2018;66:S267-S8. doi: 10.1111/jgs.15376.

249. Samson M, Wilcox SR, Liu SW. Rash and Thrombocytopenia. J Emerg Med. 2018;55(5):710-3. Epub 2018/09/29. doi: 10.1016/j.jemermed.2018.07.027. PubMed PMID: 30262250.

250. Schotthoefer AM, Hall MC, Vittala S, Bajwa R, Frost HM. Clinical Presentation and Outcomes of Children With Human Granulocytic Anaplasmosis. J Pediatric Infect Dis Soc. 2018;7(2):e9-e15. Epub 2017/05/19. doi: 10.1093/jpids/pix029. PubMed PMID: 28520981.

251. Sharma A, Orr L, Shore ET. Ards due to anaplasma and borrelia co-infection; can corticosteroids help? American Journal of Respiratory and Critical Care Medicine. 2018;197(MeetingAbstracts).

252. Sherman Z, McWilliams G. An unexpected cause of neutropenic fever. Journal of General Internal Medicine. 2018;33(2):462-3.

253. Tuan J, Weinstock C. Congestive heart failure in anaplasmosis and babe-siosis. Journal of General Internal Medicine. 2018;33(2):497.

254. Uminski K, Kadkhoda K, Houston BL, Lopez A, MacKenzie LJ, Lindsay R, et al. Anaplasmosis: An emerging tick-borne disease of importance in Canada. IDCases. 2018;14:e00472. Epub 2018/12/14. doi: 10.1016/j.idcr.2018.e00472. PubMed PMID: 30524954; PubMed Central PMCID: PMCPMC6278667.

255. Varshney A, Barkoudah E. The Simplest Explanation: Pancytopenia. Am J Med. 2018;131(9):1052-4. Epub 2018/04/13. doi: 10.1016/j.amjmed.2018.03.017. PubMed PMID: 29649461.

256. Welc-Falęciak R, Bednarska M, Szatan M, Kowalska JD, Pawełczyk A. Molecular identification of tick-borne pathogens in asymptomatic individuals with human immunodeficiency virus type 1 (HIV-1) infection: a retrospective study. BMC Infectious Diseases. 2018;18(1):N.PAG-N.PAG. doi: 10.1186/s12879-018-3140-7. PubMed PMID: 129675553. Language: English. Entry Date: In Process. Revision Date: 20190308. Publication Type: journal article.

257. Albitar HAH, Gurram PR, Esquer Garrigos Z, Sohail MR. Oh Deer! A Case of Fever and Myalgia in a Liver Transplant Recipient. Am J Med. 2019;132(9):e707-e8. Epub 2019/05/06. doi: 10.1016/j.amjmed.2019.04.011. PubMed PMID: 31051148.

258. Azab N, Smith K, Spitzer E, Weinbaum FI, Marcos L. Comparison of clinical and laboratory findings of human monocytic ehrlichiosis (HME) and human granulocytic anaplasmosis (HGA) in Long Island, New York. Open Forum Infectious Diseases. 2019;6:S126. doi: 10.1093/ofid/ofz360.289.

259. Buttar B, Szema AM. Anaplasmosis, babesia, and aphasia in a new home owner with a wooded lot. American Journal of Respiratory and Critical Care Medicine. 2019;199(9).

260. Chen R, Kou Z, Xu L, Cao J, Liu Z, Wen X, et al. Analysis of epidemiological characteristics of four natural-focal diseases in Shandong Province, China in 2009-2017: A descriptive analysis. PLoS One. 2019;14(8):e0221677. Epub 2019/08/28. doi: 10.1371/journal.pone.0221677. PubMed PMID: 31454372; PubMed Central PMCID: PMCPMC6711524.

261. Choi S, Cho YU, Kim SH. Morulae in neutrophils: A diagnostic clue for human granulocytic anaplasmosis. IDCases. 2019;15:e00506. Epub 2019/03/09. doi: 10.1016/j.idcr.2019.e00506. PubMed PMID: 30847278; PubMed Central PMCID: PMCPMC6389682.

262. Chong G, Hanna W, Scuorzo J, Ali T. ACUTE KIDNEY INJURY AND HEMOPHAGOCYTIC LYMPHOHISTIOCYTOSIS (HLH) CAUSED BY HUMAN GRANULOCYTIC ANAPLASMOSIS (HGA). American Journal of Kidney Diseases. 2019;73(5):664. doi: 10.1053/j.ajkd.2019.03.087.

263. Cooperman J. A curious cause of abdominal pain in a patient with anaplasmosis. Journal of General Internal Medicine. 2019;34(2):S438. doi: 10.1007/11606.1525-1497.

264. Dylewski J, Domingo MC, Thivierge K. A hail Mary diagnostic success: Human granulocytic anaplasmosis. Infectious Diseases in Clinical Practice. 2019;27(4):226-7. doi: 10.1097/IPC.0000000000000740.

265. El Khoury L, Furie R. Inflammatory arthritis: a unique presentation of human anaplasmosis. Clin Rheumatol. 2019;38(1):257-9. Epub 2018/12/13. doi: 10.1007/s10067-018-4395-5. PubMed PMID: 30539351.

266. Escárcega-Ávila AM, de la Mora-Covarrubias A, Quezada-Casasola A, Jiménez-Vega F. Occupational risk for personnel working in veterinary clinics through exposure to vectors of rickettsial pathogens. Ticks Tick Borne Dis. 2019;10(2):299-304. Epub 2018/11/25. doi: 10.1016/j.ttbdis.2018.10.012. PubMed PMID: 30470470.

267. Hansmann Y, Jaulhac B, Kieffer P, Martinot M, Wurtz E, Dukic R, et al. Value of PCR, Serology, and Blood Smears for Human Granulocytic Anaplasmosis Diagnosis, France. Emerg Infect Dis. 2019;25(5):996-8. Epub 2019/04/20. doi: 10.3201/eid2505.171751. PubMed PMID: 31002073; PubMed Central PMCID: PMCPMC6478229.

268. Heo DH, Hwang JH, Choi SH, Jeon M, Lee JH, Lee JH, et al. Recent Increase of Human Granulocytic Anaplasmosis and Co-Infection with Scrub Typhus or Korean Hemorrhagic Fever with Renal Syndrome in Korea. J Korean Med Sci. 2019;34(11):e87. Epub 2019/03/28. doi: 10.3346/jkms.2019.34.e87. PubMed PMID: 30914905; PubMed Central PMCID: PMCPMC6427050.

269. Herbst J, Crissinger T, Baldwin K. Diffuse Ischemic Strokes and Sickle Cell Crisis Induced by Disseminated Anaplasmosis: A Case Report. Case Rep Neurol. 2019;11(3):271-6. Epub 2019/10/15. doi: 10.1159/000502567. PubMed PMID: 31607893; PubMed Central PMCID: PMCPMC6787427.

270. Hing M, Van Den Bossche D, Lernout T, Cochez C, Pirnay JP, Heuninckx W. Prevalence of Anaplasma phagocytophilum in humans in Belgium for the period 2013-2016. Acta Clin Belg. 2019;74(4):280-5. Epub 2018/07/22. doi: 10.1080/17843286.2018.1491928. PubMed PMID: 30029581.

271. Khatri A, Lloji A, Doobay R, Wang G, Knoll B, Dhand A, et al. Anaplasma phagocytophilum presenting with orchitis in a renal transplant recipient. Transpl Infect Dis. 2019;21(4):e13129. Epub 2019/06/20. doi: 10.1111/tid.13129. PubMed PMID: 31215144.

272. Kim B, Idahosa O, Jahre JA. Fulminant Human Granulocytic Anaplasmosis after CABG: An Unusual Occurrence. Am J Med. 2019;132(10):e744-e5. Epub 2019/05/28. doi: 10.1016/j.amjmed.2019.05.006. PubMed PMID: 31132329.

273. Kim JH, Lee CS, Moon C, Kwak YG, Kim BN, Kim ES, et al. Co-infection of scrub typhus and human granulocytic anaplasmosis in Korea, 2006. Journal of Korean Medical Science. 2019;34(39). doi: 10.3346/jkms.2019.34.e257.

274. Koperwas M, Manov N, Yuan D. Anaplasmosis in a patient with non-specific febrile illness and pulmonary emboli. Journal of the American Geriatrics Society. 2019;67:S182. doi: 10.1111/jgs.15898.

275. Kumar M, Sharma A, Grover P. Triple Tick Attack. Cureus. 2019;11(2):e4064. Epub 2019/04/25. doi: 10.7759/cureus.4064. PubMed PMID: 31016091; PubMed Central PMCID: PMCPMC6464285.

276. Liu HB, Ran W, Ni XB, Zheng YC, Huo QB, Jiang BG, et al. The prevalence and clinical characteristics of tick-borne diseases at One Sentinel Hospital in Northeastern China. Parasitology. 2019;146(2):161-7. doi: 10.1017/s0031182018001178. PubMed PMID: WOS:000458677800003.

277. Lu M, Li F, Liao Y, Shen JJ, Xu JM, Chen YZ, et al. Epidemiology and Diversity of Rickettsiales Bacteria in Humans and Animals in Jiangsu and Jiangxi provinces, China. Sci Rep. 2019;9(1):13176. Epub 2019/09/13. doi: 10.1038/s41598-019-49059-3. PubMed PMID: 31511528; PubMed Central PMCID: PMCPMC6739303.

278. Moullet V, Kantamneni P, Carrera P. Septic shock or hemophagocytic lymphohistiocytosis: The importance of a timely diagnosis. Critical Care Medicine. 2019;47(1).

279. Tsai KH, Chung LH, Chien CH, Tung YJ, Wei HY, Yen TY, et al. Human granulocytic anaplasmosis in Kinmen, an offshore island of Taiwan. PLoS Negl Trop Dis. 2019;13(9):e0007728. Epub 2019/09/21. doi: 10.1371/journal.pntd.0007728. PubMed PMID: 31539395; PubMed Central PMCID: PMCPMC6774531.

280. Walkty A, Karlowsky J, Zarychanski R, Kadkhoda K, Lagacé-Wiens P. Marked elevation of serum ferritin associated with Anaplasma phagocytophilum infection. J Assoc Med Microbiol Infect Dis Can. 2019;4(1):37-41. Epub 2019/03/11. doi: 10.3138/jammi.2018-0033. PubMed PMID: 36338785; PubMed Central PMCID: PMCPMC9603192.

281. Ward A, Abdullah A, Young K, Sugunaraj JP. Diffuse alveolar hemorrhage (DAH)-a rare life-threatening manifestation of anaplasmosis. American Journal of Respiratory and Critical Care Medicine. 2019;199(9).

282. Zhang Z, Best P. Don't overlook that tick bite. Critical Care Medicine. 2019;47(1).

283. Zhuo M, Caev H, Saunders SJ, Li JH, Stillman IE, Danziger J. Acute Kidney Injury Associated With Human Granulocytic Anaplasmosis: A Case Report. American Journal of Kidney Diseases. 2019;74(5):696-9. doi: 10.1053/j.ajkd.2019.03.428. PubMed PMID: WOS:000491894500018.

284. Rare case of severe rhabdomyolysis secondary to human granulocytic anaplasmosis. Elsevier B.V.; 2020. p. 1543.e1-.e2.

285. Baker A, Wang HH, Mogg M, Derouen Z, Borski J, Grant WE. Increasing Incidence of Anaplasmosis in the United States, 2012 Through 2016. Vector Borne Zoonotic Dis. 2020;20(11):855-9. Epub 2020/07/01. doi: 10.1089/vbz.2019.2598. PubMed PMID: 32598241.

286. Becerra V, Christov M, Griffiths J, Bustos A. HUMAN GRANULOCYTIC ANAPLASMOSIS CAUSING ACUTE KIDNEY FAILURE. American Journal of Kidney Diseases. 2020;75(4):550. doi: 10.1053/j.ajkd.2020.02.052.

287. Fisher M, Patel P, Slenker A, Langstengel J, Smith S. A FATAL CASE OF CARDIOMYOPATHY SECONDARY TO HUMAN GRANULOCYTIC ANAPLASMOSIS IN AN IMMUNOCOMPROMISED PATIENT. Chest. 2020;158(4):A235. doi: 10.1016/j.chest.2020.08.240.

288. Guziejko K, Czupryna P, Pancewicz S, Świerzbińska R, Dunaj J, Kruszewska E, et al. Analysis of CCL-4, CCL-17, CCL-20 and IL-8 concentrations in the serum of patients with tick-borne encephalitis and anaplasmosis. Cytokine. 2020;125:154852. Epub 2019/09/29. doi: 10.1016/j.cyto.2019.154852. PubMed PMID: 31561102.

289. Hoepler W, Markowicz M, Schoetta A-M, Zoufaly A, Stanek G, Wenisch C. Molecular diagnosis of autochthonous human anaplasmosis in Austria - an infectious diseases case report. BMC Infectious Diseases. 2020;20(1):1-5. doi: 10.1186/s12879-020-04993-w. PubMed PMID: 142792169. Language: English. Entry Date: In Process. Revision Date: 20221004. Publication Type: journal article.

290. Keltner C. A novel case of life-threatening thrombocytopenia secondary to anaplasmosis. Infectious Diseases in Clinical Practice. 2020;28(6):e52-e4. doi: 10.1097/IPC.0000000000000888.

291. Lee SH, Shin N-R, Kim C-M, Park S, Yun NR, Kim D-M, et al. First identification of Anaplasma phagocytophilum in both a biting tick Ixodes nipponensis and a patient in Korea: a case report. BMC Infectious Diseases. 2020;20(1):N.PAG-N.PAG. doi: 10.1186/s12879-020-05522-5. PubMed PMID: 146949937. Language: English. Entry Date: 20201120. Revision Date: 20210916. Publication Type: journal article.

292. Matkovic E, Rohrer CT. Fido in the mulberry grove. Blood. 2020;136(20):2360. Epub 2020/11/13. doi: 10.1182/blood.2020008691. PubMed PMID: 33180923.

293. Misra S, Frampton J, Friedman S. THE TICK THAT INFECTED THE TICKER: A CASE OF ANAPLASMA MYOPERICARDITIS IN A VERMONT VETERAN. Journal of the American College of Cardiology. 2020;75(11):2530. doi: 10.1016/S0735-1097(20)33157-0.

294. Park HS, Shin KS, Son BR, Kim DM, Kim HS, Jeong HW. Human Granulocytic Anaplasmosis Diagnosed Based on a Peripheral Blood Smear Test in South Korea: a Case Report. Jpn J Infect Dis. 2020;73(6):469-72. Epub 2020/06/02. doi: 10.7883/yoken.JJID.2020.013. PubMed PMID: 32475869.

295. Rivera JE, Young K, Kwon TS, McKenzie PA, Grant MA, McBride DA. Anaplasmosis Presenting With Respiratory Symptoms and Pneumonitis. Open Forum Infect Dis. 2020;7(8):ofaa265. Epub 2020/08/15. doi: 10.1093/ofid/ofaa265. PubMed PMID: 32793764; PubMed Central PMCID: PMCPMC7415301.

296. Rocco JM, Mallarino-Haeger C, McCurry D, Shah N. Severe anaplasmosis represents a treatable cause of secondary hemophagocytic lymphohistiocytosis: Two cases and review of literature. Ticks Tick Borne Dis. 2020;11(5):101468. Epub 2020/07/30. doi: 10.1016/j.ttbdis.2020.101468. PubMed PMID: 32723647.

297. Saha BK, Chieng H, Itty R, Bonnier A, Shkolnik B, Beegle S. Previously Unreported Presentation of Anaplasmosis in an Endemic Area: A Report of 2 Cases. Infectious Diseases in Clinical Practice. 2020;28(1):40-3. doi: 10.1097/IPC.0000000000000794.

298. Stokes W, Lisboa LF, Lindsay LR, Fonseca K. Case Report: Anaplasmosis in Canada: Locally Acquired <it>Anaplasma phagocytophilum</it> Infection in Alberta. American Journal of Tropical Medicine and Hygiene. 2020;103(6):2478-80. doi: 10.4269/ajtmh.20-0603. PubMed PMID: WOS:000605084900055.

299. Sykes DB, Zhang EW, Leaf RSK, Nardi V, Turbett SE. Case 10-2020: An 83-Year-Old Man with Pancytopenia and Acute Renal Failure. New England Journal of Medicine. 2020;382(13):1258-66. doi: 10.1056/NEJMcpc1916250. PubMed PMID: WOS:000522357300013.

300. Tsang D, Perlman D. A case report of fever of unknown origin from concomitant acute, IgM negative PCR positive anaplasmosis and west nile virus infection. Journal of General Internal Medicine. 2020;35(SUPPL 1):S350-S1. doi: 10.1007/s11606-020-05890-3.

301. Tsang P. Anaplasmosis: Diagnostic clues for an elusive emerging infection. Modern Pathology. 2020;33(3):1431-2.

302. Yoo J, Chung J-H, Kim C-M, Yun NR, Kim D-M. Asymptomatic-anaplasmosis confirmation using genetic and serological tests and possible coinfection with spotted fever group Rickettsia: a case report. BMC Infectious Diseases. 2020;20(1):1-6. doi: 10.1186/s12879-020-05170-9. PubMed PMID: 144314167. Language: English. Entry Date: In Process. Revision Date: 20200709. Publication Type: journal article.

303. Abdelwahab N, Schmidt T, Ingraham NE, Macdonald DM, Bruen CA. COVID anticks: Not all infiltrates are COVID related. American Journal of Respiratory and Critical Care Medicine. 2021;203(9). doi: 10.1164/ajrccm-conference.2021.203.1_MeetingAbstracts.A2450.

304. Al Amri R, Rea B. Anaplasmosis with associated haemophagocytic lymphohistiocytosis. Br J Haematol. 2021;194(4):657. Epub 2021/05/13. doi: 10.1111/bjh.17453. PubMed PMID: 33977517.

305. Camprubí-Ferrer D, Portillo A, Santibáñez S, Almuedo-Riera A, Rodriguez-Valero N, Subirà C, et al. Incidence of human granulocytic anaplasmosis in returning travellers with fever. Journal of Travel Medicine. 2021;28(4):1-6. doi: 10.1093/jtm/taab056. PubMed PMID: 150610494. Language: English. Entry Date: 20211116. Revision Date: 20221003. Publication Type: journal article.

306. Cho JM, Chang J, Kim D-M, Kwak YG, Cho CR, Song JE. Human granulocytic anaplasmosis combined with rhabdomyolysis: a case report. BMC Infectious Diseases. 2021;21(1):1-5. doi: 10.1186/s12879-021-06869-z. PubMed PMID: 153786065. Language: English. Entry Date: In Process. Revision Date: 20211201. Publication Type: journal article.

307. Eldaour Y, Hariri R, Yassin M. Severe Anaplasmosis presenting as possible CVA: Case report and 3-year Anaplasma infection diagnosis data is based on PCR testing and serology. IDCases. 2021;24:e01073. Epub 2021/04/15. doi: 10.1016/j.idcr.2021.e01073. PubMed PMID: 33850717; PubMed Central PMCID: PMCPMC8022154.

308. Finnin D, Hanowitz C. A Traumatic Tick Bite: A Case Report. Clin Pract Cases Emerg Med. 2021;5(2):210-3. Epub 2021/08/27. doi: 10.5811/cpcem.2021.3.50514. PubMed PMID: 34437007; PubMed Central PMCID: PMCPMC8143818 are required to disclose all affiliations, funding sources and financial or management relationships that could be perceived as potential sources of bias. The authors disclosed none.

309. Grant L, Mohamedy I, Loertscher L. One man, three tick-borne illnesses. BMJ Case Rep. 2021;14(4). Epub 2021/04/18. doi: 10.1136/bcr-2020-241004. PubMed PMID: 33863772; PubMed Central PMCID: PMCPMC8055128.

310. Helsen G, De Cauwer H, Van Sonhoven F. Acute brachial plexitis caused by human granulocytic anaplasmosis. Acta Neurol Belg. 2021;121(2):597-8. Epub 2021/02/15. doi: 10.1007/s13760-021-01611-7. PubMed PMID: 33582895.

311. Horowitz HW, Behar C, Greene J. COVID-19 and the Consequences of Anchoring Bias. Emerg Infect Dis. 2021;27(8):2235-6. Epub 2021/07/22. doi: 10.3201/eid2708.211107. PubMed PMID: 34287136; PubMed Central PMCID: PMCPMC8314836.

312. Hsia K, Johnson J, Rice D. Splenomegaly, Non-Traumatic Splenic Rupture, and Pancytopenia in Patient with Human Granulocytic Anaplasmosis. R I Med J (2013). 2021;104(2):60-2. Epub 2021/03/03. PubMed PMID: 33648322.

313. Jalal A. 131 AN UNUSUAL CASE OF RENAL FAILURE ASSOCIATED WITH ANAPLASMOSIS - A CASE REPORT. American Journal of Kidney Diseases. 2021;77(4):608. doi: 10.1053/j.ajkd.2021.02.136.

314. Kalter J, Heffelfinger S, Veet CA. Anaplasmosis presenting as dyspnea, myalgias, arthralgias, and elevated transaminases in the SARS-CoV-2 pandemic. Journal of General Internal Medicine. 2021;36(SUPPL 1):S198. doi: 10.1007/s11606-021-06830-5.

315. Khan RRH, Zaki R, Zaki A, Mojarrab JN, Zahid A. Transient Arrhythmia in a Patient With Human Granulocytic Anaplasmosis: An Uncanny Presentation. Cureus. 2021;13(2). doi: 10.7759/cureus.13241. PubMed PMID: WOS:000617066900007.

316. Khera KD, Southerland DM, Miller NE, Garrison GM. A Case of Anaplasmosis during a Warm Minnesota Fall. J Prim Care Community Health. 2021;12:21501327211005895. Epub 2021/03/26. doi: 10.1177/21501327211005895. PubMed PMID: 33764206; PubMed Central PMCID: PMCPMC8772355.

317. Kim DY, Seo JW, Yun NR, Kim CM, Kim DM. Human granulocytic anaplasmosis in a Single University Hospital in the Republic of Korea. Sci Rep. 2021;11(1):10860. Epub 2021/05/27. doi: 10.1038/s41598-021-90327-y. PubMed PMID: 34035378; PubMed Central PMCID: PMCPMC8149831.

318. Kurian T, Makam S. A case of anaplasmosis during the COVID-19 pandemic. Consultant. 2021;61(7):E24-E5. doi: 10.25270/con.2020.11.00006.

319. Ladzinski AT, Baker M, Dunning K, Patel PP. Human Granulocytic Anaplasmosis presenting as Subacute Abdominal Pain and Hyponatremia. IDCases. 2021;25:e01183. Epub 2021/07/01. doi: 10.1016/j.idcr.2021.e01183. PubMed PMID: 34189035; PubMed Central PMCID: PMCPMC8220232.

320. Lazo K, Ashok S, Kaltsas A, Van Elden L. A VERY 'TICKY' DIAGNOSIS OF PNEUMONIA IN A PATIENT WITH CANCER. Chest. 2021;160(4):A272. doi: 10.1016/j.chest.2021.07.280.

321. Mangat R, Winbush A, Louie T. Recurrent Fevers in a Triathlete. Cureus. 2021;13(1):e12564. Epub 2021/02/13. doi: 10.7759/cureus.12564. PubMed PMID: 33575137; PubMed Central PMCID: PMCPMC7869908.

322. Moniuszko-Malinowska A, Dunaj J, Andersson MO, Chmielewski T, Czupryna P, Groth M, et al. Anaplasmosis in Poland - analysis of 120 patients. Ticks Tick Borne Dis. 2021;12(5):101763. Epub 2021/06/24. doi: 10.1016/j.ttbdis.2021.101763. PubMed PMID: 34161867.

323. Mullholand JB, Tolman N, De Obaldia A, Hennrikus E. Central nervous system involvement of anaplasmosis. BMJ Case Rep. 2021;14(12). Epub 2021/12/10. doi: 10.1136/bcr-2021-243665. PubMed PMID: 34880034; PubMed Central PMCID: PMCPMC8655545.

324. Ramanujam D, Nasrullah A, Bahr M, Ashraf O, Malik K. Human Granulocytic Anaplasmosis as a COVID-19 Mimicker. Eur J Case Rep Intern Med. 2021;8(12):003047. Epub 2022/01/22. doi: 10.12890/2021_003047. PubMed PMID: 35059343; PubMed Central PMCID: PMCPMC8765683.

325. Šimeková K, Soják Ľ, Víchová B, Balogová L, Jarošová J, Antolová D. Parasitic and Vector-Borne Infections in HIV-Positive Patients in Slovakia-Evidence of an Unexpectedly High Occurrence of Anaplasma phagocytophilum. Pathogens. 2021;10(12). Epub 2021/12/29. doi: 10.3390/pathogens10121557. PubMed PMID: 34959511; PubMed Central PMCID: PMCPMC8704717.

326. Sosa-Gutierrez CG, Cervantes-Castillo MA, Laguna-Gonzalez R, Lopez-Echeverria LY, Ojeda-Ramírez D, Oyervides M. Serological and Molecular Evidence of Patients Infected with Anaplasma phagocytophilum in Mexico. Diseases. 2021;9(2). Epub 2021/06/03. doi: 10.3390/diseases9020037. PubMed PMID: 34069232; PubMed Central PMCID: PMCPMC8161817.

327. Stice MJ, Bruen CA, Grall KJH. Anchoring on COVID-19: A Case Report of Human Granulocytic Anaplasmosis Masquerading as COVID-19. Clin Pract Cases Emerg Med. 2021;5(3):328-31. Epub 2021/08/27. doi: 10.5811/cpcem.2021.4.51970. PubMed PMID: 34437040; PubMed Central PMCID: PMCPMC8373177 are required to disclose all affiliations, funding sources and financial or management relationships that could be perceived as potential sources of bias. The authors disclosed none.

328. Wormser GP, Jacobson E, Shanker EM. Negative impact of the COVID-19 pandemic on the timely diagnosis of tick-borne infections. Diagn Microbiol Infect Dis. 2021;99(1):115226. Epub 2020/10/19. doi: 10.1016/j.diagmicrobio.2020.115226. PubMed PMID: 33070027; PubMed Central PMCID: PMCPMC7518953.

329. Anthony JA, Jordanovski D, Furer SK. Lyme carditis presenting with an incessant atrioventricular nodal reentrant tachycardia masking a variable atrioventricular block. HeartRhythm Case Reports. 2022;8(12):829-35. doi: 10.1016/j.hrcr.2022.09.006.

330. Campeau L, Roy V, Petit G, Baron G, Blouin J, Carignan A. Identification d'un agrégat inhabituel d'anaplasmose granulocytaire humaine dans la région de l'Estrie, Québec, Canada en 2021. Revue d'Epidemiologie et de Sante Publique. 2022;70:S226-S7. doi: 10.1016/j.respe.2022.06.235.

331. Capp K, Kealy H. A COVID-positive patient with ARDS secondary to anaplasmosis. Critical Care Medicine. 2022;50(1 SUPPL):89. doi: 10.1097/01.ccm.0000807164.41368.a0.

332. Chang FY, Wang RY, Yen TY, Shu PY, Yang SL. Human case of Anaplasma phagocytophilum infection in Eastern Taiwan. J Formos Med Assoc. 2022. Epub 2022/09/13. doi: 10.1016/j.jfma.2022.08.018. PubMed PMID: 36096862.

333. de Jesus M, Lopez A, Yabut J, Vu S, Manne M, Ibrahim L, et al. Anaplasmosis-induced hemophagocytic lymphohistiocytosis. Proc (Bayl Univ Med Cent). 2022;35(3):379-81. Epub 2022/05/07. doi: 10.1080/08998280.2022.2039046. PubMed PMID: 35518814; PubMed Central PMCID: PMCPMC9037399.

334. Duron O, Koual R, Musset L, Buysse M, Lambert Y, Jaulhac B, et al. Novel Chronic Anaplasmosis in Splenectomized Patient, Amazon Rainforest. Emerg Infect Dis. 2022;28(8):1673-6. Epub 2022/07/26. doi: 10.3201/eid2808.212425. PubMed PMID: 35876693; PubMed Central PMCID: PMCPMC9328922.

335. Fisher M, Heller A, Psevdos G. Fatigue and myalgias after a tick bite. Infectious Diseases in Clinical Practice. 2022;30(1):1. doi: 10.1097/IPC.0000000000001066.

336. Kandhi S, Ghazanfar H, Qureshi ZA, Kalangi H, Jyala A, Arguello Perez ES. An Atypical Presentation of a Severe Case of Anaplasma Phagocytophilum. Cureus. 2022;14(3):e23224. Epub 2022/04/23. doi: 10.7759/cureus.23224. PubMed PMID: 35449628; PubMed Central PMCID: PMCPMC9012425.

337. Ladha D, Khalife R, Hummel B, Purssell A. Human granulocytic anaplasmosis complicated by hemophagocytic syndrome and coinfection. Cmaj. 2022;194(49):E1685-e8. Epub 2022/12/20. doi: 10.1503/cmaj.220638. PubMed PMID: 36535681; PubMed Central PMCID: PMCPMC9829056.

338. LeDonne MJ, Ahmed SA, Keeney SM, Nadworny H. Trigeminal Neuralgia As the Principal Manifestation of Anaplasmosis: A Case Report. Cureus. 2022;14(1):e21668. Epub 2022/03/04. doi: 10.7759/cureus.21668. PubMed PMID: 35237471; PubMed Central PMCID: PMCPMC8882039.

339. Leikauskas JA, Read JS, Kelso P, Heitman KN, Armstrong PA, Kwit NA. Anaplasmosis-Related Fatality in Vermont: A Case Report. Vector-Borne and Zoonotic Diseases. 2022;22(3):188-90. doi: 10.1089/vbz.2021.0095. PubMed PMID: WOS:000768007500001.

340. Levy AM, Martin LM, Krakower DS, Grandin EW. Case report: human granulocytic anaplasmosis causes acute myopericarditis with atrial fibrillation. European Heart Journal-Case Reports. 2022;7(1). doi: 10.1093/ehjcr/ytad026. PubMed PMID: WOS:000923159500010.

341. Lu M, Chen Q, Qin X, Lyu Y, Teng Z, Li K, et al. Anaplasma bovis Infection in Fever and Thrombocytopenia Patients - Anhui Province, China, 2021. China CDC Wkly. 2022;4(12):249-53. Epub 2022/04/19. doi: 10.46234/ccdcw2022.053. PubMed PMID: 35433083; PubMed Central PMCID: PMCPMC9005486.

342. Merati M, Rucker JC, McKeon A, Frucht SJ, Hu J, Balcer LJ, et al. A Case of Opsoclonus-Myoclonus-Ataxia With Neuronal Intermediate Filament IgG Detected in Cerebrospinal Fluid. J Neuroophthalmol. 2022;42(2):278-81. Epub 2022/05/21. doi: 10.1097/wno.0000000000001599. PubMed PMID: 35594157; PubMed Central PMCID: PMCPMC9620397.

343. Nykytyuk S, Klymnyuk S, Panichev V, Marchuk O, Klishch I. Experience of PCR research on Lyme borreliosis in children from the Ternopil Region. Family Medicine and Primary Care Review. 2022;24(4):334-5. doi: 10.5114/fmpcr.2022.120857.

344. Singh A, Simeone SN, Cohn G, Cox J. ACUTE HUMAN GRANULOCYTIC ANAPLASMOSIS REQUIRING INTENSIVE CARE HOSPITALIZATION PRESENTING AS URTICARIAL RASH. Journal of General Internal Medicine. 2022;37:S393. doi: 10.1007/s11606-022-07653-8.

345. Song D, Almas T, Abdelghffar M, Jain S, Geetha HS, Shah V, et al. A rare case of delayed anaplasma phagocytophilum-induced pancytopenia: A diagnostic conundrum. Ann Med Surg (Lond). 2022;75:103366. Epub 2022/02/25. doi: 10.1016/j.amsu.2022.103366. PubMed PMID: 35198193; PubMed Central PMCID: PMCPMC8851287.

346. Spitalska E, Boldisova E, Palkovicova K, Sekeyova Z, Skultety L. Case studies of rickettsiosis, anaplasmosis and Q fever in Slovak population from 2011 to 2020. Biologia. 2022;77(6):1633-40. doi: 10.1007/s11756-021-00838-2. PubMed PMID: WOS:000674557700004.

347. Taşdemir C, Şimşek S, Önal U, Çetinkaya H, Aydın L, Yılmaz E. A difficult diagnosis of anaplasmosis with pneumonia: A case report. Trop Doct. 2022:494755221112737. Epub 2022/07/28. doi: 10.1177/00494755221112737. PubMed PMID: 35892170.

348. Ungar SP, Varkey J, Pierro J, Raetz E, Ratner AJ. Do Not Forget About the Ticks: An Unusual Cause of Fever, GI Distress, and Cytopenias in a Child With ALL. J Pediatr Hematol Oncol. 2022;44(5):e901-e4. Epub 2021/12/23. doi: 10.1097/mph.0000000000002369. PubMed PMID: 34935737.

349. Vyas JM, Castle AC, Bourgouin PP, Turbett SE. Case 9-2022: A 56-Year-Old Woman with Fever, Myalgias, Diarrhea, and Cough. N Engl J Med. 2022;386(12):1166-74. Epub 2022/03/24. doi: 10.1056/NEJMcpc2115846. PubMed PMID: 35320647.

350. Yabut J, De Jesus M, Kumar M. Hemophagocytic Lymphohistiocytosis-Associated Stress Cardiomyopathy. American Journal of Respiratory and Critical Care Medicine. 2022;205(1). doi: 10.1164/ajrccm-conference.2022.205.1_MeetingAbstracts.A2826.

351. Zhang Y, Chen T, Polimera H, Evans M, Bayerl MG, George MR. Hemophagocytic Lymphohistiocytosis induced by human granulocytic anaplasmosis: A case report and literature review into the immunopathogenesis. Human Pathology Reports. 2022;27. doi: 10.1016/j.hpr.2022.300598.

352. Acharya S, Montano FC, Riaz A, Juers C, Macqueen D. TICK-BORNE ILLNESS TICKING OFF THE HEART: A CASE OF CARDIOMYOPATHY DUE TO ANAPLASMOSIS. Journal of the American College of Cardiology. 2023;81(8):2657. doi: 10.1016/S0735-1097(23)03101-7.

353. Banović P, Piloto-Sardiñas E, Mijatović D, Foucault-Simonin A, Simin V, Bogdan I, et al. Differential detection of tick-borne pathogens in human platelets and whole blood using microfluidic PCR. Acta Tropica. 2023;238. doi: 10.1016/j.actatropica.2022.106756.

354. Bush V, Chaudhary J, Manu D, Hyman C. A rare occurrence of Anaplasma-associated peritonitis. Lab Med. 2023. Epub 2023/03/28. doi: 10.1093/labmed/lmad016. PubMed PMID: 36972513.

355. Dogra M, Thakur M, Kumar A, Thakur G. Tick-Borne Rhabdomyolysis: A Rare Case of Rhabdomyolysis and Acute Kidney Injury Due to Anaplasmosis. Cureus. 2023;15(2):e34835. Epub 2023/03/16. doi: 10.7759/cureus.34835. PubMed PMID: 36919073; PubMed Central PMCID: PMCPMC10008484.

356. Kartashov MY, Gladysheva AV, Shvalov AN, Tupota NL, Chernikova AA, Ternovoi VA, et al. Novel Flavi-like virus in ixodid ticks and patients in Russia. Ticks Tick Borne Dis. 2023;14(2):102101. Epub 2022/12/19. doi: 10.1016/j.ttbdis.2022.102101. PubMed PMID: 36529011.

357. Mahmoud AA, Abdelhay A, Eltaher B. Anaplasmosis and Lyme disease. Journal of Hematopathology. 2023;16(1):57-8. doi: 10.1007/s12308-022-00525-4.

358. Nigrovic LE, Neville DN, Chapman L, Balamuth F, Levas MN, Thompson AD, et al. Multiplex High-Definition Polymerase Chain Reaction Assay for the Diagnosis of Tick-borne Infections in Children. Open Forum Infect Dis. 2023;10(4):ofad121. Epub 2023/04/24. doi: 10.1093/ofid/ofad121. PubMed PMID: 37089773; PubMed Central PMCID: PMCPMC10114523.

359. Sampat HN, Sharma A, Nussbaum EZ. Case 5-2023: A 67-Year-Old Man with Interstitial Lung Disease, Fever, and Myalgias. New England Journal of Medicine. 2023;388(7):642-50. doi: 10.1056/NEJMcpc2211364.

360. Schiller D, Aufreiter L, Schöfl R. [Fever of unusual cause]. Rev Med Interne. 2023;44(3):146-7. Epub 2022/12/20. doi: 10.1016/j.revmed.2022.11.007. PubMed PMID: 36535845.

Excluded (duplicates removed)

1. Rees CW. THE EXPERIMENTAL TRANSMISSION OF ANAPLASMOSIS BY DERMACENTOR VARIABILIS. Science. 1932;75(1942):318-20. Epub 1932/03/18. doi: 10.1126/science.75.1942.318. PubMed PMID: 17779663.

2. Boynton WH, Woods GM. DEER AS CARRIERS OF ANAPLASMOSIS. Science. 1933;78(2033):559-60. Epub 1933/12/15. doi: 10.1126/science.78.2033.559. PubMed PMID: 17811937.

3. Boynton WH, Woods GM. ANAPLASMOSIS AMONG DEER IN THE NATURAL STATE. Science. 1940;91(2355):168. Epub 1940/02/16. doi: 10.1126/science.91.2355.168. PubMed PMID: 17807414.

4. Benhamou E. [Rickettsia conori in the bone marrow of a patient with pimple fever]. C R Seances Soc Biol Fil. 1945;139:688. Epub 1945/08/01. PubMed PMID: 21023460.

5. Leonova NA. [On the possibility of the transmission by lice of the spirochetes of tick recurrent fever Spirochaeta uzbekistanica (Sp. sogdianum)]. Med Parazitol (Mosk). 1945;14(3):79-82. Epub 1945/01/01. PubMed PMID: 20280628.

6. Pavlovsky EN. [Natural endemicity of tick recurrent fever in the Turkoman Socialist Soviet Republic]. Med Parazitol (Mosk). 1945;14(3):56-9. Epub 1945/01/01. PubMed PMID: 20280623.

7. Pavlovsky EN, Kuzmina LA. [On the possibility of the transmission of the spirochetes of tick recurrent fever by Ornithodorus lahorensis to monkeys and man]. Med Parazitol (Mosk). 1945;14(3):66-70. Epub 1945/01/01. PubMed PMID: 20280625.

8. Sofiev MS, Leonova NA. [New data on reservoirs of the virus of tick recurrent fever in the Uzbek Socialist Soviet Republic]. Med Parazitol (Mosk). 1945;14(3):60-5. Epub 1945/01/01. PubMed PMID: 20280624.

9. Earle KV. Pyrexia associated with tickbite. J Trop Med Hyg. 1946;49:14. Epub 1946/02/01. PubMed PMID: 21018595.

10. Florio L, Mugrage ER, Stewart MO. Colorado tick fever. Ann Intern Med. 1946;25:466-72. Epub 1946/09/01. doi: 10.7326/0003-4819-25-3-466. PubMed PMID: 20997003.

11. Koprowski H, Cox HR. Adaptation of Colorado tick fever virus to mouse and developing chick embryo. Proc Soc Exp Biol Med. 1946;62(2):320-2. Epub 1946/06/01. doi: 10.3181/00379727-62-15465. PubMed PMID: 20993212.

12. Livesay HR, Wilson DJ. Experimental studies of Bullis fever and dengue fever. The American journal of tropical medicine and hygiene. 1946;26:379-81.

13. Lotze JC. Further observations on the nature of anaplasma. Proceedings of the Helminthological Society of Washington. 1946;13(2):56.

14. Osburn LW. Cross-immunity between South African epidemic (louse-borne) typhus and murine (flea-born) typhus. S Afr J Med Sci. 1946;11(2-3):73-7. Epub 1946/11/01. PubMed PMID: 20282008.

15. Pollard M, Livesay HR, et al. Immunological studies of dengue fever and Colorado tick fever. Proc Soc Exp Biol Med. 1946;61:396-8. Epub 1946/04/01. doi: 10.3181/00379727-61-15331. PubMed PMID: 20982508.

16. Pollard M, Livesay HR, et al. Experimental studies with Bullis fever. Am J Trop Med Hyg. 1946;26:175-87. Epub 1946/03/01. doi: 10.4269/ajtmh.1946.s1-26.175. PubMed PMID: 21020338.

17. Smith HC, Howell DE. Age a factor in anaplasmosis. Veterinary medicine. 1946;41(11):407.

18. Sussman LN. Kew Gardens spotted fever. NY Med. 1946;2(15):27. Epub 1946/08/05. PubMed PMID: 20993050.

19. COLORADO tick fever. Lab Dig. 1947;10(12):2. Epub 1947/05/01. PubMed PMID: 20242512.

20. De BC, Kunz LJ, et al. Specific complement-fixing diagnostic antigens for Colorado tick fever. Proc Soc Exp Biol Med. 1947;64(2):202-8. Epub 1947/02/01. PubMed PMID: 20287378.

21. Florio L, Stewart MO. Colorado tick fever. Am J Public Health Nations Health. 1947;37(3):293-7. Epub 1947/03/01. PubMed PMID: 20288954.

22. Florio L, Stewart MO. The etiology of Colorado tick fever and an immunological comparison to dengue. Rocky Mt Med J. 1947;44(2):129. Epub 1947/02/01. PubMed PMID: 20287402.

23. Lotze JC. Variables and constants in experimental bovine anaplasmosis and their relationship to chemotherapy. American journal of veterinary research. 1947;8(28):267-74.

24. Lotze JC. Blood transfusions in bovine anaplasmosis. American journal of veterinary research. 1947;8(28):284-8.

25. Nikolaevskaia ZS. [Chemotherapy of the tick fever; therapeutic effect of sulfonamides]. Biull Eksp Biol Med. 1947;24(7):67-70. Epub 1947/01/01. PubMed PMID: 18913974.

26. Stauffer VD. Sodium cacodylate and arsenic trioxide in anaplasmosis of cattle. The North American veterinarian. 1947;28(4):219-21.

27. Dikmans G. Anaplasmosis. Abstracts International Congress on Tropical Medicine and Malaria (4th. 1948;56(4 th Congr):112.

28. Florio L, Miller MS. Epidemiology of Colorado tick fever. Am J Public Health Nations Health. 1948;38(2):211-3. Epub 1948/02/01. doi: 10.2105/ajph.38.2.211. PubMed PMID: 18905851; PubMed Central PMCID: PMCPMC1624163.

29. Tagliacozzo L. Not available. Bollettino della Società italiana di biologia sperimentale. 1948;24(6):746.

30. Arnold WT, Van Noate HF. Bullis fever; report of a case treated with para-aminobenzoic acid. Bull U S Army Med Dep. 1949;9(3):218-23. Epub 1949/03/01. PubMed PMID: 18111162.

31. Boiron H. [Considerations on the relapsing tick fever in Senegal; importance of the rat as a virus reservoir]. Sud Med Chir. 1949;81(2318):1160-3. Epub 1949/08/31. PubMed PMID: 18148753.

32. Splitter EJ. Anaplasmosis treatment with paludrine; a case report. Journal of the American Veterinary Medical Association. 1949;114(865):224.

33. Contreras Poza L. [Some commentaries on the case of "Olmer's exanthematous fever treated with chloromycetin"]. Medicamenta (Madr). 1950;8(181):311. Epub 1950/05/01. PubMed PMID: 15429621.

34. de MO. [Percentage of ticks carrying in nature, V.B., V.A.1 and V.A.2 strains of neotropic exanthematic typhus in Brazil]. Bras Med. 1950;64(22-30):85-6. Epub 1950/06/01. PubMed PMID: 15434213.

35. Galant IB. [Tick encephalitis in children]. Vopr Pediatrii. 1950;18(3):25-7. Epub 1950/01/01. PubMed PMID: 24538286.

36. Koprowski H, Cox HR, Miller MS, Florio L. Response of man to egg-adapted Colorado tick fever virus. Proc Soc Exp Biol Med. 1950;74(1):126-31. Epub 1950/05/01. doi: 10.3181/00379727-74-17830. PubMed PMID: 15430410.

37. Lacorte JG. [Pantropic viruses; tick fever virus]. Rev Bras Med. 1950;7(6):385-8. Epub 1950/06/01. PubMed PMID: 15441330.

38. Martin Borreguero A. [Studies on exanthematous Mediterranean fever; hexamethylenetetramine and vitamin B1 therapy]. Medicamenta (Madr). 1950;8(192):389. Epub 1950/12/10. PubMed PMID: 14805760.

39. Semler L. Tick fever in western Washington. Northwest Med. 1950;49(3):183. Epub 1950/03/01. PubMed PMID: 15406099.

40. Splitter EJ. Eperythrozoon suis, the etiologic agent of ictero-anemia or an anaplasmosis-like disease in swine. American journal of veterinary research. 1950;11(40):324-30.

41. T PJ, de MJ, Monteiro EL, Brandao CH, Barreto Neto LP. [Macular fever]. An Paul Med Cir. 1950;60(4):308-9. Epub 1950/10/01. PubMed PMID: 14790278.

42. de Robertis E, Epstein B. Electron microscope study of anaplasmosis in bovine red blood cells. Proceedings of the Society for Experimental Biology and Medicine Society for Experimental Biology and Medicine (New York, NY). 1951;77(2):254-8.

43. Foote LE, Brock WE, Gallaher B. Ictero-anemia, eperythrozoonosis, or anaplasmosis-like disease of swine proved to be caused by a filtrable virus. The North American veterinarian. 1951;32(1):17-23.

44. Grashchenkov NI, Gurvich AM, Fedorchuk LV. [Nature of tick encephalitis encountered in the Belorussian Soviet Republic]. Nevropatol Psikhiatriia. 1951;20(2):36-46. Epub 1951/03/01. PubMed PMID: 14843319.

45. Pavlovskii EN, Skrynnik AN. [Certain biologic properties of ticks Ornithodorus, carriers of tick recurrent typhus]. Dokl Akad Nauk SSSR. 1951;78(5):1069-72. Epub 1951/06/11. PubMed PMID: 14859994.

46. Donatien A. Premunitive vaccination against bovine anaplasmosis. Archives de l'Institut Pasteur d'Algérie Institut Pasteur. 1952;30(1):44-6.

47. Price KE, Poelma LJ, Faber JE. Preparation of an improved antigen for anaplasmosis complement-fixation. American journal of veterinary research. 1952;13(47):149-51.

48. Brock WE, Pearson CC, Kliewer IO. High-level aureomycin dosage in anaplasmosis. American journal of veterinary research. 1953;14(53):510-3.

49. Jadin J, Panier E. [A rickettsial virus of the boutonneuse fever-tick fever type isolated in Rouanda-Urundi]. Ann Soc Belg Med Trop (1920). 1953;33(2):119-22. Epub 1953/04/01. PubMed PMID: 13092708.

50. Rossi P, Triozon F. Experiments on the culture of a bovine strain of Anaplasma marginale. Bulletin de la Société de pathologie exotique et de ses filiales. 1953;46(3):312-5.

51. Miller JG. The production and identification of an anaplasmosis complement-fixing antibody. American journal of veterinary research. 1954;15(54):147-8.

52. Price KE, Brock WE, Miller JG. An evaluation of the complement-fixation test for anaplasmosis. American journal of veterinary research. 1954;15(57):511-6.

53. Weyer F. [Observations in a strain of South African tick fever]. Z Tropenmed Parasitol. 1954;5(2):205-18. Epub 1954/04/01. PubMed PMID: 13196517.

54. Moulton JE, Christensen JF. The histochemical nature of Anaplasma marginale. American journal of veterinary research. 1955;16(60):377-80.

55. Splitter EJ, Twiehaus MJ, Castro ER. Anaplasmosis in sheep in the United States. Journal of the American Veterinary Medical Association. 1955;127(942):244-5.

56. Weyer F. [A laboratory infection with tick bite fever]. Z Tropenmed Parasitol. 1955;6(2):226-30. Epub 1955/06/01. PubMed PMID: 13257556.

57. Splitter EJ, Anthony HD, Twiehaus MJ. Anaplasma ovis in the United States; experimental studies with sheep and goats. American journal of veterinary research. 1956;17(64):487-91.

58. Brock WE, Pearson CC, Staley EE, Kliewer IO. The prevention of anaplasmosis by feeding chlortetracycline. Journal of the American Veterinary Medical Association. 1957;130(10):445-6.

59. Foote LE, Levy HE, Torbert BJ, Oglesby WT. Interference between anaplasmosis and eperythrozoonosis in splenectomized cattle. American journal of veterinary research. 1957;18(68):556-9.

60. Gates DW, Madden PA, Martin WH, Roby TO. The infectivity of blood from Anaplasma-infected cattle as shown by. American journal of veterinary research. 1957;18(67):257-60.

61. Pearson CC, Brock WE, Kliewer IO. A study of tetracycline dosage in cattle which are anaplasmosis. Journal of the American Veterinary Medical Association. 1957;130(7):290-2.

62. Ristic M, White FH, Sanders DA. Detection of Anaplasma marginale by means of fluorescein-labeled antibody. American journal of veterinary research. 1957;18(69):924-8.

63. Splitter EJ, Anthony HD, Twiehaus MJ. Survey of anaplasmosis reactors in Kansas; a preliminary report. Journal of the American Veterinary Medical Association. 1957;130(10):447-8.

64. Arline RE, Mamelli JA. Laboratory studies of anaplasmosis in cattle treated with oxytetracycline. Journal of the American Veterinary Medical Association. 1958;133(10):517-9.

65. Christensen JF, Osebold JW, Rosen MN. Infection and antibody response in deer experimentally infected with Anaplasma marginale from bovine carriers. Journal of the American Veterinary Medical Association. 1958;132(7):289-92.

66. Foote LE, Geer JC, Stich YE. Electron microscopy of the Anaplasma body: ultra-thin sections of bovine erythrocytes. Science (New York, NY). 1958;128(3316):147-8.

67. Franklin TE, Redmond HE. Observations on the morphology of Anaplasma marginale with reference to projections or tails. American journal of veterinary research. 1958;19(70):252-3.

68. Ryff JF, Gilbert CS, Weibel JL, Breen H. Anaplasmosis and concurrent copper intoxication in sheep. Journal of the American Veterinary Medical Association. 1958;133(6):312-5.

69. Thomas GM. The incidence of anaplasmosis in Wyoming. Journal of the American Veterinary Medical Association. 1958;132(2):61-2.

70. Weyer F. [Observations on transmission of Brazilian spotted fever & Siberian tick fever to body lice]. Z Tropenmed Parasitol. 1958;9(3):174-93. Epub 1958/09/01. PubMed PMID: 13604761.

71. Espana C, Espana EM, Gonzalez D. Anaplasma marginale. I. Studies with phase contrast and electron microscopy. American journal of veterinary research. 1959;20:795-805.

72. Osebold JW, Christensen JF, Longhurst WM, Rosen MN. Latent Anaplasma marginale infection in wild deer demonstrated by calf inoculation. The Cornell veterinarian. 1959;49(1):97-115.

73. Schindler R. [Anaplasmosis]. Z Tropenmed Parasitol. 1959;10:164-77. Epub 1959/08/01. PubMed PMID: 14442798.

74. Weyer F. [Experiments with a strain of tick-bite fever from north Queensland]. Schweiz Z Pathol Bakteriol. 1959;22:609-20. Epub 1959/01/01. PubMed PMID: 13844238.

75. Christensen JF, Osebold JW, Harrold JB, Rosen MN. Persistence of latent Anaplasma marginale infection in deer. Journal of the American Veterinary Medical Association. 1960;136:426-7.

76. Dimopoullos GT, Bedell DM. Studies of bovine erythrocytes in anaplasmosis. I. Flocculating properties of stromata in water. Proceedings of the Society for Experimental Biology and Medicine Society for Experimental Biology and Medicine (New York, NY). 1960;105:463-6.

77. Dimopoullos GT, Schrader GT, Foote LE. Electrophoretic studies of bovine serum. III. Serum protein changes in anaplasmosis. American journal of veterinary research. 1960;21:222-5.

78. Ristic M. Studies of anaplasmosis. I. Filtration of the causative agent. American journal of veterinary research. 1960;21:890-4.

79. Ristic M. Structural characterization of Anaplasma marginale in acute and carrier infections. Journal of the American Veterinary Medical Association. 1960;136:417-25.

80. Ristic M, White FH. Detection of an Amplasma marginale antibody complex formed in vivo. Science. 1960;131:987-8.

81. Baker NF, Osebold JW, Christensen JF. Erythrocyte survival in experimental anaplasmosis. American journal of veterinary research. 1961;22:590-6.

82. Bedell DM, Oglesby WT. Effect of neomycin on experimentally induced anaplasmosis in splenectomized calves. Journal of the American Veterinary Medical Association. 1961;139:349-51.

83. Gainer JH. Demonstration of Anaplasma marginale with the fluorescent dye, acridine orange; comparisons with the complement-fixation test and Wright's stain. American journal of veterinary research. 1961;22:882-6.

84. Kreier JP, Ristic M. Studies in anaplasmosis. IV. Development of the causative agent in deer erythrocytes transfused into calves. American journal of veterinary research. 1961;22:790-4.

85. Pilcher KS, Wu WG, Muth OH. Studies on the morphology and respiration of Anaplasma marginale. American journal of veterinary research. 1961;22:298-307.

86. Roby TO, Gates DW. The comparative susceptibility of calves and adult cattle to bovine anaplasmosis. American journal of veterinary research. 1961;22:982-5.

87. Scott WL, Geer JC, Foote LE. Electron microscopy of Anaplasma marginale in the bovine erythrocyte. American journal of veterinary research. 1961;22:877-81.

88. Allbritton AR, Parker LT. Filtration of the infective agent of bovine anaplasmosis. American journal of veterinary research. 1962;23:809-12.

89. Allbritton AR, Seger CL. The transport and excretion of bile pigments in anaplasmosis. American journal of veterinary research. 1962;23:101-8.

90. Bedell DM, Dimopoullos GT. Biologic properties and characteristics of Anaplasma marginale. I. Effects of temperature on infectivity of whole blood preparations. American journal of veterinary research. 1962;23:618-25.

91. Dimopoullos GT, Bedell DM. Studies of bovine erythrocytes in anaplasmosis. II. Role of chemical and physical changes in erythrocytes in the mechanism of anemia in splenectomized calves. American journal of veterinary research. 1962;23:813-20.

92. Kuttler KL, Marble DW, Matthews NJ. Anaplasmosis complement-fixation response in calves from. American journal of veterinary research. 1962;23:1007-10.

93. Merriman GM, Buckner C, Hobbs CS. Field trial with the complement-fixation test for anaplasmosis in a herd free of clinical evidence of infection. Journal of the American Veterinary Medical Association. 1962;141:1335-8.

94. Ristic M. A capillary tube-agglutination test for anaplasmosis--a preliminary report. Journal of the American Veterinary Medical Association. 1962;141:588-94.

95. Ristic M, Watrach AM. Studies in anaplasmosis. V. Occurrence of Anaplasma marginale in bovine blood platelets. American journal of veterinary research. 1962;23:626-31.

96. Ritchie AE. Simplified specimen preparation for electron microscopic studies of an intra-erythrocytic parasite, Anaplasma marginale. Proceedings of the Society for Experimental Biology and Medicine Society for Experimental Biology and Medicine (New York, NY). 1962;110:532-5.

97. Rogers TE, Dimopoullos GT. Electrophoretic studies of bovine serum. V. Complement-fixing antibodies in serum fractions in anaplasmosis. Proceedings of the Society for Experimental Biology and Medicine Society for Experimental Biology and Medicine (New York, NY). 1962;110:359-62.

98. Salvin SB. Specificity of allergic reaction. V. Observations on the systemic delayed reaction in guinea pigs sensitized to purified protein-conjugates. Journal of immunology (Baltimore, Md : 1950). 1962;89:910-9.

99. Welter CJ, Zuschek F. Properties of Anaplasma marginale antigen used in a capillary tube-agglutination test. Journal of the American Veterinary Medical Association. 1962;141:595-9.

100. Bedell DM, Dimopoullos GT. Biologic properties and characteristics of Anaplasma marginale. II. The effects of sonic energy on the infectivity of whole blood preparations. American journal of veterinary research. 1963;24:278-82.

101. Kreier JP, Ristic M. Anaplasmosis. X. Morphologic characteristics of the parasites present in the blood of calves infected with the Oregon strain of Anaplasma marginale. American journal of veterinary research. 1963;24:676-87.

102. Kreier JP, Ristic M. Anaplasmosis. XI. Immunoserologic characteristics of the parasites present in the blood of calves infected with the Oregon strain of Anaplasma marginale. American journal of veterinary research. 1963;24:688-96.

103. Krier JP, Ristic M. Anaplasmosis. VII. Experimental Anaplasma ovis infection in white-tailed deer (Dama virginiana). American journal of veterinary research. 1963;24:567-72.

104. Kuttler KL. COMPARISONS OF COMPLEMENT-FIXATION AND CAPILLARY TUBE-AGGLUTINATION TESTS FOR DETECTION OF BOVINE ANAPLASMOSIS. Journal of the American Veterinary Medical Association. 1963;143:729-33.

105. Malherbe WD. Some observations on anaplasmosis. The Cornell veterinarian. 1963;53:71-7.

106. Ristic M, Kreier JP. ANAPLASMOSIS. XV. MORPHOLOGIC AND CHEMICAL PROPERTIES OF THE ANTIGEN USED IN THE CAPILLARY TUBE-AGGLUTINATION TEST. American journal of veterinary research. 1963;24:985-92.

107. Ristic M, Mann DK. Anaplasmosis. IX. Immunoserologic properties of soluble Anaplasma antigens. American journal of veterinary research. 1963;24:478-82.

108. Ristic M, Mann DK, Kodras R. Anaplasmosis. VIII. Biochemical and biophysical characterization of soluble Anaplasma antigens. American journal of veterinary research. 1963;24:472-7.

109. Ristic M, Watrach AM. Anaplasmosis. VI. Studies and a hypothesis concerning the cycle of development of the causative agent. American journal of veterinary research. 1963;24:267-77.

110. Rogers TE, Dimopoullos GT. STUDIES OF BOVINE ERYTHROCYTES IN ANAPLASMOSIS. IV. EFFECTS OF SONIC VIBRATION ON ANAPLASMA COMPLEMENT-FIXING ANTIGEN. Journal of bacteriology. 1963;86:160-1.

111. Schrader GT, Dimopoullos GT. Studies of bovine erythrocytes in anaplasmosis. III. Partition of erythrocytic phospholipids. American journal of veterinary research. 1963;24:283-6.

112. Amerault TE, Roby TO. AN EXO-ANTIGEN OF ANAPLASMA MARGINALE IN SERUM AND ERYTHROCYTES OF CATTLE WITH ACUTE ANAPLASMOSIS. American journal of veterinary research. 1964;25:1642-7.

113. Franklin TE, Huff JW. A RAPID COMPLEMENT FIXATION SCREENING PROCEDURE FOR ANAPLASMOSIS TESTING. American journal of veterinary research. 1964;25:1321-2.

114. Merriman GM, Owens LK, Chung PK. COMPLEMENT-FIXATION AND CAPILLARY-AGGLUTINATION TESTS IN AN ANAPLASMOSIS-INFECTED HERD OF CATTLE. Journal of the American Veterinary Medical Association. 1964;145:258-62.

115. Rogers TE, Hidalgo RJ, Dimopoullos GT. IMMUNOLOGY AND SEROLOGY OF ANAPLASMA MARGINALE. I. FRACTIONATION OF THE COMPLEMENT-FIXING ANTIGEN. Journal of bacteriology. 1964;88:81-6.

116. Ryff JF, Breen H, Thomas GM. CONTROL OF ANAPLASMOSIS UNDER WYOMING CONDITIONS. Journal of the American Veterinary Medical Association. 1964;145:43-6.

117. Ryff JF, Weibel JL, Thomas GM. RELATIONSHIP OF OVINE TO BOVINE ANAPLASMOSIS. The Cornell veterinarian. 1964;54:407-14.

118. Brock WE, Norman BB, Kliewer IO, Jones EW. AUTOANTIBODY STUDIES IN BOVINE ANAPLASMOSIS. American journal of veterinary research. 1965;26:250-3.

119. Franklin TE, Huff JW, Grumbles LC. CHLORTETRACYCLINE FOR ELIMINATION OF ANAPLASMOSIS IN CARRIER CATTLE. Journal of the American Veterinary Medical Association. 1965;147:353-6.

120. Rogers TE, Dimopoullos GT. Immunology and serology of Anaplasma marginale. II. Nature of the CF antigen. Proc Soc Exp Biol Med. 1965;120(3):685-9. Epub 1965/12/01. doi: 10.3181/00379727-120-30625. PubMed PMID: 5858692.

121. Schroeder WF, Ristic M. ANAPLASMOSIS. 18. AN ANALYSIS OF AUTOANTIGENS IN INFECTED AND NORMAL. American journal of veterinary research. 1965;26:679-82.

122. Summers WA. A rapid procedure for preparing fluorescein-isothiocyanate-labeled anti-Anaplasma gamma globulin. Am J Vet Res. 1965;26(115):1459-62. Epub 1965/11/01. PubMed PMID: 4160492.

123. Thomas GM, Radford BS. ANAPLASMOSIS IN WYOMING. Journal of the American Veterinary Medical Association. 1965;146:224.

124. Wiesenhuetter E. STUDIES ON THEILERIASIS AND OTHER BLOOD INFECTIONS IN CATTLE IN SYRIA. Berliner und Münchener tierärztliche Wochenschrift. 1965;78:31-2.

125. Valentine BL. Preservation of dimethylsulfoxide-treated Anaplasma marginale with liquid nitrogen. J Bacteriol. 1966;91(6):2385. Epub 1966/06/01. doi: 10.1128/jb.91.6.2385-.1966. PubMed PMID: 5943945; PubMed Central PMCID: PMCPMC316224.

126. David E, Unger RR. [On the problems of malignant degeneration of gliomas of the cerebrum (II)]. Arch Geschwulstforsch. 1967;30(2):135-41. Epub 1967/01/01. PubMed PMID: 4298606.

127. Gates DW, Roby TO, Amerault TE, Anthony DW. Ultrastructure of Anaplasma marginale fixed with glutaraldehyde and osmium tetroxide. Am J Vet Res. 1967;28(126):1577-80. Epub 1967/09/01. PubMed PMID: 4167894.

128. Wallace WR, Dommert AR, Dimopoullos GT. Fractionation of lipids of Anaplasma marginale. Am J Vet Res. 1967;28(123):443-6. Epub 1967/03/01. PubMed PMID: 6033695.

129. Garnham PC, Donnelly J, Hoogstraal H, Kennedy CC, Walton GA. Human babesiosis in Ireland: further observations and the medical significance of this infection. Br Med J. 1969;4(5686):768-70. Epub 1969/12/27. doi: 10.1136/bmj.4.5686.768. PubMed PMID: 4902496; PubMed Central PMCID: PMCPMC1630245.

130. Jatkar PR. Electron microscopic study of Anaplasma ovis. Am J Vet Res. 1969;30(10):1891-2. Epub 1969/10/01. PubMed PMID: 5824911.

131. Giroud P, Fiocre JB, Capponi M, Dumas N, Ryter A, Jadin J. [Positive sero-reactions with the neorickettsial group (Bedsonia, Cytoecetes phagocytaphilia) observed in animals parasitized with Anaplasma, Babesia, Theileria, in different countries: Oriental and Central Africa, Madagascar, Iran, Sardinia, France]. Bull Soc Pathol Exot Filiales. 1970;63(6):630-5. Epub 1970/11/01. PubMed PMID: 5537906.

132. D'Iakonov L P, Nadtocheĭ GA. [Anaplasma ultrastructure]. Veterinariia. 1971;10:70-5. Epub 1971/10/01. PubMed PMID: 5152088.

133. Fife EH, Jr. Current state of serological tests used to detect blood parasite infections. Exp Parasitol. 1972;31(1):136-52. Epub 1972/02/01. doi: 10.1016/0014-4894(72)90056-2. PubMed PMID: 4622044.

134. Kallick CA, Levin S, Reddi KT, Landau WL. Systemic lupus erythematosus associated with haemobartonella-like organisms. Nat New Biol. 1972;236(66):145-6. Epub 1972/04/05. doi: 10.1038/newbio236145a0. PubMed PMID: 4112548.

135. Trub A. [Incertae sedis parasites in comparative pathology]. Bull Soc Pathol Exot Filiales. 1972;65(4):506-33. Epub 1972/01/01. PubMed PMID: 4196197.

136. Miescher PA. Immunosuppressive therapy. Clinica Terapeutica. 1973;66(6):557-87.

137. Parker R. A direct counting technique for estimating high parasitaemias in infections of Babesia argentina, Babesia bigemina and Plasmodium berghei. Annals of Tropical Medicine and Parasitology. 1973;67(4):387-90.

138. Dimopoullos GT, Bedell DM. Mechanism and specificity of the capillary tube agglutination test for diagnosis of anaplasmosis. American Journal of Veterinary Research. 1974;35(12):1567-70.

139. Hadani A, Pipano E, Tsafrir N. The transmission of Babesia bigemina, Babesiella berbera and Anaplasma centrale by Boophilus annulatus (Say). Refuah Veterinarith. 1974;31(4):149-54.

140. Hidalgo RJ. Culture of Anaplasma marginale in lymph node cells. Proc Annu Meet U S Anim Health Assoc. 1974;(78):77-85. Epub 1974/01/01. PubMed PMID: 4534775.

141. Johns RW, Dimopoullos GT. In vitro uptake of 14C labeled amino acids by preparations of partially purified Anaplasma marginale bodies. Infection and Immunity. 1974;9(4):645-7.

142. Kovi J, Tillman RL, Lee SM. Malignant transformation of condyloma acuminatum. A light microscopic and ultrastructural study. Am J Clin Pathol. 1974;61(5):702-10. Epub 1974/05/01. doi: 10.1093/ajcp/61.5.702. PubMed PMID: 4827053.

143. McHardy N, Gilson C. An electron microscope study of diagnostic antigens prepared from Anaplasma marginale. TROPENMEDPARASIT. 1974;25(1):11-21.

144. Callow LL, Quiroga QC, McCosker PJ. Serological comparison of Australian and South American strains of Babesia argentina and Anaplasma marginale. International Journal for Parasitology. 1976;6(4):307-10. doi: 10.1016/0020-7519(76)90051-5.

145. Krinsky WL. Animal disease agents transmitted by horse flies and deer flies (Diptera: Tabanidae). J Med Entomol. 1976;13(3):225-75. Epub 1976/12/08. doi: 10.1093/jmedent/13.3.225. PubMed PMID: 137982.

146. Ristic M. Immunologic systems and protection in infections caused by intracellular blood protista. Veterinary Parasitology. 1976;2(1):31-47. doi: 10.1016/0304-4017(76)90051-0.

147. Ajayi SA, Wilson AJ, Campbell RSF. Experimental bovine anaplasmosis: clinico-pathological and nutritional studies. Research in Veterinary Science. 1978;25(1):76-81.

148. Gretillat S, Konarzewski B. Presence of a procaryote belonging to the genus Haemobartonella Tyzzer and Wainman, 1939, in the blood of Nigerians in the Niamey area. Bulletin de la Societe de Pathologie Exotique et de ses Filiales. 1978;71(6):412-6.

149. Parker R, Parker ML, Wilson AJ. Liquid nitrogen storage of Anaplasma marginale complement-fixation antigen by a multiple small aliquot technique. Res Vet Sci. 1978;25(3):401-2. Epub 1978/11/01. PubMed PMID: 749095.

150. Todorovic R, Gonzalez E, Lopez G. Immunization against anaplasmosis and babesiosis. Part II. Evaluation of cryo-preserved vaccines using different doses and routes of inoculation. Tropenmedizin und Parasitologie. 1978;29(2):210-4.

151. Vizcaino O, Carson CA, Lee AJ, Ristic M. Efficacy of attenuated Anaplasma marginale vaccine under laboratory and field conditions in Colombia. American Journal of Veterinary Research. 1978;39(2):299-33. PubMed Central PMCID: PMCAnchor(United States)

Fort Dodge(United States)

philips roxanne(United States)

Squibb(United States).

152. Henson JB, Noel JC. Immunology and Pathogenesis of African Animal Trypanosomiasis. Advances in Veterinary Science and Comparative Medicine1979. p. 161-82.

153. Swift BL, Reeves JD, 3rd, Thomas GM. Testicular degeneration and libido loss in beef bulls experimentally inoculated with Anaplasma marginale. Theriogenology. 1979;11(4):277-90. Epub 1979/04/01. doi: 10.1016/0093-691x(79)90070-0. PubMed PMID: 16725412.

154. Uilenberg G, van Vorstenbosch CJ, Perié NM. Blood parasites of sheep in the Netherlands. I. Anaplasma mesaeterum sp.n. (Rickettsiales, Anaplasmataceae). Vet Q. 1979;1(1):14-22. Epub 1979/01/01. doi: 10.1080/01652176.1979.9693716. PubMed PMID: 22040472.

155. Archer GL, Coleman PH, Cole RM, Duma RJ, Johnston CL, Jr. Hemotropic bacteria. N Engl J Med. 1980;302(20):1151-2. Epub 1980/05/15. doi: 10.1056/nejm198005153022018. PubMed PMID: 7366649.

156. Kallick CA, Thadhani KC, Rice TW. Identification of Anaplasmataceae (Haemobartonella) antigen and antibodies in systemic lupus erythematosus. Arthritis Rheum. 1980;23(2):197-205. Epub 1980/02/01. doi: 10.1002/art.1780230210. PubMed PMID: 6153897.

157. Lawrence JA, Foggin CM, Norval RA. The effects of war on the control of diseases of livestock in Rhodesia (Zimbabwe). Vet Rec. 1980;107(4):82-5. Epub 1980/07/26. doi: 10.1136/vr.107.4.82. PubMed PMID: 7445371.

158. Nicholls MJ, Ibata G, Rodas FV. Prevalence of antibodies to Babesia bovis and Anaplasma marginale in dairy cattle in Bolivia. Tropical Animal Health and Production. 1980;12(1):48-9. doi: 10.1007/BF02242630.

159. Uilenberg G, Rombach MC, Perié NM, Zwart D. Blood parasites of sheep in the Netherlands. II. Babesia motasi (Sporozoa, Babesiidae). Vet Q. 1980;2(1):3-14. Epub 1980/01/01. doi: 10.1080/01652176.1980.9693752. PubMed PMID: 22039853.

160. Akinboade OA, Dipeolu OO, Ogunji FO, Adegoke GO. The parasites obtained and bacteria isolated from house rats (Rattus rattus Linnaeus, 1758) caught in human habitations in Ibadan, Nigeria. International Journal of Zoonoses. 1981;8(1):26-32.

161. Kreier JP, Ristic M. The biology of hemotrophic bacteria. Annual review of microbiology. 1981;35:325-38. doi: 10.1146/annurev.mi.35.100181.001545.

162. Montenegro S, James MA, Levy MG. Utilization of culture-derived soluble antigen in the latex agglutination test for bovine babesiosis and anaplasmosis. Veterinary Parasitology. 1981;8(4):291-7. doi: 10.1016/0304-4017(81)90061-3.

163. Phillips PE. The potential role of microbial agents in the pathogenesis of systemic lupus erythematosus. J Rheumatol. 1981;8(2):344-7. Epub 1981/03/01. PubMed PMID: 6262512.

164. Rodwell BJ, Howard RJ. A fluorescent method for counting Babesia, Anaplasma, Plasmodium and Trypanosoma following DNA staining with 33258 Hoechst (bisbenzimide). Annals of Tropical Medicine and Parasitology. 1981;75(2):123-9.

165. Bundza A, Samagh BS. Acute anaplasmosis in imported cattle. Can Vet J. 1982;23(11):337-9. Epub 1982/11/01. PubMed PMID: 17422202; PubMed Central PMCID: PMCPMC1790232.

166. Ginger C. Antiparasitic Agents. Annual Reports in Medicinal Chemistry1982. p. 129-38.

167. Kocan KM, Ewing SA, Holbert D, Hair A. Morphologic characteristics of colonies of Anaplasma marginale Theiler in midgut epithelial cells of Dermacentor andersoni Stiles. American Journal of Veterinary Research. 1982;43(4):586-93.

168. Woldehiwet Z, Scott GR. In vitro propagation of Cytoecetes phagocytophila, the causative agent of tick-borne fever. Veterinary Microbiology. 1982;7(2):127-33. doi: 10.1016/0378-1135(82)90024-4.

169. Barbet AF, Anderson LW, Palmer GH, McGuire TC. Comparison of proteins synthesized by two different isolates of Anaplasma marginale. Infect Immun. 1983;40(3):1068-74. Epub 1983/06/01. doi: 10.1128/iai.40.3.1068-1074.1983. PubMed PMID: 6852911; PubMed Central PMCID: PMCPMC348159.

170. Janitschke K, De Vos AJ, Bigalke RD. Serodiagnosis of bovine besnoitiosis by ELISA and immunofluorescence tests. Onderstepoort Journal of Veterinary Research. 1984;51(4):239-43.

171. Kuttler KL, Winward LD. Serologic comparisons of 4 Anaplasma isolates as measured by the complement-fixation test. Veterinary Microbiology. 1984;9(2):181-6. doi: 10.1016/0378-1135(84)90033-6.

172. MacDiarmid SC, Durham PJ, Jones DL. Survey confirms freedom from anaplasmosis. N Z Vet J. 1984;32(9):156-7. Epub 1984/09/01. doi: 10.1080/00480169.1984.35105. PubMed PMID: 16031087.

173. Swanepoel R, Shepherd AJ, Leman PA, Shepherd SP, Miller GB. A common-source outbreak of Crimean-Congo haemorrhagic fever on a dairy farm. S Afr Med J. 1985;68(9):635-7. Epub 1985/10/26. PubMed PMID: 3933131.

174. Zwart D. Haemoparasitic diseases of bovines. Rev Sci Tech. 1985;4(3):447-78. Epub 1985/09/01. doi: 10.20506/rst.4.3.208. PubMed PMID: 32736453.

175. Akinboade OA, Sadiq NA, Akinrinmade JF. Anaplasmosis of small ruminants in Nigeria: Incidence and parasite identification through blood smear and latex agglutination test (LAT). International Journal of Zoonoses. 1986;13(3):210-4.

176. Fox JC, Jordan HE, Kocan KM, George TJ, Mullins ST, Barnett CE, et al. An overview of serological tests currently available for laboratory diagnosis of parasitic infections. Vet Parasitol. 1986;20(1-3):13-29. Epub 1986/03/01. doi: 10.1016/0304-4017(86)90089-0. PubMed PMID: 3518213.

177. Puntaric V, Borcic D, Vukelic D. Eperythrozoonosis in man. Lancet. 1986;2(8511):868-9.

178. Chema S, Chumo RS, Dolan TT, Gathuma JM, Irvin AD, James AD, et al. Clinical trial of halofuginone lactate for the treatment of East Coast fever in Kenya. Veterinary Record. 1987;120(24):575-7. doi: 10.1136/vr.120.24.575.

179. Nantulya VM, Musoke AJ, Rurangirwa FR, Saigar N, Minja SH. Monoclonal antibodies that distinguish Trypanosoma congolense, T. vivax and T. brucei. Parasite Immunol. 1987;9(4):421-31. Epub 1987/07/01. doi: 10.1111/j.1365-3024.1987.tb00520.x. PubMed PMID: 3306569.

180. Oberle SM, Palmer GH, Barbet AF, McGuire TC. Molecular size variations in an immunoprotective protein complex among isolates of Anaplasma marginale. Infect Immun. 1988;56(6):1567-73. Epub 1988/06/01. doi: 10.1128/iai.56.6.1567-1573.1988. PubMed PMID: 3372021; PubMed Central PMCID: PMCPMC259437.

181. Puntaric V, Borcic D, Vukelic D, Jeren T, Burek V, Wikerhauser T, et al. Eperythrozoonosis: A new disease in human pathology. Lijecnicki Vjesnik. 1988;110(3-4):85-7.

182. Barbet AF. Vaccines for parasitic infections. Adv Vet Sci Comp Med. 1989;33:345-75. Epub 1989/01/01. doi: 10.1016/b978-0-12-039233-9.50014-7. PubMed PMID: 2648776.

183. Chevrel B. Infectious diseases: congress of the Brazilian society for infectology. Medecine et Chirurgie Digestives. 1989;18(2):96-7.

184. Murray PK. Molecular vaccines against animal parasites. Vaccine. 1989;7(4):291-9.

185. Odiawo GO, Motelin GK. Hyperthermia associated with anaplasmosis in a dairy cow in Kenya. N Z Vet J. 1989;37(2):84. Epub 1989/06/01. doi: 10.1080/00480169.1989.35567. PubMed PMID: 16031527.

186. Aboytes-Torres R, Buening GM. Development of a recombinant Anaplasma marginale DNA probe. Veterinary Microbiology. 1990;24(3-4):391-408.

187. Allred DR, McGuire TC, Palmer GH, Leib SR, Harkins TM, McElwain TF, et al. Molecular basis for surface antigen size polymorphisms and conservation of a neutralization-sensitive epitope in Anaplasma marginale. Proc Natl Acad Sci U S A. 1990;87(8):3220-4. Epub 1990/04/01. doi: 10.1073/pnas.87.8.3220. PubMed PMID: 1691504; PubMed Central PMCID: PMCPMC53867.

188. Mangold AJ, Aguirre DH, Guglielmone AA. Post-thawing viability of vaccines for bovine babesiosis and anaplasmosis cryopreserved with glycerol. Veterinary Parasitology. 1990;37(3-4):301-6.

189. Rodriquez S, Romano AM, Aranguren E, Soyano Y. Erratum: 'A new hemolytic assay for bovine serum complement and its application during experimental bovine anaplasmosis', (Vet. Immunol. Immunopathol., 24:347-360.) (1990)). Veterinary Immunology and Immunopathology. 1990;25(3):288.

190. Thaker SR, Dutta SK, Adhya SL, Mattingly-Napier BL. Molecular cloning of Ehrlichia risticii and development of a gene probe for the diagnosis of potomac horse fever. Journal of Clinical Microbiology. 1990;28(9):1963-7. doi: 10.1128/jcm.28.9.1963-1967.1990.

191. Anderson BE, Dawson JE, Jones DC, Wilson KH. Ehrlichia chaffeensis, a new species associated with human ehrlichiosis. J Clin Microbiol. 1991;29(12):2838-42. Epub 1991/12/01. doi: 10.1128/jcm.29.12.2838-2842.1991. PubMed PMID: 1757557; PubMed Central PMCID: PMCPMC270443.

192. Barbet AF, Allred DR. The msp1 beta multigene family of Anaplasma marginale: nucleotide sequence analysis of an expressed copy. Infect Immun. 1991;59(3):971-6. Epub 1991/03/01. doi: 10.1128/iai.59.3.971-976.1991. PubMed PMID: 1671779; PubMed Central PMCID: PMCPMC258354.

193. Haider MJ. Anaplasma marginale in Lepus nigricollis (Blanford) treated with oxytetracycline. Pak J Pharm Sci. 1991;4(1):83-5. Epub 1991/01/01. PubMed PMID: 16414686.

194. Rikihisa Y. The tribe Ehrlichieae and ehrlichial diseases. Clin Microbiol Rev. 1991;4(3):286-308. Epub 1991/07/01. doi: 10.1128/cmr.4.3.286. PubMed PMID: 1889044; PubMed Central PMCID: PMCPMC358200.

195. Visser ES, Ambrosio RE, De Waal DT. An Anaplasma centrale DNA probe that differentiates between Anaplasma ovis and Anaplasma marginale DNA. Veterinary Microbiology. 1991;28(3):313-25. doi: 10.1016/0378-1135(91)90086-U.

196. Weisburg WG, Barns SM, Pelletier DA, Lane DJ. 16S ribosomal DNA amplification for phylogenetic study. J Bacteriol. 1991;173(2):697-703. Epub 1991/01/01. doi: 10.1128/jb.173.2.697-703.1991. PubMed PMID: 1987160; PubMed Central PMCID: PMCPMC207061.

197. Anderson BE, Greene CE, Jones DC, Dawson JE. Ehrlichia ewingii sp. nov., the etiologic agent of canine granulocytic ehrlichiosis. Int J Syst Bacteriol. 1992;42(2):299-302. Epub 1992/04/01. doi: 10.1099/00207713-42-2-299. PubMed PMID: 1581189.

198. Dame JB, Mahan SM, Yowell CA. Phylogenetic relationship of Cowdria ruminantium, agent of heartwater, to Anaplasma marginale and other members of the order Rickettsiales determined on the basis of 16S rRNA sequence. Int J Syst Bacteriol. 1992;42(2):270-4. Epub 1992/04/01. doi: 10.1099/00207713-42-2-270. PubMed PMID: 1581187.

199. Montenegro-James S. Prevalence and control of babesiosis in the Americas. Mem Inst Oswaldo Cruz. 1992;87 Suppl 3:27-36. Epub 1992/01/01. doi: 10.1590/s0074-02761992000700003. PubMed PMID: 1343700.

200. Orinda GO, Waltisbuhl DJ, Goodger BV, Wright IG. Serological and immunological studies with a hexane extract of Babesia bovis-infected erythrocytes. International Journal for Parasitology. 1992;22(5):677-9. doi: 10.1016/0020-7519(92)90019-H.

201. Peters JM, Dalrymple BP, Jorgensen WK. Sequence of a putative glutathione synthetase II gene and flanking regions from Anaplasma centrale. Biochem Biophys Res Commun. 1992;182(3):1040-6. Epub 1992/02/14. doi: 10.1016/0006-291x(92)91836-f. PubMed PMID: 1540152.

202. Ssenyonga GSZ, Kakoma I, Montenegro-James S, Nyeko PJ, Nanteza A, Buga R. Anaplasmosis in Uganda. II. Prevalence of bovine anaplasmosis. Scandinavian Journal of Immunology, Supplement. 1992;36(11):107-9.

203. Visser ES, McGuire TC, Palmer GH, Davis WC, Shkap V, Pipano E, et al. The Anaplasma marginale msp5 gene encodes a 19-kilodalton protein conserved in all recognized Anaplasma species. Infection and Immunity. 1992;60(12):5139-44.

204. Jemal A, Hughesjones M. A REVIEW OF THE RED IMPORTED FIRE ANT (SOLENOPSIS-INVICTA BUREN) AND ITS IMPACTS ON PLANT, ANIMAL, AND HUMAN HEALTH. Preventive Veterinary Medicine. 1993;17(1-2):19-32. doi: 10.1016/0167-5877(93)90051-t. PubMed PMID: WOS:A1993MD61800003.

205. Oberle SM, Barbet AF. Derivation of the complete msp4 gene sequence of Anaplasma marginale without cloning. Gene. 1993;136(1-2):291-4. Epub 1993/12/22. doi: 10.1016/0378-1119(93)90482-i. PubMed PMID: 8294020.

206. Orinda GO, Wright IG, Leatch G, Young AS. HUMAN INTERFERON-ALPHA FAILS TO INHIBIT THE DEVELOPMENT OF BABESIA-BIGEMINA AND ANAPLASMA-MARGINALE INFECTIONS IN CATTLE. Veterinary Parasitology. 1993;47(1-2):149-55. doi: 10.1016/0304-4017(93)90185-p. PubMed PMID: WOS:A1993KY34300016.

207. Reagan WJ. A REVIEW OF MYELOFIBROSIS IN DOGS. Toxicologic Pathology. 1993;21(2):164-9. doi: 10.1177/019262339302100208. PubMed PMID: WOS:A1993LR95300007.

208. Granulocytic ehrlichiosis: is it in your future? Emergency Medicine (00136654). 1994;26(13):37-. PubMed PMID: 107392329. Language: English. Entry Date: 19961201. Revision Date: 20150711. Publication Type: Journal Article. Journal Subset: Allied Health.

209. Aboytes-Torres R, Rodriguez SD, Vega CA. Molecular epidemiology of bovine anaplasmosis. Archives of Medical Research. 1994;25(2):247-52.

210. Arraga-Alvarado C. [Human ehrlichiosis. Review]. Invest Clin. 1994;35(4):209-22. Epub 1994/12/01. PubMed PMID: 7734523.

211. Brouqui P, Raoult D. Human ehrlichiosis. N Engl J Med. 1994;330(24):1760-1. Epub 1994/06/16. doi: 10.1056/nejm199406163302418. PubMed PMID: 8043106.

212. Chen SM, Dumler JS, Bakken JS, Walker DH. Identification of a granulocytotropic Ehrlichia species as the etiologic agent of human disease. J Clin Microbiol. 1994;32(3):589-95. Epub 1994/03/01. doi: 10.1128/jcm.32.3.589-595.1994. PubMed PMID: 8195363; PubMed Central PMCID: PMCPMC263091.

213. Drancourt M, Raoult D. Taxonomic position of the rickettsiae: current knowledge. FEMS Microbiol Rev. 1994;13(1):13-24. Epub 1994/01/01. doi: 10.1111/j.1574-6976.1994.tb00032.x. PubMed PMID: 8117465.

214. Dumler JS, Bakken JS, Eckman MR, Vanetta LL, Chen SM, Walker DH. HUMAN GRANULOCYTIC EHRLICHIOSIS - A NEW, POTENTIALLY FATAL TICK-BORNE INFECTION DIAGNOSED BY PERIPHERAL-BLOOD SMEAR AND PCR. Laboratory Investigation. 1994;70(1):A126-A. PubMed PMID: WOS:A1994MW42600745.

215. Jorgensen WK, Waldron SJ. Use of in vitro culture to isolate Babesia bovis from Theileria buffeli, Eperythrozoon wenyoni and Anaplasma spp. Veterinary Parasitology. 1994;53(1-2):45-51. doi: 10.1016/0304-4017(94)90015-9.

216. McGarey DJ, Barbet AF, Palmer GH, McGuire TC, Allred DR. Putative adhesins of Anaplasma marginale: Major surface polypeptides 1a and 1b. Infection and Immunity. 1994;62(10):4594-601.

217. Palmer GH, Eid G, Barbet AF, McGuire TC, McElwain TF. The immunoprotective Anaplasma marginale major surface protein 2 is encoded by a polymorphic multigene family. Infect Immun. 1994;62(9):3808-16. Epub 1994/09/01. doi: 10.1128/iai.62.9.3808-3816.1994. PubMed PMID: 8063397; PubMed Central PMCID: PMCPMC303035.

218. Peregrine AS. Chemotherapy and delivery systems: Haemoparasites. Veterinary Parasitology. 1994;54(1-3):223-48. doi: 10.1016/0304-4017(94)90092-2.

219. Rikihisa Y, Ewing SA, Fox JC. Western immunoblot analysis of Ehrlichia chaffeensis, E. canis, or E. ewingii infections in dogs and humans. J Clin Microbiol. 1994;32(9):2107-12. Epub 1994/09/01. doi: 10.1128/jcm.32.9.2107-2112.1994. PubMed PMID: 7814533; PubMed Central PMCID: PMCPMC263951.

220. Van Vliet AHM, Jongejan F, Van Kleef M, Van der Zeijst BAM. Molecular cloning, sequence analysis, and expression of the gene encoding the immunodominant 32-kilodalton protein of Cowdria ruminantium. Infection and Immunity. 1994;62(4):1451-6.

221. Vidotto MC, McGuire TC, McElwain TF, Palmer GH, Knowles DP, Jr. Intermolecular relationships of major surface proteins of Anaplasma marginale. Infect Immun. 1994;62(7):2940-6. Epub 1994/07/01. doi: 10.1128/iai.62.7.2940-2946.1994. PubMed PMID: 8005681; PubMed Central PMCID: PMCPMC302901.

222. Zakimi S, Tsuji N, Fujisaki K. Protein analysis of Anaplasma marginale and Anaplasma centrale by two-dimensional polyacrylamide gel electrophoresis. J Vet Med Sci. 1994;56(5):1025-7. Epub 1994/10/01. doi: 10.1292/jvms.56.1025. PubMed PMID: 7865577.

223. From the Centers for Disease Control and Prevention. Human granulocytic ehrlichiosis--New York, 1995. Jama. 1995;274(11):867. Epub 1995/09/20. PubMed PMID: 7674488.

224. Human granulocytic ehrlichiosis--New York, 1995. MMWR Morb Mortal Wkly Rep. 1995;44(32):593-5. Epub 1995/08/18. PubMed PMID: 7662028.

225. Erratum: Human granulocytic ehrlichiosis - New York, 1995 (Journal of the American Medical Association (1995) 274 (867)). JAMA. 1995;274(12):937. doi: 10.1001/jama.274.12.937.

226. Bakken JS, Dumler JS. TRANSMISSION OF HUMAN GRANULOCYTIC EHRLICHIOSIS - REPLY. Jama-Journal of the American Medical Association. 1995;273(1):23-. PubMed PMID: WOS:A1995PZ04200013.

227. Barbet AF. Recent developments in the molecular biology of anaplasmosis. Veterinary Parasitology. 1995;57(1-3):43-9. doi: 10.1016/0304-4017(94)03108-9.

228. Barlough JE, Madigan JE, DeRock E, Dumler JS, Bakken JS. Protection against Ehrlichia equi is conferred by prior infection with the human granulocytotropic Ehrlichia (HGE agent). J Clin Microbiol. 1995;33(12):3333-4. Epub 1995/12/01. doi: 10.1128/jcm.33.12.3333-3334.1995. PubMed PMID: 8586731; PubMed Central PMCID: PMCPMC228702.

229. Brouqui P, Dumler JS, Lienhard R, Brossard M, Raoult D. Human granulocytic ehrlichiosis in Europe. Lancet. 1995;346(8977):782-3. Epub 1995/09/16. doi: 10.1016/s0140-6736(95)91544-3. PubMed PMID: 7658904.

230. Dumler JS, Asanovich KM, Bakken JS, Richter P, Kimsey R, Madigan JE. Serologic cross-reactions among Ehrlichia equi, Ehrlichia phagocytophila, and human granulocytic Ehrlichia. J Clin Microbiol. 1995;33(5):1098-103. Epub 1995/05/01. doi: 10.1128/jcm.33.5.1098-1103.1995. PubMed PMID: 7542262; PubMed Central PMCID: PMCPMC228112.

231. Dumler JS, Bakken JS. Ehrlichial diseases of humans: emerging tick-borne infections. Clin Infect Dis. 1995;20(5):1102-10. Epub 1995/05/01. doi: 10.1093/clinids/20.5.1102. PubMed PMID: 7619983.

232. Fishbein DB, Dennis DT. Tick-borne diseases – a growing risk. New England Journal of Medicine. 1995;333(7):452-3. doi: 10.1056/NEJM199508173330711.

233. Johansson KE, Pettersson B, Uhlén M, Gunnarsson A, Malmqvist M, Olsson E. Identification of the causative agent of granulocytic ehrlichiosis in Swedish dogs and horses by direct solid phase sequencing of PCR products from the 16S rRNA gene. Research in Veterinary Science. 1995;58(2):109-12. doi: 10.1016/0034-5288(95)90061-6.

234. Knowles DP, Perryman LE, McElwain TF, Kappmeyer LS, Stiller D, Palmer GH, et al. Conserved recombinant antigens of Anaplasma marginale and Babesia equi for serologic diagnosis. Veterinary Parasitology. 1995;57(1-3):93-6. doi: 10.1016/0304-4017(94)03113-B.

235. Madigan JE, Richter PJ, Jr., Kimsey RB, Barlough JE, Bakken JS, Dumler JS. Transmission and passage in horses of the agent of human granulocytic ehrlichiosis. J Infect Dis. 1995;172(4):1141-4. Epub 1995/10/01. doi: 10.1093/infdis/172.4.1141. PubMed PMID: 7561199.

236. Magnarelli LA, Dumler JS, Anderson JF, Johnson RC, Fikrig E. Coexistence of antibodies to tick-borne pathogens of babesiosis, ehrlichiosis, and Lyme borreliosis in human sera. J Clin Microbiol. 1995;33(11):3054-7. Epub 1995/11/01. doi: 10.1128/jcm.33.11.3054-3057.1995. PubMed PMID: 8576376; PubMed Central PMCID: PMCPMC228637.

237. Magnarelli LA, Stafford KC, 3rd, Mather TN, Yeh MT, Horn KD, Dumler JS. Hemocytic rickettsia-like organisms in ticks: serologic reactivity with antisera to Ehrlichiae and detection of DNA of agent of human granulocytic ehrlichiosis by PCR. J Clin Microbiol. 1995;33(10):2710-4. Epub 1995/10/01. doi: 10.1128/jcm.33.10.2710-2714.1995. PubMed PMID: 8567911; PubMed Central PMCID: PMCPMC228561.

238. Michel R, Muller KD, Schmid EN. EHRLICHIA-LIKE ORGANISMS (KSL(1)) OBSERVED AS OBLIGATE INTRACELLULAR PARASITES OF SACCAMOEBA SPECIES. Endocytobiosis and Cell Research. 1995;11(1):69-80. PubMed PMID: WOS:A1995RR39000005.

239. Pancholi P, Kolbert CP, Mitchell PD, Reed KD, Jr., Dumler JS, Bakken JS, et al. Ixodes dammini as a potential vector of human granulocytic ehrlichiosis. J Infect Dis. 1995;172(4):1007-12. Epub 1995/10/01. doi: 10.1093/infdis/172.4.1007. PubMed PMID: 7561173.

240. Pretzman C, Ralph D, Stothard DR, Fuerst PA, Rikihisa Y. 16S rRNA gene sequence of Neorickettsia helminthoeca and its phylogenetic alignment with members of the genus Ehrlichia. International Journal of Systematic Bacteriology. 1995;45(2):207-11. doi: 10.1099/00207713-45-2-207.

241. Pruthi RK, Marshall WF, Wiltsie JC, Persing DH. Human Babesiosis. Mayo Clinic Proceedings. 1995;70(9):853-62. doi: 10.4065/70.9.853.

242. Reed KD, Mitchell PD, Persing DH, Kolbert CP, Cameron V, Bakken JS, et al. Transmission of human granulocytic ehrlichiosis [3]. JAMA. 1995;273(1):23. doi: 10.1001/jama.273.1.23.

243. Roux V, Raoult D. Phylogenetic analysis of the genus Rickettsia by 16S rDNA sequencing. Res Microbiol. 1995;146(5):385-96. Epub 1995/06/01. doi: 10.1016/0923-2508(96)80284-1. PubMed PMID: 8525055.

244. Uilenberg G. International collaborative research: Significance of tick-borne hemoparasitic diseases to world animal health. Veterinary Parasitology. 1995;57(1-3):19-41. doi: 10.1016/0304-4017(94)03107-8.

245. Van Vliet AHM, Van der Zeijst BAM, Camus E, Mahan SM, Martinez D, Jongejan F. Use of a specific immunogenic region on the Cowdria ruminantium MAP1 protein in a serological assay. Journal of Clinical Microbiology. 1995;33(9):2405-10. doi: 10.1128/jcm.33.9.2405-2410.1995.

246. Wormser G. HUMAN GRANULOCYTIC EHRLICHIOSIS - NEW-YORK, 1995 (VOL 274, PG 867, 1995). Jama-Journal of the American Medical Association. 1995;274(12):937-. PubMed PMID: WOS:A1995RV73400009.

247. Wormser G, McKenna D, Aguerorosenfeld M, Horowitz H, Munoz J, Nowakowski J, et al. HUMAN GRANULOCYTIC EHRLICHIOSIS - NEW-YORK, 1995 (REPRINTED FROM MMWR, VOL 44, PG 593, 1995). Jama-Journal of the American Medical Association. 1995;274(11):867-. PubMed PMID: WOS:A1995RU60300006.

248. Erratum: Direct cultivation of the causative agent of human granulocytic ehrlichiosis (New England Journal of Medicine (Jan 25, 1996) 334 (209-15)). New England Journal of Medicine. 1996;335(5):361. doi: 10.1056/NEJM199608013350522.

249. Alleman AR, Barbet AF. Evaluation of Anaplasma marginale major surface protein 3 (MSP3) as a diagnostic test antigen. Journal of Clinical Microbiology. 1996;34(2):270-6.

250. Bakken JS. Human granulocytic ehrlichiosis in the United States. Infections in Medicine. 1996;13(10):877-912.

251. Bakken JS, Krueth J, Tilden RL, Dumler JS, Kristiansen BE. Serological evidence of human granulocytic ehrlichiosis in Norway. Eur J Clin Microbiol Infect Dis. 1996;15(10):829-32. Epub 1996/10/01. doi: 10.1007/bf01701530. PubMed PMID: 8950565.

252. Bakken JS, Krueth JK, Lund T, Malkovitch D, Asanovich K, Dumler JS. Exposure to deer blood may be a cause of human granulocytic ehrlichiosis. Clin Infect Dis. 1996;23(1):198. Epub 1996/07/01. doi: 10.1093/clinids/23.1.198. PubMed PMID: 8816164.

253. Barlough JE, Madigan JE, DeRock E, Bigornia L. Nested polymerase chain reaction for detection of Ehrlichia equi genomic DNA in horses and ticks (Ixodes pacificus). Vet Parasitol. 1996;63(3-4):319-29. Epub 1996/06/01. doi: 10.1016/0304-4017(95)00904-3. PubMed PMID: 8966998.

254. Cornelissen A, Schetters TPM. Vaccines against protozoal diseases of veterinary importance. Fems Immunology and Medical Microbiology. 1996;15(2-3):61-72. doi: 10.1111/j.1574-695X.1996.tb00055.x. PubMed PMID: WOS:A1996VG61400001.

255. Dawson JE, Biggie KL, Warner CK, Cookson K, Jenkins S, Levine JF, et al. Polymerase chain reaction evidence of Ehrlichia chaffeensis, an etiologic agent of human ehrlichiosis, in dogs from southeast Virginia. American Journal of Veterinary Research. 1996;57(8):1175-9. PubMed PMID: WOS:A1996VA59000013.

256. Dawson JE, Warner CK, Standaert S, Olson JG. The interface between research and the diagnosis of an emerging tick-borne disease, human ehrlichiosis due to Ehrlichia chaffeensis. Arch Intern Med. 1996;156(2):137-42. Epub 1996/01/22. PubMed PMID: 8546547.

257. Edelman DC, Dumler JS. Evaluation of an Improved PCR Diagnostic Assay for Human Granulocytic Ehrlichiosis. Mol Diagn. 1996;1(1):41-9. Epub 1996/06/01. doi: 10.1054/modi00100041. PubMed PMID: 10330196.

258. Eid G, French DM, Lundgren AM, Barbet AF, McElwain TF, Palmer GH. Expression of major surface protein 2 antigenic variants during acute Anaplasma marginale rickettsemia. Infection and Immunity. 1996;64(3):836-41. doi: 10.1128/iai.64.3.836-841.1996.

259. Figueroa JV, Alvarez JA, Canto GJ, Ramos JA, Mosqueda JJ, Buening GM. Comparative sensitivity of two tests for the diagnosis of multiple hemoparasite infection of cattle. 1996. p. 117-27.

260. Gale KR, Gartside MG, Dimmock CM, Zakrzewski H, Leatch G. Peripheral blood lymphocyte proliferative responses in cattle infected with or vaccinated against Anaplasma marginale. Parasitology Research. 1996;82(6):551-62. doi: 10.1007/s004360050161.

261. Greig B, Asanovich KM, Armstrong PJ, Dumler JS. Geographic, clinical, serologic, and molecular evidence of granulocytic ehrlichiosis, a likely zoonotic disease, in Minnesota and Wisconsin dogs. J Clin Microbiol. 1996;34(1):44-8. Epub 1996/01/01. doi: 10.1128/jcm.34.1.44-48.1996. PubMed PMID: 8748270; PubMed Central PMCID: PMCPMC228727.

262. Keirans JE, Hutcheson HJ, Durden LA, Klompen JSH. Ixodes (Ixodes) scapularis (Acari: Ixodidae): Redescription of all active stages, distribution, hosts, geographical variation, and medical and veterinary importance. Journal of Medical Entomology. 1996;33(3):297-318. doi: 10.1093/jmedent/33.3.297. PubMed PMID: WOS:A1996UH44700005.

263. Keysary A, Waner T, Rosner M, Warner CK, Dawson JE, Zass R, et al. The first isolation, in vitro propagation, and genetic characterization of Ehrlichia canis in Israel. Vet Parasitol. 1996;62(3-4):331-40. Epub 1996/04/01. doi: 10.1016/0304-4017(95)00866-7. PubMed PMID: 8686178.

264. Khan A, Kakkar A, Lebovics E, Kapur S, Forseter G, Wormser G, et al. Liver biochemical profiles in human granulocytic ehrlichiosis (HGE). Gastroenterology. 1996;110(4):A1232-A. PubMed PMID: WOS:A1996UF73704906.

265. Lee EH, Rikihisa Y. Absence of tumor necrosis factor alpha, interleukin-6 (IL-6), and granulocyte-macrophage colony-stimulating factor expression but presence of IL-1beta, IL-8, and IL-10 expression in human monocytes exposed to viable or killed Ehrlichia chaffeensis. Infect Immun. 1996;64(10):4211-9. Epub 1996/10/01. doi: 10.1128/iai.64.10.4211-4219.1996. PubMed PMID: 8926090; PubMed Central PMCID: PMCPMC174358.

266. Madigan JE, Barlough JE, Dumler JS, Schankman NS, DeRock E. Equine granulocytic ehrlichiosis in Connecticut caused by an agent resembling the human granulocytotropic ehrlichia. J Clin Microbiol. 1996;34(2):434-5. Epub 1996/02/01. doi: 10.1128/jcm.34.2.434-435.1996. PubMed PMID: 8789032; PubMed Central PMCID: PMCPMC228814.

267. Maurin M, Raoult D. Optimum treatment of intracellular infection. Drugs. 1996;52(1):45-59. doi: 10.2165/00003495-199652010-00004.

268. Mitchell PD, Reed KD, Hofkes JM. Immunoserologic evidence of coinfection with Borrelia burgdorferi, Babesia microti, and human granulocytic Ehrlichia species in residents of Wisconsin and Minnesota. J Clin Microbiol. 1996;34(3):724-7. Epub 1996/03/01. doi: 10.1128/jcm.34.3.724-727.1996. PubMed PMID: 8904446; PubMed Central PMCID: PMCPMC228878.

269. Munderloh UG, Madigan JE, Dumler JS, Goodman JL, Hayes SF, Barlough JE, et al. Isolation of the equine granulocytic ehrlichiosis agent, Ehrlichia equi, in tick cell culture. Journal of Clinical Microbiology. 1996;34(3):664-70. doi: 10.1128/jcm.34.3.664-670.1996. PubMed PMID: WOS:A1996UD13700035.

270. Musoke AJ, Palmer GH, McElwain TF, Nene V, Imckeever D. Prospects for subunit vaccines against tick-borne diseases. British Veterinary Journal. 1996;152(6):621-39. doi: 10.1016/S0007-1935(96)80117-5.

271. Nielsen K, Smith P, Gall D, De Eshaide ST, Wagner G, Dajer A. Development and validation of an indirect enzyme immunoassay for detection of antibody to Anaplasma marginale in bovine sera. Veterinary Parasitology. 1996;67(3-4):133-42. doi: 10.1016/S0304-4017(96)01042-4.

272. Nuti M, Russino F, Grazioli D, Rombola P, Macri G, Lillini E. Anti-Ehrlichia antibodies in high-risk subjects living in the piedmont of the Veneto region. Microbiologia Medica. 1996;11(4):492-5.

273. Perez M, Rikihisa Y, Wen BH. Ehrlichia canis-like agent isolated from a man in Venezuela: Antigenic and genetic characterization. Journal of Clinical Microbiology. 1996;34(9):2133-9. doi: 10.1128/jcm.34.9.2133-2139.1996. PubMed PMID: WOS:A1996VD33500016.

274. Ramzan NN, Gross JB, Kolbert C, Persing DH. Hepatitis in patients with human granulocytic ehrlichiosis. Gastroenterology. 1996;110(4):A1302-A. PubMed PMID: WOS:A1996UF73705186.

275. Ratnasamy N, Everett ED, Roland WE, McDonald G, Caldwell CW. Central nervous system manifestations of human ehrlichiosis. Clinical Infectious Diseases. 1996;23(2):314-9. doi: 10.1093/clinids/23.2.314. PubMed PMID: WOS:A1996VA55400018.

276. Richter PJ, Kimsey RB, Madigan JE, Barlough JE, Dumler JS, Brooks DL. Ixodes pacificus (Acari: Ixodidae) as a vector of Ehrlichia equi (Rickettsiales: Ehrlichieae). Journal of Medical Entomology. 1996;33(1):1-5. PubMed PMID: WOS:A1996TP03900001.

277. Rosenblatt J, Sloan L, Magera J, Finkel M, Dattwyler R, Persing D. Detection of serum antibodies to the agent of human granulocytic ehrlichiosis using an indirect fluorescent antibody procedure. Clinical Infectious Diseases. 1996;23(4):195-. PubMed PMID: WOS:A1996VN24600241.

278. Santiago EB, Garcia PM. Emerging bacteria causing infectious diseases. Revista Clinica Espanola. 1996;196:59-65. PubMed PMID: WOS:A1996VN13100016.

279. Schaffner W, Standaert SM. Ehrlichiosis--in pursuit of an emerging infection. N Engl J Med. 1996;334(4):262-3. Epub 1996/01/25. doi: 10.1056/nejm199601253340410. PubMed PMID: 8532005.

280. Telford SR, 3rd, Dawson JE, Katavolos P, Warner CK, Kolbert CP, Persing DH. Perpetuation of the agent of human granulocytic ehrlichiosis in a deer tick-rodent cycle. Proc Natl Acad Sci U S A. 1996;93(12):6209-14. Epub 1996/06/11. doi: 10.1073/pnas.93.12.6209. PubMed PMID: 8650245; PubMed Central PMCID: PMCPMC39215.

281. Van Vliet AHM, Van Der Zeijst BAM, Camus E, Mahan SM, Martinez D, Jongejan F. Recombinant expression and use in serology of a specific fragment from the Cowdria ruminantium MAP1 protein. 1996. p. 35-45.

282. Waladde SM, Young AS, Morzaria SP. Artificial feeding of ixodid ticks. Parasitol Today. 1996;12(7):272-8. Epub 1996/07/01. doi: 10.1016/0169-4758(96)10027-2. PubMed PMID: 15275192.

283. Walker DH. Human ehrlichiosis: more trouble from ticks. Hosp Pract (1995). 1996;31(4):47-57. Epub 1996/04/15. PubMed PMID: 8609191.

284. Walker DH. Rickettsiae. In: Baron S, editor. Medical Microbiology. Galveston (TX): University of Texas Medical Branch at Galveston

Copyright © 1996, The University of Texas Medical Branch at Galveston.; 1996.

285. Walker DH, Barbour AG, Oliver JH, Lane RS, Dumler JS, Dennis DT, et al. Emerging bacterial zoonotic and vector-borne diseases - Ecological and epidemiological factors. Jama-Journal of the American Medical Association. 1996;275(6):463-9. doi: 10.1001/jama.275.6.463. PubMed PMID: WOS:A1996TU63800035.

286. Walker DH, Dumler JS. Emergence of the ehrlichioses as human health problems. Emerg Infect Dis. 1996;2(1):18-29. Epub 1996/01/01. doi: 10.3201/eid0201.960102. PubMed PMID: 8903194; PubMed Central PMCID: PMCPMC2639805.

287. Weinstein RS. Human ehrlichiosis. Am Fam Physician. 1996;54(6):1971-6. Epub 1996/11/01. PubMed PMID: 8900357.

288. Wilske B, Fingerle V. Ehrlichiosis, a New Tick-borne Infectious Disease. Munchener Medizinische Wochenschrift. 1996;138(12):202-4.

289. Wong S, Grady LJ. Ehrlichia infection as a cause of severe respiratory distress. N Engl J Med. 1996;334(4):273. Epub 1996/01/25. doi: 10.1056/nejm199601253340418. PubMed PMID: 8532017.

290. Wormser GP, Horowitz HW, Dumler JS, Schwartz I, Aguero-Rosenfeld M. False-positive Lyme disease serology in human granulocytic ehrlichiosis. Lancet. 1996;347(9006):981-2. Epub 1996/04/06. doi: 10.1016/s0140-6736(96)91475-0. PubMed PMID: 8598802.

291. Wyatt CR, Davis WC, Knowles DP, Goff WL, Palmer GH, McGuire TC. Effect on intraerythrocytic Anaplasma marginale of soluble factors from infected calf blood mononuclear cells. Infection and Immunity. 1996;64(11):4846-9. doi: 10.1128/iai.64.11.4846-4849.1996.

292. AgueroRosenfeld ME, Wormser GP, Dumler JS. Human granulocytic ehrlichiosis: A cardiac risk factor? Response. Annals of Internal Medicine. 1997;127(1):90-. doi: 10.7326/0003-4819-127-1-199707010-00024. PubMed PMID: WOS:A1997XG91400023.

293. Alleman AR, Palmer GH, McGuire TC, McElwain TF, Perryman LE, Barbet AF. Anaplasma marginale major surface protein 3 is encoded by a polymorphic, multigene family. Infect Immun. 1997;65(1):156-63. Epub 1997/01/01. doi: 10.1128/iai.65.1.156-163.1997. PubMed PMID: 8975906; PubMed Central PMCID: PMCPMC174570.

294. Andras S, Anna M. Infectology at the end of the 20th century - Human ehrlichiosis. Lege Artis Medicine. 1997;7(11):692-6.

295. Asanovich KM, Bakken JS, Madigan JE, Aguero-Rosenfeld M, Wormser GP, Dumler JS. Antigenic diversity of granulocytic Ehrlichia isolates from humans in Wisconsin and New York and a horse in California. J Infect Dis. 1997;176(4):1029-34. Epub 1997/10/23. doi: 10.1086/516529. PubMed PMID: 9333162.

296. Bakken JS, Dumler JS. Risk for acquiring human granulocytic ehrlichiosis: Exposure to deer blood or deer ticks? Reply. Clinical Infectious Diseases. 1997;24(3):532-3. doi: 10.1093/clinids/24.3.532. PubMed PMID: WOS:A1997WK38200054.

297. Bakken JS, Krueth J, Riddell D, Tilden RL, Asanovich K, Dumler JS. The effect of doxycycline on blood counts in patients infected with human granulocytic ehrlichiosis (HGE). Clinical Infectious Diseases. 1997;25(2):368.

298. Bakken JS, Krueth J, Tilden RL, Asanovich K, Walls J, Dumler JS. Duration of IFA urologic response in humans infected with the agent of Human Granulocytic Ehrlichiosis (HGE). Clinical Infectious Diseases. 1997;25(2):368.

299. Barlough JE, Madigan JE, Kramer VL, Clover JR, Hui LT, Webb JP, et al. Ehrlichia phagocytophila genogroup rickettsiae in ixodid ticks from California collected in 1995 and 1996. Journal of Clinical Microbiology. 1997;35(8):2018-21. doi: 10.1128/jcm.35.8.2018-2021.1997. PubMed PMID: WOS:A1997XL74500018.

300. Barlough JE, Madigan JE, Turoff DR, Clover JR, Shelly SM, Dumler S. An Ehrlichia strain from a llama (Lama glama) and llama-associated ticks (Ixodes pacificus). Journal of Clinical Microbiology. 1997;35(4):1005-7. doi: 10.1128/jcm.35.4.1005-1007.1997. PubMed PMID: WOS:A1997WP40500044.

301. Behl R, Klein MB, Dandelet LA, Bach RR, Goodman JL, Key NS. Induction of tissue factor procoagulant activity in HL-60 cells inocculated by the agent of human granulocytic ehrlichiosis. Blood. 1997;90(10):2002-. PubMed PMID: WOS:A1997YG42401998.

302. Belongia EA, Chyou PH, Reed KD. Lyme disease and human granulocytic ehrlichiosis. Am J Clin Pathol. 1997;108(4):479-80; author reply 82-3. Epub 1997/10/10. PubMed PMID: 9322603.

303. Belongia EA, Reed KD, Mitchell PD, Kolbert CP, Persing DH, Gill JS, et al. Prevalence of granulocytic Ehrlichia infection among white-tailed deer in Wisconsin. Journal of Clinical Microbiology. 1997;35(6):1465-8. doi: 10.1128/jcm.35.6.1465-1468.1997. PubMed PMID: WOS:A1997XA75500032.

304. Brouqui P. Human ehrlichiose, an emerging infectious disease. Medecine et Maladies Infectieuses. 1997;27(3):256-66.

305. Carter N, Miller NR. Fourth nerve palsy caused by Ehrlichia chaffeensis. J Neuroophthalmol. 1997;17(1):47-50. Epub 1997/03/01. PubMed PMID: 9093962.

306. Cinco M, Padovan D, Murgia R, Maroli M, Frusteri L, Heldtander M, et al. Coexistence of Ehrlichia phagocytophila and Borrelia burgdorferi sensu lato in Ixodes ricinus ticks from Italy as determined by 16S rRNA gene sequencing. J Clin Microbiol. 1997;35(12):3365-6. Epub 1997/12/17. doi: 10.1128/jcm.35.12.3365-3366.1997. PubMed PMID: 9399564; PubMed Central PMCID: PMCPMC230192.

307. Cohen MR. Tick-borne diseases in the United States. South Med J. 1997;90(6):663. Epub 1997/06/01. doi: 10.1097/00007611-199706000-00019. PubMed PMID: 9191750.

308. Daniels TJ, Falco RC, Schwartz I, Varde S, Robbins RG. Deer ticks (Ixodes scapularis) and the agents of Lyme disease and human granulocytic ehrlichiosis in a New York City park. Emerg Infect Dis. 1997;3(3):353-5. Epub 1997/07/01. doi: 10.3201/eid0303.970312. PubMed PMID: 9284380; PubMed Central PMCID: PMCPMC2627640.

309. Dawson JE, Warner CK, Ewing SA, Telford SR, Corstvet RE, Brennan R, et al. Fingerprinting of Ehrlichia species by repetitive element polymerase chain reaction. Am J Trop Med Hyg. 1997;57(1):109-14. Epub 1997/07/01. doi: 10.4269/ajtmh.1997.57.109. PubMed PMID: 9242329.

310. Des Vignes F, Fish D. Transmission of the agent of human granulocytic ehrlichiosis by host-seeking Ixodus scapularis (Acari:Ixodidae) in southern New York state. J Med Entomol. 1997;34(4):379-82. Epub 1997/07/01. doi: 10.1093/jmedent/34.4.379. PubMed PMID: 9220669.

311. Dumler JS. Is human granulocytic ehrlichiosis a new Lyme disease? Review and comparison of clinical, laboratory, epidemiological, and some biological features. Clin Infect Dis. 1997;25 Suppl 1:S43-7. Epub 1997/07/01. doi: 10.1086/516164. PubMed PMID: 9233663.

312. Dumler JS, Dotevall L, Gustafson R, Granström M. A population-based seroepidemiologic study of human granulocytic ehrlichiosis and Lyme borreliosis on the west coast of Sweden. J Infect Dis. 1997;175(3):720-2. Epub 1997/03/01. doi: 10.1093/infdis/175.3.720. PubMed PMID: 9041353.

313. Eliasson I, Bjöersdorff A. Does human ehrlichiosis exist in Sweden? New tick-borne zoonoses. Lakartidningen. 1997;(40):3487-8.

314. Ewing SA, Dawson JE, Mathew JS, Barker RW, Pratt KW, Telford SR, 3rd. Attempted transmission of human granulocytotropic Ehrlichia (HGE) by Amblyomma americanum and Amblyomma maculatum. Vet Parasitol. 1997;70(1-3):183-90. Epub 1997/06/01. doi: 10.1016/s0304-4017(96)01157-0. PubMed PMID: 9195722.

315. Ewing SA, Dawson JE, Panciera RJ, Mathew JS, Pratt KW, Katavolos P, et al. Dogs infected with a human granulocytotropic Ehrlichia spp. (Rickettsiales: Ehrlichieae). J Med Entomol. 1997;34(6):710-8. Epub 1998/01/24. doi: 10.1093/jmedent/34.6.710. PubMed PMID: 9439127.

316. Fingerle V, Goodman JL, Johnson RC, Kurtti TJ, Munderloh UG, Wilske B. Human granulocytic ehrlichiosis in southern Germany: increased seroprevalence in high-risk groups. J Clin Microbiol. 1997;35(12):3244-7. Epub 1997/12/17. doi: 10.1128/jcm.35.12.3244-3247.1997. PubMed PMID: 9399527; PubMed Central PMCID: PMCPMC230155.

317. Fritz CL, Kjemtrup AM, Conrad PA, Flores GR, Campbell GL, Schriefer ME, et al. Seroepidemiology of emerging tickborne infectious diseases in a Northern California community. J Infect Dis. 1997;175(6):1432-9. Epub 1997/06/01. doi: 10.1086/516476. PubMed PMID: 9180183.

318. Gale KR, Leatch G, Dimmock CM, Wood PR. Anaplasma marginale: Effect of the treatment of cattle with an interferon γ-neutralizing monoclonal antibody or the nitric oxide synthetase inhibitor aminoguanidine on the course of infection. Parasite Immunology. 1997;19(9):411-7. doi: 10.1046/j.1365-3024.1997.d01-237.x.

319. Gluckman SJ. Ehrlichia infections of humans. Infectious Diseases in Clinical Practice. 1997;6(2):96-100. doi: 10.1097/00019048-199702000-00005. PubMed PMID: WOS:A1997WK54100005.

320. Glushko GM. Human ehrlichiosis. Postgraduate Medicine. 1997;101(6):225-30. doi: 10.3810/pgm.1997.06.231. PubMed PMID: WOS:A1997XE56300015.

321. Goddard J. Rickettsial organisms transmitted by ticks: Ehrlichiosis. Infections in Medicine. 1997;14(3):224+9-30.

322. Heimer R, Van Andel A, Wormser GP, Wilson ML. Propagation of granulocytic Ehrlichia spp. from human and equine sources in HL-60 cells induced to differentiate into functional granulocytes. J Clin Microbiol. 1997;35(4):923-7. Epub 1997/04/01. doi: 10.1128/jcm.35.4.923-927.1997. PubMed PMID: 9157154; PubMed Central PMCID: PMCPMC229702.

323. Horowitz HW, Wormser GP. Human granulocytic ehrlichiosis. Clinical Immunology Newsletter. 1997;17(10-11):141-6. doi: 10.1016/S0197-1859(00)80018-7.

324. Hsieh TC, Aguero-Rosenfeld ME, Wu JM, Ng C, Papanikolaou NA, Varde SA, et al. Cellular changes and induction of apoptosis in human promyelocytic HL-60 cells infected with the agent of human granulocytic ehrlichiosis (HGE). Biochem Biophys Res Commun. 1997;232(2):298-303. Epub 1997/03/17. doi: 10.1006/bbrc.1997.6276. PubMed PMID: 9125168.

325. Jacobs RF, Schutze GE. Ehrlichiosis in children. Journal of Pediatrics. 1997;131(2):184-92. doi: 10.1016/s0022-3476(97)70152-5. PubMed PMID: WOS:A1997XU68000008.

326. Jorde UP, Aguero-Rosenfeld ME, Wormser GP, Dumler JS. Human granulocytic ehrlichiosis: A cardiac risk factor? [5] (multiple letters). Annals of Internal Medicine. 1997;127(1):89-90. doi: 10.7326/0003-4819-127-1-199707010-00023.

327. JW IJ, Zhang Y, Hodzic E, Magnarelli LA, Wilson ML, Telford SR, 3rd, et al. The early humoral response in human granulocytic ehrlichiosis. J Infect Dis. 1997;176(3):687-92. Epub 1997/09/18. doi: 10.1086/514091. PubMed PMID: 9291316.

328. Klein MB, Hu S, Chao CC, Goodman JL. The agent of human granulocytic ehrlichiosis (HGE) induces the production of myelosuppressing chemokines. Clinical Infectious Diseases. 1997;25(2):427.

329. Klein MB, Miller JS, Nelson CM, Goodman JL. Primary bone marrow progenitors of both granulocytic and monocytic lineages are susceptible to infection with the agent of human granulocytic ehrlichiosis. J Infect Dis. 1997;176(5):1405-9. Epub 1997/11/14. doi: 10.1086/517332. PubMed PMID: 9359749.

330. Klein MB, Nelson CM, Goodman JL. Antibiotic susceptibility of the newly cultivated agent of human granulocytic ehrlichiosis: promising activity of quinolones and rifamycins. Antimicrob Agents Chemother. 1997;41(1):76-9. Epub 1997/01/01. doi: 10.1128/aac.41.1.76. PubMed PMID: 8980758; PubMed Central PMCID: PMCPMC163663.

331. Kolbert CP, Bruinsma ES, Abdulkarim AS, Hofmeister EK, Tompkins RB, Telford SR, 3rd, et al. Characterization of an immunoreactive protein from the agent of human granulocytic ehrlichiosis. J Clin Microbiol. 1997;35(5):1172-8. Epub 1997/05/01. doi: 10.1128/jcm.35.5.1172-1178.1997. PubMed PMID: 9114402; PubMed Central PMCID: PMCPMC232724.

332. Krause PJ, Telford Iii SR. Emerging tick-borne zoonoses: Lyme disease, babesiosis, human granulocytic ehrlichiosis. Seminars in Pediatric Infectious Diseases. 1997;8(1):34-43.

333. Laudicina RJ, Hilger AE. Focus: tick-borne diseases. Human Ehrlichiosis: a case review. Clinical Laboratory Science. 1997;10(3):149-69. PubMed PMID: 107238109. Language: English. Entry Date: 19980201. Revision Date: 20150820. Publication Type: Journal Article.

334. Little SE, Dawson JE, Lockhart JM, Stallknecht DE, Warner CK, Davidson WR. Development and use of specific polymerase reaction for the detection of an organism resembling Ehrlichia sp. in white-tailed deer. Journal of Wildlife Diseases. 1997;33(2):246-53. doi: 10.7589/0090-3558-33.2.246. PubMed PMID: WOS:A1997WW77000009.

335. Magnarelli LA, Anderson JF, Stafford KC, Dumler JS. Antibodies to multiple tick-borne pathogens of babesiosis, ehrlichiosis, and Lyme borreliosis in white-footed mice. Journal of Wildlife Diseases. 1997;33(3):466-73. doi: 10.7589/0090-3558-33.3.466. PubMed PMID: WOS:A1997XM00400009.

336. Magnarelli LA, Ijdo JW, Anderson JF, Madigan JE, Dumler JS, Fikrig E. Antibodies to Ehrlichia equi in dogs from the northeastern United States. Journal of the American Veterinary Medical Association. 1997;211(9):1134-7. PubMed PMID: WOS:A1997YD80500016.

337. Mazzella FM, Kranwinkel R. Lyme disease and human granulocytic ehrlichiosis. Am J Clin Pathol. 1997;108(4):481; author reply 2-3. Epub 1997/10/10. PubMed PMID: 9322604.

338. Means RG, White DJ. New distribution records of Amblyomma americanum (L.) (Acari : Ixodidae) in New York State. Journal of Vector Ecology. 1997;22(2):133-45. PubMed PMID: WOS:000071877500007.

339. Nicholson WL, Comer JA, Sumner JW, Gingrich-Baker C, Coughlin RT, Magnarelli LA, et al. An indirect immunofluorescence assay using a cell culture-derived antigen for detection of antibodies to the agent of human granulocytic ehrlichiosis. J Clin Microbiol. 1997;35(6):1510-6. Epub 1997/06/01. doi: 10.1128/jcm.35.6.1510-1516.1997. PubMed PMID: 9163471; PubMed Central PMCID: PMCPMC229776.

340. Persing DH. The cold zone: a curious convergence of tick-transmitted diseases. Clin Infect Dis. 1997;25 Suppl 1:S35-42. Epub 1997/07/01. doi: 10.1086/516170. PubMed PMID: 9233662.

341. Pusterla N, Huder J, Wolfensberger C, Litschi B, Parvis A, Lutz H. Granulocytic ehrlichiosis in two dogs in Switzerland. J Clin Microbiol. 1997;35(9):2307-9. Epub 1997/09/01. doi: 10.1128/jcm.35.9.2307-2309.1997. PubMed PMID: 9276407; PubMed Central PMCID: PMCPMC229959.

342. Pusterla N, Wolfensberger C, GerberBretscher R, Lutz H. Comparison of indirect immunofluorescence for Ehrlichia phagocytophila and Ehrlichia equi in horses. Equine Veterinary Journal. 1997;29(6):490-2. doi: 10.1111/j.2042-3306.1997.tb03165.x. PubMed PMID: WOS:A1997YH60400017.

343. Ribeiro MFB, Passos LMF, Guimarães AM. Ultrastructure of Anaplasma marginale with an inclusion appendage, isolated in Minas Gerais State, Brazil. Veterinary Parasitology. 1997;70(4):271-7. doi: 10.1016/S0304-4017(97)00004-6.

344. Rikihisa Y, Zhi N, Wormser GP, Wen B, Horowitz HW, Hechemy KE. Ultrastructural and antigenic characterization of a granulocytic ehrlichiosis agent directly isolated and stably cultivated from a patient in New York state. J Infect Dis. 1997;175(1):210-3. Epub 1997/01/01. doi: 10.1093/infdis/175.1.210. PubMed PMID: 8985223.

345. Roberts R, Soave R. Emerging Pathogens Associated with Tick-Borne Infections. Braz J Infect Dis. 1997;1(1):17-26. Epub 1997/03/01. PubMed PMID: 11107234.

346. Schwartz I, Fish D, Daniels TJ. Prevalence of the rickettsial agent of human granulocytic ehrlichiosis in ticks from a hyperendemic focus of Lyme disease. New England Journal of Medicine. 1997;337(1):49-50. doi: 10.1056/nejm199707033370111. PubMed PMID: WOS:A1997XH18600011.

347. Sood SK, Stephen Dumler J. Human Ehrlichia equi/phagocytophila (EEP) infection on long Island (LI). Clinical Infectious Diseases. 1997;25(2):480.

348. Sumner JW, Nicholson WL, Massung RF. PCR amplification and comparison of nucleotide sequences from the groESL heat shock operon of Ehrlichia species. J Clin Microbiol. 1997;35(8):2087-92. Epub 1997/08/01. doi: 10.1128/jcm.35.8.2087-2092.1997. PubMed PMID: 9230387; PubMed Central PMCID: PMCPMC229908.

349. Sun W, JW IJ, Telford SR, 3rd, Hodzic E, Zhang Y, Barthold SW, et al. Immunization against the agent of human granulocytic ehrlichiosis in a murine model. J Clin Invest. 1997;100(12):3014-8. Epub 1998/01/31 20:29. doi: 10.1172/jci119855. PubMed PMID: 9399947; PubMed Central PMCID: PMCPMC508513.

350. Telford SR, 3rd. Risk for acquiring human granulocytic ehrlichiosis: exposure to deer blood or deer ticks? Clin Infect Dis. 1997;24(3):531-3. Epub 1997/03/01. doi: 10.1093/clinids/24.3.531. PubMed PMID: 9114225.

351. Telford SR, Armstrong PM, Katavolos P, Foppa I, Garcia ASO, Wilson ML, et al. A new tick-borne encephalitis-like virus infecting New England deer ticks, Ixodes dammini. Emerging Infectious Diseases. 1997;3(2):165-70. doi: 10.3201/eid0302.970209. PubMed PMID: WOS:A1997XL15500009.

352. Trotta RF, Hospenthal DR, Bennett SP, Daniels AM, Fishbain JT. Human monocytic ehrlichiosis with concurrent Lyme antibody seroconversion. Infectious Diseases in Clinical Practice. 1997;6(6):401-5. doi: 10.1097/00019048-199706060-00010. PubMed PMID: WOS:A1997XX24000016.

353. von Stedingk LV, Gürtelschmid M, Hanson HS, Gustafson R, Dotevall L, Engvall EO, et al. The human granulocytic ehrlichiosis (HGE) agent in Swedish ticks. Clin Microbiol Infect. 1997;3(5):573-4. Epub 1997/01/01. doi: 10.1111/j.1469-0691.1997.tb00311.x. PubMed PMID: 11864185.

354. Walker DH, Dumler JS. Human monocytic and granulocytic ehrlichioses - Discovery and diagnosis of emerging tick-borne infections and the critical role of the pathologist. Archives of Pathology & Laboratory Medicine. 1997;121(8):785-91. PubMed PMID: WOS:A1997XR79100003.

355. Walls JJ, Greig B, Neitzel DF, Dumler JS. Natural infection of small mammal species in Minnesota with the agent of human granulocytic ehrlichiosis. J Clin Microbiol. 1997;35(4):853-5. Epub 1997/04/01. doi: 10.1128/jcm.35.4.853-855.1997. PubMed PMID: 9157141; PubMed Central PMCID: PMCPMC229689.

356. Wormser GP, Horowitz HW, Nowakowski J, McKenna D, Schwartz I, AgueroRosenfeld M, et al. Lyme disease and human granulocytic ehrlichiosis - Reply. American Journal of Clinical Pathology. 1997;108(4):482-3. PubMed PMID: WOS:A1997XX43400019.

357. Yeh MT, Mather TN, Coughlin RT, Gingrich-Baker C, Sumner JW, Massung RF. Serologic and molecular detection of granulocytic ehrlichiosis in Rhode Island. J Clin Microbiol. 1997;35(4):944-7. Epub 1997/04/01. doi: 10.1128/jcm.35.4.944-947.1997. PubMed PMID: 9157157; PubMed Central PMCID: PMCPMC229705.

358. Zhi N, Rikihisa Y, Kim HY, Wormser GP, Horowitz HW. Comparison of major antigenic proteins of six strains of the human granulocytic ehrlichiosis agent by Western immunoblot analysis. J Clin Microbiol. 1997;35(10):2606-11. Epub 1997/10/08. doi: 10.1128/jcm.35.10.2606-2611.1997. PubMed PMID: 9316916; PubMed Central PMCID: PMCPMC230019.

359. Statewide surveillance for ehrlichiosis--Connecticut and New York, 1994-1997. MMWR Morb Mortal Wkly Rep. 1998;47(23):476-80. Epub 1998/07/02. PubMed PMID: 9649234.

360. Alberdi MP, Walker AR, Paxton EA, Sumption KJ. Natural prevalence of infection with Ehrlichia (Cytoecetes) phagocytophila of Ixodes ricinus ticks in Scotland. Veterinary Parasitology. 1998;78(3):203-13. doi: 10.1016/s0304-4017(98)00138-1. PubMed PMID: WOS:000075837800005.

361. Bakken JS. The discovery of human granulocytotropic ehrlichiosis. J Lab Clin Med. 1998;132(3):175-80. Epub 1998/09/15. doi: 10.1016/s0022-2143(98)90165-2. PubMed PMID: 9735922.

362. Bakken JS, Dumler JS, Kristiansen BE. [A new tick-borne disease--human granulocytic ehrlichiosis]. Tidsskr Nor Laegeforen. 1998;118(26):4117-8. Epub 1998/12/09. PubMed PMID: 9844520.

363. Bakken JS, Goellner P, Van Etten M, Boyle DZ, Swonger OL, Mattson S, et al. Seroprevalence of human granulocytic ehrlichiosis among permanent residents of northwestern Wisconsin. Clin Infect Dis. 1998;27(6):1491-6. Epub 1998/12/30. doi: 10.1086/515048. PubMed PMID: 9868666.

364. Barbour AG. Fall and rise of Lyme disease and other Ixodes tick-borne infections in North America and Europe. British Medical Bulletin. 1998;54(3):647-58. PubMed PMID: WOS:000080271300012.

365. Bedner E, Burfeind P, Hsieh TC, Wu JM, Aguero-Rosenfeld ME, Melamed MR, et al. Cell cycle effects and induction of apoptosis caused by infection of HL-60 cells with human granulocytic ehrlichiosis pathogen measured by flow and laser scanning cytometry. Cytometry. 1998;33(1):47-55. Epub 1998/09/02. PubMed PMID: 9725558.

366. Blandino T, Camacho M, Barrera M, Alonso M, Gallardo J, Martinez S. Characterization of an immunogen of Anaplasma marginale. 1998. p. 416-9.

367. Brizuela CM, Ortellado CA, Sanabria E, Torres O, Ortigosa D. The safety and efficacy of Australian tick-borne disease vaccine strains in cattle in Paraguay. Veterinary Parasitology. 1998;76(1-2):27-41. doi: 10.1016/S0304-4017(97)00047-2.

368. Brouqui P. Human ehrlichiose, an emerging infectious disease. Medecine et Maladies Infectieuses. 1998;28(4 SPECIAL MAY):349-53. doi: 10.1016/s0399-077x(98)70220-3.

369. Bunnell JE, Dumler JS, Childs JE, Glass GE. Retrospective serosurvey for human granulocytic ehrlichiosis agent in urban white-footed mice from Maryland. J Wildl Dis. 1998;34(1):179-81. Epub 1998/02/26. doi: 10.7589/0090-3558-34.1.179. PubMed PMID: 9476244.

370. Chang YF, Novosel V, Chang CF, Kim JB, Shin SJ, Lein DH. Detection of human granulocytic ehrlichiosis agent and Borrelia burgdorferi in ticks by polymerase chain reaction. J Vet Diagn Invest. 1998;10(1):56-9. Epub 1998/04/04. doi: 10.1177/104063879801000110. PubMed PMID: 9526861.

371. Chang YF, Novosel V, Dubovi E, Wong SJ, Chu FK, Chang CF, et al. Experimental infection of the human granulocytic ehrlichiosis agent in horses. Vet Parasitol. 1998;78(2):137-45. Epub 1998/09/15. doi: 10.1016/s0304-4017(98)00133-2. PubMed PMID: 9735918.

372. Chu FK. Rapid and sensitive PCR-based detection and differentiation of aetiologic agents of human granulocytotropic and monocytotropic ehrlichiosis. Mol Cell Probes. 1998;12(2):93-9. Epub 1998/06/20. doi: 10.1006/mcpr.1998.0150. PubMed PMID: 9633044.

373. Cinco M, Padovan D, Murgia R, Heldtander M, Engvall EO. Detection of HGE agent-like Ehrlichia in Ixodes ricinus ticks in northern Italy by PCR. Wien Klin Wochenschr. 1998;110(24):898-900. Epub 1999/02/27. PubMed PMID: 10048173.

374. Corcaci DC. [An update on human ehrlichiosis]. Rev Med Chir Soc Med Nat Iasi. 1998;102(3-4):65-8. Epub 2000/04/11. PubMed PMID: 10756846.

375. Daniels TJ, Boccia TM, Varde S, Marcus J, Le J, Bucher DJ, et al. Geographic risk for lyme disease and human granulocytic ehrlichiosis in southern New York state. Appl Environ Microbiol. 1998;64(12):4663-9. Epub 1998/12/03. doi: 10.1128/aem.64.12.4663-4669.1998. PubMed PMID: 9835546; PubMed Central PMCID: PMCPMC90906.

376. Das S, Deponte K, Marcantonio NL, Ijdo JW, Hodzic E, Katavolos P, et al. Granulocytic ehrlichiosis in tick-immune guinea pigs. Infect Immun. 1998;66(4):1803-5. Epub 1998/04/07. doi: 10.1128/iai.66.4.1803-1805.1998. PubMed PMID: 9529119; PubMed Central PMCID: PMCPMC108126.

377. Dumler JS. Ehrlichioses: emerging infections. Curr Opin Infect Dis. 1998;11(2):183-7. Epub 2006/10/13. PubMed PMID: 17033387.

378. Dumler JS, Bakken JS. Human ehrlichioses: newly recognized infections transmitted by ticks. Annu Rev Med. 1998;49:201-13. Epub 1998/03/24. doi: 10.1146/annurev.med.49.1.201. PubMed PMID: 9509259.

379. Dumler JS, Christova I. CD15 monoclonal antibodies and heparin sulfate abrogate human granulocytic ehrlichiosis (HGE) agent infection of HL60 cells. Faseb Journal. 1998;12(5):A807-A. PubMed PMID: WOS:000076006501063.

380. Duval X, Chosidow O, Tissot-Dupont H, Raoult D, Frances C. Cutaneous manifestations of rickettsiosis. Revue De Medecine Interne. 1998;19(8):548-57. doi: 10.1016/s0248-8663(99)80022-8. PubMed PMID: WOS:000075834500004.

381. Egenvall A, Bjoersdorff A, Lilliehook I, Engvall EO, Karlstam E, Artursson K, et al. Early manifestations of granulocytic ehrlichiosis in dogs inoculated experimentally with a Swedish Ehrlichia species isolate. Veterinary Record. 1998;143(15):412-7. doi: 10.1136/vr.143.15.412. PubMed PMID: WOS:000076651800006.

382. Elston DM, Edlow JA, Horowitz H, Kilchevsky E. Perinatal transmission of human granulocytic ehrlichiosis [2] (multiple letters). New England Journal of Medicine. 1998;339(26):1941-3. doi: 10.1056/NEJM199812243392615.

383. Evans J. Lyme disease. Curr Opin Rheumatol. 1998;10(4):339-46. Epub 1998/09/02. doi: 10.1097/00002281-199807000-00011. PubMed PMID: 9725096.

384. Fitzpatrick JL, Barron RCJ, Andrew L, Thompson H. Eperythrozoon ovis infection of sheep. Comparative Haematology International. 1998;8(4):230-4. doi: 10.1007/BF02752854.

385. Foley JE, Barlough JE, Kimsey RB, Madigan JE, DeRock E, Poland A. Ehrlichia spp. in cervids from California. J Wildl Dis. 1998;34(4):731-7. Epub 1998/11/14. doi: 10.7589/0090-3558-34.4.731. PubMed PMID: 9813842.

386. Fordham LA, Chung CJ, Specter BB, Merten DF, Ingram DL. Ehrlichiosis: Findings on chest radiographs in three pediatric patients. American Journal of Roentgenology. 1998;171(5):1421-4. doi: 10.2214/ajr.171.5.9798890. PubMed PMID: WOS:000076580400049.

387. French DM, McElwain TF, McGuire TC, Palmer GH. Erratum: Expression of Anaplasma marginale major surface protein 2 variants during persistent cyclic rickettsemia (Infection and Immunity 66:3 (1202)). Infection and Immunity. 1998;66(5):2400. doi: 10.1128/iai.66.5.2400-2400.1998.

388. Fritz CL, Glaser CA. Ehrlichiosis. Infectious Disease Clinics of North America. 1998;12(1):123-+. doi: 10.1016/s0891-5520(05)70413-x. PubMed PMID: WOS:000072123000011.

389. Frohlich W, Edelhofer R. The first case of equine granulocytic ehrlichiosis (EGE) in an Austrian horse. Wiener Tierarztliche Monatsschrift. 1998;85(11):389-94. PubMed PMID: WOS:000077106300004.

390. Goldman EE, Breitschwerdt EB, Grindem CB, Hegarty BC, Walls JJ, Dumler JS. Granulocytic ehrlichiosis in dogs from North Carolina and Virginia. J Vet Intern Med. 1998;12(2):61-70. Epub 1998/04/30. doi: 10.1111/j.1939-1676.1998.tb02096.x. PubMed PMID: 9560760.

391. Guy E, Tasker S, Joynson DH. Detection of the agent of human granulocytic ehrlichiosis (HGE) in UK ticks using polymerase chain reaction. Epidemiol Infect. 1998;121(3):681-3. Epub 1999/02/25. doi: 10.1017/s0950268898001708. PubMed PMID: 10030718; PubMed Central PMCID: PMCPMC2809576.

392. Heimer R, Tisdale D, Dawson JE. A single tissue culture system for the propagation of the agents of the human ehrlichioses. Am J Trop Med Hyg. 1998;58(6):812-5. Epub 1998/07/11. doi: 10.4269/ajtmh.1998.58.812. PubMed PMID: 9660470.

393. Hodzic E, Fish D, Maretzki CM, De Silva AM, Feng S, Barthold SW. Acquisition and transmission of the agent of human granulocytic ehrlichiosis by Ixodes scapularis ticks. J Clin Microbiol. 1998;36(12):3574-8. Epub 1998/11/18. doi: 10.1128/jcm.36.12.3574-3578.1998. PubMed PMID: 9817875; PubMed Central PMCID: PMCPMC105242.

394. Hodzic E, Ijdo JW, Feng S, Katavolos P, Sun W, Maretzki CH, et al. Granulocytic ehrlichiosis in the laboratory mouse. J Infect Dis. 1998;177(3):737-45. Epub 1998/03/14. doi: 10.1086/514236. PubMed PMID: 9498456.

395. Hofmeister EK, Kolbert CP, Abdulkarim AS, Magera JMH, Hopkins MK, Uhl JR, et al. Cosegregation of a novel Bartonella species with Borrelia burgdorferi and Babesia microti in Peromyscus leucopus. Journal of Infectious Diseases. 1998;177(2):409-16. doi: 10.1086/514201. PubMed PMID: WOS:000071801500019.

396. Horowitz H, Kilchevsky E. Perinatal transmission of human granulocytic ehrlichiosis - Reply. New England Journal of Medicine. 1998;339(26):1942-3. PubMed PMID: WOS:000077696600020.

397. Horowitz HW, Wormser GP. Doxycycline revisited: An old medicine for emerging diseases. Archives of Internal Medicine. 1998;158(2):192-3. doi: 10.1001/archinte.158.2.192-a. PubMed PMID: WOS:000071556300014.

398. Hsieh TC, DiPietrantonio A, Kumor A, Horowitz HW, Wormser GP. Attenuated responses to interferon (IFN) in HL-60 leukemia cells infected with the agent of Human Granulocytic Ehrlichiosis (HGE). FASEB Journal. 1998;12(5).

399. Hunfeld KP, Allwinn R, Peters S, Kraiczy P, Brade V. Serologic evidence for tick-borne pathogens other than Borrelia burgdorferi (TOBB) in Lyme borreliosis patients from midwestern Germany. Wien Klin Wochenschr. 1998;110(24):901-8. Epub 1999/02/27. PubMed PMID: 10048174.

400. Ijdo JW, Fikrig E. [Human granulocytic ehrlichiosis, a tick-borne disease]. Ned Tijdschr Geneeskd. 1998;142(31):1778-81. Epub 1998/12/18. PubMed PMID: 9856144.

401. Ijdo JW, Sun W, Zhang Y, Magnarelli LA, Fikrig E. Cloning of the gene encoding the 44-kilodalton antigen of the agent of human granulocytic ehrlichiosis and characterization of the humoral response. Infect Immun. 1998;66(7):3264-9. Epub 1998/06/25. doi: 10.1128/iai.66.7.3264-3269.1998. PubMed PMID: 9632594; PubMed Central PMCID: PMCPMC108341.

402. Ijdo JW, Zhang Y, Anderson ML, Goldberg D, Fikrig E. Heat shock protein 70 of the agent of human granulocytic ehrlichiosis binds to Borrelia burgdorferi antibodies. Clin Diagn Lab Immunol. 1998;5(1):118-20. Epub 1998/02/10. doi: 10.1128/cdli.5.1.118-120.1998. PubMed PMID: 9455892; PubMed Central PMCID: PMCPMC121403.

403. Ijdoen JW. Humane granulocytaire ehrlichiose, een ziekte overgebracht door teken. Nederlands Tijdschrift voor Geneeskunde. 1998;142(31):1778-81.

404. Johnson EM, Ewing SA, Barker RW, Fox JC, Crow DW, Kocan KM. Experimental transmission of Ehrlichia canis (Rickettsiales : Ehrlichieae) by Dermacentor variabilis (Acari : Ixodidae). Veterinary Parasitology. 1998;74(2-4):277-88. doi: 10.1016/s0304-4017(97)00073-3. PubMed PMID: WOS:000072729500017.

405. Katavolos P, Armstrong PM, Dawson JE, Telford SR, 3rd. Duration of tick attachment required for transmission of granulocytic ehrlichiosis. J Infect Dis. 1998;177(5):1422-5. Epub 1998/05/21. doi: 10.1086/517829. PubMed PMID: 9593039.

406. Kim HY, Rikihisa Y. Characterization of monoclonal antibodies to the 44-kilodalton major outer membrane protein of the human granulocytic ehrlichiosis agent. J Clin Microbiol. 1998;36(11):3278-84. Epub 1998/10/17. doi: 10.1128/jcm.36.11.3278-3284.1998. PubMed PMID: 9774579; PubMed Central PMCID: PMCPMC105315.

407. Klein MB, Hayes SF, Goodman JL. Monocytic differentiation inhibits infection and granulocytic differentiation potentiates infection by the agent of human granulocytic ehrlichiosis. Infect Immun. 1998;66(7):3410-5. Epub 1998/06/25. doi: 10.1128/iai.66.7.3410-3415.1998. PubMed PMID: 9632613; PubMed Central PMCID: PMCPMC108360.

408. Lebech AM, Hansen K, Pancholi P, Sloan LM, Magera JM, Persing DH. Immunoserologic evidence of Human Granulocytic Ehrlichiosis in Danish patients with Lyme neuroborreliosis. Scand J Infect Dis. 1998;30(2):173-6. Epub 1998/09/08. doi: 10.1080/003655498750003582. PubMed PMID: 9730306.

409. Lillieh, oulm, oulm, k I, Egenvall A, Tvedten HW. Hematopathology in dogs experimentally infected with a Swedish granulocytic Ehrlichia species. Vet Clin Pathol. 1998;27(4):116-22. Epub 2002/06/21. doi: 10.1111/j.1939-165x.1998.tb01030.x. PubMed PMID: 12075539.

410. Little SE, Stallknecht DE, Lockhart JM, Dawson JE, Davidson WR. Natural coinfection of a white-tailed deer (Odocoileus virginianus) population with three Ehrlichia spp. Journal of Parasitology. 1998;84(5):897-901. doi: 10.2307/3284616. PubMed PMID: WOS:000076517900005.

411. Lockhart JM, Davidson WR, Stallknecht DE, Dawson JE. Lack of seroreactivity to Ehrlichia chaffeensis among rodent populations. Journal of Wildlife Diseases. 1998;34(2):392-6. doi: 10.7589/0090-3558-34.2.392. PubMed PMID: WOS:000073228900026.

412. Magnarelli LA, Ijdo JW, Anderson JF, Padula SJ, Flavell RA, Fikrig E. Human exposure to a granulocytic Ehrlichia and other tick-borne agents in Connecticut. J Clin Microbiol. 1998;36(10):2823-7. Epub 1998/09/17. doi: 10.1128/jcm.36.10.2823-2827.1998. PubMed PMID: 9738027; PubMed Central PMCID: PMCPMC105071.

413. Magnarelli LA, JW IJ, Dumler JS, Heimer R, Fikrig E. Reactivity of human sera to different strains of granulocytic ehrlichiae in immunodiagnostic assays. J Infect Dis. 1998;178(6):1835-8. Epub 1998/11/17. doi: 10.1086/314516. PubMed PMID: 9815246.

414. Massung RF, Slater K, Owens JH, Nicholson WL, Mather TN, Solberg VB, et al. Nested PCR assay for detection of granulocytic ehrlichiae. J Clin Microbiol. 1998;36(4):1090-5. Epub 1998/05/23. doi: 10.1128/jcm.36.4.1090-1095.1998. PubMed PMID: 9542943; PubMed Central PMCID: PMCPMC104695.

415. Meinkoth JH, Ewing SA, Cowell RL, Dawson JE, Warner CK, Mathew JS, et al. Morphologic and molecular evidence of a dual species ehrlichial infection in a dog presenting with inflammatory central nervous system disease. Journal of Veterinary Internal Medicine. 1998;12(5):389-93. doi: 10.1111/j.1939-1676.1998.tb02140.x. PubMed PMID: WOS:000075931300012.

416. Melendez RD. Review on epidemiological factors involved in the relationship among Boophilus microplus -Bovine -Babesia spp. Revista Cientifica-Facultad De Ciencias Veterinarias. 1998;8(1):25-34. PubMed PMID: WOS:000072661900005.

417. Munodzana D, McElwain TF, Knowles DP, Palmer GH. Conformational dependence of Anaplasma marginale major surface protein 5 surface-exposed B-cell epitopes. Infect Immun. 1998;66(6):2619-24. Epub 1998/05/29. doi: 10.1128/iai.66.6.2619-2624.1998. PubMed PMID: 9596725; PubMed Central PMCID: PMCPMC108247.

418. Murphy CI, Storey JR, Recchia J, Doros-Richert LA, Gingrich-Baker C, Munroe K, et al. Major antigenic proteins of the agent of human granulocytic ehrlichiosis are encoded by members of a multigene family. Infect Immun. 1998;66(8):3711-8. Epub 1998/07/23. doi: 10.1128/iai.66.8.3711-3718.1998. PubMed PMID: 9673253; PubMed Central PMCID: PMCPMC108406.

419. Murphy GL, Ewing SA, Whitworth LC, Fox JC, Kocan AA. A molecular and serologic survey of Ehrlichia canis, E-chaffeensis, and E-ewingii in dogs and ticks from Oklahoma. Veterinary Parasitology. 1998;79(4):325-39. doi: 10.1016/s0304-4017(98)00179-4. PubMed PMID: WOS:000076995800006.

420. Nicholson WL, Muir S, Sumner JW, Childs JE. Serologic evidence of infection with Ehrlichia spp. in wild rodents (Muridae: Sigmodontinae) in the United States. J Clin Microbiol. 1998;36(3):695-700. Epub 1998/03/21. doi: 10.1128/jcm.36.3.695-700.1998. PubMed PMID: 9508298; PubMed Central PMCID: PMCPMC104611.

421. Noden BH, Radulovic S, Higgins JA, Azad AF. Molecular identification of Rickettsia typhi and R-felis in co-infected Ctenocephalides felis (Siphonaptera : Pulicidae). Journal of Medical Entomology. 1998;35(4):410-4. doi: 10.1093/jmedent/35.4.410. PubMed PMID: WOS:000075274000010.

422. Nuti M, Serafini DA, Bassetti D, Ghionni A, Russino F, Rombolà P, et al. Ehrlichia infection in Italy. Emerg Infect Dis. 1998;4(4):663-5. Epub 1998/12/29. doi: 10.3201/eid0404.980420. PubMed PMID: 9866746; PubMed Central PMCID: PMCPMC2640256.

423. Ogden NH, Bown K, Horrocks BK, Woldehiwet Z, Bennett M. Granulocytic Ehrlichia infection in ixodid ticks and mammals in woodlands and uplands of the U.K. Med Vet Entomol. 1998;12(4):423-9. Epub 1998/11/24. doi: 10.1046/j.1365-2915.1998.00133.x. PubMed PMID: 9824827.

424. Ogden NH, Woldehiwet Z, Hart CA. Granulocytic ehrlichiosis: an emerging or rediscovered tick-borne disease? J Med Microbiol. 1998;47(6):475-82. Epub 1999/01/08. doi: 10.1099/00222615-47-6-475. PubMed PMID: 9879965.

425. Ohashi N, Unver A, Zhi N, Rikihisa Y. Cloning and characterization of multigenes encoding the immunodominant 30-kilodalton major outer membrane proteins of Ehrlichia canis and application of the recombinant protein for serodiagnosis. J Clin Microbiol. 1998;36(9):2671-80. Epub 1998/08/15. doi: 10.1128/jcm.36.9.2671-2680.1998. PubMed PMID: 9705412; PubMed Central PMCID: PMCPMC105182.

426. Ohashi N, Zhi N, Zhang Y, Rikihisa Y. Immunodominant major outer membrane proteins of Ehrlichia chaffeensis are encoded by a polymorphic multigene family. Infect Immun. 1998;66(1):132-9. Epub 1998/01/10. doi: 10.1128/iai.66.1.132-139.1998. PubMed PMID: 9423849; PubMed Central PMCID: PMCPMC107868.

427. Parola P, Beati L, Cambon M, Brouqui P, Raoult D. Ehrlichial DNA amplified from Ixodes ricinus (Acari: Ixodidae) in France. J Med Entomol. 1998;35(2):180-3. Epub 1998/04/16. doi: 10.1093/jmedent/35.2.180. PubMed PMID: 9538582.

428. Popov VL, Han VC, Chen SM, Dumler JS, Feng HM, Andreadis TG, et al. Ultrastructural differentiation of the genogroups in the genus Ehrlichia. J Med Microbiol. 1998;47(3):235-51. Epub 1998/03/25. doi: 10.1099/00222615-47-3-235. PubMed PMID: 9511829.

429. Pusterla N, Huder JB, Feige K, Lutz H. Identification of a granulocytic Ehrlichia strain isolated from a horse in Switzerland and comparison with other rickettsiae of the Ehrlichia phagocytophila genogroup. J Clin Microbiol. 1998;36(7):2035-7. Epub 1998/07/03. doi: 10.1128/jcm.36.7.2035-2037.1998. PubMed PMID: 9650957; PubMed Central PMCID: PMCPMC104973.

430. Pusterla N, Huder JB, Lutz H, Braun U. Detection of Ehrlichia phagocytophila DNA in Ixodes ricinus ticks from areas in Switzerland where tick-borne fever is endemic. Journal of Clinical Microbiology. 1998;36(9):2735-6. doi: 10.1128/jcm.36.9.2735-2736.1998. PubMed PMID: WOS:000075420800064.

431. Pusterla N, Weber R, Wolfensberger C, Schär G, Zbinden R, Fierz W, et al. Serological evidence of human granulocytic ehrlichiosis in Switzerland. Eur J Clin Microbiol Infect Dis. 1998;17(3):207-9. Epub 1998/07/17. doi: 10.1007/bf01691120. PubMed PMID: 9665305.

432. Radetsky M. The emerging spectrum of tickborne infections. Current Opinion in Infectious Diseases. 1998;11(3):313-8. doi: 10.1097/00001432-199806000-00008. PubMed PMID: WOS:000073822700010.

433. Ravyn MD, Goodman JL, Kodner CB, Westad DK, Coleman LA, Engstrom SM, et al. Immunodiagnosis of human granulocytic ehrlichiosis by using culture-derived human isolates. J Clin Microbiol. 1998;36(6):1480-8. Epub 1998/06/10. doi: 10.1128/jcm.36.6.1480-1488.1998. PubMed PMID: 9620365; PubMed Central PMCID: PMCPMC104863.

434. Reubel GH, Kimsey RB, Barlough JE, Madigan JE. Experimental transmission of Ehrlichia equi to horses through naturally infected ticks (Ixodes pacificus) from northern California. Journal of Clinical Microbiology. 1998;36(7):2131-4. doi: 10.1128/jcm.36.7.2131-2134.1998. PubMed PMID: WOS:000074155400064.

435. Schauber EM, Gertz SJ, Maple WT, Ostfeld RS. Coinfection of blacklegged ticks (Acari: Ixodidae) in Dutchess County, New York, with the agents of Lyme disease and human granulocytic ehrlichiosis. J Med Entomol. 1998;35(5):901-3. Epub 1998/10/17. doi: 10.1093/jmedent/35.5.901. PubMed PMID: 9775627.

436. Sexton DJ, Corey GR, Carpenter C, Kong LQ, Gandhi T, Breitschwerdt E, et al. Dual infection with Ehrlichia chaffeensis and a spotted fever group rickettsia: a case report. Emerg Infect Dis. 1998;4(2):311-6. Epub 1998/06/11. doi: 10.3201/eid0402.980222. PubMed PMID: 9621205; PubMed Central PMCID: PMCPMC2640120.

437. Sigal LH. Musculoskeletal manifestations of Lyme arthritis. Rheumatic Disease Clinics of North America. 1998;24(2):323-+. doi: 10.1016/s0889-857x(05)70012-0. PubMed PMID: WOS:000073676700009.

438. Stafford KC, Ward JS, Magnarelli LA. Impact of controlled burns on the abundance of Ixodes scapularis (Acari : Ixodidae). Journal of Medical Entomology. 1998;35(4):510-3. doi: 10.1093/jmedent/35.4.510. PubMed PMID: WOS:000075274000027.

439. Standaert SM, Clough LA, Schaffner W, Adams JS, Neuzil KM. Neurologic manifestations of human monocytic ehrlichiosis. Infectious Diseases in Clinical Practice. 1998;7(7):358-62. doi: 10.1097/00019048-199809003-00014. PubMed PMID: WOS:000075715500014.

440. Storey JR, Doros-Richert LA, Gingrich-Baker C, Munroe K, Mather TN, Coughlin RT, et al. Molecular cloning and sequencing of three granulocytic Ehrlichia genes encoding high-molecular-weight immunoreactive proteins. Infect Immun. 1998;66(4):1356-63. Epub 1998/04/07. doi: 10.1128/iai.66.4.1356-1363.1998. PubMed PMID: 9529053; PubMed Central PMCID: PMCPMC108060.

441. Stuen S, Artursson K, Olsson Engvall E. Experimental infection of lambs with an equine granulocytic Ehrlichia species resembling the agent that causes human granulocytic ehrlichiosis (HGE). Acta Vet Scand. 1998;39(4):491-7. Epub 1999/02/02. doi: 10.1186/bf03547775. PubMed PMID: 9926463; PubMed Central PMCID: PMCPMC8050659.

442. Stuen S, Engvall EO, Artursson K. Persistence of Ehrlichia phagocytophila infection in lambs in relation to clinical parameters and antibody responses. Veterinary Record. 1998;143(20):553-5. doi: 10.1136/vr.143.20.553. PubMed PMID: WOS:000077197200009.

443. Sweeney CJ, Ghassemi M, Agger WA, Persing DH. Coinfection with Babesia microti and Borrelia burgdorferi in a western Wisconsin resident. Mayo Clinic Proceedings. 1998;73(4):338-41. PubMed PMID: WOS:000072992800006.

444. Thomas DR, Sillis M, Coleman TJ, Kench SM, Ogden NH, Salmon RL, et al. Low rates of ehrlichiosis and Lyme borreliosis in English farmworkers. Epidemiol Infect. 1998;121(3):609-14. Epub 1999/02/25. doi: 10.1017/s0950268898001514. PubMed PMID: 10030710; PubMed Central PMCID: PMCPMC2809568.

445. Torioni De Echaide S, Knowles DP, McGuire TC, Palmer GH, Suarez CE, McElwain TF. Detection of cattle naturally infected with Anaplasma marginale in a region of endemicity by nested PCR and a competitive enzyme-linked immunosorbent assay using recombinant major surface protein 5. Journal of Clinical Microbiology. 1998;36(3):777-82. doi: 10.1128/jcm.36.3.777-782.1998.

446. Van Andel AE, Magnarelli LA, Heimer R, Wilson ML. Development and duration of antibody response against Ehrlichia equi in horses. Journal of the American Veterinary Medical Association. 1998;212(12):1910-+. PubMed PMID: WOS:000074230000029.

447. Varde S, Beckley J, Schwartz I. Prevalence of tick-borne pathogens in Ixodes scapularis in a rural New Jersey County. Emerg Infect Dis. 1998;4(1):97-9. Epub 1998/03/21. doi: 10.3201/eid0401.980113. PubMed PMID: 9452402; PubMed Central PMCID: PMCPMC2627663.

448. Vidotto O, Barbosa GS, Andrade GM, Machado RZ, Da Rocha MA, Silva SS. Evaluation of a frozen trivalent attenuated vaccine against Babesiosis and Anaplasmosis in Brazil. 1998. p. 420-3.

449. Walker DH. Tick-transmitted infectious diseases in the United States. Annu Rev Public Health. 1998;19:237-69. Epub 1998/06/05. doi: 10.1146/annurev.publhealth.19.1.237. PubMed PMID: 9611619.

450. Wallace BJ, Brady G, Ackman DM, Wong SJ, Jacquette G, Lloyd EE, et al. Human granulocytic ehrlichiosis in New York. Arch Intern Med. 1998;158(7):769-73. Epub 1998/04/29. doi: 10.1001/archinte.158.7.769. PubMed PMID: 9554683.

451. Walls JJ, Asanovich KM, Bakken JS, Dumler JS. Serologic evidence of a natural infection of white-tailed deer with the agent of human granulocytic ehrlichiosis in Wisconsin and Maryland. Clin Diagn Lab Immunol. 1998;5(6):762-5. Epub 1998/11/05. doi: 10.1128/cdli.5.6.762-765.1998. PubMed PMID: 9801331; PubMed Central PMCID: PMCPMC96198.

452. Waner T, Strenger C, Keysary A, Harrus S. Kinetics of serologic cross-reactions between Ehrlichia canis and the Ehrlichia phagocytophila genogroups in experimental E-canis infection in dogs. Veterinary Immunology and Immunopathology. 1998;66(3-4):237-43. doi: 10.1016/s0165-2427(98)00198-6. PubMed PMID: WOS:000077693100003.

453. Webster P, JW IJ, Chicoine LM, Fikrig E. The agent of Human Granulocytic Ehrlichiosis resides in an endosomal compartment. J Clin Invest. 1998;101(9):1932-41. Epub 1998/06/13. doi: 10.1172/jci1544. PubMed PMID: 9576758; PubMed Central PMCID: PMCPMC508780.

454. White DJ, Talarico J, Chang HG, Birkhead GS, Heimberger T, Morse DL. Human babesiosis in New York State - Review of 139 hospitalized cases and analysis of prognostic factors. Archives of Internal Medicine. 1998;158(19):2149-54. doi: 10.1001/archinte.158.19.2149. PubMed PMID: WOS:000076577500010.

455. Wong SJ, Thomas JA. Cytoplasmic, nuclear, and platelet autoantibodies in human granulocytic ehrlichiosis patients. J Clin Microbiol. 1998;36(7):1959-63. Epub 1998/07/03. doi: 10.1128/jcm.36.7.1959-1963.1998. PubMed PMID: 9650944; PubMed Central PMCID: PMCPMC104960.

456. Wormser GP, Nowakowski J, Nadelman RB, Schwartz I, McKenna D, Holmgren D, et al. Efficacy of an OspA vaccine preparation for prevention of Lyme disease in New York state. Infection. 1998;26(4):208-12. doi: 10.1007/bf02962365. PubMed PMID: WOS:000075228400003.

457. Zhi N, Ohashi N, Rikihisa Y, Horowitz HW, Wormser GP, Hechemy K. Cloning and expression of the 44-kilodalton major outer membrane protein gene of the human granulocytic ehrlichiosis agent and application of the recombinant protein to serodiagnosis. J Clin Microbiol. 1998;36(6):1666-73. Epub 1998/06/10. doi: 10.1128/jcm.36.6.1666-1673.1998. PubMed PMID: 9620397; PubMed Central PMCID: PMCPMC104897.

458. Alberdi MP, Paxton EA, Clark AM, Watson P, Sumption KJ, Walker AR, editors. Sequence analysis of groEL gene reveals further differences between granulocytic Ehrlichia isolates of Europe and the United States. 3rd International Conference on Ticks and Tick-Borne Pathogens (TTP 3); 1999 Aug 30-Sep 03; High Tatra Mt, Slovakia2000.

459. Arraga-Alvarado C, Palmar M, Parra O, Salas P. Fine structural characterisation of a Rickettsia-like organism in human platelets from patients with symptoms of ehrlichiosis. J Med Microbiol. 1999;48(11):991-7. Epub 1999/10/27. doi: 10.1099/00222615-48-11-991. PubMed PMID: 10535642.

460. Artursson K, Gunnarsson A, Wikstrom UB, Engvall EO. A serological and clinical follow-up in horses with confirmed equine granulocytic ehrlichiosis. Equine Veterinary Journal. 1999;31(6):473-7. doi: 10.1111/j.2042-3306.1999.tb03853.x. PubMed PMID: WOS:000083614600006.

461. Arulkanthan A, Brown WC, McGuire TC, Knowles DP. Biased immunoglobulin G1 isotype responses induced in cattle with DNA expressing msp1a of Anaplasma marginale. Infect Immun. 1999;67(7):3481-7. Epub 1999/06/22. doi: 10.1128/iai.67.7.3481-3487.1999. PubMed PMID: 10377129; PubMed Central PMCID: PMCPMC116534.

462. Barnewall RE, Ohashi N, Rikihisa Y. Ehrlichia chaffeensis and E-sennetsu, but not the human granulocytic ehrlichiosis agent, colocalize with transferrin receptor and up-regulate transferrin receptor mRNA by activating iron-responsive protein 1. Infection and Immunity. 1999;67(5):2258-65. doi: 10.1128/iai.67.5.2258-2265.1999. PubMed PMID: WOS:000079909300029.

463. Baumgarten BU, Röllinghoff M, Bogdan C. Prevalence of Borrelia burgdorferi and granulocytic and monocytic ehrlichiae in Ixodes ricinus ticks from southern Germany. J Clin Microbiol. 1999;37(11):3448-51. Epub 1999/10/19. doi: 10.1128/jcm.37.11.3448-3451.1999. PubMed PMID: 10523532; PubMed Central PMCID: PMCPMC85664.

464. Bjöersdorff A, Berglund J, Kristiansen BE, Söderström C, Eliasson I. [Varying clinical picture and course of human granulocytic ehrlichiosis. Twelve Scandinavian cases of the new tick-borne zoonosis are presented]. Lakartidningen. 1999;96(39):4200-4. Epub 1999/11/02. PubMed PMID: 10544585.

465. Bjöersdorff A, Brouqui P, Eliasson I, Massung RF, Wittesjö B, Berglund J. Serological evidence of Ehrlichia infection in Swedish Lyme borreliosis patients. Scand J Infect Dis. 1999;31(1):51-5. Epub 1999/06/25. doi: 10.1080/00365549950161880. PubMed PMID: 10381218.

466. Bowie MV, Reddy GR, Semu SM, Mahan SM, Barbet AF. Potential value of major antigenic protein 2 for serological diagnosis of heartwater and related ehrlichial infections. Clinical and Diagnostic Laboratory Immunology. 1999;6(2):209-15. doi: 10.1128/cdli.6.2.209-215.1999. PubMed PMID: WOS:000079046800012.

467. Braclik M, Kucharz EJ. Human ehrlichioses. Polskie Archiwum Medycyny Wewnetrznej. 1999;101(3):241-3.

468. Brandsma AR, Little SE, Lockhart JM, Davidson WR, Stallknecht DE, Dawson JE. Novel Ehrlichia organism (Rickettsiales : Ehrlichieae) in white-tailed deer associated with lone star tick (Acari : Ixodidae) parasitism. Journal of Medical Entomology. 1999;36(2):190-4. doi: 10.1093/jmedent/36.2.190. PubMed PMID: WOS:000078828300013.

469. Brouqui P. Adaptation of the agent of the human monocyte ehrlichiosis (Ehrlichia chaffeensis) to HL60. Am J Trop Med Hyg. 1999;60(4):518-9. Epub 1999/05/29. doi: 10.4269/ajtmh.1999.60.518. PubMed PMID: 10348219.

470. Buller RS, Arens M, Hmiel SP, Paddock CD, Sumner JW, Rikhisa Y, et al. Ehrlichia ewingii, a newly recognized agent of human ehrlichiosis. N Engl J Med. 1999;341(3):148-55. Epub 1999/07/15. doi: 10.1056/nejm199907153410303. PubMed PMID: 10403852.

471. Bunnell JE, Magnarelli LA, Dumler JS. Infection of laboratory mice with the human granulocytic ehrlichiosis agent does not induce antibodies to diagnostically significant Borrelia burgdorferi antigens. J Clin Microbiol. 1999;37(6):2077-9. Epub 1999/05/15. doi: 10.1128/jcm.37.6.2077-2079.1999. PubMed PMID: 10325386; PubMed Central PMCID: PMCPMC85039.

472. Bunnell JE, Trigiani ER, Srinivas SR, Dumler JS. Development and distribution of pathologic lesions are related to immune status and tissue deposition of human granulocytic ehrlichiosis agent-infected cells in a murine model system. J Infect Dis. 1999;180(2):546-50. Epub 1999/07/09. doi: 10.1086/314902. PubMed PMID: 10395880.

473. Caracappa S. Livestock production and animal health in Sicily, Italy. Parassitologia. 1999;41 Suppl 1:17-23. Epub 2000/11/09. PubMed PMID: 11071536.

474. Childs JE, Ellis BA, Nicholson WL, Kosoy M, Sumner JW. Shared vector-borne zoonoses of the Old World and New World: home grown or translocated? Schweiz Med Wochenschr. 1999;129(31-32):1099-105. Epub 1999/09/07. PubMed PMID: 10476548.

475. Childs JE, Sumner JW, Nicholson WL, Massung RF, Standaert SM, Paddock CD. Outcome of diagnostic tests using samples from patients with culture-proven human monocytic ehrlichiosis: Implications for surveillance. Journal of Clinical Microbiology. 1999;37(9):2997-3000. doi: 10.1128/jcm.37.9.2997-3000.1999. PubMed PMID: WOS:000082038900045.

476. Christova IS, Dumler JS. Human granulocytic ehrlichiosis in Bulgaria. Am J Trop Med Hyg. 1999;60(1):58-61. Epub 1999/02/13. doi: 10.4269/ajtmh.1999.60.58. PubMed PMID: 9988323.

477. Comer JA, Nicholson WL, Sumner JW, Olson JG, Childs JE. Diagnosis of human ehrlichiosis by PCR assay of acute-phase serum. J Clin Microbiol. 1999;37(1):31-4. Epub 1998/12/17. doi: 10.1128/jcm.37.1.31-34.1999. PubMed PMID: 9854059; PubMed Central PMCID: PMCPMC84159.

478. Des Vignes F, Levin ML, Fish D. Comparative vector competence of Dermacentor variabilis and Ixodes scapularis (Acari: Ixodidae) for the agent of human granulocytic ehrlichiosis. J Med Entomol. 1999;36(2):182-5. Epub 1999/03/20. doi: 10.1093/jmedent/36.2.182. PubMed PMID: 10083755.

479. Dumler JS, Valsamakis A. Molecular diagnostics for existing and emerging infections - Complementary tools for a new era of clinical microbiology. American Journal of Clinical Pathology. 1999;112(1):S33-S9. PubMed PMID: WOS:000081173400004.

480. Dumpis U, Crook D, Oksi J. Tick-borne encephalitis. Clinical Infectious Diseases. 1999;28(4):882-90. doi: 10.1086/515195. PubMed PMID: WOS:000079611600034.

481. Edlow JA. Lyme disease and related tick-borne illnesses. Annals of Emergency Medicine. 1999;33(6):680-93. doi: 10.1016/s0196-0644(99)80007-9. PubMed PMID: WOS:000080626200007.

482. Estrada-Pena A, Jongejan F. Ticks feeding on humans: a review of records on human-biting Ixodoidea with special reference to pathogen transmission. Experimental and Applied Acarology. 1999;23(9):685-715. doi: 10.1023/a:1006241108739. PubMed PMID: WOS:000082469100001.

483. Faul JL, Doyle RL, Kao PN, Ruoss SJ. Tick-borne pulmonary disease - Update on diagnosis and management. Chest. 1999;116(1):222-30. doi: 10.1378/chest.116.1.222. PubMed PMID: WOS:000081513200037.

484. Fingerle V, Goodman JL, Johnson RC, Kurtti TJ, Munderloh UG, Wilske B. un. Wien Klin Wochenschr. 1999;111(22-23):1000-4. Epub 2000/02/10. PubMed PMID: 10666819.

485. Fingerle V, Munderloh UG, Liegl G, Wilske B. Coexistence of ehrlichiae of the phagocytophila group with Borrelia burgdorferi in Ixodes ricinus from Southern Germany. Medical Microbiology and Immunology. 1999;188(3):145-9. doi: 10.1007/s004300050117. PubMed PMID: WOS:000085309400006.

486. Foley JE, Foley P, Jecker M, Swift PK, Madigan JE. Granulocytic ehrlichiosis and tick infestation in mountain lions in California. Journal of Wildlife Diseases. 1999;35(4):703-9. doi: 10.7589/0090-3558-35.4.703. PubMed PMID: WOS:000087475300008.

487. Foley JE, Lerche NW, Dumler JS, Madigan JE. A simian model of human granulocytic ehrlichiosis. Am J Trop Med Hyg. 1999;60(6):987-93. Epub 1999/07/14. doi: 10.4269/ajtmh.1999.60.987. PubMed PMID: 10403332.

488. French DM, Brown WC, Palmer GH. Emergence of Anaplasma marginale antigenic variants during persistent rickettsemia. Infect Immun. 1999;67(11):5834-40. Epub 1999/10/26. doi: 10.1128/iai.67.11.5834-5840.1999. PubMed PMID: 10531237; PubMed Central PMCID: PMCPMC96963.

489. Golightly MG, Benach J. Tick-borne diseases. Reviews in Medical Microbiology. 1999;10(1):1-10. PubMed PMID: WOS:000079349400001.

490. Gongora Biachi RA, Velazquez JZ, Castro Sansores CJ, Martinez PG. First case of human ehrlichiosis in Mexico. Enfermedades Infecciosas y Microbiologia. 1999;19(3):139.

491. Goodman JL. Ehrlichiosis--ticks, dogs, and doxycycline. N Engl J Med. 1999;341(3):195-7. Epub 1999/07/15. doi: 10.1056/nejm199907153410311. PubMed PMID: 10403860.

492. Goodman JL, Nelson CM, Klein MB, Hayes SF, Weston BW. Leukocyte infection by the granulocytic ehrlichiosis agent is linked to expression of a selectin ligand. J Clin Invest. 1999;103(3):407-12. Epub 1999/02/02. doi: 10.1172/jci4230. PubMed PMID: 9927502; PubMed Central PMCID: PMCPMC407896.

493. Granstrom M. New emerging tick-borne diseases. Bulletin De L Academie Nationale De Medecine. 1999;183(7):1391-8. PubMed PMID: WOS:000084662800012.

494. Greenberg SB. Serious waterborne and wilderness infections. Critical Care Clinics. 1999;15(2):387-+. doi: 10.1016/s0749-0704(05)70060-9. PubMed PMID: WOS:000080605800010.

495. Gubbels JM, De Vos AP, Van Der Weide M, Viseras J, Schouls LM, De Vries E, et al. Simultaneous detection of bovine Theileria and Babesia species by reverse line blot hybridization. Journal of Clinical Microbiology. 1999;37(6):1782-9. doi: 10.1128/jcm.37.6.1782-1789.1999.

496. Gustafson R, Artursson K. [Ehrlichiosis is common among animals but can also occur in humans]. Lakartidningen. 1999;96(37):3884-7. Epub 1999/10/16. PubMed PMID: 10522093.

497. Hilton E, DeVoti J, Benach JL, Halluska ML, White DJ, Paxton H, et al. Seroprevalence and seroconversion for tick-borne diseases in a high-risk population in the northeast United States. Am J Med. 1999;106(4):404-9. Epub 1999/05/04. doi: 10.1016/s0002-9343(99)00046-7. PubMed PMID: 10225242.

498. Horowitz HW, Aguero-Rosenfeld ME, Wormser GP. Recurrent human granulocytic ehrlichiosis and Lyme disease - In response. Annals of Internal Medicine. 1999;130(12):1029-30. doi: 10.7326/0003-4819-130-12-199906150-00101. PubMed PMID: WOS:000080894700022.

499. Hsieh T, DiPietrantonio AM, Horowitz HW, Dumler JS, Aguero-Rosenfeld ME, Wormser GP, et al. Changes in expression of the 44-kilodalton outer surface membrane antigen (p44 kD) for monitoring progression of infection and antimicrobial susceptibility of the human granulocytic ehrlichiosis (HGE) agent in HL-60 cells. Biochem Biophys Res Commun. 1999;257(2):351-5. Epub 1999/04/13. doi: 10.1006/bbrc.1999.0457. PubMed PMID: 10198216.

500. Hunfeld KP, Brade V. Prevalence of antibodies against the human granulocytic ehrlichiosis agent in Lyme borreliosis patients from Germany. Eur J Clin Microbiol Infect Dis. 1999;18(3):221-4. Epub 1999/06/05. doi: 10.1007/s100960050264. PubMed PMID: 10357060.

501. Jerrard D. Ehrlichiosis. Journal of Emergency Medicine. 1999;17(1):27-30. doi: 10.1016/s0736-4679(98)00117-6. PubMed PMID: WOS:000082179300005.

502. Johnson JR, Horowitz HW, Aguero-Rosenfeld ME, Wormser GP. Recurrent human granulocytic ehrlichiosis and Lyme disease [4] (multiple letters). Annals of Internal Medicine. 1999;130(12):1029-30. doi: 10.7326/0003-4819-130-12-199906150-00014.

503. JW IJ, Wu C, Magnarelli LA, Fikrig E. Serodiagnosis of human granulocytic ehrlichiosis by a recombinant HGE-44-based enzyme-linked immunosorbent assay. J Clin Microbiol. 1999;37(11):3540-4. Epub 1999/10/19. doi: 10.1128/jcm.37.11.3540-3544.1999. PubMed PMID: 10523549; PubMed Central PMCID: PMCPMC85687.

504. Kordick SK, Breitschwerdt EB, Hegarty BC, Southwick KL, Colitz CM, Hancock SI, et al. Coinfection with multiple tick-borne pathogens in a Walker Hound kennel in North Carolina. J Clin Microbiol. 1999;37(8):2631-8. Epub 1999/07/16. doi: 10.1128/jcm.37.8.2631-2638.1999. PubMed PMID: 10405413; PubMed Central PMCID: PMCPMC85300.

505. Kramer VL, Randolph MP, Hui LT, Irwin WE, Gutierrez AG, Vugia DJ. Detection of the agents of human ehrlichioses in ixodid ticks from California. Am J Trop Med Hyg. 1999;60(1):62-5. Epub 1999/02/13. doi: 10.4269/ajtmh.1999.60.62. PubMed PMID: 9988324.

506. Krupa W, Pancewicz S, Snarska-Furła I, Zajkowska J. [Ehrlichiosis: a tick-born infection]. Pol Merkur Lekarski. 1999;6(32):107-9. Epub 1999/05/25. PubMed PMID: 10337186.

507. Leutenegger CM, Pusterla N, Mislin CN, Weber R, Lutz H. Molecular evidence of coinfection of ticks with Borrelia burgdorferi sensu late and the human granulocytic ehrlichiosis agent in Switzerland. Journal of Clinical Microbiology. 1999;37(10):3390-1. doi: 10.1128/jcm.37.10.3390-3391.1999. PubMed PMID: WOS:000082644800058.

508. Levin ML, des Vignes F, Fish D. Disparity in the natural cycles of Borrelia burgdorferi and the agent of human granulocytic ehrlichiosis. Emerging Infectious Diseases. 1999;5(2):204-8. doi: 10.3201/eid0502.990203. PubMed PMID: WOS:000079735500003.

509. Lilliehook I, Johannisson A, Magnusson U, Egenvall A, Trowald-Wigh G, Hakansson L. Granulocyte function in dogs experimentally infected with a Swedish granulocytic Ehrlichia species. Veterinary Immunology and Immunopathology. 1999;67(2):141-52. doi: 10.1016/s0165-2427(98)00219-0. PubMed PMID: WOS:000078816000004.

510. Little SE, Howerth EW. Ehrlichia chaffeensis in archived tissues of a white-tailed deer. Journal of Wildlife Diseases. 1999;35(3):596-9. doi: 10.7589/0090-3558-35.3.596. PubMed PMID: WOS:000081607700025.

511. Magnarelli LA, Ijdo JW, Stafford KC, Fikrig E. Infections of granulocytic ehrlichiae and Borrelia burgdorferi in white-tailed deer in Connecticut. Journal of Wildlife Diseases. 1999;35(2):266-74. doi: 10.7589/0090-3558-35.2.266. PubMed PMID: WOS:000080009000012.

512. Magnarelli LA, Stafford KC, Ijdo JW, Fikrig E, Oliver JH, Hutcheson HJ, et al. Antibodies to granulocytic ehrlichiae in white-footed and cotton mice in eastern United States. Journal of Wildlife Diseases. 1999;35(2):259-65. doi: 10.7589/0090-3558-35.2.259. PubMed PMID: WOS:000080009000011.

513. Mahan SM, Allsopp B, Kocan KM, Palmer GH, Jongejan F. Vaccine strategies for Cowdria ruminantium infections and their application to other ehrlichial infections. Parasitology Today. 1999;15(7):290-4. doi: 10.1016/s0169-4758(99)01468-4. PubMed PMID: WOS:000081360300009.

514. Mauel MJ, Carlton SJ, Mather TN. Polymerase chain reaction detection efficiency of the human granulocytic ehrlichiosis agent (Rickettsiaceae: Ehrlichieae) in ticks (Acari: Ixodidae) is dependent on the DNA extraction method. J Med Entomol. 1999;36(6):649-52. Epub 1999/12/11. doi: 10.1093/jmedent/36.6.649. PubMed PMID: 10593061.

515. McHolland LE, Caldwell DR. Pyruvate metabolism by Anaplasma marginale in cell-free culture. Can J Microbiol. 1999;45(2):185-9. Epub 1999/06/25. doi: 10.1139/w98-215. PubMed PMID: 10380651.

516. Mott J, Barnewall RE, Rikihisa Y. Human granulocytic ehrlichiosis agent and Ehrlichia chaffeensis reside in different cytoplasmic compartments in HL-60 cells. Infect Immun. 1999;67(3):1368-78. Epub 1999/02/20. doi: 10.1128/iai.67.3.1368-1378.1999. PubMed PMID: 10024584; PubMed Central PMCID: PMCPMC96470.

517. Munderloh UG, Jauron SD, Fingerle V, Leitritz L, Hayes SF, Hautman JM, et al. Invasion and intracellular development of the human granulocytic ehrlichiosis agent in tick cell culture. J Clin Microbiol. 1999;37(8):2518-24. Epub 1999/07/16. doi: 10.1128/jcm.37.8.2518-2524.1999. PubMed PMID: 10405394; PubMed Central PMCID: PMCPMC85271.

518. Nicholson WL, Castro MB, Kramer VL, Sumner JW, Childs JE. Dusky-footed wood rats (Neotoma fuscipes) as reservoirs of granulocytic Ehrlichiae (Rickettsiales: Ehrlichieae) in northern California. J Clin Microbiol. 1999;37(10):3323-7. Epub 1999/09/17. doi: 10.1128/jcm.37.10.3323-3327.1999. PubMed PMID: 10488199; PubMed Central PMCID: PMCPMC85556.

519. Ning Z, Ohashi N, Rikihisa Y. Multiple p44 genes encoding major outer membrane proteins are expressed in the human granulocyte ehrlichiosis agent. Journal of Biological Chemistry. 1999;274(25):17828-36. doi: 10.1074/jbc.274.25.17828.

520. Nutt AK, Raufman JP. Gastrointestinal and hepatic manifestations of human ehrlichiosis: 8 cases and a review of the literature. Digestive Diseases. 1999;17(1):37-43. doi: 10.1159/000016901. PubMed PMID: WOS:000081869100005.

521. Oteo JA, Blanco JR. [Granulocytic human ehrlichiosis. A zoonosis emerging in Spain?]. Enferm Infecc Microbiol Clin. 1999;17(6):267-8. Epub 1999/08/10. PubMed PMID: 10439534.

522. Palmer GH, Rurangirwa FR, Kocan KM, Brown WC. Molecular basis for vaccine development against the ehrlichial pathogen Anaplasma marginale. Parasitology Today. 1999;15(7):281-6. doi: 10.1016/s0169-4758(99)01469-6. PubMed PMID: WOS:000081360300007.

523. Petrovec M, Sumner JW, Nicholson WL, Childs JE, Strle F, Barlic J, et al. Identity of ehrlichial DNA sequences derived from Ixodes ricinus ticks with those obtained from patients with human granulocytic ehrlichiosis in Slovenia. J Clin Microbiol. 1999;37(1):209-10. Epub 1998/12/17. doi: 10.1128/jcm.37.1.209-210.1999. PubMed PMID: 9854093; PubMed Central PMCID: PMCPMC84210.

524. Plier ML, Young KM, Barlough JE, Madigan JE, Dumler JS. Equine granulocytic ehrlichiosis: a case report with DNA analysis and species comparison. Vet Clin Pathol. 1999;28(4):127-30. Epub 2002/06/21. doi: 10.1111/j.1939-165x.1999.tb01062.x. PubMed PMID: 12075508.

525. Pretorius AM, Venter TP, Van der Ryst E, Kelly PJ. Possible human Ehrlichiosis in the Free State [10]. South African Medical Journal. 1999;89(9):961.

526. Pusterla N, Huder JB, Leutenegger CM, Braun U, Madigan JE, Lutz H. Quantitative real-time PCR for detection of members of the Ehrlichia phagocytophila genogroup in host animals and Ixodes ricinus ticks. J Clin Microbiol. 1999;37(5):1329-31. Epub 1999/04/16. doi: 10.1128/jcm.37.5.1329-1331.1999. PubMed PMID: 10203480; PubMed Central PMCID: PMCPMC84766.

527. Pusterla N, Leutenegger CM, Chae JS, Lutz H, Kimsey RB, Dumler JS, et al. Quantitative evaluation of ehrlichial burden in horses after experimental transmission of human granulocytic Ehrlichia agent by intravenous inoculation with infected leukocytes and by infected ticks. J Clin Microbiol. 1999;37(12):4042-4. Epub 1999/11/24. doi: 10.1128/jcm.37.12.4042-4044.1999. PubMed PMID: 10565928; PubMed Central PMCID: PMCPMC85876.

528. Pusterla N, Leutenegger CM, Huder JB, Weber R, Braun U, Lutz H. Evidence of the human granulocytic ehrlichiosis agent in Ixodes ricinus ticks in Switzerland. J Clin Microbiol. 1999;37(5):1332-4. Epub 1999/04/16. doi: 10.1128/jcm.37.5.1332-1334.1999. PubMed PMID: 10203481; PubMed Central PMCID: PMCPMC84767.

529. Pusterla N, Pusterla JB, Braun U, Lutz H. Experimental cross-infections with Ehrlichia phagocytophila and human granulocytic ehrlichia-like agent in cows and horses. Vet Rec. 1999;145(11):311-4. Epub 1999/10/09. doi: 10.1136/vr.145.11.311. PubMed PMID: 10515618.

530. Ravyn MD, Lamb LJ, Jemmerson R, Goodman JL, Johnson RC. Characterization of monoclonal antibodies to an immunodominant protein of the etiologic agent of human granulocytic ehrlichiosis. Am J Trop Med Hyg. 1999;61(1):171-6. Epub 1999/08/04. doi: 10.4269/ajtmh.1999.61.171. PubMed PMID: 10432075.

531. Rydkina E, Roux V, Raoult D. Determination of the genome size of Ehrlichia spp., using pulsed field gel electrophoresis. FEMS Microbiol Lett. 1999;176(1):73-8. Epub 1999/07/27. doi: 10.1111/j.1574-6968.1999.tb13644.x. PubMed PMID: 10418133.

532. Sadikot R, Shaver MJ, Reeves WB. Ehrlichia chaffeensis in a renal transplant recipient. American Journal of Nephrology. 1999;19(6):674-6. doi: 10.1159/000013540. PubMed PMID: WOS:000084083500008.

533. Schouls LM, Van De Pol I, Rijpkema SG, Schot CS. Detection and identification of Ehrlichia, Borrelia burgdorferi sensu lato, and Bartonella species in Dutch Ixodes ricinus ticks. J Clin Microbiol. 1999;37(7):2215-22. Epub 1999/06/12. doi: 10.1128/jcm.37.7.2215-2222.1999. PubMed PMID: 10364588; PubMed Central PMCID: PMCPMC85121.

534. Sigal LH. Borrelia burgdorferi outer surface protein A vaccine: Importance, role, and value. Journal of Pediatrics. 1999;135(5):539-41. doi: 10.1016/s0022-3476(99)70048-x. PubMed PMID: WOS:000083519400004.

535. Sparagano OAE, Allsopp M, Mank RA, Rijpkema SGT, Figueroa JV, Jongejan F. Molecular detection of pathogen DNA in ticks (Acari : Ixodidae): A review. Experimental and Applied Acarology. 1999;23(12):929-60. doi: 10.1023/a:1006313803979. PubMed PMID: WOS:000086102900001.

536. Stafford KC, 3rd, Massung RF, Magnarelli LA, Ijdo JW, Anderson JF. Infection with agents of human granulocytic ehrlichiosis, lyme disease, and babesiosis in wild white-footed mice (Peromyscus leucopus) in Connecticut. J Clin Microbiol. 1999;37(9):2887-92. Epub 1999/08/17. doi: 10.1128/jcm.37.9.2887-2892.1999. PubMed PMID: 10449470; PubMed Central PMCID: PMCPMC85405.

537. Stefan N, Elsner S, Schnaidt M, Wernet D, Stumvoll M. Thrombocytopenia and Borrelia burgdorferi: An association remains unproven - Reply. Clinical Infectious Diseases. 1999;29(6):1604-5. doi: 10.1086/313565. PubMed PMID: WOS:000084693400072.

538. Sulsona CR, Mahan SM, Barbet AF. The map1 gene of Cowdria ruminantium is a member of a multigene family containing both conserved and variable genes. Biochemical and Biophysical Research Communications. 1999;257(2):300-5. doi: 10.1006/bbrc.1999.0459. PubMed PMID: WOS:000079925000010.

539. Thomas DR, Salmon RL, Coleman TJ, Morgan-Capner P, Sillis M, Caul EO, et al. Occupational exposure to animals and risk of zoonotic illness in a cohort of farmers, farmworkers, and their families in England. Journal of Agricultural Safety and Health. 1999;5(4):373-82.

540. Tuo W, MacMillan H, Günter N, Bazer FW, Brown WC. Upregulation of interleukin-4 and IFN-γ expression by IFN-τ, a member of the type I IFN family. Journal of Interferon and Cytokine Research. 1999;19(2):179-87. doi: 10.1089/107999099314324.

541. Walls JJ, Aguero-Rosenfeld M, Bakken JS, Goodman JL, Hossain D, Johnson RC, et al. Inter- and intralaboratory comparison of Ehrlichia equi and human granulocytic ehrlichiosis (HGE) agent strains for serodiagnosis of HGE by the immunofluorescent-antibody test. J Clin Microbiol. 1999;37(9):2968-73. Epub 1999/08/17. doi: 10.1128/jcm.37.9.2968-2973.1999. PubMed PMID: 10449483; PubMed Central PMCID: PMCPMC85424.

542. Waner T, Baneth G, Strenger C, Keysary A, King R, Harrus S. Antibodies reactive with Ehrlichia canis, Ehrlichia phagocytophila genogroup antigens and the spotted fever group rickettsial antigens, in free-ranging jackals (Canis aureus syriacus) from Israel. Vet Parasitol. 1999;82(2):121-8. Epub 1999/05/13. doi: 10.1016/s0304-4017(99)00002-3. PubMed PMID: 10321583.

543. Wormser GP. Vaccination as a modality to prevent Lyme disease - A status report. Infectious Disease Clinics of North America. 1999;13(1):135-+. doi: 10.1016/s0891-5520(05)70047-7. PubMed PMID: WOS:000079651800011.

544. Wu JM, Whyzmuzis CA, Bertone MG, Zhou BS, Hsieh TC. Quantification of the human granulocytic ehrlichiosis agent based on analysis of rRNA isolated from control and infected HL-60 cells. Biochem Biophys Res Commun. 1999;262(1):7-13. Epub 1999/08/17. doi: 10.1006/bbrc.1999.1134. PubMed PMID: 10448059.

545. Zeman P, Pazdiora P, Rebl K, Lukesova D, Klanica J. Granulocytic ehrlichiosis also in the Czech Republic? Prakticky Lekar. 1999;79(10):575-7.

546. Zhi N, Ohashi N, Rikihisa Y. Multiple p44 genes encoding major outer membrane proteins are expressed in the human granulocytic ehrlichiosis agent. J Biol Chem. 1999;274(25):17828-36. Epub 1999/06/11. doi: 10.1074/jbc.274.25.17828. PubMed PMID: 10364227.

547. Aguero-Rosenfeld ME, Kalantarpour F, Baluch M, Horowitz HW, McKenna DF, Raffalli JT, et al. Serology of culture-confirmed cases of human granulocytic ehrlichiosis. J Clin Microbiol. 2000;38(2):635-8. Epub 2000/02/03. doi: 10.1128/jcm.38.2.635-638.2000. PubMed PMID: 10655359; PubMed Central PMCID: PMCPMC86164.

548. Akkoyunlu M, Fikrig E. Gamma interferon dominates the murine cytokine response to the agent of human granulocytic ehrlichiosis and helps to control the degree of early rickettsemia. Infect Immun. 2000;68(4):1827-33. Epub 2000/03/18. doi: 10.1128/iai.68.4.1827-1833.2000. PubMed PMID: 10722570; PubMed Central PMCID: PMCPMC97354.

549. Alberdi MP, Walker AR, Urquhart KA. Field evidence that roe deer (Capreolus capreolus) are a natural host for Ehrlichia phagocytophila. Epidemiology & Infection. 2000;124(2):315-23. doi: 10.1017/S0950268899003684. PubMed PMID: 104720938. Language: English. Entry Date: 20110610. Revision Date: 20190529. Publication Type: journal article.

550. Alleman AR, Barbet AF, Bowie MV, Sorenson HL, Wong SJ, Belanger M. Expression of a gene encoding the major antigenic protein 2 homolog of Ehrlichia chaffeensis and potential application for serodiagnosis. Journal of Clinical Microbiology. 2000;38(10):3705-9. doi: 10.1128/jcm.38.10.3705-3709.2000. PubMed PMID: WOS:000089707600030.

551. Aquilini D, Parola P, Salvo E, Paladini A. Seroepidemiology of the rickettsioses, human granulocytic ehrlichiosis, Lyme disease, Q fever, and tularemia in forestry workers in Tuscany, Italy. Journal of Spirochetal and Tick-borne Diseases. 2000;7(FALL):35-41.

552. Bakken JS, Dumler JS. Human granulocytic ehrlichiosis. Clin Infect Dis. 2000;31(2):554-60. Epub 2000/09/15. doi: 10.1086/313948. PubMed PMID: 10987720.

553. Banerjee R, Anguita J, Fikrig E. Granulocytic ehrlichiosis in mice deficient in phagocyte oxidase or inducible nitric oxide synthase. Infect Immun. 2000;68(7):4361-2. Epub 2000/06/17. doi: 10.1128/iai.68.7.4361-4362.2000. PubMed PMID: 10858261; PubMed Central PMCID: PMCPMC101771.

554. Banerjee R, Anguita J, Roos D, Fikrig E. Cutting edge: infection by the agent of human granulocytic ehrlichiosis prevents the respiratory burst by down-regulating gp91phox. J Immunol. 2000;164(8):3946-9. Epub 2001/02/07. doi: 10.4049/jimmunol.164.8.3946. PubMed PMID: 10754283.

555. Barbet AF, Lundgren A, Yi J, Rurangirwa FR, Palmer GH. Antigenic variation of Anaplasma marginale by expression of MSP2 mosaics. Infect Immun. 2000;68(11):6133-8. Epub 2000/10/18. doi: 10.1128/iai.68.11.6133-6138.2000. PubMed PMID: 11035716; PubMed Central PMCID: PMCPMC97690.

556. Barbour AG, Restrepo BI. Antigenic variation in vector-borne pathogens. Emerg Infect Dis. 2000;6(5):449-57. Epub 2000/09/22. doi: 10.3201/eid0605.000502. PubMed PMID: 10998374; PubMed Central PMCID: PMCPMC2627965.

557. Bätzing-Feigenbaum J, Kallischnigg G, Rüden H, Talaska T. [Human granulocytic ehrlichiosis. New tick bite disease lies in wait also in German forests]. MMW Fortschr Med. 2000;142(35):32-4. Epub 2000/09/28. PubMed PMID: 11006704.

558. Batzing-Feigenbaum J, Kallischnigg G, Ruden H, Talaska T. 'Human granulocytic ehrlichiosis'. An uncommon infection transmitted by ticks. MMW-Fortschritte der Medizin. 2000;142(35):32-4.

559. Beeching NJ, Hart CA, Duerden BI. Tropical and exotic infections. Journal of Medical Microbiology. 2000;49(1):5-27. doi: 10.1099/0022-1317-49-1-5. PubMed PMID: WOS:000084606400002.

560. Behl R, Klein MB, Dandelet L, Bach RR, Goodman JL, Key NS. Induction of tissue factor procoagulant activity in myelomonocytic cells inoculated by the agent of human granulocytic ehrlichiosis. Thromb Haemost. 2000;83(1):114-8. Epub 2000/02/11. PubMed PMID: 10669164.

561. Brouqui P, Dumler JS. Serologic evidence of human monocytic and granulocytic ehrlichiosis in Israel. Emerg Infect Dis. 2000;6(3):314-5. Epub 2000/05/29. doi: 10.3201/eid0603.000316. PubMed PMID: 10827125; PubMed Central PMCID: PMCPMC2640865.

562. Bullock PM, Ames TR, Robinson RA, Greig B, Mellencamp MA, Dumler JS. Ehrlichia equi infection of horses from Minnesota and Wisconsin: detection of seroconversion and acute disease investigation. J Vet Intern Med. 2000;14(3):252-7. Epub 2000/06/01. doi: 10.1892/0891-6640(2000)014<0252:eiohfm>2.3.co;2. PubMed PMID: 10830537.

563. Callahan EF, Adal KA, Tomecki KJ. Cutaneous (non-HIV) infections. Dermatologic Clinics. 2000;18(3):497-+. doi: 10.1016/s0733-8635(05)70197-2. PubMed PMID: WOS:000088493600013.

564. Cao WC, Gao YM, Zhang PH, Zhang XT, Dai QH, Dumler JS, et al. Identification of Ehrlichia chaffeensis by nested PCR in ticks from southern China. Journal of Clinical Microbiology. 2000;38(7):2778-80. doi: 10.1128/jcm.38.7.2778-2780.2000. PubMed PMID: WOS:000088042100062.

565. Cao WC, Zhao QM, Zhang PH, Dumler JS, Zhang XT, Fang LQ, et al. Granulocytic Ehrlichiae in Ixodes persulcatus ticks from an area in China where Lyme disease is endemic. J Clin Microbiol. 2000;38(11):4208-10. Epub 2000/11/04. doi: 10.1128/jcm.38.11.4208-4210.2000. PubMed PMID: 11060091; PubMed Central PMCID: PMCPMC87564.

566. Caturegli P, Asanovich KM, Walls JJ, Bakken JS, Madigan JE, Popov VL, et al. ankA: an Ehrlichia phagocytophila group gene encoding a cytoplasmic protein antigen with ankyrin repeats. Infect Immun. 2000;68(9):5277-83. Epub 2000/08/19. doi: 10.1128/iai.68.9.5277-5283.2000. PubMed PMID: 10948155; PubMed Central PMCID: PMCPMC101789.

567. Chae JS, Foley JE, Dumler JS, Madigan JE. Comparison of the nucleotide sequences of 16S rRNA, 444 Ep-ank, and groESL heat shock operon genes in naturally occurring Ehrlichia equi and human granulocytic ehrlichiosis agent isolates from Northern California. J Clin Microbiol. 2000;38(4):1364-9. Epub 2000/04/04. doi: 10.1128/jcm.38.4.1364-1369.2000. PubMed PMID: 10747108; PubMed Central PMCID: PMCPMC86446.

568. Chang YF, McDonough SP, Chang CF, Shin KS, Yen W, Divers T. Human granulocytic ehrlichiosis agent infection in a pony vaccinated with a Borrelia burgdorferi recombinant OspA vaccine and challenged by exposure to naturally infected ticks. Clin Diagn Lab Immunol. 2000;7(1):68-71. Epub 2000/01/05. doi: 10.1128/cdli.7.1.68-71.2000. PubMed PMID: 10618280; PubMed Central PMCID: PMCPMC95825.

569. Chomel B. Emerging bacterial zoonoses. Point Veterinaire. 2000;31(207):15-22. PubMed PMID: WOS:000087437600005.

570. Cizman M, Avsic-Zupanc T, Petrovec M, Ruzic-Sabljic E, Pokorn M. Seroprevalence of ehrlichiosis, Lyme borreliosis and tick-borne encephalitis infections in children and young adults in Slovenia. Wien Klin Wochenschr. 2000;112(19):842-5. Epub 2000/12/01. PubMed PMID: 11098535.

571. Comer JA, Nicholson WL, Paddock CD, Sumner JW, Childs JE. Detection of antibodies reactive with Ehrlichia chaffeensis in the raccoon. Journal of Wildlife Diseases. 2000;36(4):705-12. doi: 10.7589/0090-3558-36.4.705. PubMed PMID: WOS:000165170300010.

572. Curran KL, Kidd JB, Vassallo J, Van Meter VL. Borrelia burgdorferi and the causative agent of human granulocytic ehrlichiosis in deer ticks, Delaware. Emerg Infect Dis. 2000;6(4):408-11. Epub 2000/07/25. doi: 10.3201/eid0604.000417. PubMed PMID: 10905979; PubMed Central PMCID: PMCPMC2640882.

573. De Waal DT. Anaplasmosis control and diagnosis in South Africa. 2000. p. 474-83.

574. DiPietrantonio AM, Hsieh TC, Wu JM. Specific processing of poly(ADP-ribose) polymerase, accompanied by activation of caspase-3 and elevation/reduction of ceramide/hydrogen peroxide levels, during induction of apoptosis in host HL-60 cells infected by the human granulocytic ehrlichiosis (HGE) agent. IUBMB Life. 2000;49(1):49-55. Epub 2000/04/20. doi: 10.1080/713803590. PubMed PMID: 10772341.

575. Dumler JS, Trigiani ER, Bakken JS, Aguero-Rosenfeld ME, Wormser GP. Serum cytokine responses during acute human granulocytic ehrlichiosis. Clin Diagn Lab Immunol. 2000;7(1):6-8. Epub 2000/01/05. doi: 10.1128/cdli.7.1.6-8.2000. PubMed PMID: 10618268; PubMed Central PMCID: PMCPMC95813.

576. Duppenthaler A, Pfammatter JP, Aebi C. Myopericarditis associated with central European tick-borne encephalitis. Eur J Pediatr. 2000;159(11):854-6. Epub 2000/11/18. doi: 10.1007/pl00008353. PubMed PMID: 11079201.

577. Ebel GD, Campbell EN, Goethert HK, Spielman A, Telford SR. Enzootic transmission of deer tick virus in New England and Wisconsin sites. American Journal of Tropical Medicine and Hygiene. 2000;63(1-2):36-42. doi: 10.4269/ajtmh.2000.63.36. PubMed PMID: WOS:000168629200006.

578. Egenvall A, Bonnett BN, Gunnarsson A, Hedhammar A, Shoukri M, Bornstein S, et al. Sero-prevalence of granulocytic Ehrlichia spp. and Borrelia burgdorferi sensu lato in Swedish dogs 1991-94. Scandinavian Journal of Infectious Diseases. 2000;32(1):19-25. doi: 10.1080/00365540050164164. PubMed PMID: 105977423. Language: English. Entry Date: 20080215. Revision Date: 20210331. Publication Type: Journal Article.

579. Egenvall A, Lilliehook I, Bjoersdorff A, Engvall EO, Karlstam E, Artursson K, et al. Detection of granulocytic Ehrlichia species DNA by PCR in persistently infected dogs. Veterinary Record. 2000;146(7):186-90. doi: 10.1136/vr.146.7.186. PubMed PMID: WOS:000085590000010.

580. Evans J. Lyme disease. Current Opinion in Rheumatology. 2000;12(4):311-7. doi: 10.1097/00002281-200007000-00014. PubMed PMID: WOS:000087909000014.

581. Foley JE. Human ehrlichiosis: Review of clinical disease and epidemiology for the physician. Infectious Diseases in Clinical Practice. 2000;9(3):93-8. doi: 10.1097/00019048-200009030-00001. PubMed PMID: WOS:000085520300001.

582. Frohman L, Lama P. Annual update of systemic disease - 1999: Emerging and re-emerging infections (Part II). Journal of Neuro-Ophthalmology. 2000;20(1):48-58. doi: 10.1097/00041327-200020010-00016. PubMed PMID: WOS:000086627200016.

583. García-Pérez AL, Mandaluniz N, Barral M, Juste RA. Microscopic and PCR findings in sheep after experimental infection with Ehrlichia phagocytophila. Small Rumin Res. 2000;37(1-2):19-25. Epub 2000/05/20. doi: 10.1016/s0921-4488(99)00128-5. PubMed PMID: 10818299.

584. Hardalo CJ, Quagliarello V, Dumler JS. Silica and lung cancer: Hazard or risk (multiple letters) [1]. Annals of Occupational Hygiene. 2000;44(4):321.

585. Herron MJ, Nelson CM, Larson J, Snapp KR, Kansas GS, Goodman JL. Intracellular parasitism by the human granulocytic ehrlichiosis bacterium through the P-selectin ligand, PSGL-1. Science. 2000;288(5471):1653-6. Epub 2000/06/02. doi: 10.1126/science.288.5471.1653. PubMed PMID: 10834846.

586. Homer MJ, Aguilar-Delfin I, Telford SR, Krause PJ, Persing DH. Babesiosis. Clinical Microbiology Reviews. 2000;13(3):451-+. doi: 10.1128/cmr.13.3.451-469.2000. PubMed PMID: WOS:000088136300006.

587. Houpikian P, Brouqui P. Ehrlichiosis in children. Journal of Spirochetal and Tick-borne Diseases. 2000;7(FALL):59-63.

588. Hu RJ, Rowley WA. Relationship between weights of the engorged nymphal stage and resultant sexes in Ixodes scapularis and Dermacentor variabilis (Acari : Ixodidae) ticks. Journal of Medical Entomology. 2000;37(1):198-200. doi: 10.1603/0022-2585-37.1.198. PubMed PMID: WOS:000089624900031.

589. JW IJ, Meek JI, Cartter ML, Magnarelli LA, Wu C, Tenuta SW, et al. The emergence of another tickborne infection in the 12-town area around Lyme, Connecticut: human granulocytic ehrlichiosis. J Infect Dis. 2000;181(4):1388-93. Epub 2000/04/06. doi: 10.1086/315389. PubMed PMID: 10751139.

590. Kalantarpour F, Chowdhury I, Wormser GP, Aguero-Rosenfeld ME. Survival of the human granulocytic ehrlichiosis agent under refrigeration conditions. J Clin Microbiol. 2000;38(6):2398-9. Epub 2000/06/02. doi: 10.1128/jcm.38.6.2398-2399.2000. PubMed PMID: 10835014; PubMed Central PMCID: PMCPMC86820.

591. Kelly PJ. Canine ehrlichioses: an update. Journal of the South African Veterinary Association. 2000;71(2):77-86. PubMed PMID: WOS:000089412400002.

592. Kim HY, Rikihisa Y. Expression of interleukin-1beta, tumor necrosis factor alpha, and interleukin-6 in human peripheral blood leukocytes exposed to human granulocytic ehrlichiosis agent or recombinant major surface protein P44. Infect Immun. 2000;68(6):3394-402. Epub 2000/05/19. doi: 10.1128/iai.68.6.3394-3402.2000. PubMed PMID: 10816490; PubMed Central PMCID: PMCPMC97610.

593. Klein MB, Hu S, Chao CC, Goodman JL. The agent of human granulocytic ehrlichiosis induces the production of myelosuppressing chemokines without induction of proinflammatory cytokines. J Infect Dis. 2000;182(1):200-5. Epub 2000/07/07. doi: 10.1086/315641. PubMed PMID: 10882598.

594. Kocan KM, Blouin EF, Barbet AF. Anaplasmosis control: Past, present, and future. 2000. p. 501-9.

595. Krause PJ, Lepore T, Sikand VK, Gadbaw JJ, Burke G, Telford SR, et al. Atovaquone and azithromycin for the treatment of babesiosis. New England Journal of Medicine. 2000;343(20):1454-8. doi: 10.1056/nejm200011163432004. PubMed PMID: WOS:000165271300004.

596. Leal M, Noda A, Reyna-Bello A, Casas B, Précigout E, Aso PM, et al. Identification and characterization of corpuscular, soluble and secreted antigens of a Venezuelan isolate of Anaplasma marginale. Veterinary Parasitology. 2000;94(1-2):1-15. doi: 10.1016/S0304-4017(00)00371-X.

597. Levin ML, Fish D. Acquisition of coinfection and simultaneous transmission of Borrelia burgdorferi and Ehrlichia phagocytophila by Ixodes scapularis ticks. Infection and Immunity. 2000;68(4):2183-6. doi: 10.1128/iai.68.4.2183-2186.2000. PubMed PMID: WOS:000086010300059.

598. Levin ML, Fish D. Immunity reduces reservoir host competence of Peromyscus leucopus for Ehrlichia phagocytophila. Infection and Immunity. 2000;68(3):1514-8. doi: 10.1128/iai.68.3.1514-1518.2000. PubMed PMID: WOS:000085407400069.

599. Liz JS, Anderes L, Sumner JW, Massung RF, Gern L, Rutti B, et al. PCR detection of granulocytic ehrlichiae in Ixodes ricinus ticks and wild small mammals in western Switzerland. J Clin Microbiol. 2000;38(3):1002-7. Epub 2000/03/04. doi: 10.1128/jcm.38.3.1002-1007.2000. PubMed PMID: 10698987; PubMed Central PMCID: PMCPMC86323.

600. Lotric-Furlan S, Petrovec M, Avsic-Zupanc T, Strle F. Clinical distinction between human granulocytic ehrlichiosis and the initial phase of tick-borne encephalitis. J Infect. 2000;40(1):55-8. Epub 2000/04/13. doi: 10.1053/jinf.1999.0587. PubMed PMID: 10762112.

601. Madigan JE, Pusterla N. Ehrlichial diseases. Veterinary Clinics of North America-Equine Practice. 2000;16(3):487-+. doi: 10.1016/s0749-0739(17)30091-3. PubMed PMID: WOS:000166893500008.

602. Magnarelli LA, Ijdo JW, Padula SJ, Flavell RA, Fikrig E. Serologic diagnosis of Lyme borreliosis by using enzyme-linked immunosorbent assays with recombinant antigens. J Clin Microbiol. 2000;38(5):1735-9. Epub 2000/05/02. doi: 10.1128/jcm.38.5.1735-1739.2000. PubMed PMID: 10790090; PubMed Central PMCID: PMCPMC86574.

603. Magnarelli LA, Ijdo JW, Van Andel AE, Wu CY, Padula SJ, Fikrig E. Serologic confirmation of Ehrlichia equi and Borrelia burgdorferi infections in horses from the northeastern United States. Journal of the American Veterinary Medical Association. 2000;217(7):1045-50. doi: 10.2460/javma.2000.217.1045. PubMed PMID: WOS:000089532300028.

604. Mahy BWJ, Brown CC. Emerging zoonoses: crossing the species barrier. Revue Scientifique Et Technique De L Office International Des Epizooties. 2000;19(1):33-40. doi: 10.20506/rst.19.1.1212. PubMed PMID: WOS:000086242500005.

605. Martin ME, Bunnell JE, Dumler JS. Pathology, immunohistology, and cytokine responses in early phases of human granulocytic ehrlichiosis in a murine model. Journal of Infectious Diseases. 2000;181(1):374-8. doi: 10.1086/315206. PubMed PMID: WOS:000085229300050.

606. Massung RF, Owens JH, Ross D, Reed KD, Petrovec M, Bjoersdorff A, et al. Sequence analysis of the ank gene of granulocytic ehrlichiae. J Clin Microbiol. 2000;38(8):2917-22. Epub 2000/08/02. doi: 10.1128/jcm.38.8.2917-2922.2000. PubMed PMID: 10921951; PubMed Central PMCID: PMCPMC87147.

607. McBride JW, Yu XJ, Walker DH. Glycosylation of homologous immunodominant proteins of Ehrlichia chaffeensis and Ehrlichia canis. Infect Immun. 2000;68(1):13-8. Epub 1999/12/22. doi: 10.1128/iai.68.1.13-18.2000. PubMed PMID: 10603362; PubMed Central PMCID: PMCPMC97095.

608. McQuiston JH, Childs JE, Chamberland ME, Tabor E. Transmission of tick-borne agents of disease by blood transfusion: a review of known and potential risks in the United States. Transfusion. 2000;40(3):274-84. doi: 10.1046/j.1537-2995.2000.40030274.x. PubMed PMID: WOS:000086036200004.

609. Melendez RD. Future perspectives on veterinary hemoparasite research in the tropics at the start of this century. Ann N Y Acad Sci. 2000;916:253-8. Epub 2001/02/24. doi: 10.1111/j.1749-6632.2000.tb05297.x. PubMed PMID: 11193629.

610. Mott J, Rikihisa Y. Human granulocytic ehrlichiosis agent inhibits superoxide anion generation by human neutrophils. Infect Immun. 2000;68(12):6697-703. Epub 2000/11/18. doi: 10.1128/iai.68.12.6697-6703.2000. PubMed PMID: 11083784; PubMed Central PMCID: PMCPMC97769.

611. Ostfeld R, Keesing F. The function of biodiversity in the ecology of vector-borne zoonotic diseases. Canadian Journal of Zoology. 2000;78(12):2061-78. doi: 10.1139/cjz-78-12-2061. PubMed PMID: WOS:000165450400002.

612. Oteo J. Erratum: First report of human granulocytic ehrlichiosis from southern Europe (Spain) (Emerging Infectious Diseases 6:4 (431-432)). Emerging Infectious Diseases. 2000;6(6):663.

613. Oteo J. Erratum: First report of human granulocytic ehrlichiosis from Southern Europe (Spain) (Emerging Infectious Diseases (2000) 6:4 (431)). Emerging Infectious Diseases. 2000;6(5):562.

614. Oteo JA, Blanco JR, de Artola VM, Ibarra V. First report of human granulocytic ehrlichiosis from southern Europe (Spain). Emerging Infectious Diseases. 2000;6(4):430-2. doi: 10.3201/eid0604.000425. PubMed PMID: WOS:000088660900025.

615. Palmer GH, Brown WC, Rurangirwa FR. Antigenic variation in the persistence and transmission of the ehrlichia Anaplasma marginale. Microbes and Infection. 2000;2(2):167-76. doi: 10.1016/S1286-4579(00)00271-9.

616. Perdrizet GA, Olson NH, Krause PJ, Banever GT, Spielman A, Cable RG. Babesiosis in a renal transplant recipient acquired through blood transfusion. Transplantation. 2000;70(1):205-8. Epub 2000/08/05. PubMed PMID: 10919602.

617. Pick N, Potasman I, Strenger C, Keysary A, Schwartz I. Ehrlichiosis associated vasculitis. J Intern Med. 2000;247(6):674-8. Epub 2000/07/08. doi: 10.1046/j.1365-2796.2000.00680.x. PubMed PMID: 10886489.

618. Pusterla N, Braun U, Leutenegger CM, Reusch C, Lutz H. Ehrlichiae of veterinary importance in Switzerland. Schweizer Archiv Fur Tierheilkunde. 2000;142(7):367-73. PubMed PMID: WOS:000088091600002.

619. Pusterla N, Chae JS, DeRock E, Madigan JE. One-tube PCR for the detection of the Ehrlichia phagocytophila genogroup. Vet Rec. 2000;147(1):22-3. Epub 2000/09/07. doi: 10.1136/vr.147.1.22. PubMed PMID: 10975349.

620. Pusterla N, Chang CC, Chomel BB, Chae JS, Foley JE, DeRock E, et al. Serologic and molecular evidence of Ehrlichia spp. in coyotes in California. Journal of Wildlife Diseases. 2000;36(3):494-9. doi: 10.7589/0090-3558-36.3.494. PubMed PMID: WOS:000088560500011.

621. Pusterla N, Madigan JE, Asanovich KM, Chae JS, Derock E, Leutenegger CM, et al. Experimental inoculation with human granulocytic Ehrlichia agent derived from high- and low-passage cell culture in horses. J Clin Microbiol. 2000;38(3):1276-8. Epub 2000/03/04. doi: 10.1128/jcm.38.3.1276-1278.2000. PubMed PMID: 10699041; PubMed Central PMCID: PMCPMC88606.

622. Rymaszewska A. Ehrlichiosis - A new impendence from ticks. Postepy Mikrobiologii. 2000;39(2):189-98.

623. Shibata S, Kawahara M, Rikihisa Y, Fujita H, Watanabe Y, Suto C, et al. New Ehrlichia species closely related to Ehrlichia chaffeensis isolated from Ixodes ovatus ticks in Japan. Journal of Clinical Microbiology. 2000;38(4):1331-8. doi: 10.1128/jcm.38.4.1331-1338.2000. PubMed PMID: WOS:000086302500004.

624. Shkap V, Pipano E. Culture-derived parasites in vaccination of cattle against tick-borne diseases. 2000. p. 154-71.

625. Siebinga JT, Jongejan F. Tick-borne fever (Ehrlichia phagocytophila) outbreaks on a dairy farm in Friesland, the Netherlands. Tijdschrift Voor Diergeneeskunde. 2000;125(3):74-80. PubMed PMID: WOS:000085663700002.

626. Standaert SM, Yu T, Scott MA, Childs JE, Paddock CD, Nicholson WL, et al. Primary isolation of Ehrlichia chaffeensis from patients with febrile illnesses: Clinical and molecular characteristics. Journal of Infectious Diseases. 2000;181(3):1082-8. doi: 10.1086/315346. PubMed PMID: WOS:000086344400036.

627. Suksawat J, Hegarty BC, Breitschwerdt EB. Seroprevalence of Ehrlichia canis, Ehrlichia equi, and Ehrlichia risticii in sick dogs from North Carolina and Virginia. Journal of Veterinary Internal Medicine. 2000;14(1):50-5. PubMed PMID: WOS:000085063500008.

628. Sumner JW, Storch GA, Buller RS, Liddell AM, Stockham SL, Rikihisa Y, et al. PCR amplification and phylogenetic analysis of groESL operon sequences from Ehrlichia ewingii and Ehrlichia muris. Journal of Clinical Microbiology. 2000;38(7):2746-9. doi: 10.1128/jcm.38.7.2746-2749.2000. PubMed PMID: WOS:000088042100052.

629. Tajima T, Zhi N, Lin Q, Rikihisa Y, Horowitz HW, Ralfalli J, et al. Comparison of two recombinant major outer membrane proteins of the human granulocytic ehrlichiosis agent for use in an enzyme-linked immunosorbent assay. Clin Diagn Lab Immunol. 2000;7(4):652-7. Epub 2000/07/07. doi: 10.1128/cdli.7.4.652-657.2000. PubMed PMID: 10882667; PubMed Central PMCID: PMCPMC95929.

630. Terkeltaub RA. Lyme disease 2000 - Emerging zoonoses complicate patient work-up and treatment. Geriatrics. 2000;55(7):34-+. PubMed PMID: WOS:000088194400014.

631. Tuo W, Palmer GH, McGuire TC, Zhu D, Brown WC. Interleukin-12 as an adjuvant promotes immunoglobulin G and type 1 cytokine recall responses to major surface protein 2 of the ehrlichial pathogen Anaplasma marginale. Infect Immun. 2000;68(1):270-80. Epub 1999/12/22. doi: 10.1128/iai.68.1.270-280.2000. PubMed PMID: 10603398; PubMed Central PMCID: PMCPMC97131.

632. Viseshakul N, Kamper S, Bowie MV, Barbet AF. Sequence and expression analysis of a surface antigen gene family of the rickettsia Anaplasma marginale. Gene. 2000;253(1):45-53. Epub 2000/08/05. doi: 10.1016/s0378-1119(00)00241-9. PubMed PMID: 10925201.

633. Walls JJ, Caturegli P, Bakken JS, Asanovich KM, Dumler JS. Improved sensitivity of PCR for diagnosis of human granulocytic ehrlichiosis using epank1 genes of Ehrlichia phagocytophila-group ehrlichiae. J Clin Microbiol. 2000;38(1):354-6. Epub 2000/01/05. doi: 10.1128/jcm.38.1.354-356.2000. PubMed PMID: 10618115; PubMed Central PMCID: PMCPMC88723.

634. Wensveen B. Human granulocytic ehrlichiosis due to a tick bite. Pharmaceutisch Weekblad. 2000;135(37):1398.

635. Wicki R, Sauter P, Mettler C, Natsch A, Enzler T, Pusterla N, et al. Swiss Army Survey in Switzerland to determine the prevalence of Francisella tularensis, members of the Ehrlichia phagocytophila genogroup, Borrelia burgdorferi sensu lato, and tick-borne encephalitis virus in ticks. Eur J Clin Microbiol Infect Dis. 2000;19(6):427-32. Epub 2000/08/18. doi: 10.1007/s100960000283. PubMed PMID: 10947217.

636. Wormser GP, Nadelman RB, Dattwyler RJ, Dennis DT, Shapiro ED, Steere AC, et al. Practice guidelines for the treatment of Lyme disease. Clinical Infectious Diseases. 2000;31(SUPPL. 1):S1-S14.

637. Yang D, Xiuzheng TAI, Ying QIU, Sheng YUN. Prevalence of Eperythrozoon spp. infection and congenital eperythrozoonosis in humans in Inner Mongolia, China. Epidemiology and Infection. 2000;125(2):421-6. doi: 10.1017/S0950268899004392.

638. Yoshiie K, Kim HY, Mott J, Rikihisa Y. Intracellular infection by the human granulocytic ehrlichiosis agent inhibits human neutrophil apoptosis. Infect Immun. 2000;68(3):1125-33. Epub 2000/02/26. doi: 10.1128/iai.68.3.1125-1133.2000. PubMed PMID: 10678916; PubMed Central PMCID: PMCPMC97257.

639. Yu X, McBride JW, Zhang XF, Walker DH. Characterization of the complete transcriptionally active Ehrlichia chaffeensis 28 kDa outer membrane protein multigene family. Gene. 2000;248(1-2):59-68. doi: 10.1016/s0378-1119(00)00147-5. PubMed PMID: WOS:000087111700007.

640. Yu XJ, McBride JW, Diaz CM, Walker DH. Molecular cloning and characterization of the 120-kilodalton protein gene of Ehrlichia canis and application of the recombinant 120-kilodalton protein for serodiagnosis of canine ehrlichiosis. Journal of Clinical Microbiology. 2000;38(1):369-74. PubMed PMID: WOS:000084689800066.

641. Zeidner NS, Burkot TR, Massung R, Nicholson WL, Dolan MC, Rutherford JS, et al. Transmission of the agent of human granulocytic ehrlichiosis by Ixodes spinipalpis ticks: evidence of an enzootic cycle of dual infection with Borrelia burgdorferi in Northern Colorado. J Infect Dis. 2000;182(2):616-9. Epub 2000/07/29. doi: 10.1086/315715. PubMed PMID: 10915099.

642. Zeidner NS, Dolan MC, Massung R, Piesman J, Fish D. Coinfection with Borrelia burgdorferi and the agent of human granulocytic ehrlichiosis suppresses IL-2 and IFN gamma production and promotes an IL-4 response in C3H/HeJ mice. Parasite Immunol. 2000;22(11):581-8. Epub 2000/12/16. doi: 10.1046/j.1365-3024.2000.00339.x. PubMed PMID: 11116438.

643. Akkoyunlu M, Malawista SE, Anguita J, Fikrig E. Exploitation of interleukin-8-induced neutrophil chemotaxis by the agent of human granulocytic ehrlichiosis. Infect Immun. 2001;69(9):5577-88. Epub 2001/08/14. doi: 10.1128/iai.69.9.5577-5588.2001. PubMed PMID: 11500432; PubMed Central PMCID: PMCPMC98672.

644. Alciati S, Belligni E, Del Colle S, Pugliese A. Human infections tick-transmitted. Panminerva Medica. 2001;43(4):295-304. PubMed PMID: WOS:000172325100013.

645. Alekseev AN, Dubinina HV, Semenov AV, Bolshakov CV. Evidence of ehrlichiosis agents found in ticks (Acari: Ixodidae) collected from migratory birds. J Med Entomol. 2001;38(4):471-4. Epub 2001/07/31. doi: 10.1603/0022-2585-38.4.471. PubMed PMID: 11476325.

646. Alekseev AN, Dubinina HV, Van De Pol I, Schouls LM. Identification of Ehrlichia spp, and Borrelia burgdorferi in Ixodes ticks in the Baltic regions of Russia. Journal of Clinical Microbiology. 2001;39(6):2237-42. doi: 10.1128/jcm.39.6.2237-2242.2001. PubMed PMID: WOS:000169097100032.

647. Armstrong PM, Brunet LR, Spielman A, Telford SR, 3rd. Risk of Lyme disease: perceptions of residents of a Lone Star tick-infested community. Bull World Health Organ. 2001;79(10):916-25. Epub 2001/11/06. PubMed PMID: 11693973; PubMed Central PMCID: PMCPMC2566683.

648. Bakken JS, Aguero-Rosenfeld ME, Tilden RL, Wormser GP, Horowitz HW, Raffalli JT, et al. Serial measurements of hematologic counts during the active phase of human granulocytic ehrlichiosis. Clin Infect Dis. 2001;32(6):862-70. Epub 2001/03/15. doi: 10.1086/319350. PubMed PMID: 11247709.

649. Bakken JS, Dumler JS. Proper nomenclature for the human granulocytic ehrlichiosis agent. Emerg Infect Dis. 2001;7(3):486. Epub 2001/06/01. doi: 10.3201/eid0703.010333. PubMed PMID: 11384543; PubMed Central PMCID: PMCPMC2631780.

650. Barbet AF, Yi J, Lundgren A, McEwen BR, Blouin EF, Kocan KM. Antigenic variation of Anaplasma marginale: Major surface protein 2 diversity during cyclic transmission between ticks and cattle. Infection and Immunity. 2001;69(5):3057-66. doi: 10.1128/iai.69.5.3057-3066.2001. PubMed PMID: WOS:000168158400037.

651. Belongia EA, Reed KD, Mitchell PD, Mueller-Rizner N, Vandermause M, Finkel MF, et al. Tickborne infections as a cause of nonspecific febrile illness in Wisconsin. Clin Infect Dis. 2001;32(10):1434-9. Epub 2001/04/24. doi: 10.1086/320160. PubMed PMID: 11317244.

652. Bjöersdorff A, Bergström S, Massung RF, Haemig PD, Olsen B. Ehrlichia-infected ticks on migrating birds. Emerg Infect Dis. 2001;7(5):877-9. Epub 2001/12/19. doi: 10.3201/eid0705.017517. PubMed PMID: 11747702; PubMed Central PMCID: PMCPMC2631880.

653. Borjesson DL, Simon SI, Tablin F, Barthold SW. Thrombocytopenia in a mouse model of human granulocytic ehrlichiosis. J Infect Dis. 2001;184(11):1475-9. Epub 2001/12/26. doi: 10.1086/324518. PubMed PMID: 11709793.

654. Brayton KA, Knowles DP, McGuire TC, Palmer GH. Efficient use of a small genome to generate antigenic diversity in tick-borne ehrlichial pathogens. Proc Natl Acad Sci U S A. 2001;98(7):4130-5. Epub 2001/03/29. doi: 10.1073/pnas.071056298. PubMed PMID: 11274438; PubMed Central PMCID: PMCPMC31191.

655. Brouqui P, Salvo E, Dumler JS, Raoult D. Diagnosis of granulocytic ehrlichiosis in humans by immunofluorescence assay. Clin Diagn Lab Immunol. 2001;8(1):199-202. Epub 2001/01/04. doi: 10.1128/cdli.8.1.199-202.2001. PubMed PMID: 11139221; PubMed Central PMCID: PMCPMC96036.

656. Brown WC, McGuire TC, Zhu DM, Lewin HA, Sosnow J, Palmer GH. Highly conserved regions of the immunodominant major surface protein 2 of the genogroup II ehrlichial pathogen Anaplasma marginale are rich in naturally derived CD4(+) T lymphocyte epitopes that elicit strong recall responses. Journal of Immunology. 2001;166(2):1114-24. doi: 10.4049/jimmunol.166.2.1114. PubMed PMID: WOS:000166259600052.

657. Brown WC, Palmer GH, Lewin HA, McGuire TC. CD4(+) T lymphocytes from calves immunized with Anaplasma marginale major surface protein 1 (MSP1), a heteromeric complex of MSP1a and MSP1b, preferentially recognize the MSP1a carboxyl terminus that is conserved among strains. Infection and Immunity. 2001;69(11):6853-62. doi: 10.1128/iai.69.11.6853-6862.2001. PubMed PMID: WOS:000171739200037.

658. Burkot TR, Maupin GO, Schneider BS, Denatale C, Happ CM, Rutherford JS, et al. Use of a sentinel host system to study the questing behavior of Ixodes spinipalpis and its role in the transmission of Borrelia bissettii, human granulocytic ehrlichiosis, and Babesia microti. Am J Trop Med Hyg. 2001;65(4):293-9. Epub 2001/11/06. doi: 10.4269/ajtmh.2001.65.293. PubMed PMID: 11693872.

659. Carter SE, Ravyn MD, Xu Y, Johnson RC. Molecular typing of the etiologic agent of human granulocytic ehrlichiosis. J Clin Microbiol. 2001;39(9):3398-401. Epub 2001/08/30. doi: 10.1128/jcm.39.9.3398-3401.2001. PubMed PMID: 11526190; PubMed Central PMCID: PMCPMC88358.

660. Castro MB, Nicholson WL, Kramer VL, Childs JE. Persistent infection in Neotoma fuscipes (Muridae: Sigmodontinae) with Ehrlichia phagocytophila sensu lato. Am J Trop Med Hyg. 2001;65(4):261-7. Epub 2001/11/06. doi: 10.4269/ajtmh.2001.65.261. PubMed PMID: 11693866.

661. Chang CC, Chomel BB, Kasten RW, Romano V, Tietze N. Molecular evidence of Bartonella spp. in questing adult Ixodes pacificus ticks in California. Journal of Clinical Microbiology. 2001;39(4):1221-6. doi: 10.1128/jcm.39.4.1221-1226.2001. PubMed PMID: WOS:000167946500003.

662. Chaput EK, Meek J. Spatial analysis of human granulocytic ehrlichiosis (HGE) in the 12-town area around Lyme, Connecticut. American Journal of Epidemiology. 2001;153(11):S212-S. PubMed PMID: WOS:000169006400759.

663. Chomel BB, Mac Donald KA, Kasten RW, Chang CC, Wey AC, Foley JE, et al. Aortic valve endocarditis in a dog due to Bartonella clarridgeiae. Journal of Clinical Microbiology. 2001;39(10):3548-54. doi: 10.1128/JCM.39.10.3548-3554.2001.

664. Christova I, Schouls L, van de Pol I, Park J, Panayotov S, Lefterova V, et al. High prevalence of granulocytic Ehrlichiae and Borrelia burgdorferi sensu lato in Ixodes ricinus ticks from Bulgaria. Journal of Clinical Microbiology. 2001;39(11):4172-4. doi: 10.1128/jcm.39.11.4172-4174.2001. PubMed PMID: WOS:000171934200061.

665. Comer JA, Paddock CD, Childs JE. Urban zoonoses caused by Bartonella, Coxiella, Ehrlichia, and Rickettsia species. Vector Borne Zoonotic Dis. 2001;1(2):91-118. Epub 2003/03/26. doi: 10.1089/153036601316977714. PubMed PMID: 12653141.

666. Corn JL, Nettles VF. Health protocol for translocation of free-ranging elk. Journal of Wildlife Diseases. 2001;37(3):413-26. doi: 10.7589/0090-3558-37.3.413. PubMed PMID: WOS:000170227300001.

667. Dawson JE, Paddock CD, Warner CK, Greer PW, Bartlett JH, Ewing SA, et al. Tissue diagnosis of Ehrlichia chaffeensis in patients with fatal ehrlichiosis by use of immunohistochemistry, in situ hybridization, and polymerase chain reaction. Am J Trop Med Hyg. 2001;65(5):603-9. Epub 2001/11/22. doi: 10.4269/ajtmh.2001.65.603. PubMed PMID: 11716122.

668. De Echaide ST, Knowles DP, McGuire TC, Palmer GH, Suarez CE, McElwain TF. Erratum: Detection of cattle naturally infected with Anaplasma marginale in a region of endemicity by nested PCR and a competitive enzyme-linked immunosorbent assay using recombinant major surface protein 5 (Journal of Clinical Microbiology (1998) 36:3 (777-782)). Journal of Clinical Microbiology. 2001;39(3):1207.

669. De Martino SJ, Carlyon JA, Fikrig E. Coinfection with Borrelia burgdorferi and the agent of human granulocytic ehrlichiosis. N Engl J Med. 2001;345(2):150-1. Epub 2001/07/14. doi: 10.1056/nejm200107123450218. PubMed PMID: 11450674.

670. des Vignes F, Piesman J, Heffernan R, Schulze TL, Stafford KC, Fish D. Effect of tick removal on transmission of Borrelia burgdorferi and Ehrlichia phagocytophila by Ixodes scapularis nymphs. Journal of Infectious Diseases. 2001;183(5):773-8. doi: 10.1086/318818. PubMed PMID: WOS:000166836500012.

671. Drebot MA, Lindsay R, Barker IK, Artsob H. Characterization of a human granulocytic ehrlichiosis-like agent from Ixodes scapularis, Ontario, Canada. Emerg Infect Dis. 2001;7(3):479-80. Epub 2001/06/01. doi: 10.3201/eid0703.010327. PubMed PMID: 11384537; PubMed Central PMCID: PMCPMC2631786.

672. Duh D, Petrovec M, Avsic-Zupanc T. Diversity of Babesia infecting European sheep ticks (Ixodes ricinus). Journal of Clinical Microbiology. 2001;39(9):3395-7. doi: 10.1128/jcm.39.9.3395-3397.2001. PubMed PMID: WOS:000170837500067.

673. Dumler JS, Barbet AF, Bekker CP, Dasch GA, Palmer GH, Ray SC, et al. Reorganization of genera in the families Rickettsiaceae and Anaplasmataceae in the order Rickettsiales: unification of some species of Ehrlichia with Anaplasma, Cowdria with Ehrlichia and Ehrlichia with Neorickettsia, descriptions of six new species combinations and designation of Ehrlichia equi and 'HGE agent' as subjective synonyms of Ehrlichia phagocytophila. Int J Syst Evol Microbiol. 2001;51(Pt 6):2145-65. Epub 2002/01/05. doi: 10.1099/00207713-51-6-2145. PubMed PMID: 11760958.

674. Dumler JS, Walker DH. Tick-borne ehrlichioses. Lancet. 2001:21-8. PubMed PMID: WOS:000167996700014.

675. Fernandez-Soto P, Perez-Sanchez R, Encinas-Grandes A. Molecular detection of Ehrlichia phagocytophila genogroup organisms in larvae of Neotrombicula autumnalis (Acari : Trombiculidae) captured in Spain. Journal of Parasitology. 2001;87(6):1482-3. doi: 10.2307/3285325. PubMed PMID: WOS:000172910100045.

676. Foley JE, Foley P, Madigan JE. Spatial distribution of seropositivity to the causative agent of granulocytic ehrlichiosis in dogs in California. American Journal of Veterinary Research. 2001;62(10):1599-605. doi: 10.2460/ajvr.2001.62.1599. PubMed PMID: WOS:000171270700014.

677. Gao D, Cao W, Zhang X. [Investigations on Human ehrlichia infectious people in Daxingan Mountains]. Zhonghua Liu Xing Bing Xue Za Zhi. 2001;22(2):137-41. Epub 2002/02/28. PubMed PMID: 11860865.

678. Guerrero A, Losada I, de Lucas S, Oteo JA. Ehrlichiosis infection prevalence in Spain or cross reactions. Medicina Clinica. 2001;116(8):315-. doi: 10.1016/s0025-7753(01)71809-x. PubMed PMID: WOS:000167969600010.

679. Gusa AA, Buller RS, Storch GA, Huycke MM, Machado LJ, Slater LN, et al. Identification of a p28 gene in Ehrlichia ewingii: evaluation of gene for use as a target for a species-specific PCR diagnostic assay. J Clin Microbiol. 2001;39(11):3871-6. Epub 2001/10/30. doi: 10.1128/jcm.39.11.3871-3876.2001. PubMed PMID: 11682500; PubMed Central PMCID: PMCPMC88457.

680. Hinrichsen VL, Whitworth UG, Breitschwerdt EB, Hegarty BC, Mather TN. Assessing the association between the geographic distribution of deer ticks and seropositivity rates to various tick-transmitted disease organisms in dogs. Journal of the American Veterinary Medical Association. 2001;218(7):1092-7. doi: 10.2460/javma.2001.218.1092. PubMed PMID: WOS:000167720400018.

681. Hodzic E, Borjesson DL, Feng S, Barthold SW. Acquisition dynamics of Borrelia burgdorferi and the agent of human granulocytic ehrlichiosis at the host-vector interface. Vector Borne Zoonotic Dis. 2001;1(2):149-58. Epub 2003/04/12. doi: 10.1089/153036601316977750. PubMed PMID: 12680352.

682. Hodzic E, Feng SL, Fish D, Leutenegger CM, Freet KJ, Barthold SW. Infection of mice with the agent of human granulocytic ehrlichiosis after different routes of inoculation. Journal of Infectious Diseases. 2001;183(12):1781-6. doi: 10.1086/320735. PubMed PMID: WOS:000169107000010.

683. Horowitz HW, Hsieh TC, Aguero-Rosenfeld ME, Kalantarpour F, Chowdhury I, Wormser GP, et al. Antimicrobial susceptibility of Ehrlichia phagocytophila. Antimicrob Agents Chemother. 2001;45(3):786-8. Epub 2001/02/22. doi: 10.1128/aac.45.3.786-788.2001. PubMed PMID: 11181361; PubMed Central PMCID: PMCPMC90374.

684. Hulínská D, Kurzová D, Drevová H, Votýpka J. [First detection of Ehrlichiosis detected serologically and with the polymerase chain reaction in patients with borreliosis in the Czech Republic]. Cas Lek Cesk. 2001;140(6):181-4. Epub 2001/05/12. PubMed PMID: 11347209.

685. Inokuma H, Brouqui P, Drancourt M, Raoult D. Citrate synthase gene sequence: a new tool for phylogenetic analysis and identification of Ehrlichia. J Clin Microbiol. 2001;39(9):3031-9. Epub 2001/08/30. doi: 10.1128/jcm.39.9.3031-3039.2001. PubMed PMID: 11526124; PubMed Central PMCID: PMCPMC88292.

686. Inokuma H, Nane G, Uechi T, Yonahara Y, Brouqui P, Okuda M, et al. Survey of tick infestation and tick-borne ehrlichial infection of dogs in Ishigaki Island, Japan. Journal of Veterinary Medical Science. 2001;63(11):1225-7. doi: 10.1292/jvms.63.1225. PubMed PMID: WOS:000172614900011.

687. Inokuma H, Terada Y, Kamio T, Raoult D, Brouqui P. Analysis of the 16S rRNA gene sequence of Anaplasma centrale and its phylogenetic relatedness to other ehrlichiae. Clinical and Diagnostic Laboratory Immunology. 2001;8(2):241-4. doi: 10.1128/CDLI.8.2.241-244.2001.

688. Jauron SD, Nelson CM, Fingerle V, Ravyn MD, Goodman JL, Johnson RC, et al. Host cell-specific expression of a p44 epitope by the human granulocytic ehrlichiosis agent. J Infect Dis. 2001;184(11):1445-50. Epub 2001/12/26. doi: 10.1086/324428. PubMed PMID: 11709787.

689. Jenkins A, Kristiansen BE, Allum AG, Aakre RK, Strand L, Kleveland EJ, et al. Borrelia burgdorferi sensu lato and Ehrlichia spp. in Ixodes ticks from southern Norway. Journal of Clinical Microbiology. 2001;39(10):3666-71. doi: 10.1128/jcm.39.10.3666-3671.2001. PubMed PMID: WOS:000171382600038.

690. Kafetzis DA, Maltezou HC, Constantopoulou I, Antonaki G, Liapi G, Mathioudakis I. Lack of association between Kawasaki syndrome and infection with Rickettsia conorii, Rickettsia typhi, Coxiella burnetii or Ehrlichia phagocytophila group. Pediatr Infect Dis J. 2001;20(7):703-6. Epub 2001/07/24. doi: 10.1097/00006454-200107000-00012. PubMed PMID: 11465844.

691. Kristiansen BE, Jenkins A, Tveten Y, Karsten B, Line Ø, Bjöersdorff A. [Human granulocytic ehrlichiosis in Norway]. Tidsskr Nor Laegeforen. 2001;121(7):805-6. Epub 2001/04/17. PubMed PMID: 11301703.

692. Lane RS, Foley JE, Eisen L, Lennette ET, Peot MA. Acarologic risk of exposure to emerging tick-borne bacterial pathogens in a semirural community in northern California. Vector Borne Zoonotic Dis. 2001;1(3):197-210. Epub 2003/03/26. doi: 10.1089/153036601753552567. PubMed PMID: 12653148.

693. Leal M, Noda A, Reyna-Bello A, Casas B, Précigout E, Aso PM, et al. Erratum: Identification and characterization of corpuscular, soluble and secreted antigens of a Venezuelan isolate of Anaplasma marginale (Veterinary Parasitology (2000) 94 (1-15) PII: S030440170000371X). Veterinary Parasitology. 2001;96(4):329. doi: 10.1016/S0304-4017(01)00375-2.

694. Levin ML, Fish D. Interference Between the Agents of Lyme Disease and Human Granulocytic Ehrlichiosis in a Natural Reservoir Host. Vector-Borne and Zoonotic Diseases. 2001;1(2):139-+. doi: 10.1089/153036601316977741. PubMed PMID: WOS:000210777800005.

695. Li JSY, Yager E, Reilly M, Freeman C, Reddy GR, Reilly AA, et al. Outer membrane protein-specific monoclonal antibodies protect SCID mice from fatal infection by the obligate intracellular bacterial pathogen Ehrlichia chaffeensis. Journal of Immunology. 2001;166(3):1855-62. PubMed PMID: WOS:000166622700054.

696. Liang FT, Bowers LC, Philipp MT. C-terminal invariable domain of VlsE is immunodominant but its antigenicity is scarcely conserved among strains of Lyme disease spirochetes. Infection and Immunity. 2001;69(5):3224-31. doi: 10.1128/iai.69.5.3224-3231.2001. PubMed PMID: WOS:000168158400057.

697. Lodes MJ, Mohamath R, Reynolds LD, McNeill P, Kolbert CP, Bruinsma ES, et al. Serodiagnosis of human granulocytic ehrlichiosis by using novel combinations of immunoreactive recombinant proteins. J Clin Microbiol. 2001;39(7):2466-76. Epub 2001/06/28. doi: 10.1128/jcm.39.7.2466-2476.2001. PubMed PMID: 11427556; PubMed Central PMCID: PMCPMC88172.

698. Lotric-Furlan S, Petrovec M, Avsic-Zupanc T, Nicholson WL, Sumner JW, Childs JE, et al. Prospective assessment of the etiology of acute febrile illness after a tick bite in Slovenia. Clin Infect Dis. 2001;33(4):503-10. Epub 2001/07/20. doi: 10.1086/322586. PubMed PMID: 11462187.

699. Magnarelli L, Ijdo J, Wu C, Fikrig E. Recombinant protein-44-based class-specific enzyme-linked immunosorbent assays for serologic diagnosis of human granulocytic ehrlichiosis. Eur J Clin Microbiol Infect Dis. 2001;20(7):482-5. Epub 2001/09/20. doi: 10.1007/s100960100542. PubMed PMID: 11561804.

700. Magnarelli LA, Ijdo JW, Van Andel AE, Wu C, Oliver JH, Jr., Fikrig E. Reactivity of serum samples of dogs and horses tested by use of class-specific recombinant-based enzyme-linked immunosorbent assays for detection of granulocytic ehrlichiosis. Am J Vet Res. 2001;62(9):1365-9. Epub 2001/09/19. doi: 10.2460/ajvr.2001.62.1365. PubMed PMID: 11560261.

701. Maloo SH, Thorpe W, Kioo G, Ngumi P, Rowlands GJ, Perry BD. Seroprevalences of vector-transmitted infections of small-holder dairy cattle in coastal Kenya. Preventive Veterinary Medicine. 2001;52(1):1-16. doi: 10.1016/S0167-5877(01)00234-3.

702. Martin ME, Caspersen K, Dumler JS. Immunopathology and ehrlichial propagation are regulated by interferon-gamma and interleukin-10 in a murine model of human granulocytic ehrlichiosis. Am J Pathol. 2001;158(5):1881-8. Epub 2001/05/05. doi: 10.1016/s0002-9440(10)64145-4. PubMed PMID: 11337387; PubMed Central PMCID: PMCPMC1891945.

703. Maurin M, Abergel C, Raoult D. DNA gyrase-mediated natural resistance to fluoroquinolones in Ehrlichia spp. Antimicrob Agents Chemother. 2001;45(7):2098-105. Epub 2001/06/16. doi: 10.1128/aac.45.7.2098-2105.2001. PubMed PMID: 11408229; PubMed Central PMCID: PMCPMC90606.

704. Meeus PF, Barbet AF. Ingenious gene generation. Trends Microbiol. 2001;9(8):353-5; discussion 5-6. Epub 2001/08/22. doi: 10.1016/s0966-842x(01)02112-6. PubMed PMID: 11514195.

705. Müllegger RR. Clinical aspects and diagnosis of erythema migrans and borrelial lymphocytoma. Acta Dermatovenerologica Alpina, Panonica et Adriatica. 2001;10(4):152-8.

706. Oteo JA, Gil H, Barral M, Perez A, Jimenez S, Blanco JR, et al. Presence of granulocytic ehrlichia in ticks and serological evidence of human infection in La Rioia, Spain. Epidemiology and Infection. 2001;127(2):353-8. doi: 10.1017/s0950268801005878. PubMed PMID: WOS:000172246300021.

707. Padgett KA, Lane RS. Life cycle of Ixodes pacificus (Acari : Ixodidae): Timing of developmental processes under field and laboratory conditions. Journal of Medical Entomology. 2001;38(5):684-93. doi: 10.1603/0022-2585-38.5.684. PubMed PMID: WOS:000171279100012.

708. Palmer GH, Rurangirwa FR, McElwain TF. Strain composition of the Ehrlichia Anaplasma marginale within persistently infected cattle, a mammalian reservoir for tick transmission. Journal of Clinical Microbiology. 2001;39(2):631-5. doi: 10.1128/jcm.39.2.631-635.2001. PubMed PMID: WOS:000166776100036.

709. Parola P, Didier R. Ticks and tickborne bacterial diseases in humans: An emerging infectious threat. Clinical Infectious Diseases. 2001;32(6):897-928. doi: 10.1086/319347. PubMed PMID: WOS:000167461500008.

710. Parola P, Raoult D. [Molecular tools in the epidemiology of tick-borne bacterial diseases]. Ann Biol Clin (Paris). 2001;59(2):177-82. Epub 2001/04/03. PubMed PMID: 11282521.

711. Parola P, Raoult D. Tick-borne bacterial diseases emerging in Europe. Clin Microbiol Infect. 2001;7(2):80-3. Epub 2001/04/12. doi: 10.1046/j.1469-0691.2001.00200.x. PubMed PMID: 11298147.

712. Paskewitz SM, Vandermause M, Belongia EA, Kazmierczak JJ. Ixodes scapularis (Acari : Ixodidae): Abundance and rate of infection with Borrelia burgdorferi in four state parks in Wisconsin. Journal of Medical Entomology. 2001;38(1):33-8. doi: 10.1603/0022-2585-38.1.33. PubMed PMID: WOS:000166456500006.

713. Poland GA. Prevention of Lyme disease: A review of the evidence. Mayo Clinic Proceedings. 2001;76(7):713-24. doi: 10.4065/76.7.713. PubMed PMID: WOS:000169576600008.

714. Pott-Opitz U. Ehrlichiose in a domestic cat. A case report. Tieraerztliche Praxis Ausgabe Kleintiere Heimtiere. 2001;29(3):198-202. PubMed PMID: WOS:000169378800009.

715. Pusterla N, Anderson RJ, House JK, Pusterla JB, DeRock E, Madigan JE. Susceptibility of cattle to infection with Ehrlichia equi and the agent of human granulocytic ehrlichiosis. Journal of the American Veterinary Medical Association. 2001;218(7):1160-+. doi: 10.2460/javma.2001.218.1160. PubMed PMID: WOS:000167720400030.

716. Ravyn MD, Kodner CB, Carter SE, Jarnefeld JL, Johnson RC. Isolation of the etiologic agent of human granulocytic ehrlichiosis from the white-footed mouse (Peromyscus leucopus). J Clin Microbiol. 2001;39(1):335-8. Epub 2001/01/04. doi: 10.1128/jcm.39.1.335-338.2001. PubMed PMID: 11136794; PubMed Central PMCID: PMCPMC87725.

717. Ryan R, Krause PJ, Radolf J, Freeman K, Spielman A, Lenz R, et al. Diagnosis of babesiosis using an immunoblot serologic test. Clin Diagn Lab Immunol. 2001;8(6):1177-80. Epub 2001/11/01. doi: 10.1128/cdli.8.6.1177-1180.2001. PubMed PMID: 11687460; PubMed Central PMCID: PMCPMC96246.

718. Scott JD, Fernando K, Banerjee SN, Durden LA, Byrne SK, Banerjee M, et al. Birds disperse ixodid (Acari : Ixodidae) and Borrelia burgdorferi-infected ticks in Canada. Journal of Medical Entomology. 2001;38(4):493-500. doi: 10.1603/0022-2585-38.4.493. PubMed PMID: WOS:000169823600005.

719. Semenov AV, Alekseev AN, Dubinina EV, Kaufmann U, Jensen RM. [Detection of the genotypic heterogeneity of Ixodes persulcatus Schulze (Acari: Ixodidae) of the North-West region of Russia and characteristics of distribution of tick-borne pathogens causing Lyme disease and Ehrlichia infections in various genotypes]. Med Parazitol (Mosk). 2001;(3):11-5. Epub 2001/10/30. PubMed PMID: 11680364.

720. Shadick NA, Liang MH, Phillips CB, Fossel K, Kuntz KM. The cost-effectiveness of vaccination against Lyme disease. Archives of Internal Medicine. 2001;161(4):554-61. doi: 10.1001/archinte.161.4.554. PubMed PMID: WOS:000167075800008.

721. Shaw SE, Day MJ, Birtles RJ, Breitschwerdt EB. Tick-borne infectious diseases of dogs. Trends in Parasitology. 2001;17(2):74-80. doi: 10.1016/S1471-4922(00)01856-0.

722. Shoda LKM, Kegerreis KA, Suarez CE, Mwangi W, Knowles DP, Brown WC. Immunostimulatory CpG-modified plasmid DNA enhances IL-12, TNF-alpha, and NO production by bovine macrophages. Journal of Leukocyte Biology. 2001;70(1):103-12. PubMed PMID: WOS:000169799600014.

723. Skarphédinsson S, Søgaard P, Pedersen C. Seroprevalence of human granulocytic ehrlichiosis in high-risk groups in Denmark. Scand J Infect Dis. 2001;33(3):206-10. Epub 2001/04/17. doi: 10.1080/00365540151060860. PubMed PMID: 11303811.

724. Skotarczak B, Rymaszewska A. [Prevelance of the etiological agent of human ehrlichiosis (HGE) in ticks from west-north Poland]. Wiad Parazytol. 2001;47(1):95-101. Epub 2006/08/08. PubMed PMID: 16888958.

725. Sotomayor EA, Popov VL, Feng HM, Walker DH, Olano JP. Animal model of fatal human monocytotropic ehrlichiosis. American Journal of Pathology. 2001;158(2):757-69. doi: 10.1016/s0002-9440(10)64018-7. PubMed PMID: WOS:000166925900045.

726. Stromdahl EY, Evans SR, O'Brien JJ, Gutierrez AG. Prevalence of infection in ticks submitted to the human tick test kit program of the U.S. Army Center for Health Promotion and Preventive Medicine. J Med Entomol. 2001;38(1):67-74. Epub 2001/03/28. doi: 10.1603/0022-2585-38.1.67. PubMed PMID: 11268694.

727. Stuen S, Bergstrom K. The effect of two different oxytetracycline treatments in experimental Ehrlichia phagocytophila infected lambs. Acta Veterinaria Scandinavica. 2001;42(3):339-46. doi: 10.1186/1751-0147-42-339. PubMed PMID: WOS:000173749100004.

728. Stuen S, Bergstrom K. Persistence of Ehrlichia phagocytophila infection in two age groups of lambs. Acta Veterinaria Scandinavica. 2001;42(4):453-8. doi: 10.1186/1751-0147-42-453. PubMed PMID: WOS:000174421200004.

729. Stuen S, Engvall EO, van de Pol I, Schouls LM. Granulocytic ehrlichiosis in a roe deer calf in Norway. Journal of Wildlife Diseases. 2001;37(3):614-6. doi: 10.7589/0090-3558-37.3.614. PubMed PMID: WOS:000170227300021.

730. Tan HP, Dumler JS, Maley WR, Klein AS, Burdick JF, Poordad FF, et al. Human monocytic ehrlichiosis: An emerging pathogen in transplantation. Transplantation. 2001;71(11):1678-80. doi: 10.1097/00007890-200106150-00030. PubMed PMID: WOS:000169420900030.

731. Thomas V, Anguita J, Barthold SW, Fikrig E. Coinfection with Borrelia burgdorferi and the agent of human granulocytic ehrlichiosis alters murine immune responses, pathogen burden, and severity of Lyme arthritis. Infect Immun. 2001;69(5):3359-71. Epub 2001/04/09. doi: 10.1128/iai.69.5.3359-3371.2001. PubMed PMID: 11292759; PubMed Central PMCID: PMCPMC98295.

732. Thompson C, Spielman A, Krause PJ. Coinfecting deer-associated zoonoses: Lyme disease, babesiosis, and ehrlichiosis. Clin Infect Dis. 2001;33(5):676-85. Epub 2001/08/07. doi: 10.1086/322681. PubMed PMID: 11486290.

733. Unver A, Felek S, Paddock CD, Zhi N, Horowitz HW, Wormser GP, et al. Western blot analysis of sera reactive to human monocytic ehrlichiosis and human granulocytic ehrlichiosis agents. J Clin Microbiol. 2001;39(11):3982-6. Epub 2001/10/30. doi: 10.1128/jcm.39.11.3982-3986.2001. PubMed PMID: 11682518; PubMed Central PMCID: PMCPMC88475.

734. van der Merwe D, Swan GE, Botha CJ. Use of ethnoveterinary medicinal plants in cattle by Setswana-speaking people in the Madikwe area of the North West Province of South Africa. Journal of the South African Veterinary Association. 2001;72(4):189-96. doi: 10.4102/jsava.v72i4.651.

735. Van Solingen RM, Evans J. Lyme disease. Curr Opin Rheumatol. 2001;13(4):293-9. Epub 2001/09/14. doi: 10.1097/00002281-200107000-00009. PubMed PMID: 11555731.

736. Walker AR, Alberdi MP, Urquhart KA, Rose H. Risk factors in habitats of the tick Ixodes ricinus influencing human exposure to Ehrlichia phagocytophila bacteria. Med Vet Entomol. 2001;15(1):40-9. Epub 2001/04/12. doi: 10.1046/j.1365-2915.2001.00271.x. PubMed PMID: 11297100.

737. Weinberg GA. Laboratory diagnosis of ehrlichiosis and babesiosis. Pediatr Infect Dis J. 2001;20(4):435-7. Epub 2001/05/03. doi: 10.1097/00006454-200104000-00012. PubMed PMID: 11332670.

738. Wittesjö B, Bjöersdorff A, Eliasson I, Berglund J. First long-term study of the seroresponse to the agent of human granulocytic ehrlichiosis among residents of a tick-endemic area of Sweden. Eur J Clin Microbiol Infect Dis. 2001;20(3):173-8. Epub 2001/05/12. doi: 10.1007/s100960100463. PubMed PMID: 11347666.

739. Woessner R, Gaertner BC, Grauer MT, Weber K, Mueller-Lantzsch N, Hunfeld KP, et al. Incidence and prevalence of infection with human granulocytic ehrlichiosis agent in Germany. A prospective study in young healthy subjects. Infection. 2001;29(5):271-3. Epub 2001/11/02. doi: 10.1007/s15010-001-2005-x. PubMed PMID: 11688905.

740. Yawetz S, Mark EJ, Aquino SLH, Colvin RB. A 76-year-old man with fever, dyspnea, pulmonary infiltrates, pleural effusions, and confusion - Ehrlichiosis, human granulocytic form. New England Journal of Medicine. 2001;345(22):1627-34. doi: 10.1056/NEJMcpc3756515. PubMed PMID: WOS:000172383900008.

741. Yu XJ, Zhang XF, McBride JW, Zhang Y, Walker DH. Phylogenetic relationships of Anaplasma marginale and 'Ehrlichia platys' to other Ehrlichia species determined by GroEL amino acid sequences. Int J Syst Evol Microbiol. 2001;51(Pt 3):1143-6. Epub 2001/06/21. doi: 10.1099/00207713-51-3-1143. PubMed PMID: 11414267.

742. Zhang Y, Shoda LKM, Brayton KA, Estes DM, Palmer GH, Brown WC. Induction of interleukin-6 and interleukin-12 in bovine B lymphocytes, monocytes, and macrophages by a CpG oligodeoxynucleotide (ODN 2059) containing the GTCGTT motif. Journal of Interferon and Cytokine Research. 2001;21(10):871-81. doi: 10.1089/107999001753238123. PubMed PMID: WOS:000172099900013.

743. The veterinary community. ARS announces veterinary research award winners. American Journal of Veterinary Research. 2002;63(4):474.

744. Notification that new names and new combinations have appeared in volume 51, part 6, of the IJSEM. International Journal of Systematic and Evolutionary Microbiology. 2002;52(1):5-6. doi: 10.1099/00207713-52-1-5.

745. Aga E, Katschinski DM, van Zandbergen G, Laufs H, Hansen B, Muller K, et al. Inhibition of the spontaneous apoptosis of neutrophil granulocytes by the intracellular parasite Leishmania major. Journal of Immunology. 2002;169(2):898-905. doi: 10.4049/jimmunol.169.2.898. PubMed PMID: WOS:000176753500033.

746. Aguero-Rosenfeld ME. Diagnosis of human granulocytic ehrlichiosis: state of the art. Vector Borne Zoonotic Dis. 2002;2(4):233-9. Epub 2003/06/14. doi: 10.1089/153036602321653815. PubMed PMID: 12804164.

747. Aguero-Rosenfeld ME, Donnarumma L, Zentmaier L, Jacob J, Frey M, Noto R, et al. Seroprevalence of antibodies that react with Anaplasma phagocytophila, the agent of human granulocytic ehrlichiosis, in different populations in Westchester County, New York. J Clin Microbiol. 2002;40(7):2612-5. Epub 2002/06/29. doi: 10.1128/jcm.40.7.2612-2615.2002. PubMed PMID: 12089287; PubMed Central PMCID: PMCPMC120546.

748. Akkoyunlu M. Elimination of erythrocytes from blood prior to DNA extraction improves the sensitivity of anaplasma phagocytophila PCR. Scand J Infect Dis. 2002;34(10):788. Epub 2002/12/13. doi: 10.1080/00365540210147949. PubMed PMID: 12477341.

749. Alexiou Daniel S, Manika K, Arvanitidou M, Diza E, Symeonidis N, Antoniadis A. Serologic evidence of human granulocytic ehrlichiosis, Greece [1]. Emerging Infectious Diseases. 2002;8(6):643-4. doi: 10.3201/eid0806.010500.

750. Bakken JS, Haller I, Riddell D, Walls JJ, Dumler JS. The serological response of patients infected with the agent of human granulocytic ehrlichiosis. Clin Infect Dis. 2002;34(1):22-7. Epub 2001/12/04. doi: 10.1086/323811. PubMed PMID: 11731941.

751. Bakken JS, Haller I, Riddell D, Walls JJ, Dumler JS. Erratum: The serological response of patients infected with the agent of human granulocytic ehrlichiosis. (Clinical Infections Diseases (January 2002) 34 (22-27). Clinical Infectious Diseases. 2002;34(4):561.

752. Belongia EA. Epidemiology and impact of coinfections acquired from Ixodes ticks. Vector Borne Zoonotic Dis. 2002;2(4):265-73. Epub 2003/06/14. doi: 10.1089/153036602321653851. PubMed PMID: 12804168.

753. Bjöersdorff A, Bagert B, Massung RF, Gusa A, Eliasson I. Isolation and characterization of two European strains of Ehrlichia phagocytophila of equine origin. Clin Diagn Lab Immunol. 2002;9(2):341-3. Epub 2002/03/05. doi: 10.1128/cdli.9.2.341-343.2002. PubMed PMID: 11874874; PubMed Central PMCID: PMCPMC119922.

754. Blanco JR, Oteo JA. Human granulocytic ehrlichiosis in Europe. Clin Microbiol Infect. 2002;8(12):763-72. Epub 2003/01/10. doi: 10.1046/j.1469-0691.2002.00557.x. PubMed PMID: 12519349.

755. Borjesson DL, Barthold SW. The mouse as a model for investigation of human granulocytic ehrlichiosis: current knowledge and future directions. Comp Med. 2002;52(5):403-13. Epub 2002/10/31. PubMed PMID: 12405632.

756. Borjesson DL, Simon SI, Hodzic E, Ballantyne CM, Barthold SW. Kinetics of CD11b/CD18 up-regulation during infection with the agent of human granulocytic ehrlichiosis in mice. Laboratory Investigation. 2002;82(3):303-11. doi: 10.1038/labinvest.3780424. PubMed PMID: WOS:000174619500008.

757. Brayton KA, Palmer GH, Lundgren A, Yi J, Barbet AF. Antigenic variation of Anaplasma marginale msp2 occurs by combinatorial gene conversion. Mol Microbiol. 2002;43(5):1151-9. Epub 2002/03/29. doi: 10.1046/j.1365-2958.2002.02792.x. PubMed PMID: 11918803.

758. Brown WC, McGuire TC, Mwangi W, Kegerreis KA, Macmillan H, Lewin HA, et al. Major histocompatibility complex class II DR-restricted memory CD4(+) T lymphocytes recognize conserved immunodominant epitopes of Anaplasma marginale major surface protein 1a. Infection and Immunity. 2002;70(10):5521-32. doi: 10.1128/iai.70.10.5521-5532.2002. PubMed PMID: WOS:000178125500022.

759. Carlyon JA, Chan WT, Galán J, Roos D, Fikrig E. Repression of rac2 mRNA expression by Anaplasma phagocytophila is essential to the inhibition of superoxide production and bacterial proliferation. J Immunol. 2002;169(12):7009-18. Epub 2002/12/10. doi: 10.4049/jimmunol.169.12.7009. PubMed PMID: 12471136.

760. Caspersen K, Park JH, Patil S, Dumler JS. Genetic variability and stability of Anaplasma phagocytophila msp2 (p44). Infect Immun. 2002;70(3):1230-4. Epub 2002/02/21. doi: 10.1128/iai.70.3.1230-1234.2002. PubMed PMID: 11854205; PubMed Central PMCID: PMCPMC127805.

761. Chaput EK, Meek JI, Heimer R. Spatial analysis of human granulocytic ehrlichiosis near Lyme, Connecticut. Emerg Infect Dis. 2002;8(9):943-8. Epub 2002/08/27. doi: 10.3201/eid0809.020103. PubMed PMID: 12194771; PubMed Central PMCID: PMCPMC2732548.

762. Choi E. Tularemia and Q fever. Medical Clinics of North America. 2002;86(2):393-416. doi: 10.1016/S0025-7125(03)00094-4.

763. Collins NE, Allsopp MTEP, Allsopp BA. Molecular diagnosis of theileriosis and heartwater in bovines in Africa. Transactions of the Royal Society of Tropical Medicine and Hygiene. 2002;96(SUPPL. 1):S217. doi: 10.1016/s0035-9203(02)90079-9.

764. Daniel SA, Manika K, Arvanitidou M, Diza E, Symeonidis N, Antoniadis A. Serologic evidence of human granulocytic ehrlichiosis, Greece. Emerg Infect Dis. 2002;8(6):643-4. Epub 2002/05/25. doi: 10.3201/eid0806.010500. PubMed PMID: 12023926; PubMed Central PMCID: PMCPMC2738483.

765. Daniels TJ, Battaly GR, Liveris D, Falco RC, Schwartz I. Avian reservoirs of the agent of human granulocytic ehrlichiosis? Emerg Infect Dis. 2002;8(12):1524-5. Epub 2002/12/25. doi: 10.3201/eid0812.010527. PubMed PMID: 12498679; PubMed Central PMCID: PMCPMC2738500.

766. De la Fuente J, Van Den Bussche RA, Garcia-Garcia JC, Rodríguez SD, García MA, Guglielmone AA, et al. Phylogeography of New World isolates of Anaplasma marginale based on major surface protein sequences. Veterinary Microbiology. 2002;88(3):275-85. doi: 10.1016/S0378-1135(02)00122-0.

767. DeNatale CE, Burkot TR, Schneider BS, Zeidner NS. Novel potential reservoirs for Borrelia sp. and the agent of human granulocytic ehrlichiosis in Colorado. J Wildl Dis. 2002;38(2):478-82. Epub 2002/06/01. doi: 10.7589/0090-3558-38.2.478. PubMed PMID: 12038153.

768. Donovan BJ, Weber DJ, Rublein JC, Raasch RH. Treatment of tick-borne diseases. Ann Pharmacother. 2002;36(10):1590-7. Epub 2002/09/24. doi: 10.1345/aph.1C089. PubMed PMID: 12243610.

769. Eisen L, Eisen RJ, Lane RS. Seasonal activity patterns of Ixodes pacificus nymphs in relation to climatic conditions. Medical and Veterinary Entomology. 2002;16(3):235-44. doi: 10.1046/j.1365-2915.2002.00372.x. PubMed PMID: WOS:000177775900002.

770. Fang QQ, Mixson TR, Hughes M, Dunham B, Sapp J. Prevalence of the agent of human granulocytic ehrlichiosis in Ixodes scapularis (Acari: Ixodidae) in the coastal southeastern United States. J Med Entomol. 2002;39(2):251-5. Epub 2002/04/05. doi: 10.1603/0022-2585-39.2.251. PubMed PMID: 11931023.

771. Foley JE, Kramer V, Weber D. Experimental infection of dusky-footed wood rats (Neotoma fuscipes) with Ehrlichia phagocytophila sensu lato. Journal of Wildlife Diseases. 2002;38(1):194-8. doi: 10.7589/0090-3558-38.1.194. PubMed PMID: WOS:000173689100027.

772. Ganta RR, Wilkerson MJ, Cheng CM, Rokey AM, Chapes SK. Persistent Ehrlichia chaffeensis infection occurs in the absence of functional major histocompatibility complex class II genes. Infection and Immunity. 2002;70(1):380-8. doi: 10.1128/iai.70.1.380-388.2002. PubMed PMID: WOS:000172847600049.

773. Getchell JK, Vatta AF, Motswatswe PW, Krecek RC, Moerane R, Pell AN, et al. Raising livestock in resource-poor communities of the North West Province of South Africa--a participatory rural appraisal study. J S Afr Vet Assoc. 2002;73(4):177-84. Epub 2003/04/01. doi: 10.4102/jsava.v73i4.583. PubMed PMID: 12665130.

774. Gonçalves Ruiz PM, Passos LMF, Martins MS, Patarroyo JH, Ribeiro MFB. Antigenic characterization of morphologically distinct Anaplasma marginale isolates using a panel of monoclonal antibodies. Veterinary Parasitology. 2002;107(1-2):169-77. doi: 10.1016/S0304-4017(02)00106-1.

775. Govaerts M, Verhaert P, Jongejan F, Goddeeris BM. Characterisation of the 33 kDa piroplasm surface antigen of Theileria orientalis/sergenti/buffeli isolates from West Java, Indonesia. Veterinary Parasitology. 2002;104(2):103-17. doi: 10.1016/S0304-4017(01)00621-5.

776. Groen J, Koraka P, Nur YA, Avsic-Zupanc T, Goessens WH, Ott A, et al. Serologic evidence of ehrlichiosis among humans and wild animals in The Netherlands. Eur J Clin Microbiol Infect Dis. 2002;21(1):46-9. Epub 2002/03/28. doi: 10.1007/s10096-001-0659-z. PubMed PMID: 11915850.

777. Grzeszczuk A, Stańczak J, Kubica-Biernat B. Serological and molecular evidence of human granulocytic ehrlichiosis focus in the Bialłowieża Primeval forest (Puszcza Bialłowieska), northeastern Poland. European Journal of Clinical Microbiology and Infectious Diseases. 2002;21(1):6-11. doi: 10.1007/s10096-001-0649-1.

778. Guillaume B, Heyman P, Lafontaine S, Vandenvelde C, Delmée M, Bigaignon G. Seroprevalence of human granulocytic ehrlichiosis infection in Belgium. Eur J Clin Microbiol Infect Dis. 2002;21(5):397-400. Epub 2002/06/20. doi: 10.1007/s10096-002-0720-6. PubMed PMID: 12072927.

779. Heo EJ, Park JH, Koo JR, Park MS, Park MY, Dumler JS, et al. Serologic and molecular detection of Ehrlichia chaffeensis and Anaplasma phagocytophila (human granulocytic ehrlichiosis agent) in Korean patients. J Clin Microbiol. 2002;40(8):3082-5. Epub 2002/08/01. doi: 10.1128/jcm.40.8.3082-3085.2002. PubMed PMID: 12149387; PubMed Central PMCID: PMCPMC120627.

780. Ijdo JW, Adelson-Mitty J. Erratum: "Granulocytic ehrlichiosis in an HIV-positive patient" (Infections in Medicine (2002) vol. 19 (39-42)). Infections in Medicine. 2002;19(3):105.

781. Inokuma H, Fujii K, Matsumoto K, Okuda M, Nakagome K, Kosugi R, et al. Demonstration of Anaplasma (Ehrlichia) platys inclusions in peripheral blood platelets of a dog in Japan. Veterinary Parasitology. 2002;110(1-2):145-52. doi: 10.1016/S0304-4017(02)00289-3.

782. Inokuma H, Fujii K, Okuda M, Onishi T, Beaufils JP, Raoult D, et al. Determination of the nucleotide sequences of heat shock operon groESL and the citrate synthase gene (gltA) of Anaplasma (Ehrlichia) platys for phylogenetic and diagnostic studies. Clinical and Diagnostic Laboratory Immunology. 2002;9(5):1132-6. doi: 10.1128/CDLI.9.5.1132-1136.2002.

783. Jackson CA, Lovrich SD, Agger WA, Callister SM. Reassessment of a midwestern Lyme disease focus for Borrelia burgdorferi and the human granulocytic ehrlichiosis agent. Journal of Clinical Microbiology. 2002;40(6):2070-3. doi: 10.1128/jcm.40.6.2070-2073.2002. PubMed PMID: WOS:000176159200028.

784. JW IJ, Wu C, Telford SR, 3rd, Fikrig E. Differential expression of the p44 gene family in the agent of human granulocytic ehrlichiosis. Infect Immun. 2002;70(9):5295-8. Epub 2002/08/17. doi: 10.1128/iai.70.9.5295-5298.2002. PubMed PMID: 12183586; PubMed Central PMCID: PMCPMC128253.

785. Kager L, Kastner U, Gadner H, Stane G. Human granulocytic ehrlichiosis: A new tick-borne disease. Padiatrische Praxis. 2002;61(3):455-61.

786. Kano FS, Vidotto O, Pacheco RC, Vidotto MC. Antigenic characterization of Anaplasma marginale isolates from different regions of Brazil. Veterinary Microbiology. 2002;87(2):131-8. doi: 10.1016/S0378-1135(02)00051-2.

787. Kelly DJ, Richards AL, Temenak J, Strickman D, Dasch GA. The past and present threat of rickettsial diseases to military medicine and international public health. Clinical Infectious Diseases. 2002;34(SUPPL. 4):S145-S69.

788. Kim HY, Mott J, Zhi N, Tajima T, Rikihisa Y. Cytokine gene expression by peripheral blood leukocytes in horses experimentally infected with Anaplasma phagocytophila. Clinical and Diagnostic Laboratory Immunology. 2002;9(5):1079-84. doi: 10.1128/cdli.9.5.1079-1084.2002. PubMed PMID: WOS:000177897700022.

789. Kim HY, Rikihisa Y. Roles of p38 mitogen-activated protein kinase, NF-kappaB, and protein kinase C in proinflammatory cytokine mRNA expression by human peripheral blood leukocytes, monocytes, and neutrophils in response to Anaplasma phagocytophila. Infect Immun. 2002;70(8):4132-41. Epub 2002/07/16. doi: 10.1128/iai.70.8.4132-4141.2002. PubMed PMID: 12117921; PubMed Central PMCID: PMCPMC128199.

790. Kondrusik M, Puciło K, Świerzbińska R, Pancewicz S, Zajkowska J, Grygorczuk S, et al. Ehrlichia chaffeensis antibodies prevalence among patients bitten by ticks. Polski Merkuriusz Lekarski. 2002;13(78):462-4.

791. Kroft SH. Infectious diseases manifested in the peripheral blood. Clinics in Laboratory Medicine. 2002;22(1):253-+. doi: 10.1016/s0272-2712(03)00074-x. PubMed PMID: WOS:000174935800013.

792. Lantos PM, Krause PJ. Ehrlichiosis in children. Semin Pediatr Infect Dis. 2002;13(4):249-56. Epub 2002/12/20. doi: 10.1053/spid.2002.127200. PubMed PMID: 12491230.

793. Layfield D, Guilfoile P. The prevalence of Borrelia burgdorferi (Spirochaetales: Spirochaetaceae) and the agent of human granulocytic ehrlichiosis (Rickettsiaceae: Ehrlichieae) in Ixodes scapularis (Acari:Ixodidae) collected during 1998 and 1999 from Minnesota. Journal of Medical Entomology. 2002;39(1):218-20. doi: 10.1603/0022-2585-39.1.218.

794. Leiby DA, Chung AP, Cable RG, Trouern-Trend J, McCullough J, Homer MJ, et al. Relationship between tick bites and the seroprevalence of Babesia microti and Anaplasma phagocytophila (previously Ehrlichia sp.) in blood donors. Transfusion. 2002;42(12):1585-91. Epub 2002/12/11. doi: 10.1046/j.1537-2995.2002.00251.x. PubMed PMID: 12473139.

795. Leutenegger CM, Pusterla N, Wicki R, Lutz H. New molecular tools in the diagnosis of tick-borne diseases. Schweizer Archiv Fur Tierheilkunde. 2002;144(8):395-404. doi: 10.1024/0036-7281.144.8.395. PubMed PMID: WOS:000177745400002.

796. Levin ML, Nicholson WL, Massung RF, Sumner JW, Fish D. Comparison of the Reservoir Competence of Medium-Sized Mammals and Peromyscus leucopus for Anaplasma phagocytophilum in Connecticut. Vector-Borne and Zoonotic Diseases. 2002;2(3):125-+. doi: 10.1089/15303660260613693. PubMed PMID: WOS:000210780400002.

797. Lew AE, Bock RE, Minchin CM, Masaka S. Erratum: A msp1α polymerase chain reaction assay for specific detection and differentiation of Anaplasma marginale isolates (Veterinary Microbiology (2002) 86 (325-335) PII: S0378113502000172). Veterinary Microbiology. 2002;87(4):365-6. doi: 10.1016/S0378-1135(02)00069-X.

798. Lin Q, Zhi N, Ohashi N, Horowitz HW, Aguero-Rosenfeld ME, Raffalli J, et al. Analysis of sequences and loci of p44 homologs expressed by Anaplasma phagocytophila in acutely infected patients. J Clin Microbiol. 2002;40(8):2981-8. Epub 2002/08/01. doi: 10.1128/jcm.40.8.2981-2988.2002. PubMed PMID: 12149362; PubMed Central PMCID: PMCPMC120678.

799. Liz JS, Sumner JW, Pfister K, Brossard M. PCR detection and serological evidence of granulocytic ehrlichial infection in roe deer (Capreolus capreolus) and chamois (Rupicapra rupicapra). Journal of Clinical Microbiology. 2002;40(3):892-7. doi: 10.1128/JCM.40.3.892-897.2002.

800. Long SW, Zhang XF, Qi H, Standaert S, Walker DH, Yu XJ. Antigenic variation of Ehrlichia chaffeensis resulting from differential expression of the 28-kilodalton protein gene family. Infection and Immunity. 2002;70(4):1824-31. doi: 10.1128/iai.70.4.1824-1831.2002. PubMed PMID: WOS:000174573200017.

801. Lotric-Furlan S, Petrovec M, Avsic-Zupanc T, Logar M, Strle F. Epidemiological, clinical and laboratory distinction between human granulocytic ehrlichiosis and the initial phase of tick-borne encephalitis. Wien Klin Wochenschr. 2002;114(13-14):636-40. Epub 2002/11/09. PubMed PMID: 12422617.

802. Magnarelli LA, Ijdo JW, Sherman BA, Bushmich SL, Levy SA, Fikrig E. Antibodies to granulocytic ehrlichiae in cattle from Connecticut. Journal of Medical Microbiology. 2002;51(4):326-31. doi: 10.1099/0022-1317-51-4-326. PubMed PMID: WOS:000174521100008.

803. Magnarelli LA, Lawrenz M, Norris SJ, Fikrig E. Comparative reactivity of human sera to recombinant VIsE and other Borrelia burgdorferi antigens in class-specific enzyme-linked immunosorbent assays for Lyme borreliosis. Journal of Medical Microbiology. 2002;51(8):649-55. doi: 10.1099/0022-1317-51-8-649.

804. Massung RF, Lee K, Mauel M, Gusa A. Characterization of the rRNA genes of Ehrlichia chaffeensis and Anaplasma phagocytophila. DNA Cell Biol. 2002;21(8):587-96. Epub 2002/09/07. doi: 10.1089/104454902320308960. PubMed PMID: 12215262.

805. Massung RF, Mauel MJ, Owens JH, Allan N, Courtney JW, Stafford KC, 3rd, et al. Genetic variants of Ehrlichia phagocytophila, Rhode Island and Connecticut. Emerg Infect Dis. 2002;8(5):467-72. Epub 2002/05/09. doi: 10.3201/eid0805.010251. PubMed PMID: 11996680; PubMed Central PMCID: PMCPMC3369764.

806. Mbati PA, Hlatshwayo M, Mtshali MS, Mogaswane KR, De Waal TD, Dipeolu OO. Ticks and tick-borne diseases of livestock belonging to resource-poor farmers in the eastern Free State of South Africa. Exp Appl Acarol. 2002;28(1-4):217-24. Epub 2003/10/23. doi: 10.1023/a:1025306701803. PubMed PMID: 14570134.

807. Morozova OV, Dobrotvorsky AK, Livanova NN, Tkachev SE, Bakhvalova VN, Beklemishev AB, et al. PCR detection of Borrelia burgdorferi sensu lato, tick-borne encephalitis virus, and the human granulocytic ehrlichiosis agent in Ixodes persulcatus ticks from Western Siberia, Russia. J Clin Microbiol. 2002;40(10):3802-4. Epub 2002/10/02. doi: 10.1128/jcm.40.10.3802-3804.2002. PubMed PMID: 12354885; PubMed Central PMCID: PMCPMC130855.

808. Mott J, Rikihisa Y, Tsunawaki S. Effects of Anaplasma phagocytophila on NADPH oxidase components in human neutrophils and HL-60 cells. Infect Immun. 2002;70(3):1359-66. Epub 2002/02/21. doi: 10.1128/iai.70.3.1359-1366.2002. PubMed PMID: 11854221; PubMed Central PMCID: PMCPMC127795.

809. Mwangi W, Brown WC, Lewin HA, Howard CJ, Hope JC, Baszler TV, et al. DNA-encoded fetal liver tyrosine kinase 3 ligand and granulocyte macrophage-colony-stimulating factor increase dendritic cell recruitment to the inoculation site and enhance antigen-specific CD4+ T cell responses induced by DNA vaccination of outbred animals. Journal of Immunology. 2002;169(7):3837-46. doi: 10.4049/jimmunol.169.7.3837.

810. Neer TM, Breitschwerdt EB, Greene RT, Lappin MR. Consensus statement on ehrlichial disease of small animals from the infectious disease study group of the ACVIM. Journal of Veterinary Internal Medicine. 2002;16(3):309-15. doi: 10.1892/0891-6640(2002)016<0309:Csoedo>2.3.Co;2. PubMed PMID: WOS:000175601700016.

811. Oehme R, Hartelt K, Backe H, Brockmann S, Kimmig P. Foci of tick-borne diseases in southwest Germany. Int J Med Microbiol. 2002;291 Suppl 33:22-9. Epub 2002/07/27. doi: 10.1016/s1438-4221(02)80005-4. PubMed PMID: 12141751.

812. Ogden NH, Casey ANJ, French NP, Woldehiwet Z. A review of studies on the transmission of Anaplasma phagocytophilum from sheep: implications for the force of infection in endemic cycles. Experimental and Applied Acarology. 2002;28(1):195-202. doi: 10.1023/a:1025394315915. PubMed PMID: WOS:000184875900019.

813. Ohashi N, Zhi N, Lin Q, Rikihisa Y. Characterization and transcriptional analysis of gene clusters for a type IV secretion machinery in human granulocytic and monocytic ehrlichiosis agents. Infect Immun. 2002;70(4):2128-38. Epub 2002/03/16. doi: 10.1128/iai.70.4.2128-2138.2002. PubMed PMID: 11895979; PubMed Central PMCID: PMCPMC127848.

814. Olano JP, Walker DH. Human ehrlichioses. Med Clin North Am. 2002;86(2):375-92. Epub 2002/05/02. doi: 10.1016/s0025-7125(03)00093-2. PubMed PMID: 11982308.

815. Olano JP, Walker DH. Human ehrlichioses: Diagnostic challenges and therapeutic recommendations. Infections in Medicine. 2002;19(7):318-25. PubMed PMID: WOS:000176803400008.

816. Palmer GH. The highest priority: what microbial genomes are telling us about immunity. Vet Immunol Immunopathol. 2002;85(1-2):1-8. Epub 2002/02/28. doi: 10.1016/s0165-2427(01)00415-9. PubMed PMID: 11867162.

817. Pantanowitz L, Telford SR, Cannon ME. Tick-borne diseases in transfusion medicine. Transfusion Medicine. 2002;12(2):85-106. doi: 10.1046/j.1365-3148.2002.00358.x. PubMed PMID: WOS:000175234400001.

818. Petrovec M, Bidovec A, Sumner JW, Nicholson WL, Childs JE, Avsic-Zupanc T. Infection with Anaplasma phagocytophila in cervids from Slovenia: evidence of two genotypic lineages. Wien Klin Wochenschr. 2002;114(13-14):641-7. Epub 2002/11/09. PubMed PMID: 12422618.

819. Preziosi DE, Cohn LA. The increasingly complicated story of Ehrlichia. Compendium on Continuing Education for the Practicing Veterinarian. 2002;24(4):277-88. PubMed PMID: WOS:000175149500001.

820. Pusterla N, Chae JS, Kimsey RB, Berger Pusterla J, DeRock E, Dumler JS, et al. Transmission of Anaplasma phagocytophila (human granulocytic ehrlichiosis agent) in horses using experimentally infected ticks (Ixodes scapularis). J Vet Med B Infect Dis Vet Public Health. 2002;49(10):484-8. Epub 2002/12/18. doi: 10.1046/j.1439-0450.2002.00598.x. PubMed PMID: 12485358.

821. Ramsey AH, Belongia EA, Gale CM, Davis JP. Outcomes of treated human granulocytic ehrlichiosis cases. Emerg Infect Dis. 2002;8(4):398-401. Epub 2002/04/25. doi: 10.3201/eid0804.010222. PubMed PMID: 11971774; PubMed Central PMCID: PMCPMC2730236.

822. Rurangirwa FR, Brayton KA, McGuire TC, Knowles DP, Palmer GH. Conservation of the unique rickettsial rRNA gene arrangement in Anaplasma. Int J Syst Evol Microbiol. 2002;52(Pt 4):1405-9. Epub 2002/08/01. doi: 10.1099/00207713-52-4-1405. PubMed PMID: 12148657.

823. Safdar N, Love RB, Maki DG. Severe Ehrlichia chaffeensis infection in a lung transplant recipient: A review of ehrlichiosis in the immunocompromised patient. Emerging Infectious Diseases. 2002;8(3):320-3. doi: 10.3201/eid0803.010249. PubMed PMID: WOS:000174434800015.

824. Santino I, del Piano M, Sessa R, Favia G, Iori A. Detection of four Borrelia burgdorferi genospecies and first report of human granulocytic ehrlichiosis agent in Ixodes ricinus ticks collected in central Italy. Epidemiol Infect. 2002;129(1):93-7. Epub 2002/09/05. doi: 10.1017/s0950268802007057. PubMed PMID: 12211602; PubMed Central PMCID: PMCPMC2869880.

825. Santino I, Grillo R, Nicoletti M, Santapaola D, Speziale D, Sessa R, et al. Prevalence of IgG antibodies against Borrelia Burgdorferi s.l. and Ehrlichia Phagocytophila in sera of patients presenting symptoms of Lyme disease in a central region of Italy. Int J Immunopathol Pharmacol. 2002;15(3):245-8. Epub 2003/02/11. doi: 10.1177/039463200201500313. PubMed PMID: 12575927.

826. Schultz SM, Nicholson WL, Comer JA, Childs JE, Humphreys JG. Serologic evidence of infection with granulocytic ehrlichiae in black bears in Pennsylvania. Journal of Wildlife Diseases. 2002;38(1):47-53. doi: 10.7589/0090-3558-38.1.47. PubMed PMID: WOS:000173689100007.

827. Shkap V, Molad T, Brayton KA, Brown WC, Palmer GH. Expression of major surface protein 2 variants with conserved T-cell epitopes in Anaplasma centrale vaccinates. Infection and Immunity. 2002;70(2):642-8. doi: 10.1128/IAI.70.2.642-648.2002.

828. Shkap V, Molad T, Fish L, Palmer G. Detection of the Anaplasma centrale vaccine strain and specific differentiation from anaplasma marginale in vaccinated and infected cattle. Parasitology Research. 2002;88(6):546-52. doi: 10.1007/s00436-002-0612-9.

829. Sigal LH. Vaccination for Lyme disease: cost-effectiveness versus cost and value. Arthritis and Rheumatism. 2002;46(6):1439-42. doi: 10.1002/art.10283. PubMed PMID: WOS:000176199200003.

830. Skotarczak B, Wodecka B, Cichocka A. Coexistence DNA of Borrelia burgdorferi sensu lato and Babesia microti in Ixodes ricinus ticks from north-western Poland. Annals of Agricultural and Environmental Medicine. 2002;9(1):25-8. PubMed PMID: WOS:000176473800003.

831. Snapp KR, Heitzig CE, Marth JD, Goodman JL. Structural modifications of PSGL-1 required for adhesion and entry of the causative agent of human granulocytic ehrlichiosis (HGE). Glycobiology. 2002;12(10):646-. PubMed PMID: WOS:000178448200024.

832. Stańczak J, Racewicz M, Kruminis-Lozowska W, Kubica-Biernat B. Coinfection of Ixodes ricinus (Acari: Ixodidae) in northern Poland with the agents of Lyme borreliosis (LB) and human granulocytic ehrlichiosis (HGE). Int J Med Microbiol. 2002;291 Suppl 33:198-201. Epub 2002/07/27. doi: 10.1016/s1438-4221(02)80045-5. PubMed PMID: 12141746.

833. Stich RW, Rikihisa Y, Ewing SA, Needham GR, Grover DL, Jittapalapong S. Detection of Ehrlichia canis in canine carrier blood and in individual experimentally infected ticks with a p30-based PCR assay. Journal of Clinical Microbiology. 2002;40(2):540-6. doi: 10.1128/jcm.40.2.540-546.2002. PubMed PMID: WOS:000173731900034.

834. Stuen S, Van de Pol I, Bergstrom K, Sehouls LA. Identification of Anaplasma phagocytophila (formerly Ehrlichia phagocytophila) variants in blood from sheep in Norway. Journal of Clinical Microbiology. 2002;40(9):3192-7. doi: 10.1128/jcm.40.9.3192-3197.2002. PubMed PMID: WOS:000177829900013.

835. Szentgyorgyi L, Kapiller Z. Human babesiosis. Magyar Allatorvosok Lapja. 2002;124(9):563-70. PubMed PMID: WOS:000178656300008.

836. Tarello W. Cutaneous lesions in dogs with Dirofilaria (Nochtiella) repens infestation and concurrent tick-borne transmitted diseases. Veterinary Dermatology. 2002;13(5):267-74. doi: 10.1046/j.1365-3164.2002.00305.x. PubMed PMID: WOS:000178337900007.

837. Telford 3rd SR, Korenberg EI, Goethert HK, Kovalevskii IV, Gorelova NB, Spielman A. Detection of natural foci of babesiosis and granulocytic ehrlichiosis in Russia. Zhurnal mikrobiologii, epidemiologii, i immunobiologii. 2002;(6):21-5.

838. Wang GQ, Ojaimi C, Wu HY, Saksenberg V, Iyer R, Liveris D, et al. Disease severity in a murine model of Lyme borreliosis is associated with the genotype of the infecting Borrelia burgdorferi sensu stricto strain. Journal of Infectious Diseases. 2002;186(6):782-91. doi: 10.1086/343043. PubMed PMID: WOS:000177694800008.

839. Wang T, Malawista SE, Pal U, Grey M, Meek J, Akkoyunlu M, et al. Superoxide anion production during Anaplasma phagocytophila infection. J Infect Dis. 2002;186(2):274-80. Epub 2002/07/23. doi: 10.1086/341451. PubMed PMID: 12134266.

840. Wen BH, Jian R, Zhang YZ, Chen R. Simultaneous detection of Anaplasma marginale and a new Ehrlichia species closely related to Ehrlichia chaffeensis by sequence analyses of 16S ribosomal DNA in Boophilus microplus ticks from Tibet. Journal of Clinical Microbiology. 2002;40(9):3286-90. doi: 10.1128/jcm.40.9.3286-3290.2002. PubMed PMID: WOS:000177829900028.

841. Westerman EL. Chills, fever, and myalgia in a farmer. Infections in Medicine. 2002;19(6):252.

842. Whist SK, Storset AK, Larsen HJS. Functions of neutrophils in sheep experimentally infected with Ehrlichia phagocytophila. Veterinary Immunology and Immunopathology. 2002;86(3-4):183-93. doi: 10.1016/S0165-2427(02)00038-7.

843. Woldehiwet Z, Horrocks BK, Scaife H, Ross G, Munderloh UG, Bown K, et al. Cultivation of an ovine strain of Ehrlichia phagocytophila in tick cell cultures. Journal of Comparative Pathology. 2002;127(2-3):142-9. doi: 10.1053/jcpa.2002.0574. PubMed PMID: WOS:000178368000008.

844. Wormser GP. Impressions of the IX Conference on Lyme Borreliosis and Other Tick-Borne Diseases, August 18-22, 2002. Vector-Borne and Zoonotic Diseases. 2002;2(4):201-7. doi: 10.1089/153036602321653770. PubMed PMID: WOS:000210780800001.

845. Yabsley MJ, Varela AS, Tate CM, Dugan VG, Stallknecht DE, Little SE, et al. Ehrlichia ewingii infection in white-tailed deer (Odocoileus virginianus). Emerg Infect Dis. 2002;8(7):668-71. Epub 2002/07/04. doi: 10.3201/eid0807.020018. PubMed PMID: 12095432; PubMed Central PMCID: PMCPMC2730327.

846. Zaidi SA, Singer C. Gastrointestinal and hepatic manifestations of tickborne diseases in the United States. Clinical Infectious Diseases. 2002;34(9):1206-12. doi: 10.1086/339871. PubMed PMID: WOS:000174901600007.

847. Zeman P. [Comparison of extraction methods for detection of Ehrlichia DNA in whole blood using PCR]. Epidemiol Mikrobiol Imunol. 2002;51(4):143-7. Epub 2003/01/21. PubMed PMID: 12532899.

848. Zeman P. Comparison of some extraction methods for the detection of ehrlichian DNA in full blood by means of PCR. Epidemiologie, Mikrobiologie, Imunologie. 2002;51(4):143-7.

849. Zeman P, Cinatl J, Kabickova H. Sodium valproate facilitates the propagation of granulocytic ehrlichiae (Anaplasma phagocytophilum) in HL-60 cells. Exp Appl Acarol. 2002;28(1-4):203-8. Epub 2003/10/23. doi: 10.1023/a:1025398432754. PubMed PMID: 14570132.

850. Zeman P, Pazdiora P, Cinatl J. HGE antibodies in sera of patients with TBE in the Czech Republic. Int J Med Microbiol. 2002;291 Suppl 33:190-3. Epub 2002/07/27. doi: 10.1016/s1438-4221(02)80042-x. PubMed PMID: 12141745.

851. Zeman P, Pazdiora P, Rébl K, Činátl J. Manifestation of antibodies against granulocytic ehrlichiae in the population of western and central Bohemia. Epidemiologie, Mikrobiologie, Imunologie. 2002;51(1):13-8.

852. Zhi N, Ohashi N, Rikihisa Y. Activation of a p44 pseudogene in Anaplasma phagocytophila by bacterial RNA splicing: a novel mechanism for post-transcriptional regulation of a multigene family encoding immunodominant major outer membrane proteins. Mol Microbiol. 2002;46(1):135-45. Epub 2002/10/09. doi: 10.1046/j.1365-2958.2002.03143.x. PubMed PMID: 12366837.

853. Zhi N, Ohashi N, Tajima T, Mott J, Stich RW, Grover D, et al. Erratum: Transcript heterogeneity of the p44 multigene family in a human granulocytic ehrlichiosis agent transmitted by ticks (Infection and Immunity (2002) 70:3 (1175-1184)). Infection and Immunity. 2002;70(8):4754. doi: 10.1128/IAI.70.8.4754.2002.

854. Zhi N, Ohashi N, Tajima T, Mott J, Stich RW, Grover D, et al. Transcript heterogeneity of the p44 multigene family in a human granulocytic ehrlichiosis agent transmitted by ticks. Infection and Immunity. 2002;70(3):1175-84. doi: 10.1128/IAI.70.3.1175-1184.2002.

855. Zhi N, Ohashi N, Tajima T, Mott J, Stich RW, Grover D, et al. Transcript heterogeneity of the p44 multigene family in a human granulocytic ehrlichiosis agent transmitted by ticks (vol 70, pg 1177, 2002). Infection and Immunity. 2002;70(8):4754-. doi: 10.1128/IAI.70.8.4754.2002-a. PubMed PMID: WOS:000176909400097.

856. Aguero-Rosenfeld ME. Laboratory aspects of tick-borne diseases: lyme, human granulocytic ehrlichiosis and babesiosis. Mt Sinai J Med. 2003;70(3):197-206. Epub 2003/05/24. PubMed PMID: 12764539.

857. Allen LAH. Mechanisms of pathogenesis: Evasion of killing by polymorphonuclear leukocytes. Microbes and Infection. 2003;5(14):1329-35. doi: 10.1016/j.micinf.2003.09.011.

858. Arens MQ, Liddell AM, Buening G, Gaudreault-Keener M, Sumner JW, Comer JA, et al. Detection of Ehrlichia spp. in the blood of wild white-tailed deer in Missouri by PCR assay and serologic analysis. Journal of Clinical Microbiology. 2003;41(3):1263-5. doi: 10.1128/jcm.41.3.1263-1265.2003. PubMed PMID: WOS:000181616500055.

859. Arnez M, Luznik-Bufon T, Avsic-Zupanc T, Ruzic-Sabljic E, Petrovec M, Lotric-Furlan S, et al. Causes of febrile illnesses after a tick bite in Slovenian children. Pediatr Infect Dis J. 2003;22(12):1078-83. Epub 2003/12/23. doi: 10.1097/01.inf.0000101477.90756.50. PubMed PMID: 14688569.

860. Arnez M, Luznik-Bufon T, Avsic-Zupanc T, Ruzić-Sabljić E, Petrovec M, Lotric-Furlan S, et al. Etiology of tick-borne febrile illnesses in Slovenian children. Ann N Y Acad Sci. 2003;990:353-4. Epub 2003/07/16. doi: 10.1111/j.1749-6632.2003.tb07388.x. PubMed PMID: 12860651.

861. Barbet AF, Meeus PF, Bélanger M, Bowie MV, Yi J, Lundgren AM, et al. Expression of multiple outer membrane protein sequence variants from a single genomic locus of Anaplasma phagocytophilum. Infect Immun. 2003;71(4):1706-18. Epub 2003/03/26. doi: 10.1128/iai.71.4.1706-1718.2003. PubMed PMID: 12654783; PubMed Central PMCID: PMCPMC152091.

862. Borjesson DL, Simon SI, Hodzic E, DeCock HE, Ballantyne CM, Barthold SW. Roles of neutrophil beta 2 integrins in kinetics of bacteremia, extravasation, and tick acquisition of Anaplasma phagocytophila in mice. Blood. 2003;101(8):3257-64. Epub 2002/12/14. doi: 10.1182/blood-2002-04-1019. PubMed PMID: 12480703.

863. Bown KJ, Begon M, Bennett M, Woldehiwet Z, Ogden NH. Seasonal dynamics of Anaplasma phagocytophila in a rodent-tick (Ixodes trianguliceps) system, United Kingdom. Emerging Infectious Diseases. 2003;9(1):63-70. PubMed PMID: WOS:000180503300010.

864. Brouqui P, Sanogo YO, Caruso G, Merola F, Raoult D. Candidatus Ehrlichia walkerii - A new Ehrlichia detected in Ixodes ricinus tick collected from asymptomatic humans in Northern Italy. In: Hechemy KE, AvsicZupanc T, Childs JE, Raoult DA, editors. Rickettsiology: Present and Future Directions. Annals of the New York Academy of Sciences. 9902003. p. 134-40.

865. Brown WC, Brayton KA, Styer CM, Palmer GH. The hypervariable region of Anaplasma marginale major surface protein 2 (MSP2) contains multiple immunodominant CD4(+) T lymphocyte epitopes that elicit variant-specific proliferative and IFN-gamma responses in MSP2 vaccinates. Journal of Immunology. 2003;170(7):3790-8. doi: 10.4049/jimmunol.170.7.3790. PubMed PMID: WOS:000181754100042.

866. Brownstein JS, Holford TR, Fish D. A climate-based model predicts the spatial distribution of the Lyme disease vector Ixodes scapularis in the United States. Environmental Health Perspectives. 2003;111(9):1152-7. doi: 10.1289/ehp.6052. PubMed PMID: WOS:000184992700023.

867. Bunnell JE, Price SD, Das A, Shields TM, Glass GE. Geographic information systems and spatial analysis of adult Ixodes scapularis (Acari: Ixodidae) in the Middle Atlantic region of the U.S.A. J Med Entomol. 2003;40(4):570-6. Epub 2003/12/19. doi: 10.1603/0022-2585-40.4.570. PubMed PMID: 14680128.

868. Cable RG, Leiby DA. Risk and prevention of transfusion-transmitted babesiosis and other tick-borne diseases. Current Opinion in Hematology. 2003;10(6):405-11. doi: 10.1097/00062752-200311000-00002. PubMed PMID: WOS:000186154100002.

869. Cao WC, Zhao QM, Zhang PH, Yang H, Wu XM, Wen BH, et al. Prevalence of Anaplasma phagocytophila and Borrelia burgdorferi in Ixodes persulcatus ticks from northeastern China. Am J Trop Med Hyg. 2003;68(5):547-50. Epub 2003/06/19. doi: 10.4269/ajtmh.2003.68.547. PubMed PMID: 12812342.

870. Carlyon JA, Akkoyunlu M, Xia L, Yago T, Wang T, Cummings RD, et al. Murine neutrophils require α 1,3-fucosylation but not PSGL-1 for productive infection with Anaplasma phagocytophilum. Blood. 2003;102(9):3387-95. doi: 10.1182/blood-2003-02-0621.

871. Carlyon JA, Fikrig E. Invasion and survival strategies of Anaplasma phagocytophilum. Cell Microbiol. 2003;5(11):743-54. Epub 2003/10/09. doi: 10.1046/j.1462-5822.2003.00323.x. PubMed PMID: 14531890.

872. Chae JS, Kim CM, Kim EH, Hur EJ, Klein TA, Kang TK, et al. Molecular epidemiological study for tick-borne disease (Ehrlichia and Anaplasma spp.) surveillance at selected U.S. military training sites/installations in Korea. Ann N Y Acad Sci. 2003;990:118-25. Epub 2003/07/16. doi: 10.1111/j.1749-6632.2003.tb07349.x. PubMed PMID: 12860612.

873. Choi KS, Dumler JS. Early induction and late abrogation of respiratory burst in A-phagocytophilum - Infected neutrophils. In: Hechemy KE, AvsicZupanc T, Childs JE, Raoult DA, editors. Rickettsiology: Present and Future Directions. Annals of the New York Academy of Sciences. 9902003. p. 488-93.

874. Choi KS, Garyu J, Park J, Dumler JS. Diminished adhesion of Anaplasma phagocytophilum-infected neutrophils to endothelial cells is associated with reduced expression of leukocyte surface selectin. Infect Immun. 2003;71(8):4586-94. Epub 2003/07/23. doi: 10.1128/iai.71.8.4586-4594.2003. PubMed PMID: 12874338; PubMed Central PMCID: PMCPMC166008.

875. Chomel BB. Control and prevention of emerging zoonoses. Journal of Veterinary Medical Education. 2003;30(2):145-7. doi: 10.3138/jvme.30.2.145.

876. Chomel BB, Wey AC, Kasten RW. Isolation of Bartonella washoensis from a dog with mitral valve endocarditis. Journal of Clinical Microbiology. 2003;41(11):5327-32. doi: 10.1128/jcm.41.11.5327-5332.2003. PubMed PMID: WOS:000186665500080.

877. Christova I, Van De Pol J, Yazar S, Velo E, Schouls L. Identification of Borrelia burgdorferi sensu lato, Anaplasma and Ehrlichia species, and spotted fever group Rickettsiae in ticks from Southeastern Europe. Eur J Clin Microbiol Infect Dis. 2003;22(9):535-42. Epub 2003/08/26. doi: 10.1007/s10096-003-0988-1. PubMed PMID: 12938010.

878. Ciceroni L, Bartoloni A, Leoncinic F, Ciarrocchi S, Pinto A, Favia G, et al. Risk of tick-borne bacterial diseases in humans in the Florence area, Tuscany. In: Hechemy KE, AvsicZupanc T, Childs JE, Raoult DA, editors. Rickettsiology: Present and Future Directions. Annals of the New York Academy of Sciences. 9902003. p. 346-9.

879. Cohn LA. Ehrlichiosis and related infections. Veterinary Clinics of North America - Small Animal Practice. 2003;33(4):863-84. doi: 10.1016/S0195-5616(03)00031-7.

880. Courtney JW, Dryden RL, Montgomery J, Schneider BS, Smith G, Massung RF. Molecular characterization of Anaplasma phagocytophilum and Borrelia burgdorferi in Ixodes scapularis ticks from Pennsylvania. Journal of Clinical Microbiology. 2003;41(4):1569-73. doi: 10.1128/JCM.41.4.1569-1573.2003.

881. Courtney JW, Dryden RL, Wyleto P, Schneider BS, Massung RF. Characterization of Anaplasma phagocytophila and Borrelia burgdorferi genotypes in Ixodes scapularis ticks from Pennsylvania. 2003. p. 131-3.

882. Courtney JW, Massung RF. Multiplex Taqman PCR assay for rapid detection of Anaplasma phagocytophila and Borrelia burgdorferi. Ann N Y Acad Sci. 2003;990:369-70. Epub 2003/07/16. doi: 10.1111/j.1749-6632.2003.tb07393.x. PubMed PMID: 12860656.

883. Cousins JK, Bono MJ. Clinician's guide to tick-borne disease. Emergency Medicine (00136654). 2003;35(7):50-4. PubMed PMID: 106728812. Language: English. Entry Date: 20040430. Revision Date: 20150711. Publication Type: Journal Article. Journal Subset: Allied Health.

884. De la Fuente J, Van Den Bussche RA, Prado TM, Kocan KM. Anaplasma marginale msp1α genotypes evolved under positive selection pressure but are not markers for geographic isolates. Journal of Clinical Microbiology. 2003;41(4):1609-16. doi: 10.1128/JCM.41.4.1609-1616.2003.

885. Derdakova M, Halanova M, Stanko M, Stefancikova A, Cislakova L, Pet'ko B. Molecular evidence for Anaplasma phagocytophilium and Borrelia burgdorferi sensu lato in Ixodes ricinus ticks from Eastern Slovakia. Annals of Agricultural and Environmental Medicine. 2003;10(2):269-71. PubMed PMID: WOS:000187536600023.

886. Dumler JS. Molecular methods for ehrlichiosis and Lyme disease. Clin Lab Med. 2003;23(4):867-84, vi. Epub 2004/01/09. doi: 10.1016/s0272-2712(03)00080-5. PubMed PMID: 14711096.

887. Dumler JS, Asanovich KM, Bakken JS. Analysis of genetic identity of North American Anaplasma phagocytophilum strains by pulsed-field ge1 electrophoresis. Journal of Clinical Microbiology. 2003;41(7):3392-4. doi: 10.1128/JCM.41.7.3392-3394.2003.

888. Eisen L, Dolan MC, Piesman J, Lane RS. Vector competence of Ixodes pacificus and I-spinipalpis (Acari : ixodidae), and reservoir competence of the dusky-footed woodrat (Neotoma fuscipes) and the deer mouse (Peromyscus maniculatus), for Borrelia bissettii. Journal of Medical Entomology. 2003;40(3):311-20. doi: 10.1603/0022-2585-40.3.311. PubMed PMID: WOS:000183019100011.

889. Fenollar F, La Scola B, Inokuma H, Dumler JS, Taylor MJ, Raoult D. Culture and phenotypic characterization of a Wolbachia pipientis isolate. Journal of Clinical Microbiology. 2003;41(12):5434-41. doi: 10.1128/jcm.41.12.5434-5441.2003. PubMed PMID: WOS:000187228800012.

890. Foley JE, Leutenegger CM, Dumler JS, Pedersen NC, Madigan JE. Evidence for modulated immune response to Anaplasma phagocytophila sensu lato in cats with FIV-induced immunosuppression. Comparative Immunology, Microbiology and Infectious Diseases. 2003;26(2):103-13. doi: 10.1016/S0147-9571(02)00023-1.

891. Franz JK, Krause A. Lyme disease (Lyme borreliosis). Best Practice & Research in Clinical Rheumatology. 2003;17(2):241-64. doi: 10.1016/s1521-6942(02)00129-8. PubMed PMID: WOS:000183663700006.

892. Fumarola D, Fumarola L, Brandonisio O. Human Ehrlichiosis, an emerging tick-borne transmitted zoonosis also in human pathology. Igiene Moderna. 2003;119(4):229-36.

893. Futse JE, Ueti MW, Knowles Jr DP, Palmer GH. Erratum: Transmission of Anaplasma marginale by Boophilus microplus: Retention of Vector Competence in the Absence of Vector-Pathogen Interaction (Journal of Clinical Microbiology (2003) 41:8 (3829-3834)). Journal of Clinical Microbiology. 2003;41(11):5354. doi: 10.1128/JCM.41.11.5354.2003.

894. Gardner SL, Holman RC, Krebs JW, Berkelman R, Childs JE. National surveillance for the human ehrlichioses in the United States, 1997-2001, and proposed methods for evaluation of data quality. Ann N Y Acad Sci. 2003;990:80-9. Epub 2003/07/16. doi: 10.1111/j.1749-6632.2003.tb07341.x. PubMed PMID: 12860604.

895. Goddard J, Sumner JW, Nicholson WL, Paddock CD, Shen J, Piesman J. Survey of ticks collected in Mississippi for Rickettsia, Ehrlichia, and Borrelia species. J Vector Ecol. 2003;28(2):184-9. Epub 2004/01/13. PubMed PMID: 14714667.

896. Goethert HK, Telford Iii SR. Enzootic transmission of Babesia divergens among cottontail rabbits on Nantucket Island, Massachusetts. American Journal of Tropical Medicine and Hygiene. 2003;69(5):455-60. doi: 10.4269/ajtmh.2003.69.455.

897. Goethert HK, Telford Iii SR. Enzootic transmission of the agent of human granulocytic ehrlichiosis among cottontail rabbits. American Journal of Tropical Medicine and Hygiene. 2003;68(6):633-7. doi: 10.4269/ajtmh.2003.68.633.

898. Goethert HK, Telford Iii SR. Enzootic transmission of Anaplasma bovis in Nantucket cottontail rabbits. Journal of Clinical Microbiology. 2003;41(8):3744-7. doi: 10.1128/JCM.41.8.3744-3747.2003.

899. Heyman P, Cochez C, Bigaignon G, Guillaume B, Zizi M, Vandenvelde C. Human Granulocytic Ehrlichiosis in Belgium: an underestimated cause of disease. J Infect. 2003;47(2):129-32. Epub 2003/07/16. doi: 10.1016/s0163-4453(03)00056-2. PubMed PMID: 12860146.

900. Hildebrandt A, Schmidt KH, Wilske B, Dorn W, Straube E, Fingerle V. Prevalence of four species of Borrelia burgdorferi sensu lato and coinfection with Anaplasma phagocytophila in Ixodes ricinus ticks in central Germany. Eur J Clin Microbiol Infect Dis. 2003;22(6):364-7. Epub 2003/06/05. doi: 10.1007/s10096-003-0926-2. PubMed PMID: 12783276.

901. Hoelzle LE, Adelt D, Hoelzle K, Heinritzi K, Wittenbrink MM. Development of a diagnostic PCR assay based on novel DNA sequences for the detection of Mycoplasma suis (Eperythrozoon suis) in porcine blood. Veterinary Microbiology. 2003;93(3):185-96. doi: 10.1016/S0378-1135(03)00040-3.

902. Holden K, Boothby JT, Anand S, Massung RF. Detection of Borrelia burgdorferi, Ehrlichia chaffeensis, and Anaplasma phagocytophilum in ticks (Acari: Ixodidae) from a coastal region of California. J Med Entomol. 2003;40(4):534-9. Epub 2003/12/19. doi: 10.1603/0022-2585-40.4.534. PubMed PMID: 14680123.

903. Hulínská D, Votýpka J, Plch J, Bojar M, Honegr K, Šnelerová M. Sequential analysis and real-time lightcycler polymerase chain reaction in the identification of Borrelia and Ehrlichia spp. Klinicka Mikrobiologie a Infekcni Lekarstvi. 2003;9(5):253-8.

904. Inokuma H, Brouqui P, Dumler JS, Raoult D. Serotyping isolates of Anaplasma phagocytophilum by using monoclonal antibodies. Clin Diagn Lab Immunol. 2003;10(5):969-72. Epub 2003/09/11. doi: 10.1128/cdli.10.5.969-972.2003. PubMed PMID: 12965936; PubMed Central PMCID: PMCPMC193879.

905. Kager L, Kastner U, Gadner H, Stanek G. Human granulocytic ehrlichiosis. A new tickborne disease. Internistische Praxis. 2003;43(2):287-93.

906. Kim CM, Kim MS, Park MS, Park JH, Chae JS. Identification of Ehrlichia chaffeensis, Anaplasma phagocytophilum, and A-bovis in Haemaphysalis longicornis and Ixodes persulcatus ticks from Korea. Vector-Borne and Zoonotic Diseases. 2003;3(1):17-26. doi: 10.1089/153036603765627424. PubMed PMID: WOS:000220169800003.

907. Kocan KM, de la Fuente J, Guglielmone AA, Melendez RD. Antigens and alternatives for control of Anaplasma marginale infection in cattle. Clinical Microbiology Reviews. 2003;16(4):698-+. doi: 10.1128/cmr.16.4.698-712.2003. PubMed PMID: WOS:000186049700009.

908. Krause PJ. Babesiosis diagnosis and treatment. Vector-Borne and Zoonotic Diseases. 2003;3(1):45-51. doi: 10.1089/153036603765627451. PubMed PMID: WOS:000220169800006.

909. Krbková L, Štroblová H. Human granulocyte ehrlichiosis - Diagnostic possibilities and clinical criteria today. Klinicka Mikrobiologie a Infekcni Lekarstvi. 2003;9(6):307-10.

910. Laskay T, Van Zandbergen G, Solbach W. Neutrophil granulocytes - Trojan horses for Leishmania major and other intracellular microbes? Trends in Microbiology. 2003;11(5):210-4. doi: 10.1016/S0966-842X(03)00075-1.

911. Lee KN, Padmalayam I, Baumstark B, Baker SL, Massung RF. Characterization of the ftsZ gene from Ehrlichia chaffeensis, Anaplasma phagocytophilum, and Rickettsia rickettsii, and use as a differential PCR target. DNA Cell Biol. 2003;22(3):179-86. Epub 2003/06/14. doi: 10.1089/104454903321655800. PubMed PMID: 12804116.

912. Liddell AM, Stockham SL, Scott MA, Sumner JW, Paddock CD, Gaudreault-Keener M, et al. Predominance of Ehrlichia ewingii in Missouri dogs. Journal of Clinical Microbiology. 2003;41(10):4617-22. doi: 10.1128/jcm.41.10.4617-4622.2003. PubMed PMID: WOS:000185922900020.

913. Lin M, Rikihisa Y. Obligatory intracellular parasitism by Ehrlichia chaffeensis and Anaplasma phagocytophilum involves caveolae and glycosylphosphatidylinositol-anchored proteins. Cell Microbiol. 2003;5(11):809-20. Epub 2003/10/09. doi: 10.1046/j.1462-5822.2003.00322.x. PubMed PMID: 14531896.

914. Lin M, Rikihisa Y. Ehrlichia chaffeensis and Anaplasma phagocytophilum lack genes for lipid A biosynthesis and incorporate cholesterol for their survival. Infect Immun. 2003;71(9):5324-31. Epub 2003/08/23. doi: 10.1128/iai.71.9.5324-5331.2003. PubMed PMID: 12933880; PubMed Central PMCID: PMCPMC187327.

915. Lin Q, Rikihisa Y, Ohashi N, Zhi N. Mechanisms of variable p44 expression by Anaplasma phagocytophilum. Infect Immun. 2003;71(10):5650-61. Epub 2003/09/23. doi: 10.1128/iai.71.10.5650-5661.2003. PubMed PMID: 14500485; PubMed Central PMCID: PMCPMC201068.

916. Lin Q, Rikihisa Y, Ohashi N, Zhi N. Erratum: Mechanisms of Variable p44 Expression by Anaplasma phagocytophilum (Infection and Immunity (2003) 71:10 (5650-5661)). Infection and Immunity. 2003;71(12):7238. doi: 10.1128/IAI.71.12.7238.2003.

917. Littman MP. Canine borreliosis. Veterinary Clinics of North America-Small Animal Practice. 2003;33(4):827-+. doi: 10.1016/s0195-5616(03)00037-8. PubMed PMID: WOS:000184259900011.

918. Loftis AD, Massung RF, Levin ML. Quantitative real-time PCR assay for detection of Ehrlichia chaffeensis. Journal of Clinical Microbiology. 2003;41(8):3870-2. doi: 10.1128/jcm.41.8.3870-3872.2003. PubMed PMID: WOS:000184746500067.

919. López J, Rivera M, Concha JC, Gatica S, Loeffeholz M, Barriga O. [Serologic evidence for human Ehrlichiosis in Chile]. Rev Med Chil. 2003;131(1):67-70. Epub 2003/03/20. PubMed PMID: 12643221.

920. Lotric-Furlan S, Petrovec M, Avsic-Zupanc T, Strle F. Comparison of patients fulfilling criteria for confirmed and probable human granulocytic ehrlichiosis. Ann N Y Acad Sci. 2003;990:344-5. Epub 2003/07/16. doi: 10.1111/j.1749-6632.2003.tb07385.x. PubMed PMID: 12860648.

921. Lotric-Furlan S, Petrovec M, Avsic-Zupanc T, Strle F. Human granulocytic ehrlichiosis in Slovenia. Ann N Y Acad Sci. 2003;990:279-84. Epub 2003/07/16. doi: 10.1111/j.1749-6632.2003.tb07377.x. PubMed PMID: 12860640.

922. Mahan SM. Antigenic Variation in Anaplasma Marginale and Ehrlichia (Cowdria) Ruminantium. Antigenic Variation2003. p. 243-72.

923. Makala LH, Mangani P, Fujisaki K, Nagasawa H. The current status of major tick borne diseases in Zambia. Veterinary Research. 2003;34(1):27-45. doi: 10.1051/vetres:2002056.

924. Makinen J, Vuorinen I, Oksi J, Peltomaa M, He QS, Marjamaki M, et al. Prevalence of granulocytic Ehrlichia and Borrelia burgdorferi sensu lato in Ixodes ricinus ticks collected from Southwestern Finland and from Vormsi Island in Estonia. Apmis. 2003;111(2):355-62. doi: 10.1034/j.1600-0463.2003.1110209.x. PubMed PMID: WOS:000182548300009.

925. Mannelli A, Boggiatto G, Grego E, Cinco M, Murgia R, Stefanelli S, et al. Acarological risk of exposure to agents of tick-borne zoonoses in the first recognized Italian focus of Lyme borreliosis. Epidemiol Infect. 2003;131(3):1139-47. Epub 2004/02/13. doi: 10.1017/s0950268803001328. PubMed PMID: 14959782; PubMed Central PMCID: PMCPMC2870064.

926. Martins-Neto RG. The fossil tabanids (Diptera Tabanidae): when they began to appreciate warm blood and when they began transmit diseases? Mem Inst Oswaldo Cruz. 2003;98 Suppl 1:29-34. Epub 2003/04/12. doi: 10.1590/s0074-02762003000900006. PubMed PMID: 12687759.

927. Massung RF, Mather TN, Priestley RA, Levin ML. Transmission efficiency of the AP-variant 1 strain of Anaplasma phagocytophila. Ann N Y Acad Sci. 2003;990:75-9. Epub 2003/07/16. doi: 10.1111/j.1749-6632.2003.tb07340.x. PubMed PMID: 12860603.

928. Massung RF, Priestley RA, Levin ML. Route of transmission alters the infectivity of Anaplasma phagocytophila in mice. Ann N Y Acad Sci. 2003;990:494-5. Epub 2003/07/16. doi: 10.1111/j.1749-6632.2003.tb07416.x. PubMed PMID: 12860679.

929. Massung RF, Priestley RA, Miller NJ, Mather TN, Levin ML. Inability of a variant strain of Anaplasma phagocytophilum to infect mice. J Infect Dis. 2003;188(11):1757-63. Epub 2003/11/26. doi: 10.1086/379725. PubMed PMID: 14639548.

930. Massung RF, Slater KG. Comparison of PCR assays for detection of the agent of human granulocytic ehrlichiosis, Anaplasma phagocytophilum. J Clin Microbiol. 2003;41(2):717-22. Epub 2003/02/08. doi: 10.1128/jcm.41.2.717-722.2003. PubMed PMID: 12574272; PubMed Central PMCID: PMCPMC149680.

931. Maurin M, Bakken JS, Dumler JS. Antibiotic susceptibilities of Anaplasma (Ehrlichia) phagocytophilum strains from various geographic areas in the United States. Antimicrob Agents Chemother. 2003;47(1):413-5. Epub 2002/12/25. doi: 10.1128/aac.47.1.413-415.2003. PubMed PMID: 12499227; PubMed Central PMCID: PMCPMC149043.

932. McBride JW, Comer JE, Walker DH. Novel immunoreactive glycoprotein orthologs of Ehrlichia spp. 2003. p. 678-84.

933. McGinley-Smith DE, Tsao SS. Dermatoses from ticks. Journal of the American Academy of Dermatology. 2003;49(3):363-92. doi: 10.1067/s0190-9622(03)01868-1. PubMed PMID: WOS:000185012000001.

934. McQuiston JH, McCall CL, Nicholson WL. Ehrlichiosis and related infections. J Am Vet Med Assoc. 2003;223(12):1750-6. Epub 2003/12/24. doi: 10.2460/javma.2003.223.1750. PubMed PMID: 14690204.

935. McQuiston JH, McCall CL, Nicholson WL. Zoonosis update - Ehrlichiosis and related infections. Journal of the American Veterinary Medical Association. 2003;223(12):1750-6. doi: 10.2460/javma.2003.223.1750. PubMed PMID: WOS:000187313700024.

936. Meeus PFM, Brayton KA, Palmer GH, Barbet AF. Conservation of a gene conversion mechanism in two distantly related paralogues of Anaplasma marginale. Molecular Microbiology. 2003;47(3):633-43. doi: 10.1046/j.1365-2958.2003.03331.x. PubMed PMID: WOS:000180776700004.

937. Misić-Majerus L, Bujić N, Madarić V, Avsic-Zupanc T. [An abortive type of tick-borne meningoencephalitis]. Acta Med Croatica. 2003;57(2):111-6. Epub 2003/07/26. PubMed PMID: 12879690.

938. Morshed MG, Scott JD, Fernando K, Mann RB, Durden LA. Lyme disease spirochete, Borrelia burgdorferi endemic at epicenter in Rondeau Provincial Park, Ontario. Journal of Medical Entomology. 2003;40(1):91-4. doi: 10.1603/0022-2585-40.1.91. PubMed PMID: WOS:000180895500014.

939. Munderloh UG, Tate CM, Lynch MJ, Howerth EW, Kurtti TJ, Davidson WR. Isolation of an Anaplasma sp. organism from white-tailed deer by tick cell culture. Journal of Clinical Microbiology. 2003;41(9):4328-35. doi: 10.1128/jcm.41.9.4328-4335.2003. PubMed PMID: WOS:000185246800048.

940. Nicholson WL, Kuhar DJ, Humphreys JG, Childs JE. Serologic evidence for a novel Ehrlichia species in woodchucks (Marmota monax) from Pennsylvania, USA. In: Hechemy KE, AvsicZupanc T, Childs JE, Raoult DA, editors. Rickettsiology: Present and Future Directions. Annals of the New York Academy of Sciences. 9902003. p. 90-3.

941. Ogden NH, Casey ANJ, Woldehiwet Z, French NP. Transmission of Anaplasma phagocytophilum to Ixodes ricinus ticks from sheep in the acute and post-acute phases of infection. Infection and Immunity. 2003;71(4):2071-8. doi: 10.1128/iai.71.4.2071-2078.2003. PubMed PMID: WOS:000181926200057.

942. Olano JP, Hogrefe W, Seaton B, Walker DH. Clinical manifestations, epidemiology, and laboratory diagnosis of human monocytotropic ehrlichiosis in a commercial laboratory setting. Clinical and Diagnostic Laboratory Immunology. 2003;10(5):891-6. doi: 10.1128/cdli.10.5.891-896.2003. PubMed PMID: WOS:000185383000028.

943. Oporto B, Gil H, Barral M, Hurtado A, Juste RA, Garcia-Perez AL. A survey on Anaplasma phagocytophila in wild small mammals and roe deer (Capreolus capreolus) in Northern Spain. Ann N Y Acad Sci. 2003;990:98-102. Epub 2003/07/16. doi: 10.1111/j.1749-6632.2003.tb07344.x. PubMed PMID: 12860607.

944. Paddock CD, Childs JE. Ehrlichia chaffeensis: A prototypical emerging pathogen. Clinical Microbiology Reviews. 2003;16(1):37-+. doi: 10.1128/cmr.16.1.37-64.2003. PubMed PMID: WOS:000180464800003.

945. Park J, Choi KS, Dumler JS. Major surface protein 2 of Anaplasma phagocytophilum facilitates adherence to granulocytes. Infect Immun. 2003;71(7):4018-25. Epub 2003/06/24. doi: 10.1128/iai.71.7.4018-4025.2003. PubMed PMID: 12819090; PubMed Central PMCID: PMCPMC161989.

946. Park J, Choi KS, Grab DJ, Dumler JS. Divergent interactions of Ehrlichia chaffeensis- and Anaplasma phagocytophilum-infected leukocytes with endothelial cell barriers. Infect Immun. 2003;71(12):6728-33. Epub 2003/11/26. doi: 10.1128/iai.71.12.6728-6733.2003. PubMed PMID: 14638757; PubMed Central PMCID: PMCPMC308917.

947. Park J, Kim KJ, Grab DJ, Dumler JS. Anaplasma phagocytophilum major surface protein-2 (Msp2) forms multimeric complexes in the bacterial membrane. FEMS Microbiol Lett. 2003;227(2):243-7. Epub 2003/11/01. doi: 10.1016/s0378-1097(03)00687-6. PubMed PMID: 14592715.

948. Park JH, Heo EJ, Choi KS, Dumler JS, Chae JS. Detection of antibodies to Anaplasma phagocytophilum and Ehrlichia chaffeensis antigens in sera of Korean patients by western immunoblotting and indirect immunofluorescence assays. Clin Diagn Lab Immunol. 2003;10(6):1059-64. Epub 2003/11/11. doi: 10.1128/cdli.10.6.1059-1064.2003. PubMed PMID: 14607867; PubMed Central PMCID: PMCPMC262439.

949. Parola P, Cornet JP, Sanogo YO, Miller RS, Thien HV, Gonzalez JP, et al. Detection of Ehrlichia spp., Anaplasma spp., Rickettsia spp., and other eubacteria in ticks from the Thai-Myanmar border and Vietnam. J Clin Microbiol. 2003;41(4):1600-8. Epub 2003/04/19. doi: 10.1128/jcm.41.4.1600-1608.2003. PubMed PMID: 12682151; PubMed Central PMCID: PMCPMC153861.

950. Pavia CS. Current and novel therapies for Lyme disease. Expert Opinion on Investigational Drugs. 2003;12(6):1003-16. doi: 10.1517/eoid.12.6.1003.21783. PubMed PMID: WOS:000183476400009.

951. Petrovec M, Sixl W, Marth E, Bushati N, Wüst G. Domestic animals as indicators of Anaplasma species infections in northern Albania. 2003. p. 112-5.

952. Porwancher R. Lyme disease presenting as an influenza-like illness. American Journal of Medicine. 2003;115(1):73-. doi: 10.1016/s0002-9343(03)00240-7. PubMed PMID: WOS:000184134900016.

953. Pratt KM, Gill JE, Leiby DA, Johnson ST, Trouem-Trend J, Cable RG. Evidence of Anaplasma phagocytophilum, the agent of Human Granulocytic Ehrlichiosis, in blood donors from tick-borne disease endemic areas of Connecticut. Transfusion. 2003;43(9):45A-A. PubMed PMID: WOS:000185045700151.

954. Rand PW, Lubelczyk C, Lavigne GR, Elias S, Holman MS, Lacombe EH, et al. Deer density and the abundance of Ixodes scapularis (Acari : Ixodidae). Journal of Medical Entomology. 2003;40(2):179-84. doi: 10.1603/0022-2585-40.2.179. PubMed PMID: WOS:000181992300009.

955. Raoult D. The new rickttsioses. Antibiotiques. 2003;5(4):213-7.

956. Riding G, Hope M, Waltisbuhl D, Willadsen P. Identification of novel protective antigens from Anaplasma marginale. Vaccine. 2003;21(17-18):1874-83. doi: 10.1016/S0264-410X(03)00004-5.

957. Rikihisa Y. Mechanisms to create a safe haven by members of the family Anaplasmataceae. Ann N Y Acad Sci. 2003;990:548-55. Epub 2003/07/16. doi: 10.1111/j.1749-6632.2003.tb07425.x. PubMed PMID: 12860688.

958. Rikihisa Y, Zhang C, Christensen BM. Molecular characterization of Aegyptianella pullorum (Rickettsiales, Anaplasmataceae). J Clin Microbiol. 2003;41(11):5294-7. Epub 2003/11/08. doi: 10.1128/jcm.41.11.5294-5297.2003. PubMed PMID: 14605188; PubMed Central PMCID: PMCPMC262475.

959. Salazar JC, Pope CD, Sellati TJ, Feder HM, Kiely TG, Dardick KR, et al. Coevolution of markers of innate and adaptive immunity in skin and peripheral blood of patients with erythema migrans. Journal of Immunology. 2003;171(5):2660-70. doi: 10.4049/jimmunol.171.5.2660. PubMed PMID: WOS:000184970900061.

960. Sanogo Y, Davoust B, Inokuma H, Camicas JL, Parola P, Brouqui P. First evidence of Anaplasma platys in Rhipicephalus sanguineus (Acari : Ixodida) collected from dogs in Africa. Onderstepoort Journal of Veterinary Research. 2003;70(3):205-12. PubMed PMID: WOS:000186440400004.

961. Sanogo YO, Parola P, Shpynov S, Camicas JL, Brouqui P, Caruso G, et al. Genetic diversity of bacterial agents detected in ticks removed from asymptomatic patients in northeastern Italy. Ann N Y Acad Sci. 2003;990:182-90. Epub 2003/07/16. doi: 10.1111/j.1749-6632.2003.tb07360.x. PubMed PMID: 12860623.

962. Santino I, Iori A, Nicoletti M, Valletta S, Cimmino C, Scoarughi GL, et al. Prevalence of Borrelia Burgdorferi sensu lato genomospecies and of the human granulocytic ehrlichiosis (HGE) agent in Ixodes ricinus ticks collected in the area of Monti Lepini, Italy. Int J Immunopathol Pharmacol. 2003;16(2):105-8. Epub 2003/06/12. doi: 10.1177/039463200301600203. PubMed PMID: 12797900.

963. Scaife H, Woldehiwet Z, Hart CA, Edwards SW. Anaplasma phagocytophilum reduces neutrophil apoptosis in vivo. Infection and Immunity. 2003;71(4):1995-2001. doi: 10.1128/iai.71.4.1995-2001.2003. PubMed PMID: WOS:000181926200047.

964. Scarpulla M, Caristo ME, Macri G, Lillini E. Equine ehrlichiosis in Italy. 2003. p. 259-63.

965. Sehdev AES, Dumler JS. Hepatic pathology in human monocytic ehrlichiosis - Ehrlichia chaffeensis infection. American Journal of Clinical Pathology. 2003;119(6):859-65. doi: 10.1309/f7eab5p7321716lj. PubMed PMID: WOS:000183173200012.

966. Shah SS, McGowan JP. Rickettsial, ehrlichial and Bartonella infections of the myocardium and pericardium. Front Biosci. 2003;8:e197-201. Epub 2002/11/29. doi: 10.2741/995. PubMed PMID: 12456377.

967. Shukla SK, Vandermause MF, Belongia EA, Reed KD, Paskewitz SM, Kazmierczak J. Importance of primer specificity for PCR detection of Anaplasma phagocytophila among Ixodes scapularis ticks from Wisconsin. Journal of Clinical Microbiology. 2003;41(8):4006-. doi: 10.1128/jcm.41.8.4006.2003. PubMed PMID: WOS:000184746500106.

968. Sidel'nikov Iu N, Mediannikov O, Ivanov LI, Zdanovskaia NI. [The first case of granulocytic ehrlichiosis in the Far East of the Russian Federation]. Klin Med (Mosk). 2003;81(2):67-8. Epub 2003/04/11. PubMed PMID: 12685241.

969. Sixl W, Petrovec M, Marth E, Wust G, Stunzner D, Schweiger R, et al. Investigation of Anaplasma phagocytophila infections in Ixodes ricinus and Dermacentor reticulatus ticks in Austria. In: Hechemy KE, AvsicZupanc T, Childs JE, Raoult DA, editors. Rickettsiology: Present and Future Directions. Annals of the New York Academy of Sciences. 9902003. p. 94-7.

970. Skerget M, Wenisch C, Daxboeck F, Krause R, Haberl R, Stuenzner D. Cat or dog ownership and seroprevalence of ehrlichiosis, Q fever, and cat-scratch disease. Emerg Infect Dis. 2003;9(10):1337-40. Epub 2003/11/12. doi: 10.3201/eid0910.030206. PubMed PMID: 14609477; PubMed Central PMCID: PMCPMC3033070.

971. Skotarczak B. Canine ehrlichiosis. Annals of Agricultural and Environmental Medicine. 2003;10(2):137-41. PubMed PMID: WOS:000187536600002.

972. Skotarczak B, Rymaszewska A, Adamska M. Polymerase chain reaction in detection of human granulocytic ehrlichiosis (HGE) agent DNA in Ixodes ricinus ticks. Folia Med Cracov. 2003;44(1-2):179-86. Epub 2004/07/06. PubMed PMID: 15232900.

973. Skotarczak B, Rymaszewska A, Wodecka B, Sawczuk M. Molecular evidence of coinfection of Borrelia burgdorferi sensu lato, human granulocytic ehrlichiosis agent, and Babesia microti in ticks from northwestern Poland. J Parasitol. 2003;89(1):194-6. Epub 2003/03/28. doi: 10.1645/0022-3395(2003)089[0194:Meocob]2.0.Co;2. PubMed PMID: 12659331.

974. Stafford KC, Denicola AJ, Kilpatrick HJ. Reduced abundance of Ixodes scapularis (Acari : Ixodidae) and the tick parasitoid Ixodiphagus hookeri (Hymenoptera : Encyrtidae) with reduction of white-tailed deer. Journal of Medical Entomology. 2003;40(5):642-52. doi: 10.1603/0022-2585-40.5.642. PubMed PMID: WOS:000185604800007.

975. Steere AC, Dhar A, Hernandez J, Fischer PA, Sikand VK, Schoen RT, et al. Systemic symptoms without erythema migrans as the presenting picture of early Lyme disease. American Journal of Medicine. 2003;114(1):58-62. doi: 10.1016/S0002-9343(02)01440-7.

976. Steere AC, McHugh G, Suarez C, Hoitt J, Damle N, Sikand VK. Prospective study of coinfection in patients with erythema migrans. Clin Infect Dis. 2003;36(8):1078-81. Epub 2003/04/10. doi: 10.1086/368187. PubMed PMID: 12684924.

977. Stricker RB, Gaito A, Harris NS, Burrascano JJ. Coinfection in patients with lyme disease: how big a risk? Clin Infect Dis. 2003;37(9):1277-8; author reply 8-9. Epub 2003/10/15. doi: 10.1086/378893. PubMed PMID: 14557980.

978. Stricker RB, Lautin A. The Lyme Wars: time to listen. Expert Opin Investig Drugs. 2003;12(10):1609-14. Epub 2003/10/02. doi: 10.1517/13543784.12.10.1609. PubMed PMID: 14519082.

979. Stricker RB, Phillips SE. Lyme disease without erythema migrans: Cause for concern? [1]. American Journal of Medicine. 2003;115(1):72-3. doi: 10.1016/S0002-9343(03)00244-4.

980. Stuen S, Bergström K, Petrovec M, De Pol IV, Schouls LM. Differences in clinical manifestations and hematological and serological responses after experimental infection with genetic variants of Anaplasma phagocytophilum in sheep. Clinical and Diagnostic Laboratory Immunology. 2003;10(4):692-5. doi: 10.1128/CDLI.10.4.692-695.2003.

981. Sumption KJ, Paxton EA, Bell-Sakyi L. Development of a polyclonal competitive enzyme-linked immunosorbent assay for detection of antibodies to Ehrlichia ruminantium. Clin Diagn Lab Immunol. 2003;10(5):910-6. Epub 2003/09/11. doi: 10.1128/cdli.10.5.910-916.2003. PubMed PMID: 12965926; PubMed Central PMCID: PMCPMC193895.

982. Taillardat-Bisch AV, Raoult D, Drancourt M. RNA polymerase beta-subunit-based phylogeny of Ehrlichia spp., Anaplasma spp., Neorickettsia spp. and Wolbachia pipientis. Int J Syst Evol Microbiol. 2003;53(Pt 2):455-8. Epub 2003/04/25. doi: 10.1099/ijs.0.02411-0. PubMed PMID: 12710612.

983. Tarello W. Canine granulocytic ehrlichiosis (CGE) in Italy. Acta Veterinaria Hungarica. 2003;51(1):73-90. doi: 10.1556/AVet.51.2003.1.7.

984. Teng CH, Barr SC, Chang YF. Cloning and characterization of putative zinc protease genes of Ehrlichia canis. DNA Seq. 2003;14(2):109-21. Epub 2003/06/27. doi: 10.1080/1042517031000073736. PubMed PMID: 12825352.

985. Teng CH, Palaniappan RUM, Chang YF. Cloning and characterization of an Ehrlichia canis gene encoding a protein localized to the morula membrane. Infection and Immunity. 2003;71(4):2218-25. doi: 10.1128/iai.71.4.2218-2225.2003. PubMed PMID: WOS:000181926200073.

986. Topolovec J, Puntarić D, Antolović-Pozgain A, Vuković D, Topolovec Z, Milas J, et al. Serologically detected "new" tick-borne zoonoses in eastern Croatia. Croat Med J. 2003;44(5):626-9. Epub 2003/09/30. PubMed PMID: 14515426.

987. von Loewenich FD, Baumgarten BU, Schroppel K, Geissdorfer W, Rollinghoff M, Bogdan C. High diversity of ankA sequences of Anaplasma phagocytophilum among Ixodes ricinus ticks in Germany. Journal of Clinical Microbiology. 2003;41(11):5033-40. doi: 10.1128/jcm.41.11.5033-5040.2003. PubMed PMID: WOS:000186665500018.

988. Von Loewenich FD, Stumpf G, Baumgarten BU, Röllinghoff M, Dumler JS, Bogdan C. A case of equine granulocytic ehrlichiosis provides molecular evidence for the presence of pathogenic Anaplasma phagocytophilum (HGE agent) in Germany. European Journal of Clinical Microbiology and Infectious Diseases. 2003;22(5):303-5. doi: 10.1007/s10096-003-0935-1.

989. Von Loewenich FD, Stumpf G, Baumgarten BU, Rollinghoff M, Dumler JS, Bogdan C. Human granulocytic ehrlichiosis in Germany - Evidence from serological studies, tick analyses, and a case of equine ehrlichiosis. In: Hechemy KE, AvsicZupanc T, Childs JE, Raoult DA, editors. Rickettsiology: Present and Future Directions. Annals of the New York Academy of Sciences. 9902003. p. 116-7.

990. Wang GQ, Liveris D, Brei B, Wu HY, Falco RC, Fish D, et al. Real-time PCR for simultaneous detection and quantification of Borrelia burgdorferi in field-collected Ixodes scapularis ticks from the northeastern United States. Applied and Environmental Microbiology. 2003;69(8):4561-5. doi: 10.1128/aem.69.8.4561-4565.2003. PubMed PMID: WOS:000184672500030.

991. Wen B, Cao W, Pan H. Ehrlichiae and ehrlichial diseases in china. Ann N Y Acad Sci. 2003;990:45-53. Epub 2003/07/16. doi: 10.1111/j.1749-6632.2003.tb07335.x. PubMed PMID: 12860598.

992. Whist SK, Storset AK, Johansen GM, Larsen HJS. Modulation of leukocyte populations and immune responses in sheep experimentally infected with Anaplasma (formerly Ehrlichia) phagocytophilum. Veterinary Immunology and Immunopathology. 2003;94(3-4):163-75. doi: 10.1016/S0165-2427(03)00101-6.

993. Winslow GM, Yager E, Li JSY. Mechanisms of humoral immunity during Ehrlichia chaffeensis infection. In: Hechemy KE, AvsicZupanc T, Childs JE, Raoult DA, editors. Rickettsiology: Present and Future Directions. Annals of the New York Academy of Sciences. 9902003. p. 435-43.

994. Yabsley MJ, Dugan VG, Stallknecht DE, Little SE, Lockhart JM, Dawson JE, et al. Evaluation of a prototype Ehrlichia chaffeensis surveillance system using white-tailed deer (Odocoileus virginianus) as natural sentinels. Vector-Borne and Zoonotic Diseases. 2003;3(4):195-207. doi: 10.1089/153036603322662183. PubMed PMID: WOS:000220171800004.

995. Yago T, Leppänen A, Carlyon JA, Akkoyunlu M, Karmakar S, Fikrig E, et al. Structurally distinct requirements for binding of P-selectin glycoprotein ligand-1 and sialyl Lewis x to Anaplasma phagocytophilum and P-selectin. J Biol Chem. 2003;278(39):37987-97. Epub 2003/07/09. doi: 10.1074/jbc.M305778200. PubMed PMID: 12847092.

996. Zhang XF, Zhang JZ, Long SW, Ruble RP, Yu XJ. Experimental Ehrlichia chaffeensis infection in beagles. Journal of Medical Microbiology. 2003;52(11):1021-6. doi: 10.1099/jmm.0.05234-0. PubMed PMID: WOS:000220238800013.

997. Zhang Y, Palmer GH, Abbott JR, Howard CJ, Hope JC, Brown WC. CpG ODN 2006 and IL-12 are comparable for priming Th1 lymphocyte and IgG responses in cattle immunized with a rickettsial outer membrane protein in alum. Vaccine. 2003;21(23):3307-18. doi: 10.1016/s0264-410x(03)00176-2. PubMed PMID: WOS:000184043200029.

998. Zinsstag J, Schelling E. Vector-borne diseases in humans and animals: activities of the Swiss Tropical Institute and risks for Switzerland. Schweiz Arch Tierheilkd. 2003;145(12):559-66, 68-9. Epub 2004/01/17. doi: 10.1024/0036-7281.145.12.559. PubMed PMID: 14725182.

999. Zintl A, Mulcahy G, Skerrett HE, Taylor SM, Gray JS. Babesia divergens, a bovine blood parasite of veterinary and zoonotic importance. Clinical Microbiology Reviews. 2003;16(4):622-+. doi: 10.1128/cmr.16.4.622-636.2003. PubMed PMID: WOS:000186049700003.

1000. Abbott JR, Palmer GH, Howard CJ, Hope JC, Brown WC. Anaplasma marginale major surface protein 2 CD4(+)-T-cell epitopes are evenly distributed in conserved and hypervariable regions (HVR), whereas linear B-cell epitopes are predominantly located in the HVR. Infection and Immunity. 2004;72(12):7360-6. doi: 10.1128/iai.72.12.7360-7366.2004. PubMed PMID: WOS:000225453900075.

1001. Adamska M, Skotarczak B, editors. Detection of Anaplasma phagocytophilum DNA in blood, spleen and liver of roe deer (Capreolus capreolus) from northwestern Poland. 9th European Multicolloquium of Parasitology; 2004 Jul 18-23; Valencia, SPAIN2004.

1002. Adelson ME, Rao RVS, Tilton RC, Cabets K, Eskow E, Fein L, et al. Prevalence of Borrelia burgdorferi, Bartonella spp., Babesia microti, and Anaplasma phagocytophila in Ixodes scapularis ticks collected in northern New Jersey. Journal of Clinical Microbiology. 2004;42(6):2799-801. doi: 10.1128/jcm.42.6.2799-2801.2004. PubMed PMID: WOS:000222015800071.

1003. Alekseev AN, Dubinina HV, Jushkova OV. First report on the coexistence and compatibility of seven tick-borne pathogens in unfed adult Ixodes persulcatus Schulze (Acarina: Ixodidae). Int J Med Microbiol. 2004;293 Suppl 37:104-8. Epub 2004/05/19. doi: 10.1016/s1433-1128(04)80015-9. PubMed PMID: 15146991.

1004. Amiel C, Abadia G, Choudat D. [Human granulocytic ehrlichiosis in Europe]. Med Mal Infect. 2004;34(3):111-22. Epub 2004/12/25. doi: 10.1016/j.medmal.2003.10.002. PubMed PMID: 15617351.

1005. Bakken JS, Dumler JS. Ehrlichiosis and anaplasmosis. Infections in Medicine. 2004;21(9):433-51.

1006. Barigye R, García-Ortiz MA, Rojas Ramírez EE, Rodríguez SD. Identification of IgG2-specific antigens in Mexican Anaplasma marginale strains. 2004. p. 84-94.

1007. Benson MJ, Gawronski JD, Eveleigh DE, Benson DR. Intracellular symbionts and other bacteria associated with deer ticks (Ixodes scapularis) from Nantucket and Wellfleet, Cape Cod, Massachusetts. Applied and Environmental Microbiology. 2004;70(1):616-20. doi: 10.1128/aem.70.1.616-620.2004. PubMed PMID: WOS:000188115300080.

1008. Bormane A, Lucenko I, Duks A, Mavtchoutko V, Ranka R, Salmina K, et al. Vectors of tick-borne diseases and epidemiological situation in Latvia in 1993-2002. Int J Med Microbiol. 2004;293 Suppl 37:36-47. Epub 2004/05/19. doi: 10.1016/s1433-1128(04)80007-x. PubMed PMID: 15146983.

1009. Branger S, Rolain JM, Raoult D. Evaluation of antibiotic susceptibilities of Ehrlichia canis, Ehrlichia chaffeensis, and Anaplasma phagocytophilum by real-time PCR. Antimicrob Agents Chemother. 2004;48(12):4822-8. Epub 2004/11/25. doi: 10.1128/aac.48.12.4822-4828.2004. PubMed PMID: 15561862; PubMed Central PMCID: PMCPMC529244.

1010. Brouqui P, Bacellar F, Baranton G, Birtles RJ, Bjoërsdorff A, Blanco JR, et al. Guidelines for the diagnosis of tick-borne bacterial diseases in Europe. Clinical Microbiology and Infection. 2004;10(12):1108-32. doi: 10.1111/j.1469-0691.2004.01019.x.

1011. Brülisauer F, Thoma R, Cagienard A, Hofmann-Lehmann R, Lutz H, Meli ML, et al. Anaplasmosis in a Swiss dairy farm: An epidemiological outbreak investigation. Schweizer Archiv fur Tierheilkunde. 2004;146(10):451-9. doi: 10.1024/0036-7281.146.10.451.

1012. Brzostek T. [Human granulocytic ehrlichiosis co-incident with Lyme borreliosis in pregnant woman--a case study]. Przegl Epidemiol. 2004;58(2):289-94. Epub 2004/11/03. PubMed PMID: 15517809.

1013. Bunikis J, Tsao J, Luke CJ, Luna MG, Fish D, Barbour AG. Borrelia burgdorferi infection in a natural population of Peromyscus leucopus mice: A longitudinal study in an area where lyme borreliosis is highly endemic. Journal of Infectious Diseases. 2004;189(8):1515-23. doi: 10.1086/382594. PubMed PMID: WOS:000220735400023.

1014. Calic SB, Galvão MA, Bacellar F, Rocha CM, Mafra CL, Leite RC, et al. Human ehrlichioses in Brazil: first suspect cases. Braz J Infect Dis. 2004;8(3):259-62. Epub 2004/10/12. doi: 10.1590/s1413-86702004000300011. PubMed PMID: 15476059.

1015. Carlyon JA, Abdel-Latif D, Pypaert M, Lacy P, Fikrig E. Anaplasma phagocytophilum utilizes multiple host evasion mechanisms to thwart NADPH oxidase-mediated killing during neutrophil infection. Infect Immun. 2004;72(8):4772-83. Epub 2004/07/24. doi: 10.1128/iai.72.8.4772-4783.2004. PubMed PMID: 15271939; PubMed Central PMCID: PMCPMC470610.

1016. Casey ANJ, Birtles RJ, Radford AD, Bown KJ, French NP, Woldehiwet Z, et al. Groupings of highly similar major surface protein (p44)-encoding paralogues: a potential index of genetic diversity amongst isolates of Anaplasma phagocytophilum. Microbiology (Reading). 2004;150(Pt 3):727-34. Epub 2004/03/03. doi: 10.1099/mic.0.26648-0. PubMed PMID: 14993322.

1017. Choi KS, Grab DJ, Dumler JS. Anaplasma phagocytophilum infection induces protracted neutrophil degranulation. Infect Immun. 2004;72(6):3680-3. Epub 2004/05/25. doi: 10.1128/iai.72.6.3680-3683.2004. PubMed PMID: 15155684; PubMed Central PMCID: PMCPMC415649.

1018. Choi KS, Scorpio DG, Dumler JS. Anaplasma phagocytophilum ligation to toll-like receptor (TLR) 2, but not to TLR4, activates macrophages for nuclear factor-kappa B nuclear translocation. J Infect Dis. 2004;189(10):1921-5. Epub 2004/05/04. doi: 10.1086/386284. PubMed PMID: 15122530.

1019. Cinco M, Barbone F, Grazia Ciufolini M, Mascioli M, Anguero Rosenfeld M, Stefanel P, et al. Seroprevalence of tick-borne infections in forestry rangers from northeastern Italy. Clin Microbiol Infect. 2004;10(12):1056-61. Epub 2004/12/21. doi: 10.1111/j.1469-0691.2004.01026.x. PubMed PMID: 15606631.

1020. Courtney JW, Kostelnik LM, Zeidner NS, Massung RF. Multiplex real-time PCR for detection of anaplasma phagocytophilum and Borrelia burgdorferi. J Clin Microbiol. 2004;42(7):3164-8. Epub 2004/07/10. doi: 10.1128/jcm.42.7.3164-3168.2004. PubMed PMID: 15243077; PubMed Central PMCID: PMCPMC446246.

1021. De La Fuente J, Garcia-Garcia JC, Barbet AF, Blouin EF, Kocan KM. Adhesion of outer membrane proteins containing tandem repeats of Anaplasma and Ehrlichia species (Rickettsiales: Anaplasmataceae) to tick cells. Veterinary Microbiology. 2004;98(3-4):313-22. doi: 10.1016/j.vetmic.2003.11.001.

1022. de la Fuente J, Naranjo V, Ruiz-Fons F, Vicente J, Estrada-Pena A, Almazan C, et al. Prevalence of tick-borne pathogens in ixodid ticks (Acari : Ixodidae) collected from European wild boar (Sus scrofa) and Iberian red deer (Cervus elaphus hispanicus) in central Spain. European Journal of Wildlife Research. 2004;50(4):187-96. doi: 10.1007/s10344-004-0060-1. PubMed PMID: WOS:000225975100005.

1023. DeShields A, Borman-Shoap E, Peters JE, Gaudreault-Keener M, Arens MQ, Storch GA. Detection of pathogenic Ehrlichia in ticks collected at acquisition sites of human ehrlichiosis in Missouri. Mo Med. 2004;101(2):132-6. Epub 2004/05/04. PubMed PMID: 15119113.

1024. Dodd RY, Leiby DA. Emerging infectious threats to the blood supply. 2004. p. 191-207.

1025. Dolan MC, Maupin GO, Schneider BS, Denatale C, Hamon N, Cole C, et al. Control of immature Ixodes scapularis (Acari: Ixodidae) on rodent reservoirs of Borrelia burgdorferi in a residential community of southeastern Connecticut. J Med Entomol. 2004;41(6):1043-54. Epub 2004/12/21. doi: 10.1603/0022-2585-41.6.1043. PubMed PMID: 15605643.

1026. Dumler JS, Brouqui P. Molecular diagnosis of human granulocytic anaplasmosis. Expert Rev Mol Diagn. 2004;4(4):559-69. Epub 2004/07/01. doi: 10.1586/14737159.4.4.559. PubMed PMID: 15225103.

1027. Durden LA, Polur RN, Nims T, Banks CW, Oliver JH, Jr. Ectoparasites and other epifaunistic arthropods of sympatric cotton mice and golden mice: comparisons and implications for vector-borne zoonotic diseases. J Parasitol. 2004;90(6):1293-7. Epub 2005/02/18. doi: 10.1645/ge-333r. PubMed PMID: 15715219.

1028. Ehlers S. Commentary: adaptive immunity in the absence of innate immune responses? The un-Tolled truth of the silent invaders. Eur J Immunol. 2004;34(7):1783-8. Epub 2004/06/24. doi: 10.1002/eji.200425250. PubMed PMID: 15214026.

1029. Ehlers S. Adaptive immunity in the absence of innate immune responses? The un-Tolled truth of the silent invaders. European Journal of Immunology. 2004;34(7):1783-8. doi: 10.1002/eji.200425250. PubMed PMID: WOS:000222563800002.

1030. Felek S, Telford S, Falco RC, Rikihisa Y. Sequence analysis of p44 homologs expressed by Anaplasma phagocytophilum in infected ticks feeding on naive hosts and in mice infected by tick attachment. Infection and Immunity. 2004;72(2):659-66. doi: 10.1128/iai.72.2.659-666.2004. PubMed PMID: WOS:000188766400006.

1031. Fenollar F, Raoult D. Molecular genetic methods for the diagnosis of fastidious microorganisms. Apmis. 2004;112(11-12):785-807. doi: 10.1111/j.1600-0463.2004.apm11211-1206.x. PubMed PMID: WOS:000226305700006.

1032. Foley JE, Foley P, Brown RN, Lane RS, Dumler JS, Madigan JE. Ecology of Anaplasma phagocytophilum and Borrelia burgdorferi in the western United States. Journal of Vector Ecology. 2004;29(1):41-50. PubMed PMID: WOS:000222528600005.

1033. Garcia-Garcia JC, De La Fuente J, Kocan KM, Blouin EF, Halbur T, Onet VC, et al. Mapping of B-cell epitopes in the N-terminal repeated peptides of Anaplasma marginale major surface protein 1a and characterization of the humoral immune response of cattle immunized with recombinant and whole organism antigens. Veterinary Immunology and Immunopathology. 2004;98(3-4):137-51. doi: 10.1016/j.vetimm.2003.11.003. PubMed Central PMCID: PMCNovartis(United States).

1034. Genchi C, Rizzoli A. Tick-borne diseases: An emerging public health problem? Parassitologia. 2004;46(1-2):107.

1035. Gill J, Pratt K, Leiby D, Johnson S, Trouern-Trend J, Cable R. Human granulocytic ehrlichiosis: The next tick-borne disease to pose a blood safety threat? Transfusion. 2004;44(9):10A-A. PubMed PMID: WOS:000223575600033.

1036. Grygorczuk S, Hermanowska-Szpakowicz T, Kondrusik M, Pancewicz S, Zajkowska J. [Ehrlichiosis--a disease rarely recognized in Poland]. Wiad Lek. 2004;57(9-10):456-61. Epub 2005/03/16. PubMed PMID: 15765762.

1037. Grzeszczuk A, Stańczak J, Kubica-Biernat B, Racewicz M, Kruminis-Łozowska W, Prokopowicz D. Human anaplasmosis in north-eastern Poland: seroprevalence in humans and prevalence in Ixodes ricinus ticks. Ann Agric Environ Med. 2004;11(1):99-103. Epub 2004/07/09. PubMed PMID: 15236505.

1038. Gunasekara D, Fujii Y, Rusuvai E, Yoshiie K, Ohashi N, Nakamura M. Human monocytic cells upregulate superoxide-generating activity and mRNAs for its components in response to heat-stable and heat-unstable factors released to medium conditioned with Ehrlichia chaffeensis-infected THP-1 cells. Acta Medica Nagasakiensia. 2004;49(1-2):39-44.

1039. Hamilton KS, Standaert SM, Kinney MC. Characteristic peripheral blood findings in human ehrlichiosis. Mod Pathol. 2004;17(5):512-7. Epub 2004/02/21. doi: 10.1038/modpathol.3800075. PubMed PMID: 14976527.

1040. Hartelt K, Oehme R, Frank H, Brockmann SO, Hassler D, Kimmig P. Pathogens and symbionts in ticks: prevalence of Anaplasma phagocytophilum (Ehrlichia sp.), Wolbachia sp., Rickettsia sp., and Babesia sp. in Southern Germany. Int J Med Microbiol. 2004;293 Suppl 37:86-92. Epub 2004/05/19. doi: 10.1016/s1433-1128(04)80013-5. PubMed PMID: 15146989.

1041. Hofmann-Lehmann R, Meli ML, Dreher UM, Gönczi E, Deplazes P, Braun U, et al. Concurrent infections with vector-borne pathogens associated with fatal hemolytic anemia in a cattle herd in Switzerland. Journal of Clinical Microbiology. 2004;42(8):3775-80. doi: 10.1128/JCM.42.8.3775-3780.2004.

1042. Holman MS, Caporale DA, Goldberg J, Lacombe E, Lubelczyk C, Rand PW, et al. Anaplasma phagocytophilum, Babesia microti, and Borrelia burgdorferi in Ixodes scapularis, southern coastal Maine. Emerging Infectious Diseases. 2004;10(4):744-6. doi: 10.3201/eid1004.030566. PubMed PMID: WOS:000220578600034.

1043. Hubalek Z. An annotated checklist of pathogenic microorganisms associated with migratory birds. Journal of Wildlife Diseases. 2004;40(4):639-59. doi: 10.7589/0090-3558-40.4.639. PubMed PMID: WOS:000226454000003.

1044. Hulíninská D, Dřevová H, Votýpka J, Langrová K, Kurzová Z. Prevalence of Borrelia burgdorferi sensu lato species among patients in the Czech Republic; direct sequencing analysis and real-time polymerase chain reaction. Epidemiologie, Mikrobiologie, Imunologie. 2004;53(4):183-91.

1045. Hulinska D, Langrova K, Pejcoch M, Pavlasek I. Detection of Anaplasma phagocytophilum in animals by real-time polymerase chain reaction. Apmis. 2004;112(4-5):239-47. doi: 10.1111/j.1600-0463.2004.apm11204-0503.x. PubMed PMID: WOS:000222916700003.

1046. Hunfeld KP, Bittner T, Rödel R, Brade V, Cinatl J. New real-time PCR-based method for in vitro susceptibility testing of Anaplasma phagocytophilum against antimicrobial agents. Int J Antimicrob Agents. 2004;23(6):563-71. Epub 2004/06/15. doi: 10.1016/j.ijantimicag.2004.02.019. PubMed PMID: 15194126.

1047. Hunfeld KP, Brade V. Zoonotic Babesia: possibly emerging pathogens to be considered for tick-infested humans in Central Europe. Int J Med Microbiol. 2004;293 Suppl 37:93-103. Epub 2004/05/19. doi: 10.1016/s1433-1128(04)80014-7. PubMed PMID: 15146990.

1048. Inayoshi M, Naitou H, Kawamori F, Masuzawa T, Ohashi N. Characterization of Ehrlichia species from Ixodes ovatus ticks at the foot of Mt. Fuji, Japan. Microbiology and Immunology. 2004;48(10):737-45. doi: 10.1111/j.1348-0421.2004.tb03599.x. PubMed PMID: WOS:000224471200004.

1049. Jongejan F, Uilenberg G. The global importance of ticks. Parasitology. 2004;129(SUPPL.):S3-S14. doi: 10.1017/S0031182004005967.

1050. JW IJ, Mueller AC. Neutrophil NADPH oxidase is reduced at the Anaplasma phagocytophilum phagosome. Infect Immun. 2004;72(9):5392-401. Epub 2004/08/24. doi: 10.1128/iai.72.9.5392-5401.2004. PubMed PMID: 15322037; PubMed Central PMCID: PMCPMC517486.

1051. Katz B, Waites K. Emerging intracellular bacterial infections. Clin Lab Med. 2004;24(3):627-49, vi. Epub 2004/08/25. doi: 10.1016/j.cll.2004.05.005. PubMed PMID: 15325059.

1052. Korenberg EI. Problems in the study and prophylaxis of mixed infections transmitted by ixodid ticks. International Journal of Medical Microbiology. 2004;293:80-5. doi: 10.1016/s1433-1128(04)80012-3. PubMed PMID: WOS:000221637700012.

1053. Lafont F, Abrami L, Van Der Goot FG. Bacterial subversion of lipid rafts. Current Opinion in Microbiology. 2004;7(1):4-10. doi: 10.1016/j.mib.2003.12.007.

1054. Lane RS, Steinlein DB, Mun J. Human behaviors elevating exposure to Ixodes pacificus (Acari: Ixodidae) nymphs and their associated bacterial zoonotic agents in a hardwood forest. J Med Entomol. 2004;41(2):239-48. Epub 2004/04/06. doi: 10.1603/0022-2585-41.2.239. PubMed PMID: 15061284.

1055. Lásiková S, Moravcová L, Pícha D, Holecková D, Zdárský E. [Detection of anti-Ehrlichia antibodies and direct demonstration of Ehrlichia nucleic acid using the polymerase chain reaction (PCR) in patients in the Czech Republic]. Klin Mikrobiol Infekc Lek. 2004;10(1):25-9. Epub 2004/04/22. PubMed PMID: 15100980.

1056. Leiby DA, Gill JE. Transfusion-transmitted tick-borne infections: a cornucopia of threats. Transfus Med Rev. 2004;18(4):293-306. Epub 2004/10/22. doi: 10.1016/j.tmrv.2004.07.001. PubMed PMID: 15497129.

1057. Levin ML, Coble DJ, Ross DE. Reinfection with Anaplasma phagocytophilum in BALB/c mice and cross-protection between two sympatric isolates. Infection and Immunity. 2004;72(8):4723-30. doi: 10.1128/iai.72.8.4723-4730.2004. PubMed PMID: WOS:000222932600048.

1058. Levin ML, Ross DE. Acquisition of different isolates of Anaplasma phagocytophilum by Ixodes scapularis from a model animal. Vector-Borne and Zoonotic Diseases. 2004;4(1):53-9. doi: 10.1089/153036604773082997. PubMed PMID: WOS:000220590400006.

1059. Lin Q, Rikihisa Y, Felek S, Wang X, Massung RF, Woldehiwet Z. Anaplasma phagocytophilum has a functional msp2 gene that is distinct from p44. Infect Immun. 2004;72(7):3883-9. Epub 2004/06/24. doi: 10.1128/iai.72.7.3883-3889.2004. PubMed PMID: 15213131; PubMed Central PMCID: PMCPMC427402.

1060. Lin Q, Rikihisa Y, Massung RF, Woldehiwet Z, Falco RC. Polymorphism and transcription at the p44-1/p44-18 genomic locus in Anaplasma phagocytophilum strains from diverse geographic regions. Infection and Immunity. 2004;72(10):5574-81. doi: 10.1128/IAI.72.10.5574-5581.2004.

1061. Loa CC, Adelson ME, Mordechai E, Raphaelli I, Tilton RC. Serological diagnosis of human babesiosis by IgG enzyme-linked immunosorbent assay. Curr Microbiol. 2004;49(6):385-9. Epub 2005/02/08. doi: 10.1007/s00284-004-4373-9. PubMed PMID: 15696612.

1062. Lodes MJ, Dillon DC, Houghton RL, Skeiky YA. Expression cloning. Methods Mol Med. 2004;94:91-106. Epub 2004/02/13. doi: 10.1385/1-59259-679-7:91. PubMed PMID: 14959824.

1063. Loftis AD, Nicholson WL, Levin ML. Evaluation of immunocompetent and immunocompromised mice (Mus musculus) for infection with Ehrlichia chaffeensis and transmission to Amblyomma americanum ticks. Vector-Borne and Zoonotic Diseases. 2004;4(4):323-33. doi: 10.1089/vbz.2004.4.323. PubMed PMID: WOS:000226379700008.

1064. Lohr CV, Brayton KA, Barbet AF, Palmer GH. Characterization of the Anaplasma marginale msp2 locus and its synteny with the omp1/p30 loci of Ehrlichia chaffeensis and E-canis. Gene. 2004;325:115-21. doi: 10.1016/j.gene.2003.10.003. PubMed PMID: WOS:000188291200012.

1065. Lotric-Furlan S, Petrovec M, Avsic-Zupanc T, Strle F. Comparison of patients fulfilling criteria for confirmed and probable human granulocytic ehrlichiosis. Scand J Infect Dis. 2004;36(11-12):817-22. Epub 2005/03/15. doi: 10.1080/00365540410021171. PubMed PMID: 15764167.

1066. MacDonald KA, Chomel BB, Kittleson MD, Kasten RW, Thomas WP, Pesavento P. A Prospective Study of Canine Infective Endocarditis in Northern California (1999-2001): Emergence of Bartonella as a Prevalent Etiologic Agent. Journal of Veterinary Internal Medicine. 2004;18(1):56-64. doi: 10.1892/0891-6640(2004)18<56:APSOCI>2.0.CO;2.

1067. Maender JL, Tyring SK. Treatment and prevention of rickettsial and ehrlichial infections. Dermatol Ther. 2004;17(6):499-504. Epub 2004/12/02. doi: 10.1111/j.1396-0296.2004.04052.x. PubMed PMID: 15571498.

1068. Magnarelli LA, Ijdo JW, Ramakrishnan U, Henderson DW, Stafford KC, Fikrig E. Use of recombinant antigens of Borrelia burgdorferi and Anaplasma phagocytophilum in enzyme-linked immunosorbent assays to detect antibodies in white-tailed deer. Journal of Wildlife Diseases. 2004;40(2):249-58. doi: 10.7589/0090-3558-40.2.249. PubMed PMID: WOS:000222553000010.

1069. Massung RF, Priestley RA, Levin ML. Transmission route efficacy and kinetics of Anaplasma phagocytophilum infection in the white-footed mouse, Peromyscus leucopus. Vector-Borne and Zoonotic Diseases. 2004;4(4):310-8. doi: 10.1089/vbz.2004.4.310. PubMed PMID: WOS:000226379700006.

1070. Mayer-Scholl A, Averhoff P, Zychlinsky A. How do neutrophils and pathogens interact? Curr Opin Microbiol. 2004;7(1):62-6. Epub 2004/03/24. doi: 10.1016/j.mib.2003.12.004. PubMed PMID: 15036142.

1071. Molad T, Brayton KA, Palmer GH, Michaeli S, Shkap V. Molecular conservation of MSP4 and MSP5 in Anaplasma marginale and A-centrale vaccine strain. Veterinary Microbiology. 2004;100(1-2):55-64. doi: 10.1016/j.vetmic.2004.01.018. PubMed PMID: WOS:000221634700007.

1072. Müllegger RR. Dermatological manifestations of Lyme borreliosis. European Journal of Dermatology. 2004;14(5):296-309.

1073. Munderloh UG, Lynch MJ, Herron MJ, Palmer AT, Kurtti TJ, Nelson RD, et al. Infection of endothelial cells with Anaplasma marginale and A-phagocytophilum. Veterinary Microbiology. 2004;101(1):53-64. doi: 10.1016/j.vetmic.2004.02.011. PubMed PMID: WOS:000222474700006.

1074. Nafeev AA. [Laboratory diagnosis of natural focus tick-borne infections]. Klin Lab Diagn. 2004;(8):46-7. Epub 2004/10/06. PubMed PMID: 15461005.

1075. Narasimhan S, Montgomery RR, DePonte K, Tschudi C, Marcantonio N, Anderson JF, et al. Disruption of Ixodes scapularis anticoagulation by using RNA interference. Proc Natl Acad Sci U S A. 2004;101(5):1141-6. Epub 2004/01/28. doi: 10.1073/pnas.0307669100. PubMed PMID: 14745044; PubMed Central PMCID: PMCPMC337020.

1076. Nielsen H, Fournier PE, Pedersen IS, Krarup H, Ejlertsen T, Raoult D. Serological and molecular evidence of Rickettsia helvetica in Denmark. Scandinavian Journal of Infectious Diseases. 2004;36(8):559-63. doi: 10.1080/00365540410020776. PubMed PMID: WOS:000223243000003.

1077. Nolen-Walston RD, D'Oench SM, Hanelt LM, Sharkey LC, Paradis MR. Acute recumbency associated with Anaplasma phagocytophilum infection in a horse. Journal of the American Veterinary Medical Association. 2004;224(12):1964-6+31. doi: 10.2460/javma.2004.224.1964.

1078. Olano JP, Wen G, Feng HM, McBride JW, Walker DH. Animal model - Histologic, serologic, and molecular analysis of persistent ehrlichiosis in a murine model. American Journal of Pathology. 2004;165(3):997-1006. doi: 10.1016/s0002-9440(10)63361-5. PubMed PMID: WOS:000223732000028.

1079. Oteo JA, Blanco JR. Epidemiological importance of human Ehrlichiosis in Europe. Infektoloski Glasnik. 2004;24(1):5-10.

1080. Park J, Kim KJ, Choi KS, Grab DJ, Dumler JS. Anaplasma phagocytophilum AnkA binds to granulocyte DNA and nuclear proteins. Cell Microbiol. 2004;6(8):743-51. Epub 2004/07/09. doi: 10.1111/j.1462-5822.2004.00400.x. PubMed PMID: 15236641.

1081. Parola P. Tick-borne rickettsial diseases: emerging risks in Europe. Comp Immunol Microbiol Infect Dis. 2004;27(5):297-304. Epub 2004/07/01. doi: 10.1016/j.cimid.2004.03.006. PubMed PMID: 15225980.

1082. Pazdiora ZP. Contemporary knowledge about ehrlichiosis. Prakticky Lekar. 2004;84(12):714-7.

1083. Pérez-Eid C. [Emergence of tick-borne diseases in temperate countries]. Ann Biol Clin (Paris). 2004;62(2):149-54. Epub 2004/03/30. PubMed PMID: 15047466.

1084. Reine NJ. Infection and blood transfusion: A guide to donor screening. Clinical Techniques in Small Animal Practice. 2004;19(2):68-74. doi: 10.1053/j.ctsap.2004.01.002.

1085. Remy V, Hansmann Y, Christmann D. Early clinical presentation following ticks' bite: etiological diagnosis? Medecine Et Maladies Infectieuses. 2004;34:S24-S7. doi: 10.1016/s0399-077x(04)90008-x. PubMed PMID: WOS:000223100600009.

1086. Rizzoli A, Rosà R, Mantelli B, Pecchioli E, Hauffe H, Tagliapietra V, et al. [Ixodes ricinus, transmitted diseases and reservoirs]. Parassitologia. 2004;46(1-2):119-22. Epub 2004/08/13. PubMed PMID: 15305699.

1087. Rosen H. Bacterial responses to neutrophil phagocytosis. Current Opinion in Hematology. 2004;11(1):1-6. doi: 10.1097/00062752-200401000-00002.

1088. Ross DE, Levin ML. Effects of Anaplasma phagocytophilum infection on the molting success of Ixodes scapularis (Acari : Ixodidae) larvae. Journal of Medical Entomology. 2004;41(3):476-83. doi: 10.1603/0022-2585-41.3.476. PubMed PMID: WOS:000221409200030.

1089. Rymaszewska A, Adamska M. Participation of Ixodes ricinus developmental stages in transmission of Anaplasma (Ehrlichia) phagocytophila. Wiadomości parazytologiczne. 2004;50(3):563-9.

1090. Rymaszewska A, Skotarczak B, editors. Identification of Anaplasma phagocytophilum in Ixodes ricinus ticks in West Pomerania, Poland. 9th European Multicolloquium of Parasitology; 2004 Jul 18-23; Valencia, SPAIN2004.

1091. Saito-Ito A, Yano Y, Dantrakool A, Hashimoto T, Takada N. Survey of rodents and ticks in human babesiosis emergence area in Japan: First detection of Babesia microti-like parasites in Ixodes ovatus. Journal of Clinical Microbiology. 2004;42(5):2268-70. doi: 10.1128/jcm.42.5.2268-2270.2004. PubMed PMID: WOS:000221424100069.

1092. Sambri V, Marangoni A, Storni E, Cavrini F, Moroni A, Sparacino M, et al. [Tick borne zoonosis: selected clinical and diagnostic aspects]. Parassitologia. 2004;46(1-2):109-13. Epub 2004/08/13. PubMed PMID: 15305697.

1093. Santino I, Cammarata E, Franco S, Galdiero F, Oliva B, Sessa R, et al. Multicentric study of seroprevalence of Borrelia burgdorferi and Anaplasma phagocytophila in high-risk groups in regions of central and southern Italy. Int J Immunopathol Pharmacol. 2004;17(2):219-23. Epub 2004/06/03. doi: 10.1177/039463200401700214. PubMed PMID: 15171823.

1094. Santos AS, Santos-Silva MM, Almeida VC, Bacellar F, Dumler JS. Detection of Anaplasma phagocytophilum DNA in Ixodes ticks (Acari : Ixodidae) from Madeira Island and Setubal District mainland Portugal. Emerging Infectious Diseases. 2004;10(9):1643-8. doi: 10.3201/eid1009.040276. PubMed PMID: WOS:000223740200018.

1095. Scorpio DG, Akkoyunlu M, Fikrig E, Dumler JS. CXCR2 blockade influences Anaplasma phagocytophilum propagation but not histopathology in the mouse model of human granulocytic anaplasmosis. Clin Diagn Lab Immunol. 2004;11(5):963-8. Epub 2004/09/11. doi: 10.1128/cdli.11.5.963-968.2004. PubMed PMID: 15358660; PubMed Central PMCID: PMCPMC515272.

1096. Scorpio DG, Caspersen K, Ogata H, Park J, Dumler JS. Restricted changes in major surface protein-2 (msp2) transcription after prolonged in vitro passage of Anaplasma phagocytophilum. BMC Microbiol. 2004;4:1. Epub 2004/01/10. doi: 10.1186/1471-2180-4-1. PubMed PMID: 14713314; PubMed Central PMCID: PMCPMC317292.

1097. Scott JD, Fernando K, Durden LA, Morshed MG. Lyme disease spirochete, Borrelia burgdorferi, endemic in epicenter at Turkey Point, Ontario. Journal of Medical Entomology. 2004;41(2):226-30. doi: 10.1603/0022-2585-41.2.226. PubMed PMID: WOS:000220185500012.

1098. Shpynov SN, Rudakov NV, Iastrebov VK, Leonova GN, Khazova TG, Egorova NV, et al. [New evidence for the detection of ehrlichia and anaplasma in ixodes ticks in Russia and Kazakhstan]. Med Parazitol (Mosk). 2004;(2):10-4. Epub 2004/06/15. PubMed PMID: 15193042.

1099. Skotarczak B, Adamska M, Rymaszewska A, Suproń M, Sawczuk M, Maciejewska A. [Anaplasma phagocytophila and protozoans of Babesia genus in dogs from endemic areas of Lyme disease in north-western Poland]. Wiad Parazytol. 2004;50(3):555-61. Epub 2006/07/27. PubMed PMID: 16865968.

1100. Skotarczak B, Adamska M, Supron M. Blood DNA analysis for Ehrlichia (Anaplasma) phagocytophila and Babesia spp. of dogs from northern Poland. Acta Veterinaria Brno. 2004;73(3):347-51. doi: 10.2754/avb200473030347. PubMed PMID: WOS:000224132100007.

1101. Sréter T, Sréter-Lancz Z, Széll Z, Kálmán D. Anaplasma phagocytophilum: an emerging tick-borne pathogen in Hungary and Central Eastern Europe. Ann Trop Med Parasitol. 2004;98(4):401-5. Epub 2004/07/02. doi: 10.1179/000349804225003343. PubMed PMID: 15228721.

1102. Stadtbäumer K, Leschnik MW, Nell B. Tick-borne encephalitis virus as a possible cause of optic neuritis in a dog. Veterinary Ophthalmology. 2004;7(4):271-7. doi: 10.1111/j.1463-5224.2004.04030.x. PubMed Central PMCID: PMCAventis(Austria)

Glaxo Wellcome(Austria)

Hoechst(Austria)

Merck(Austria)

Pfizer(Austria).

1103. Stańczak J, Gabre RM, Kruminis-Łozowska W, Racewicz M, Kubica-Biernat B. Ixodes ricinus as a vector of Borrelia burgdorferi sensu lato, Anaplasma phagocytophilum and Babesia microti in urban and suburban forests. Ann Agric Environ Med. 2004;11(1):109-14. Epub 2004/07/09. PubMed PMID: 15236507.

1104. Stanek G, Gray J, Strle F, Wormser G, Hengge UR. Lyme borreliosis. Lancet Infectious Diseases. 2004;4(4):197-9. doi: 10.1016/S1473-3099(04)00965-X.

1105. Steele Jr JCH. Emerging infections and their causative agents. Clinics in Laboratory Medicine. 2004;24(3):xi-xiii. doi: 10.1016/j.cll.2004.05.012.

1106. Steere AC, Coburn J, Glickstein L. The emergence of Lyme disease. Journal of Clinical Investigation. 2004;113(8):1093-101. doi: 10.1172/jci200421681. PubMed PMID: WOS:000221365100005.

1107. Stich RW, Olah GA, Brayton KA, Brown WC, Fechheimer M, Green-Church K, et al. Identification of a novel Anaplasma marginale appendage-associated protein that localizes with actin filaments during intraerythrocytic infection. Infection and Immunity. 2004;72(12):7257-64. doi: 10.1128/IAI.72.12.7257-7264.2004.

1108. Stone JH, Dierberg K, Aram G, Dumler JS. Human monocytic ehrlichilosis. Jama-Journal of the American Medical Association. 2004;292(18):2263-70. doi: 10.1001/jama.292.18.2263. PubMed PMID: WOS:000225070000029.

1109. Strle F. Human granulocytic ehrlichiosis in Europe. Int J Med Microbiol. 2004;293 Suppl 37:27-35. Epub 2004/05/19. doi: 10.1016/s1433-1128(04)80006-8. PubMed PMID: 15146982.

1110. Süss J, Fingerle V, Hunfeld KP, Schrader C, Wilske B. [Tick-borne human pathogenic microorganisms found in Europe and those considered nonpathogenic. Part II: Bacteria, parasites and mixed infections]. Bundesgesundheitsblatt Gesundheitsforschung Gesundheitsschutz. 2004;47(5):470-86. Epub 2004/06/19. doi: 10.1007/s00103-004-0837-0. PubMed PMID: 15205761.

1111. Szentgyorgyi L, Kapiller Z. Human ehrlichiosis. Literature review. Magyar Allatorvosok Lapja. 2004;126(5):300-7. PubMed PMID: WOS:000221616900007.

1112. Tamás S, Zsuzsanna SL, Zoltán S, Dóra K. About granulocytic anaplasmosis of humans and animals in the context of first detection of Anaplasma phagocytophilum in Hungary. Short secondary communication and literature review. Magyar Allatorvosok Lapja. 2004;126(11):694-700.

1113. Telford SR, 3rd, Goethert HK. Emerging tick-borne infections: rediscovered and better characterized, or truly 'new' ? Parasitology. 2004;129 Suppl:S301-27. Epub 2005/06/09. doi: 10.1017/s0031182003004669. PubMed PMID: 15940821.

1114. Tomasiewicz K, Modrzewska R, Buczek A, Stańczak J, Maciukajć J. The risk of exposure to Anaplasma phagocytophilum infection in Mid-Eastern Poland. Ann Agric Environ Med. 2004;11(2):261-4. Epub 2005/01/04. PubMed PMID: 15627334.

1115. Torpy JM, Glass T, Glass RM. Ehrlichiosis. JAMA. 2004;292(18):2302. doi: 10.1001/jama.292.18.2302.

1116. Uilenberg G, Thiaucourt F, Jongejan F. On molecular taxonomy: what is in a name? Experimental and Applied Acarology. 2004;32(4):301-12. doi: 10.1023/B:APPA.0000023235.23090.a7. PubMed PMID: WOS:000220733000008.

1117. Van Donkersgoed J, Gertonson A, Bridges M, Raths D, Dargatz D, Wagner B, et al. Prevalence of antibodies to bluetongue virus and Anaplasma marginale in Montana yearling cattle entering Alberta feedlots: Fall 2001. Canadian Veterinary Journal. 2004;45(6):486-92.

1118. von Loewenich FD, Scorpio DG, Reischl U, Dumler JS, Bogdan C. Control of Anaplasma phagocytophilum, an obligate intracellular pathogen, in the absence of inducible nitric oxide synthase, phagocyte NADPH oxidase, tumor necrosis factor, Toll-like receptor (TLR)2 and TLR4, or the TLR adaptor molecule MyD88. European Journal of Immunology. 2004;34(7):1789-97. doi: 10.1002/eji.200425250. PubMed PMID: WOS:000222563800003.

1119. Wang T, Akkoyunlu M, Banerjee R, Fikrig E. Interferon-gamma deficiency reveals that 129Sv mice are inherently more susceptible to Anaplasma phagocytophilum than C57BL/6 mice. Fems Immunology and Medical Microbiology. 2004;42(3):299-305. doi: 10.1016/j.femsim.2004.06.001. PubMed PMID: WOS:000224681200004.

1120. Wang X, Rikihisa Y, Lai TH, Kumagai Y, Zhi N, Reed SM. Rapid sequential changeover of expressed p44 genes during the acute phase of Anaplasma phagocytophilum infection in horses. Infection and Immunity. 2004;72(12):6852-9. doi: 10.1128/IAI.72.12.6852-6859.2004.

1121. Yabsley MJ, Norton TM, Powell MR, Davidson WR. Molecular and serologic evidence of tick-borne ehrlichiae in three species of lemurs from St. Catherines Island, Georgia, USA. Journal of Zoo and Wildlife Medicine. 2004;35(4):503-9. doi: 10.1638/03-116. PubMed PMID: WOS:000226450200009.

1122. Zeman P, Januska J, Orolinova M, Stuen S, Struhar V, Jebavy L. High seroprevalence of granulocytic ehrlichiosis distinguishes sheep that were the source of an alimentary epidemic of tick-borne encephalitis. Wien Klin Wochenschr. 2004;116(17-18):614-6. Epub 2004/11/02. doi: 10.1007/s00508-004-0191-0. PubMed PMID: 15515879.

1123. Zhang JZ, Guo H, Winslow GM, Yu XJ. Expression of members of the 28-kilodalton major outer membrane protein family of Ehrlichia chaffeensis during persistent infection. Infection and Immunity. 2004;72(8):4336-43. doi: 10.1128/iai.72.8.4336-4343.2004. PubMed PMID: WOS:000222932600002.

1124. Zwoliński J, Chmielewska-Badora J, Cisak E, Buczek A, Dutkiewicz J. [Prevalence of antibodies to Anaplasma phagocytophilum and Borrelia burgdorferi in forestry workers from the Lublin region]. Wiad Parazytol. 2004;50(2):221-7. Epub 2006/07/25. PubMed PMID: 16859027.

1125. Abbott JR, Palmer GH, Kegerreis KA, Hetrick PF, Howard CJ, Hope JC, et al. Rapid and long-term disappearance of CD4(+) T lymphocyte responses specific for Anaplasma marginale major surface protein-2 (MSP2) in MSP2 vaccinates following challenge with live A-marginale. Journal of Immunology. 2005;174(11):6702-15. doi: 10.4049/jimmunol.174.11.6702. PubMed PMID: WOS:000229298400024.

1126. Alberti A, Addis MF, Sparagano O, Zobba R, Chessa B, Cubeddu T, et al. Anaplasma phagocytophilum, Sardinia, Italy [8]. Emerging Infectious Diseases. 2005;11(8):1322-4. doi: 10.3201/eid1108.050085.

1127. Alberti A, Zobba R, Chessa B, Addis MF, Sparagano O, Pinna Parpaglia ML, et al. Equine and canine Anaplasma phagocytophilum strains isolated on the island of Sardinia (Italy) are phylogenetically related to pathogenic strains from the United States. Appl Environ Microbiol. 2005;71(10):6418-22. Epub 2005/10/06. doi: 10.1128/aem.71.10.6418-6422.2005. PubMed PMID: 16204571; PubMed Central PMCID: PMCPMC1265917.

1128. Amsden JR, Warmack S, Gubbins PO. Tick-borne bacterial, rickettsial, spirochetal, and protozoal infectious diseases in the United States: A comprehensive review. Pharmacotherapy. 2005;25(2):191-210. doi: 10.1592/phco.25.2.191.56948. PubMed PMID: WOS:000226796500006.

1129. Arsenault WG, Messick JB. Acute granulocytic ehrlichiosis in a rottweiler. Journal of the American Animal Hospital Association. 2005;41(5):323-6. doi: 10.5326/0410323.

1130. Bakoss P. Infectious diseases have been a scourge to mankind? Epidemiologie, Mikrobiologie, Imunologie. 2005;54(2):47-53.

1131. Barbet AF, Agnes JT, Moreland AL, Lundgren AM, Alleman AR, Noh SM, et al. Identification of functional promoters in the msp2 expression loci of Anaplasma marginale and Anaplasma phagocytophilum. Gene. 2005;353(1):89-97. Epub 2005/06/07. doi: 10.1016/j.gene.2005.03.036. PubMed PMID: 15935572.

1132. Beldomenico PM, Chomel BB, Foley JE, Sacks BN, Baldi CJ, Kasten RW, et al. Environmental factors associated with Bartonella vinsonii subsp berkhoffii seropositivity in free-ranging coyotes from northern California. Vector-Borne and Zoonotic Diseases. 2005;5(2):110-9. doi: 10.1089/vbz.2005.5.110. PubMed PMID: WOS:000230637900004.

1133. Bell CA, Patel R. A real-time combined polymerase chain reaction assay for the rapid detection and differentiation of Anaplasma phagocytophilum, Ehrlichia chaffeensis, and Ehrlichia ewingii. Diagn Microbiol Infect Dis. 2005;53(4):301-6. Epub 2005/11/03. doi: 10.1016/j.diagmicrobio.2005.06.019. PubMed PMID: 16263231.

1134. Bexfield NH, Villiers EJ, Herrtage ME. Immune-mediated haemolytic anaemia and thromboytopenia associated with Anaplasma phagocytophilum in a dog. Journal of Small Animal Practice. 2005;46(11):543-8. doi: 10.1111/j.1748-5827.2005.tb00284.x. PubMed PMID: WOS:000233517100005.

1135. Blaškovič D, Barák I. Oligo-chip based detection of tick-borne bacteria. FEMS Microbiology Letters. 2005;243(2):473-8. doi: 10.1016/j.femsle.2005.01.010.

1136. Bluszcz-Roznowska A, Olszok I, Kucharz EJ. [Ehrlichiosis]. Przegl Lek. 2005;62(12):1529-31. Epub 2006/06/22. PubMed PMID: 16786789.

1137. Borjesson DL, Brazzell JL, Feferman R. Platelet dysfunction after association with Anaplasma phagocytophilum in vitro. Ann N Y Acad Sci. 2005;1063:413-5. Epub 2006/02/17. doi: 10.1196/annals.1355.074. PubMed PMID: 16481550.

1138. Borjesson DL, Kobayashi SD, Whitney AR, Voyich JM, Argue CM, Deleo FR. Insights into pathogen immune evasion mechanisms: Anaplasma phagocytophilum fails to induce an apoptosis differentiation program in human neutrophils. J Immunol. 2005;174(10):6364-72. Epub 2005/05/10. doi: 10.4049/jimmunol.174.10.6364. PubMed PMID: 15879137.

1139. Brayton KA, Kappmeyer LS, Herndon DR, Dark MJ, Tibbals DL, Palmer GH, et al. Complete genome sequencing of Anaplasma marginale reveals that the surface is skewed to two superfamilies of outer membrane proteins. Proc Natl Acad Sci U S A. 2005;102(3):844-9. Epub 2004/12/25. doi: 10.1073/pnas.0406656102. PubMed PMID: 15618402; PubMed Central PMCID: PMCPMC545514.

1140. Buckingham SC. Tick-borne infections in children: epidemiology, clinical manifestations, and optimal management strategies. Paediatr Drugs. 2005;7(3):163-76. Epub 2005/06/28. doi: 10.2165/00148581-200507030-00003. PubMed PMID: 15977962.

1141. Bukowska B, Walory J. Anaplasma phagocytophilum - Epidemiology, diagnostics and therapy. Postepy Mikrobiologii. 2005;44(3):211-26.

1142. Caporale DA, Johnson CM, Millard BJ. Presence of Borrelia burgdorferi (Spirochaetales : Spirochaetaceae) in southern Kettle Moraine State Forest, Wisconsin, and characterization of strain W97F51. Journal of Medical Entomology. 2005;42(3):457-72. doi: 10.1603/0022-2585(2005)042[0457:Pobbss]2.0.Co;2. PubMed PMID: WOS:000229067400036.

1143. Carlyon JA. Laboratory Maintenance of Anaplasma phagocytophilum. Curr Protoc Microbiol. 2005;Chapter 3:Unit 3A.2. Epub 2008/09/05. doi: 10.1002/9780471729259.mc03a02s00. PubMed PMID: 18770564.

1144. Carlyon JA, Ryan D, Archer K, Fikrig E. Effects of Anaplasma phagocytophilum on host cell ferritin mRNA and protein levels. Infect Immun. 2005;73(11):7629-36. Epub 2005/10/22. doi: 10.1128/iai.73.11.7629-7636.2005. PubMed PMID: 16239567; PubMed Central PMCID: PMCPMC1273867.

1145. Choi KS, Park JT, Dumler JS. Anaplasma phagocytophilum delay of neutrophil apoptosis through the p38 mitogen-activated protein kinase signal pathway. Infect Immun. 2005;73(12):8209-18. Epub 2005/11/22. doi: 10.1128/iai.73.12.8209-8218.2005. PubMed PMID: 16299317; PubMed Central PMCID: PMCPMC1307085.

1146. Christova I, Gladnishka T. Prevalence of infection with Francisella tularensis, Borrelia burgdorferi sensu lato and Anaplasma phagocytophilum in rodents from an endemic focus of tularemia in Bulgaria. Ann Agric Environ Med. 2005;12(1):149-52. Epub 2005/07/21. PubMed PMID: 16028881.

1147. Cisak E, Chmielewska-Badora J, Zwoliński J, Wójcik-Fatla A, Polak J, Dutkiewicz J. Risk of tick-borne bacterial diseases among workers of Roztocze National Park (south-eastern Poland). Ann Agric Environ Med. 2005;12(1):127-32. Epub 2005/07/21. PubMed PMID: 16028877.

1148. Coetzee JF, Apley MD, Kocan KM, Rurangirwa FR, Van Donkersgoed J. Comparison of three oxytetracycline regimens for the treatment of persistent Anaplasma marginale infections in beef cattle. Veterinary Parasitology. 2005;127(1):61-73. doi: 10.1016/j.vetpar.2004.08.017. PubMed Central PMCID: PMCMerial(Canada)

Pfizer(United States).

1149. Coleman JL, LeVine D, Thill C, Kuhlow C, Benach JL. Babesia microti and Borrelia burgdorferi follow independent courses of infection in mice. Journal of Infectious Diseases. 2005;192(9):1634-41. doi: 10.1086/496891. PubMed PMID: WOS:000232333000017.

1150. De Barros Macieira D, Belle Messick J, De Mello Figueiredo Cerqueira A, Alexandre Freire IM, Coelho Linhares GF, De Oliveira Almeida NK, et al. Prevalence of Ehrlichia canis infection in thrombocytopenic dogs from Rio de Janeiro, Brazil. Veterinary Clinical Pathology. 2005;34(1):44-8.

1151. de la Fuente J, Ayoubi P, Blouin EF, Almazán C, Naranjo V, Kocan KM. Gene expression profiling of human promyelocytic cells in response to infection with Anaplasma phagocytophilum. Cell Microbiol. 2005;7(4):549-59. Epub 2005/03/12. doi: 10.1111/j.1462-5822.2004.00485.x. PubMed PMID: 15760455.

1152. de la Fuente J, Lew A, Lutz H, Meli ML, Hofmann-Lehmann R, Shkap V, et al. Genetic diversity of anaplasma species major surface proteins and implications for anaplasmosis serodiagnosis and vaccine development. Animal health research reviews / Conference of Research Workers in Animal Diseases. 2005;6(1):75-89. doi: 10.1079/AHR2005104.

1153. de la Fuente J, Massung RF, Wong SJ, Chu FK, Lutz H, Meli M, et al. Sequence analysis of the msp4 gene of Anaplasma phagocytophilum strains. J Clin Microbiol. 2005;43(3):1309-17. Epub 2005/03/08. doi: 10.1128/jcm.43.3.1309-1317.2005. PubMed PMID: 15750101; PubMed Central PMCID: PMCPMC1081214.

1154. de la Fuente J, Naranjo V, Ruiz-Fons F, Hofle U, de Mera IGF, Villanua D, et al. Potential vertebrate reservoir hosts and invertebrate vectors of Anaplasma marginale and A-phagocytophilum in central Spain. Vector-Borne and Zoonotic Diseases. 2005;5(4):390-401. doi: 10.1089/vbz.2005.5.390. PubMed PMID: WOS:000234778200010.

1155. Demma LJ, Holman RC, McQuiston JH, Krebs JW, Swerdlow DL. Epidemiology of human ehrlichiosis and anaplasmosis in the United States, 2001-2002. Am J Trop Med Hyg. 2005;73(2):400-9. Epub 2005/08/17. PubMed PMID: 16103612.

1156. Dinglasan RR, Jacobs-Lorena M. Insight into a conserved lifestyle: Protein-carbohydrate adhesion strategies of vector-borne pathogens. Infection and Immunity. 2005;73(12):7797-807. doi: 10.1128/IAI.73.12.7797-7807.2005.

1157. Doyle CK, Labruna MB, Breitschwerdt EB, Tang YW, Corstvet RE, Hegarty BC, et al. Detection of medically important Ehrlichia by quantitative multicolor TaqMan real-time polymerase chain reaction of the dsb gene. J Mol Diagn. 2005;7(4):504-10. Epub 2005/10/21. doi: 10.1016/s1525-1578(10)60581-8. PubMed PMID: 16237220; PubMed Central PMCID: PMCPMC1888493.

1158. Dugan VG, Gaydos JK, Stallknecht DE, Little SE, Beall AD, Mead DG, et al. Detection of Ehrlichia spp. in raccoons (Procyon lotor) from Georgia. Vector-Borne and Zoonotic Diseases. 2005;5(2):162-71. doi: 10.1089/vbz.2005.5.162. PubMed PMID: WOS:000230637900011.

1159. Dumler JS. Anaplasma and Ehrlichia infection. Ann N Y Acad Sci. 2005;1063:361-73. Epub 2006/02/17. doi: 10.1196/annals.1355.069. PubMed PMID: 16481544.

1160. Dumler JS, Choi KS, Garcia-Garcia JC, Barat NS, Scorpio DG, Garyu JW, et al. Human granulocytic anaplasmosis and Anaplasma phagocytophilum. Emerg Infect Dis. 2005;11(12):1828-34. Epub 2006/02/21. doi: 10.3201/eid1112.050898. PubMed PMID: 16485466; PubMed Central PMCID: PMCPMC3367650.

1161. Elston DM. New and emerging infectious diseases. Journal of the American Academy of Dermatology. 2005;52(6):1062-8. doi: 10.1016/j.jaad.2005.02.048.

1162. Foley JE, Queen EV, Sacks B, Foley P. GIS-facilitated spatial epidemiology of tick-borne diseases in coyotes (Canis latrans) in northern and coastal California. Comparative Immunology Microbiology and Infectious Diseases. 2005;28(3):197-212. doi: 10.1016/j.cimid.2005.01.006. PubMed PMID: WOS:000229201000004.

1163. Foster J, Ganatra M, Kamal I, Ware J, Makarova K, Ivanova N, et al. The Wolbachia genome of Brugia malayi: Endosymbiont evolution within a human pathogenic nematode. Plos Biology. 2005;3(4):599-614. doi: 10.1371/journal.pbio.0030121. PubMed PMID: WOS:000228279900008.

1164. Franzen P, Aspan A, Egenvall A, Gunnarsson A, Aberg L, Pringle J. Acute clinical, hematologic, serologic, and polymerase chain reaction findings in horses experimentally infected with a European strain of Anaplasma phagocytophilum. Journal of Veterinary Internal Medicine. 2005;19(2):232-9. doi: 10.1892/0891-6640(2005)19<232:Achsap>2.0.Co;2. PubMed PMID: WOS:000227882600016.

1165. Futse JE, Brayton KA, Knowles Jr DP, Palmer GH. Structural basis for segmental gene conversion in generation of Anaplasma marginale outer membrane protein variants. Molecular Microbiology. 2005;57(1):212-21. doi: 10.1111/j.1365-2958.2005.04670.x.

1166. Garyu JW, Choi KS, Grab DJ, Dumler JS. Defective phagocytosis in Anaplasma phagocytophilum-infected neutrophils. Infect Immun. 2005;73(2):1187-90. Epub 2005/01/25. doi: 10.1128/iai.73.2.1187-1190.2005. PubMed PMID: 15664962; PubMed Central PMCID: PMCPMC547103.

1167. Garyu JW, Dumler JS. Anaplasma phagocytophilum infection reduces expression of phagocytosis-related receptors on neutrophils. Ann N Y Acad Sci. 2005;1063:416-9. Epub 2006/02/17. doi: 10.1196/annals.1355.075. PubMed PMID: 16481551.

1168. Ge Y, Yoshiie K, Kuribayashi F, Lin M, Rikihisa Y. Anaplasma phagocytophilum inhibits human neutrophil apoptosis via upregulation of bfl-1, maintenance of mitochondrial membrane potential and prevention of caspase 3 activation. Cell Microbiol. 2005;7(1):29-38. Epub 2004/12/25. doi: 10.1111/j.1462-5822.2004.00427.x. PubMed PMID: 15617521.

1169. Go RS, Lovrich SD, Callister SM, Meyer LA, Bottner WA, Farnen JP. Human cyclic thrombocytopenia and Anaplasma spp. infection. Eur J Haematol. 2005;74(2):182-3. Epub 2005/01/19. doi: 10.1111/j.1600-0609.2004.00361.x. PubMed PMID: 15654914.

1170. Goodfellow M, Shaw S. Exotic diseases of dogs and cats at risk of importation to Ireland. Ir Vet J. 2005;58(5):271-7. Epub 2005/01/01. doi: 10.1186/2046-0481-58-5-271. PubMed PMID: 21851670; PubMed Central PMCID: PMCPMC3113903.

1171. Grzeszczuk A, Stańczak J, Pogorzelska J, Prokopowicz D. [Diagnostics of human granulocytic anaplasmosis]. Wiad Parazytol. 2005;51(2):109-14. Epub 2006/07/15. PubMed PMID: 16838619.

1172. Güner ES, Watanabe M, Kadosaka T, Polat E, Gargili A, Gulanber A, et al. Seroepidemiology of Borrelia burgdorferi sensu lato and Anaplasma phagocytophilum in wild mice captured in northern Turkey. Epidemiology & Infection. 2005;133(2):331-6. doi: 10.1017/s0950268804003309. PubMed PMID: 136868279. Language: English. Entry Date: 20050603. Revision Date: 20220513. Publication Type: journal article.

1173. Harrus S, Baneth G. Drivers for the emergence and re-emergence of vector-borne protozoal and bacterial diseases. International Journal for Parasitology. 2005;35(11-12):1309-18. doi: 10.1016/j.ijpara.2005.06.005. PubMed PMID: WOS:000232901200012.

1174. Hercogová J, Dobrá N, Vaňousová D. Lyme borreliosis. Cesko-Slovenska Dermatologie. 2005;80(6):309-20.

1175. Herron MJ, Ericson ME, Kurtti TJ, Munderloh UG. The interactions of Anaplasma phagocytophilum, endothelial cells, and human neutrophils. Ann N Y Acad Sci. 2005;1063:374-82. Epub 2006/02/17. doi: 10.1196/annals.1355.090. PubMed PMID: 16481545.

1176. Holden K, Hodzic E, Feng S, Freet KJ, Lefebvre RB, Barthold SW. Coinfection with Anaplasma phagocytophilum alters Borrelia burgdorferi population distribution in C3H/HeN mice. Infection and Immunity. 2005;73(6):3440-4. doi: 10.1128/iai.73.6.3440-3444.2005. PubMed PMID: WOS:000229485900029.

1177. Holzer BR. Tick borne diseases. Therapeutische Umschau. 2005;62(11):757-63. doi: 10.1024/0040-5930.62.11.757.

1178. Huang H, Unver A, Perez MJ, Orellana NG, Rikihisa Y. Prevalence and molecular analysis of Anaplasma platys in dogs in Lara, Venezuela. Brazilian Journal of Microbiology. 2005;36(3):211-6. doi: 10.1590/s1517-83822005000300002. PubMed PMID: WOS:000238450000002.

1179. Kirtz G, Meli M, Leidinger E, Ludwig P, Thum D, Czettel B, et al. Anaplasma phagocytophilum infection in a dog: Identifying the causative agent using PCR. Journal of Small Animal Practice. 2005;46(6):300-3. doi: 10.1111/j.1748-5827.2005.tb00325.x. PubMed Central PMCID: PMCMerial.

1180. Lane RS, Mun J, Eisen RJ, Eisen L. Western gray squirrel (Rodentia : Sciuridae): A primary reservoir host of Borrelia burgdorferi in Californian oak woodlands? Journal of Medical Entomology. 2005;42(3):388-96. doi: 10.1603/0022-2585(2005)042[0388:Wgsrsa]2.0.Co;2. PubMed PMID: WOS:000229067400028.

1181. Leblond A, Pradier S, Pitel PH, Fortier G, Boireau P, Chadoeuf J, et al. An epidemiological survey of equine anaplasmosis (Anaplasma phagocytophilum) in Southern France. Revue Scientifique Et Technique-Office International Des Epizooties. 2005;24(3):899-908. doi: 10.20506/rst.24.3.1612. PubMed PMID: WOS:000236519900008.

1182. Lester SJ, Breitschwerdt EB, Collis CD, Hegarty BC. Anaplasma phagocytophilum infection (granulocytic anaplasmosis) in a dog from Vancouver Island. Canadian Veterinary Journal. 2005;46(9):825-7. PubMed Central PMCID: PMCApotex(Canada).

1183. Lew A, Jorgensen W. Molecular approaches to detect and study the organisms causing bovine tick borne diseases: Babesiosis and anaplasmosis. African Journal of Biotechnology. 2005;4(4):292-302.

1184. Loftis AD, Gill JS, Schriefer ME, Levin ML, Eremeeva ME, Gilchrist MJR, et al. Detection of Rickettsia, Borrelia, and Bartonella in Carios kelleyi (Acari : Argasidae). Journal of Medical Entomology. 2005;42(3):473-80. doi: 10.1603/0022-2585(2005)042[0473:Dorbab]2.0.Co;2. PubMed PMID: WOS:000229067400037.

1185. Lopez JE, Siems WF, Palmer GH, Brayton KA, McGuire TC, Norimine J, et al. Identification of novel antigenic proteins in a complex Anaplasma marginale outer membrane immunogen by mass spectrometry and genomic mapping. Infection and Immunity. 2005;73(12):8109-18. doi: 10.1128/IAI.73.12.8109-8118.2005.

1186. Lorenz EC, Thomas KG. 47-Year-old man with fever and headache. Mayo Clinic Proceedings. 2005;80(3):411-4. doi: 10.4065/80.3.411.

1187. Lotric-Furlan S, Rojko T, Strle F. Concentration of procalcitonin and C-reactive protein in patients with human granulocytic anaplasmosis and the initial phase of tick-borne encephalitis. Ann N Y Acad Sci. 2005;1063:439-41. Epub 2006/02/17. doi: 10.1196/annals.1355.081. PubMed PMID: 16481557.

1188. Magnarelli L, Fikrig E. Detection of antibodies to Borrelia burgdorferi in naturally infected horses in the USA by enzyme-linked immunosorbent assay using whole-cell and recombinant antigens. Research in Veterinary Science. 2005;79(2):99-103. doi: 10.1016/j.rvsc.2004.11.009.

1189. Magnarelli LA, Bushmich SL, Ijdo JW, Fikrig E. Seroprevalence of antibodies against Borrelia burgdorferi and Anaplasma phagocytophilum in cats. American Journal of Veterinary Research. 2005;66(11):1895-9. doi: 10.2460/ajvr.2005.66.1895. PubMed PMID: WOS:000232936200007.

1190. Marquez-Jimenez FJ, Hidalgo-Pontiveros A, Contreras-Chova F, Rodriguez-Liebana JJ, Muniain-Ezcurra MA. Ticks (Acarina : Ixodidae) as vectors and reservoirs of pathogen microorganims in Spain. Enfermedades Infecciosas Y Microbiologia Clinica. 2005;23(2):94-102. doi: 10.1157/13071613. PubMed PMID: WOS:000227421700010.

1191. Massung RF, Courtney JW, Hiratzka SL, Pitzer VE, Smith G, Dryden RL. Anaplasma phagocytophilum in white-tailed deer. Emerg Infect Dis. 2005;11(10):1604-6. Epub 2005/12/02. doi: 10.3201/eid1110.041329. PubMed PMID: 16318705; PubMed Central PMCID: PMCPMC3366735.

1192. Massung RF, Zeidner NS, Dolan MC, Roellig D, Gabitzsch E, Troughton DR. Prophylactic use of sustained-release doxycycline blocks tick-transmitted infection by Anaplasma phagocytophilum in a murine model. In: Hechemy KE, Oteo JA, Raoult DA, Silverman DJ, Blanco JR, editors. Rickettsioses: From Genome to Proteome, Pathobiology, and Rickettsiae as an International Threat. Annals of the New York Academy of Sciences. 10632005. p. 436-8.

1193. Mattner J, DeBord KL, Ismail N, Goff RD, Cantu C, Zhou DP, et al. Exogenous and endogenous glycolipid antigens activate NKT cells during microbial infections. Nature. 2005;434(7032):525-9. doi: 10.1038/nature03408. PubMed PMID: WOS:000227836000046.

1194. Merino FJ, Nebreda T, Serrano JL, Fernández-Soto P, Encinas A, Pérez-Sánchez R. Tick species and tick-borne infections identified in population from a rural area of Spain. Epidemiol Infect. 2005;133(5):943-9. Epub 2005/09/27. doi: 10.1017/s0950268805004061. PubMed PMID: 16181517; PubMed Central PMCID: PMCPMC2870328.

1195. Mišić-Majerus L, Bujić N, Madarić V, Avšič-Županc T. Hepatitis caused by tick-borne encephalitis virus (TBEV) - A rare clinical manifestation outside the central nervous system involvement. Acta Medica Croatica. 2005;59(4):347-52.

1196. Morshed MG, Scott JD, Fernando K, Beati L, Mazerolle DF, Geddes G, et al. Migratory songbirds disperse ticks across Canada, and first isolation of the Lyme disease spirochete, Borrelia burgdorferi, from the avian tick, Ixodes auritulus. Journal of Parasitology. 2005;91(4):780-90. doi: 10.1645/ge-3437.1. PubMed PMID: WOS:000231814200009.

1197. Muramatsu Y, Ikeda E, Morita C, Tamura Y. Detection of ehrlichial DNA in small rodents captured in a woodland area of Hokkaido, the northernmost island of Japan, where Lyme disease is endemic. Jpn J Infect Dis. 2005;58(5):316-9. Epub 2005/10/27. PubMed PMID: 16249629.

1198. Mwangi W, Brown WC, Splitter GA, Zhuang Y, Kegerreis K, Palmer GH. Enhancement of antigen acquisition by dendritic cells and MHC class II-restricted epitope presentation to CD4+ T cells using VP22 DNA vaccine vectors that promote intercellular spreading following initial transfection. J Leukoc Biol. 2005;78(2):401-11. Epub 2005/04/29. doi: 10.1189/jlb.1204722. PubMed PMID: 15857936.

1199. Norimine J, Brown WC. Intrahaplotype and interhaplotype pairing of bovine leukocyte antigen DQA and DQB molecules generate functional DQ molecules important for priming CD4(+) T-lymphocyte responses. Immunogenetics. 2005;57(10):750-62. Epub 2005/10/13. doi: 10.1007/s00251-005-0045-6. PubMed PMID: 16220347.

1200. Ohashi N, Inayoshi M, Kitamura K, Kawamori F, Kawaguchi D, Nishimura Y, et al. Anaplasma phagocytophilum-infected ticks, Japan. Emerging Infectious Diseases. 2005;11(11):1780-3. doi: 10.3201/eid1111.050407. PubMed PMID: WOS:000233076300025.

1201. Oteo JA, Brouqui P. [Ehrlichiosis and human anaplasmosis]. Enferm Infecc Microbiol Clin. 2005;23(6):375-80. Epub 2005/06/23. doi: 10.1157/13076178. PubMed PMID: 15970171.

1202. Palmer GH, Knowles Jr DP, Rodriguez JL, Gnad DP, Hollis LC, Marston T, et al. Erratum: Stochastic transmission of multiple genotypically distinct Anaplasma marginale strains in a herd with high prevalence of Anaplasma infection (Journal of Clinical Microbiology (2004) 42, 11 (5381-5384)). Journal of Clinical Microbiology. 2005;43(7):3587. doi: 10.1128/JCM.43.7.3587.2005.

1203. Park HS, Lee JH, Jeong EJ, Park TK, Kim TY, Chae JS, et al. Differentiation of Anaplasmataceae through partial groEL gene analysis. Microbiology and Immunology. 2005;49(7):655-62. doi: 10.1111/j.1348-0421.2005.tb03644.x. PubMed PMID: WOS:000230410300011.

1204. Parola P, Davoust B, Raoult D. Tick- and flea-borne rickettsial emerging zoonoses. Vet Res. 2005;36(3):469-92. Epub 2005/04/23. doi: 10.1051/vetres:2005004. PubMed PMID: 15845235.

1205. Pedra JH, Sukumaran B, Carlyon JA, Berliner N, Fikrig E. Modulation of NB4 promyelocytic leukemic cell machinery by Anaplasma phagocytophilum. Genomics. 2005;86(3):365-77. Epub 2005/07/12. doi: 10.1016/j.ygeno.2005.05.008. PubMed PMID: 16005178.

1206. Poitout FM, Shinozaki JK, Stockwell PJ, Holland CJ, Shukla SK. Genetic variants of Anaplasma phagocytophilum infecting dogs in western Washington State. Journal of Clinical Microbiology. 2005;43(2):796-801. doi: 10.1128/jcm.43.2.796-801.2005. PubMed PMID: WOS:000227045600042.

1207. Portillo A, Santos AS, Santibáñez S, Pérez-Martínez L, Blanco JR, Ibarra V, et al. Detection of a non-pathogenic variant of Anaplasma phagocytophilum in Ixodes ricinus from La Rioja, Spain. Ann N Y Acad Sci. 2005;1063:333-6. Epub 2006/02/17. doi: 10.1196/annals.1355.053. PubMed PMID: 16481536.

1208. Radzijevskaja J, Indriulytė R, Paulauskas A, Ambrasienė D, Turčinavičienė J. Genetics Polymorphism Study of Ixodes Ricinus L. Populations in Lithuania using RAPD Markers. Acta Zoologica Lituanica. 2005;15(4):341-8. doi: 10.1080/13921657.2005.10512699.

1209. Reisinger EC, Fritzsche C, Tomaso H, Gasser R. Tick transmitted diseases. Medizinische Welt. 2005;56(1-2):25-9.

1210. Rolain JM, Gouriet F, Brouqui P, Larrey D, Janbon F, Vene S, et al. Concomitant or consecutive infection with Coxiella burnetii and tickborne diseases. Clinical Infectious Diseases. 2005;40(1):82-8. doi: 10.1086/426440. PubMed PMID: WOS:000227492300013.

1211. Ruscio M. Laboratory medicine response in transmitted tick-borne diseases. Rivista Italiana della Medicina di Laboratorio. 2005;1(SUPPL. 3):103-7.

1212. Rymaszewska A. Identification of Anaplasma phagocytophilum on the basis of a fragment of the 16S rDNA gene. Folia Biologica-Krakow. 2005;53(3-4):199-203. doi: 10.3409/173491605775142765. PubMed PMID: WOS:000234096300016.

1213. Sarih MH, M'Ghirbi Y, Bouattour A, Gern L, Baranton G, Postic D. Detection and identification of Ehrlichia spp. in ticks collected in Tunisia and Morocco. Journal of Clinical Microbiology. 2005;43(3):1127-32. doi: 10.1128/jcm.43.3.1127-1132.2005. PubMed PMID: WOS:000227538900019.

1214. Schulze TL, Jordan RA, Schulze CJ, Mixson T, Papero M. Relative encounter frequencies and prevalence of selected Borrelia, Ehrlichia, and Anaplasma infections in Amblyomma americanum and Ixodes scapularis (Acari : Ixodidae) ticks from central New Jersey. Journal of Medical Entomology. 2005;42(3):450-6. doi: 10.1603/0022-2585(2005)042[0450:Refapo]2.0.Co;2. PubMed PMID: WOS:000229067400035.

1215. Scorpio DG, Von Loewenich FD, Bogdan C, Dumler JS. Innate immune tissue injury and murine HGA - Tissue injury in the murine model of granulocytic anaplasmosis relates to host innate immune response and not pathogen load. In: Hechemy KE, Oteo JA, Raoult DA, Silverman DJ, Blanco JR, editors. Rickettsioses: From Genome to Proteome, Pathobiology, and Rickettsiae as an International Threat. Annals of the New York Academy of Sciences. 10632005. p. 425-8.

1216. Sebaihia M, Thomson NR, Crossman L, Parkhill J. Livelihood hazards. Nature Reviews Microbiology. 2005;3(4):278-9. doi: 10.1038/nrmicro1132.

1217. Singu V, Liu H, Cheng CM, Ganta RR. Ehrlichia chaffeensis expresses macrophage- and tick cell-specific 28-kilodalton outer membrane proteins. Infection and Immunity. 2005;73(1):79-87. doi: 10.1128/iai.73.1.79-87.2005. PubMed PMID: WOS:000226037700007.

1218. Sirigireddy KR, Ganta RR. Multiplex detection of Ehrlichia and Anaplasma species pathogens in peripheral blood by real-time reverse transcriptase-polymerase chain reaction. Journal of Molecular Diagnostics. 2005;7(2):308-16. doi: 10.1016/s1525-1578(10)60559-4. PubMed PMID: WOS:000228736900020.

1219. Skarphedinsson S, Jensen PM, Kristiansen K. Survey of tickborne infections in Denmark. Emerging Infectious Diseases. 2005;11(7):1055-61. doi: 10.3201/eid1107.041265. PubMed PMID: WOS:000230106600011.

1220. Sparagano OAE. Impact of ticks and tick-borne diseases on agriculture and human populations in Europe. Journal of Agricultural Science. 2005;143:463-8. doi: 10.1017/s0021859605005526. PubMed PMID: WOS:000235201600003.

1221. Spencer BR, Johnson S, Nguyen ML, Cable RG, Rios J, Leiby DA. Longitudinal study of exposure to human granulocytic ehrlichiosis in healthy blood donors. Transfusion. 2005;45(3):16A-A. PubMed PMID: WOS:000231807600054.

1222. Sréter T, Kálmán D, Sréterné Lancz Z, Széll Z, Egyed L. [Babesia microti and Anaplasma phagocytophilum: two emerging zoonotic pathogens in Europe and Hungary]. Orv Hetil. 2005;146(13):595-600. Epub 2005/04/29. PubMed PMID: 15856623.

1223. Sréter T, Sréterné Lancz Z, Széll Z, Egyed L. [Rickettsia helvetica: an emerging tick-borne pathogen in Hungary and Europe]. Orv Hetil. 2005;146(50):2547-52. Epub 2006/01/31. PubMed PMID: 16440500.

1224. Sreter-Lancz Z, Sreter T, Szell Z, Egyed L. Molecular evidence of Rickettsia helvetica and R-monacensis infections in Ixodes ricinus from Hungary. Annals of Tropical Medicine and Parasitology. 2005;99(3):325-9. doi: 10.1179/136485905x28027. PubMed PMID: WOS:000228727300011.

1225. Stanek G. [Tick-borne pathogens in Central Europe]. Wien Klin Wochenschr. 2005;117(11-12):373-80. Epub 2005/08/02. doi: 10.1007/s00508-005-0368-1. PubMed PMID: 16053190.

1226. Stricker RB, Lautin A, Burrascano JJ. Lyme disease: point/counterpoint. Expert Rev Anti Infect Ther. 2005;3(2):155-65. Epub 2005/05/28. doi: 10.1586/14787210.3.2.155. PubMed PMID: 15918774.

1227. Stuen S, Whist SK, Bergström K, Moum T. Possible exclusion of genotypes in Anaplasma phagocytophilum-infected lambs. Veterinary Record. 2005;156(16):518-20. doi: 10.1136/vr.156.16.518.

1228. Sukumaran B, Carlyon JA, Cai JL, Berliner N, Fikrig E. Early transcriptional response of human neutrophils to Anaplasma phagocytophilum infection. Infect Immun. 2005;73(12):8089-99. Epub 2005/11/22. doi: 10.1128/iai.73.12.8089-8099.2005. PubMed PMID: 16299303; PubMed Central PMCID: PMCPMC1307096.

1229. Tarello W. Microscopic and clinical evidence for Anaplasma (Ehrlichia) phagocytophilum infection in Italian cats. Veterinary Record. 2005;156(24):772-4. doi: 10.1136/vr.156.24.772. PubMed Central PMCID: PMCMerial.
[truncated: 1,149,957 more chars]
