# Supplementary material for: Human granulocytotropic anaplasmosis—A systematic review and analysis of the literature
Source: PLoS Negl Trop Dis. 2024 Aug 5;18(8):e0012313. doi: 10.1371/journal.pntd.0012313 (PMC11326711; doi:10.1371/journal.pntd.0012313)
Supplement: S5 Text — (DOCX) [file pntd.0012313.s005.docx]

**Analysis of HGA CRID with coinfection(s)**

***Signs and symptoms***

For 108 of the 138 HGA CRID with coinfection(s), data on signs and symptoms was available. S1 Fig shows the frequency of the most commonly reported signs and symptoms.

**S1 Fig Frequency of signs and symptoms in human granulocytotropic anaplasmosis cases with coinfection (n=108 cases).**

**
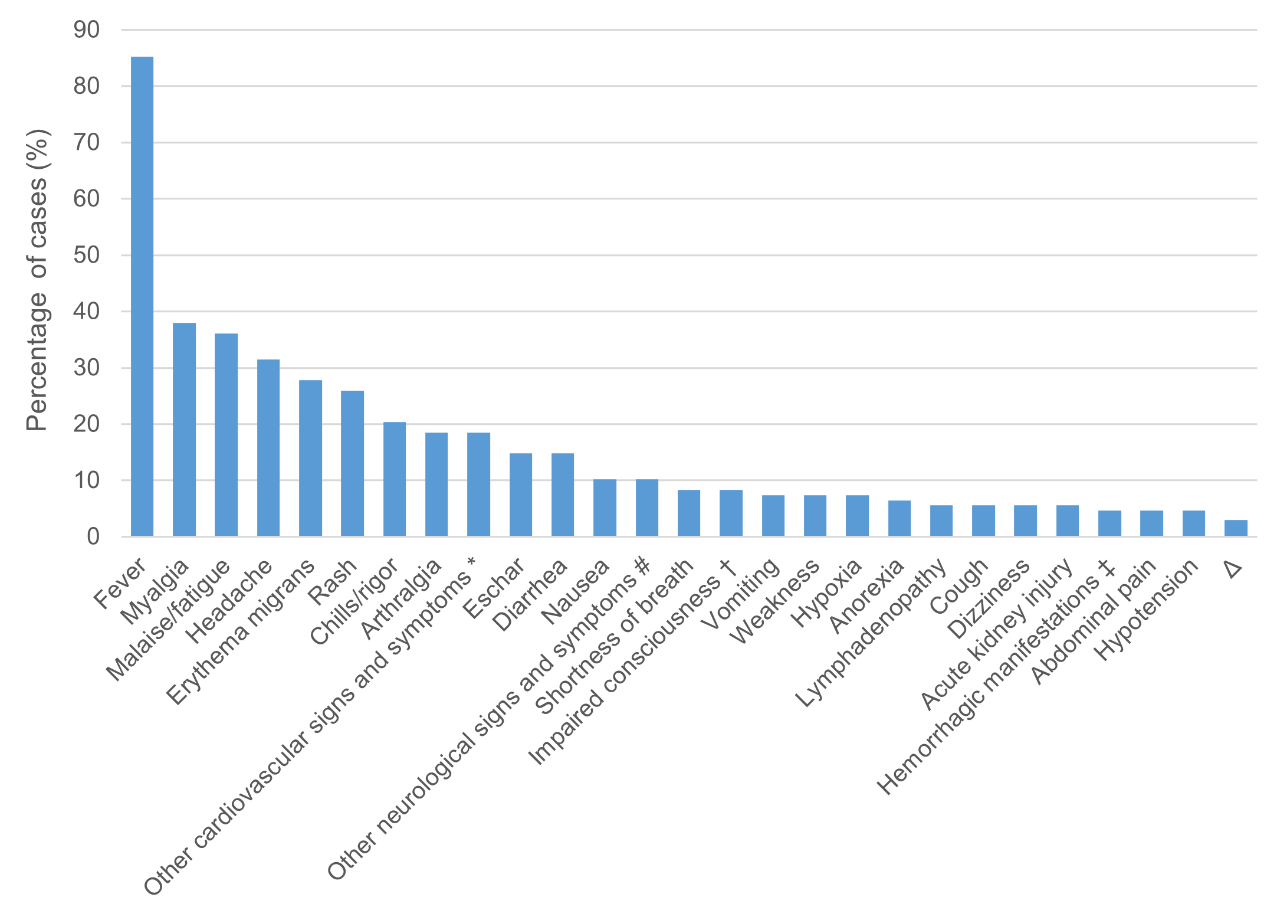
**

* other cardiovascular signs and symptoms: relative bradycardia (55.0%); irregular pulse (20.0%); irregular pulse plus heart failure (10.0%); and new murmurs (10.0%); heart failure (5.0%).

^#^ other neurological signs and symptoms: unspecified symptoms of neuritis (18.2%); pain + reduced sensation and mild paresis of one arm (9.1%); tremors (9.1%); aphasia (9.1%); one-sided weakness (9.1%); complex partial seizures + symmetric hyperreflexia (9.1%); facial paresis + horizontal diplopia (9.1%); limb paralysis (9.1%); paresthesia of the hands (9.1%); dysarthria (9.1%).

^†^ definition: altered mental state, confusion, somnolence, delirium, coma

^‡^ hemorrhagic manifestations: hematuria (40.0%); petechiae (20.0%); gingival bleeding (20.0%); epistaxis + bleeding from the mouth (20.0%).

^Δ^ Other rare signs and symptoms not included in the figure: 2-3%: Meningism (definition: headache plus neck stiffness and/or photophobia; 1-1.9%: sweats, conjunctivitis; <1%: hepatosplenomegaly.

***Laboratory findings***

Of the 138 HGA CRID with coinfection(s), data on laboratory findings was available for 91 cases. S2 Fig shows the frequency and S1 Table the median values and ranges of the most common abnormal findings in HGA CRID with coinfection(s).

**S2 Fig Abnormal laboratory findings related to human granulocytotropic anaplasmosis with coinfection (n=91 cases).**

**
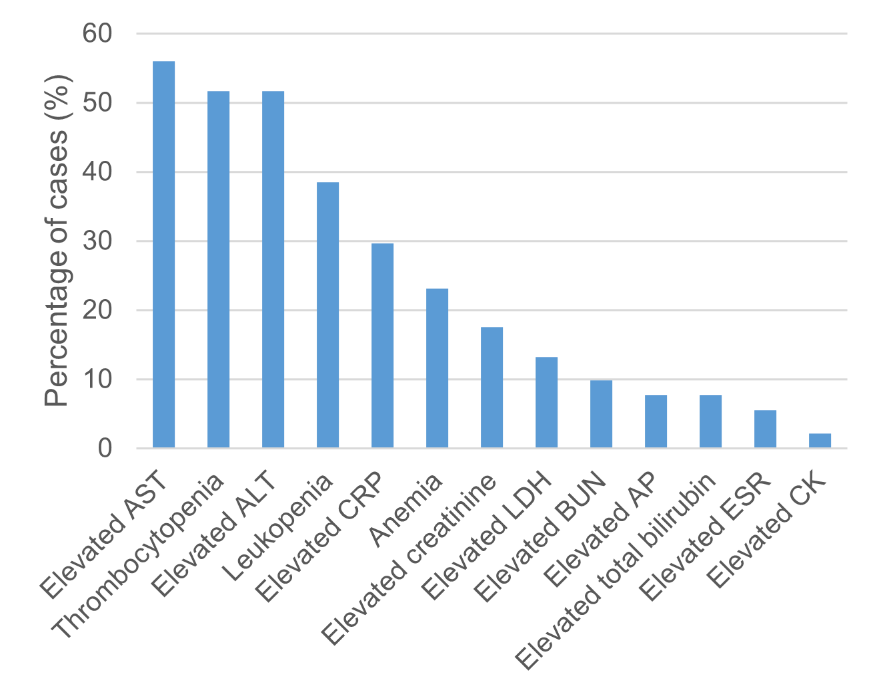
**

ALT, alanine aminotransferase; AP, alkaline phosphatase; AST, aspartate aminotransferase; BUN, blood urea nitrogen; CK, creatine kinase; CRP, C-reactive protein; ESR, erythrocyte sedimentation rate; LDH, lactate dehydrogenase.

**S1 Table. Abnormal laboratory findings in human granulocytotropic anaplasmosis cases with coinfection (n=2-55)**

| Laboratory parameters | **Median (range)** | **Range** | **Data available (n)** |
| --- | --- | --- | --- |
| Leukocytes (10^3^/µl) | 4.9 | 0.8-27.3 | 55 |
| Thrombocytes (10^3^/µl) | 88 | 10-299 | 53 |
| Hemoglobin (g/dl) | 12.9 | 7.9-18 | 29 |
| Hematocrit (%) | 31 | 19-47 | 10 |
| AST (U/l) | 84 | 22-769 | 49 |
| ALT (U/l) | 83 | 15-410 | 49 |
| AP (U/l) | 124 | 54-314 | 12 |
| Total bilirubin (µmol/l) | 57 | 15-222 | 8 |
| CRP (mg/l) | 63 | 11-503 | 24 |
| ESR (mm/h) | 67 | 4-124 | 6 |
| LDH (U/l) | 408 | 145-1617 | 10 |
| BUN (mg/dl) | 40 | 13-166 | 11 |
| Creatinine (µmol/l) | 141 | 88-1052 | 15 |
| CK (U/l) | N.c. | 216-490 | 2 |

ALT, alanine aminotransferase; AP, alkaline phosphatase; AST, aspartate aminotransferase; BUN, blood urea nitrogen; CK, creatine kinase; CRP, C-reactive protein; ESR, erythrocyte sedimentation rate; LDH, lactate dehydrogenase; N.c., not calculated.

***Complication(s)***

Complications were reported in 30 (31.6%) of 95 HGA CRID with coinfection(s) (S2 Fig).

**S2 Fig Frequency of complications in HGA coinfection cases reported with individual data (n=95).**

**
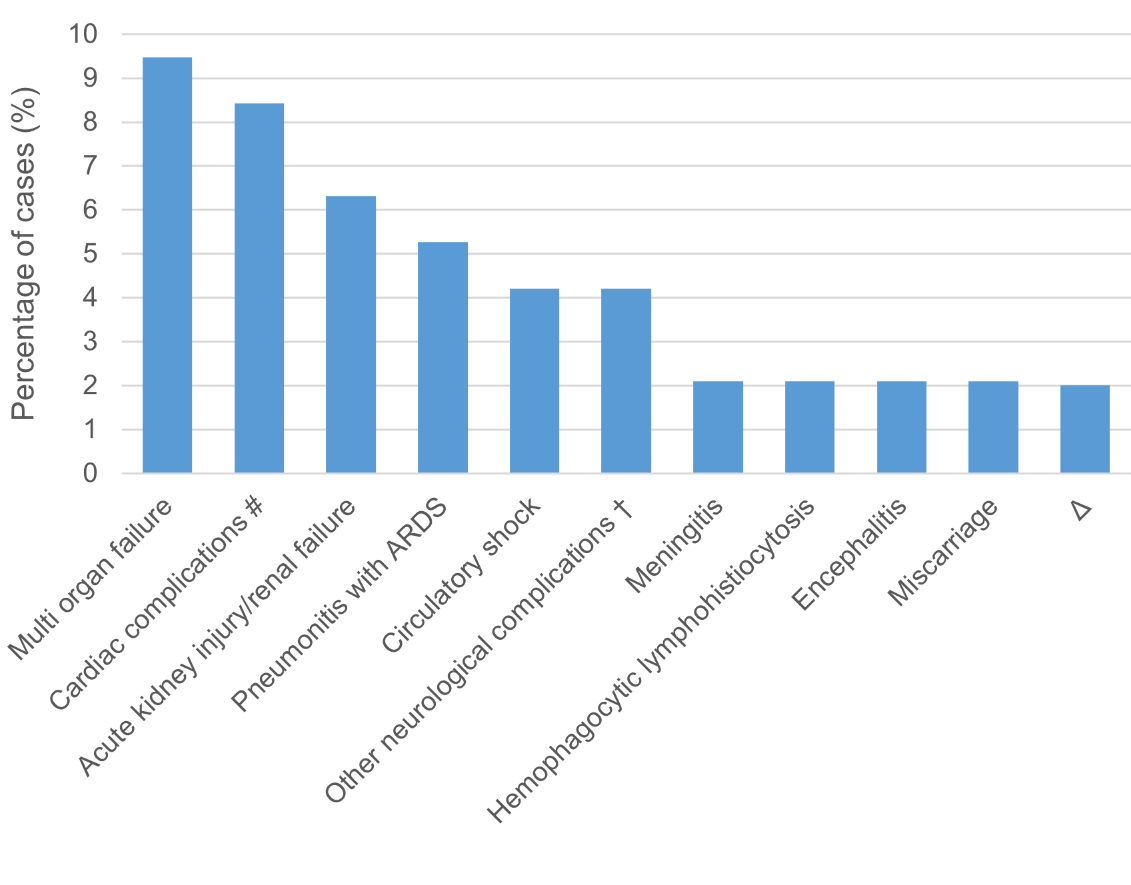
**

ARDS, acute respiratory distress syndrome.

* as in many cases multiple complications were concomitantly present, the number of complications exceeds the number of cases.

^#^ cardiac complications: cardiac arrhythmia (25.0%); cardiomyopathy and/or heart failure + cardiac arrhythmia (25.0%); cardiomyopathy and/or heart failure (12.5%); cardiomyopathy and/or heart failure + carditis (12.5%); carditis + cardiac arrhythmia (12.5%); and cardiomyopathy and/or heart failure + cardiac arrest (12.5%).

^†^ other neurologic complications: neuritis or myelitis (50%), and stroke (50%).

^Δ^ other more rare complications not included in the figure: 1-2%: pneumonitis without ARDS, secondary opportunistic infection with pulmonary aspergillosis, coagulopathy, hemorrhagic manifestations requiring transfusion, severe polyarthritis, splenic infarction, autoimmune adrenal insufficiency, and bone marrow failure.

***Outcome***

Data on the outcome was available for 95 of the HGA CRID with coinfection(s): 4 (4.2%) died due to acute complications related to their infections (S2 Table).

Of the survivors with respectively available data, 3 (3.2%) were reported to suffer from sequelae (S3 Table.).

**S2 Table. Reported fatal cases of human granulocytotropic anaplasmosis (n=4).**

| No. | Year of publication | Age of patient (years) | Sex of patient | Country of infection | Pre-existing medical condition | Immuno-suppressive treatment | Hospital admission | Time between first symptoms and medical presentation (days) | Presumed mode of transmission | Level of diagnostic certainty ^#^ | Coinfection(s) | Antimicrobial treatment | Time between presentation to hospital and specific therapy (days) | | Time from first symptoms to death (days) | | Complications/cause of death | | Ref. | |  |
| --- | --- | --- | --- | --- | --- | --- | --- | --- | --- | --- | --- | --- | --- | --- | --- | --- | --- | --- | --- | --- | --- |
| 1 | 2002 | 67 | Male | Czech Republic | Psoriatic arthritis | Corticosteroids and methotrexate | Yes | N.r. | Tick | A+ | *Borrelia garinii* | N.r. | | N.r. | | N.r. | | Multi-organ failure (renal failure, cardiomyopathy, endomyocarditis) | | [1] | |
| 2 | 2008 | 50 | Female | China | N.r. | Corticosteroids | Yes | 1 | Tick | D | SFTSV | Inappropriate antimicrobial treatment* | | N.r. | | N.r. | | Multi-organ failure, copious bleeding from nose and mouth | | [2] | |
| 3 | 2017 | 57 | Male | Greece | N.r. | N.r. | Yes | 31 | Tick | A+ | Influenza | Doxycycline | | 3 | | 51 | | HLH, multi-organ failure (acute renal failure, ARDS) | | [3] | |
| 4 | 2023 | 81 | Male | USA | Ischemic cardiomyopathy | N.r. | Yes | N.r. | Tick | A+ | *B. burgdorferi* | Doxycycline | | N.r. | | N.r. | | Cardiogenic and septic shock requiring vasopressor support, stroke | | [4] | |

ARDS, acute respiratory distress syndrome; HLH, hemophagocytic lymphohistiocytosis; N.r., none/not reported; Ref., reference; SFTSV, severe fever with thrombocytopenia syndrome virus; USA, United States of America.

* inappropriate antimicrobial treatment included amoxicillin, ceftriaxone, cefazolin, cefepime, gentamicin, metronidazole, clindamycin.

^#^ A+, diagnosed by PCR, culture and/or immunostaining of biopsy/autopsy tissue; D, clinical diagnosis.

**S3 Table. Reported sequelae of human granulocytotropic anaplasmosis (n=3).**

| No. | Year of publication | Age of patient (years) | Sex of patient | Country of infection | Pre-existing medical condition | Immuno-suppressive treatment | Time between first symptoms and medical presentation (days) | Level of diagnostic certainty^†^ | Coinfection(s) | Antimicrobial therapy | Time between presentation to hospital and specific therapy (days) | Complications | Sequelae | Ref. |
| --- | --- | --- | --- | --- | --- | --- | --- | --- | --- | --- | --- | --- | --- | --- |
| 1 | 2007 | 41 | Male | USA | N.r. | N.r. | 7 | A+ | *E. chaffeensis* | Doxycycline | N.r. | Encephalopathy | At 6 months MRI markedly improved but persisting short-term memory impairment and rare seizures | [5] |
| 2 | 2019 | 26 | Female | USA | Sickle cell disease with functional hyposplenism/asplenia | N.r. | N.r. | B+ | *Babesia* spp. | Doxycycline | N.r. | Sickle-cell crisis, multi-organ failure (ARDS, ARF, heart failure bone marrow failure), hypoxic brain injury | Permanent hypoxic brain injury demanding placement in long-term care facility | [6] |
| 3 | 2021 | 70 | Male | USA | N.r. | N.r. | N.r. | B | *Borrelia burgdorferi, Babesia microti* | Doxycycline | N.r. | N.r. | Symptoms of neuropathy at the site of his presumed tick bite at 1 year | [7] |

ARDS, acute respiratory distress syndrome; ARF, acute renal failure; MRI, magnet resonance imaging; N.r., none/not reported; Ref, reference; USA, United States of America.

^†^ A+, diagnosed by PCR, culture and/or immunostaining of biopsy/autopsy tissue; B+, diagnosed by microscopy of blood smear or buffy coat preparation; B, diagnosed by single IgG IFA or ELISA serology.

**References**

1. Hulinska D, Votypka J, Plch J, Vlcek E, Valesová M, Bojar M, et al. Molecular and microscopical evidence of Ehrlichia spp. and Borrelia burgdorferi sensu lato in patients, animals and ticks in the Czech Republic. New Microbiol. 2002;25(4):437-48. Epub 2002/11/20. PubMed PMID: 12437223.

2. Zhang L, Liu Y, Ni D, Li Q, Yu Y, Yu XJ, et al. Nosocomial transmission of human granulocytic anaplasmosis in China. Jama. 2008;300(19):2263-70. Epub 2008/11/20. doi: 10.1001/jama.2008.626. PubMed PMID: 19017912.

3. Tsiodras S, Spanakis N, Spanakos G, Pervanidou D, Georgakopoulou T, Campos E, et al. Fatal human anaplasmosis associated with macrophage activation syndrome in Greece and the Public Health response. J Infect Public Health. 2017;10(6):819-23. Epub 2017/02/13. doi: 10.1016/j.jiph.2017.01.002. PubMed PMID: 28189511.

4. Mahmoud AA, Abdelhay A, Eltaher B. Anaplasmosis and Lyme disease. Journal of Hematopathology. 2023;16(1):57-8. doi: 10.1007/s12308-022-00525-4.

5. Young NP, Klein CJ. Encephalopathy with seizures having PCR-positive Anaplasma phagocytophilum and Ehrlichia chaffeensis. Eur J Neurol. 2007;14(2):e3-4. Epub 2007/01/26. doi: 10.1111/j.1468-1331.2006.01582.x. PubMed PMID: 17250712.

6. Herbst J, Crissinger T, Baldwin K. Diffuse Ischemic Strokes and Sickle Cell Crisis Induced by Disseminated Anaplasmosis: A Case Report. Case Rep Neurol. 2019;11(3):271-6. Epub 2019/10/15. doi: 10.1159/000502567. PubMed PMID: 31607893; PubMed Central PMCID: PMCPMC6787427.

7. Grant L, Mohamedy I, Loertscher L. One man, three tick-borne illnesses. BMJ Case Rep. 2021;14(4). Epub 2021/04/18. doi: 10.1136/bcr-2020-241004. PubMed PMID: 33863772; PubMed Central PMCID: PMCPMC8055128.
