# Supplementary material for: Human granulocytotropic anaplasmosis—A systematic review and analysis of the literature
Source: PLoS Negl Trop Dis. 2024 Aug 5;18(8):e0012313. doi: 10.1371/journal.pntd.0012313 (PMC11326711; doi:10.1371/journal.pntd.0012313)
Supplement: S6 Text — (DOCX) [file pntd.0012313.s006.docx]

**Analysis of HGA infection cases reported with non-individual data (CRNID)**

Because of the often cumulative/pooled reported data of CRNID (i.e., case series, cohorts etc.), the analysis was more limited compared with CRID.

For 1346 cases, the patient’s sex was reported: 744 (55.3%) were male, 602 (44.7%) were female.

Immunocompromisation was reported for 3 cases (unspecified).

The suspected route of transmission was reported in 1129 cases: 1128 (99.9%) tick-borne, 1 (0.1%) direct contact to deer blood. Among 163 patients actively assessed for the history of a tick bite, 105 (64.4%) recalled a tick bite. In 542 (98.5%) of 550 cases with respectively available data, outdoor activities were reported as risk factor for HGA. For 368 cases the outdoor activities were specified: 342 were occupational and 26 were recreational.

Of the 2330 HGA CRNID cases, data on whether they were symptomatic or asymptomatic was available for 1936: 1927 (99.5%) were symptomatic, 9 (0.5%) were asymptomatic. Hospitalization was reported for 627 (60.8%) of 1032 CRNID with the respectively available data.

***Signs and symptoms***

For 1229 of the 2330 CRNID (Fig 5), data on signs and symptoms was available. S3 Fig shows the frequency of the most commonly reported symptoms. The less frequently reported symptoms not included in the figure were jaundice (32 cases), facial swelling (20 cases), oliguria (19 cases), relative bradycardia (15 cases), sore throat (9 cases), ascites (9 cases), and paresthesia (7 cases).

**S3 Fig Signs and symptoms of cases of human granulocytotropic anaplasmosis reported with non-individual data (n=1229 cases).**

**
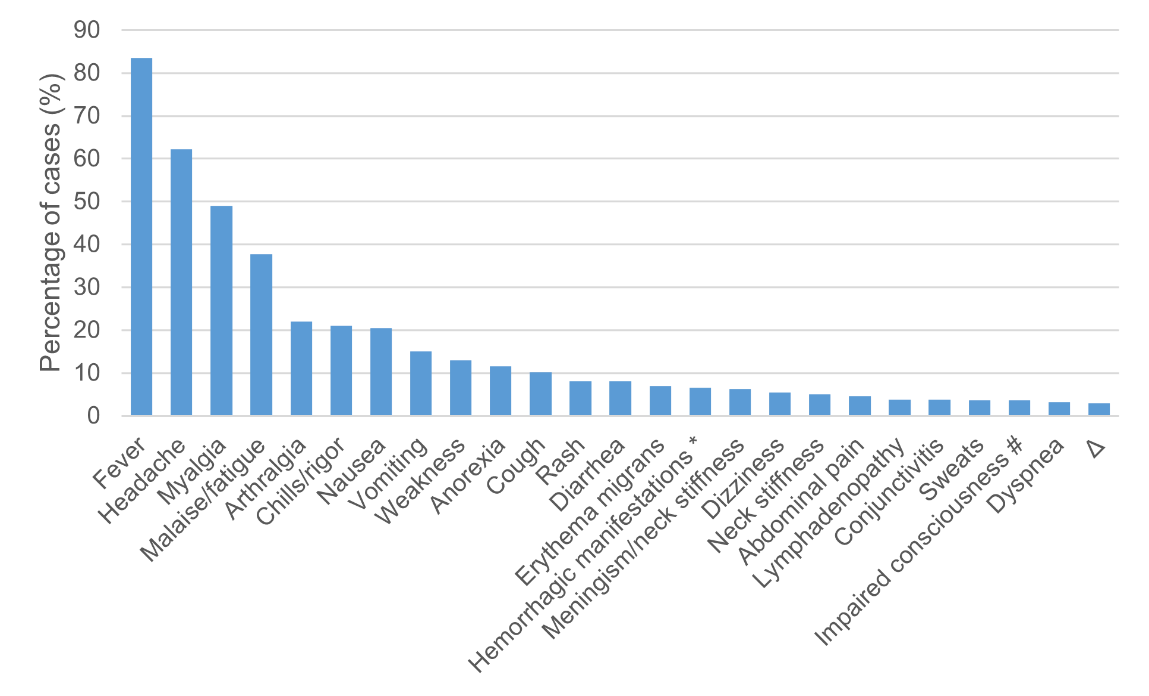
**

* hemorrhagic manifestations: ecchymosis (48.8%); internal bleeding including gastrointestinal bleeding (20.0%); epistaxis (17.5%); and petechiae (13.8%).

^#^ = altered mental state, confusion, somnolence, delirium, coma

^Δ^ more rare signs and symptoms not included in the figure: 2-3%: eschar, jaundice, vertigo; 1-1.9%: hepatosplenomegaly, facial swelling, oliguria, relative bradycardia; <1%: sore throat, ascites, paresthesia.

***Laboratory findings***

S4 Fig shows the frequency of the most common abnormal findings in CRNID.

**S4 Fig Laboratory findings in human granulocytotropic anaplasmosis CRNID (n=906 cases).**

**
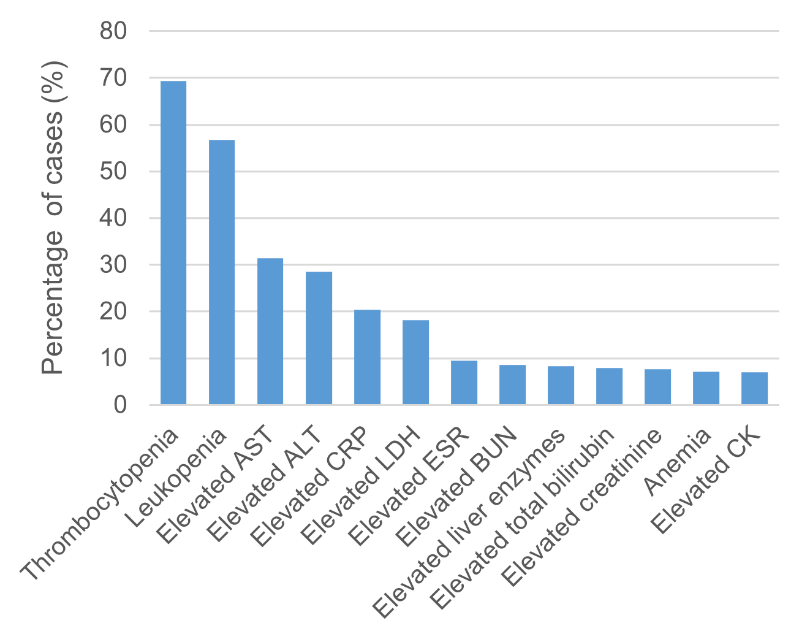
**

ALT, alanine aminotransferase; AST, aspartate aminotransferase; BUN, blood urea nitrogen; CK, creatine kinase; CRNID, cases reported with non-individual data; CRP, C-reactive protein; ESR, erythrocyte sedimentation rate; LDH, lactate dehydrogenase.

***Complications***

Data on complications were available for 182 of the 2330 CRNID (S4 Fig). Complications were reported in 94 (51.6%) of cases and specified in 71 cases. 68 of these 71 specified cases are reported by two Chinese studies: systemic inflammatory response syndrome (SIRS) in 17 cases, multi-organ dysfunction syndrome (MODS) in 30 cases, and concomitantly present SIRS and MODS in 21 cases. The other 3 cases were: syncope (n=1), renal failure plus polymyositis plus meningitis (n=1), and severe thrombocytopenia plus subdural hematoma (n=1).

***Treatment***

Data on antimicrobial treatment was available for 536 of the analyzed 2330 HGA CRNID. 383 (71.4%) cases received antimicrobial treatment, 25 (4.7%) cases received no antimicrobial treatment, and for 128 (23.9%) it was unclear whether they received antimicrobial treatment. Of the 383 cases receiving antimicrobial treatment, 347 (90.6%) received appropriate treatment for HGA, 14 (3.7%) did not receive appropriate antimicrobial treatment for HGA, and in 22 (5.7%) cases it was not clear whether antimicrobial treatment was appropriate. Of the appropriately treated cases, 331 (95.4%) received doxycycline and 16 (4.6%) cases received tetracycline.

***Outcome***

Data on the outcome was available for 1339 of the HGA CRNID: 39 (2.9%) died (in 37 cases death was reported HGA-related, in 2 cases it was unclear whether death was HGA-related or due to another cause) and 1300 (97.1%) survived. Of the survivors with respectively available data (n=328), 326 (99.4%) fully recovered and 2 (0.6%) suffered from sequelae (unspecified).
