# Supplementary material for: Human granulocytotropic anaplasmosis—A systematic review and analysis of the literature
Source: PLoS Negl Trop Dis. 2024 Aug 5;18(8):e0012313. doi: 10.1371/journal.pntd.0012313 (PMC11326711; doi:10.1371/journal.pntd.0012313)
Supplement: S1 Table — (DOCX) [file pntd.0012313.s007.docx]

| **Screening and Selecting Tool – Data extraction Sheet** | | | | | | | |
| --- | --- | --- | --- | --- | --- | --- | --- |
| **Reviewer name:** | | | | **Date:** | | | |
| **Author name/type of study/country:** | | | | **Year:** | | | |
| **Title:** | | | | **Journal:** | | | |
| **Studies excluded because:** | | | | | | | |
| Not related to human anaplasmosis | Language | Unsuitable study type (e.g. Review) | Duplicate | Insufficient data | Seroprevalence only, no clinical cases | Related to veterinary anaplasmosis | Not retrievable |
|  | **Number of HA cases reported in the reference** | | | | | | |
|  | **Number of cases already reported** | | | | | | |
|  | **Cohort study: (HA out of how many cases of what)** | | | | | | |
|  | **Running patient No.** | | | | | | |
|  | **Patient specificity of data** | | | | | | |
|  | **No data on epidemiology** | | | | | | |
|  | **Patient's age** | | | | | | |
|  | **Patient's sex** | | | | | | |
|  | **Most likely country of acquisition** | | | | | | |
|  | **Most likely province of acquisition** | | | | | | |
|  | **If imported: Time between end of trip and symptoms** | | | | | | |
|  | **Country of Diagnosis** | | | | | | |
|  | **Province of Diagnosis** | | | | | | |
|  | **Autochthonous or imported case** | | | | | | |
|  | **Occupational/ recreational risk factor for tick bite** | | | | | | |
|  | **Year of acquisition** | | | | | | |
|  | **Pre-existing conditions** | | | | | | |
|  | **Immunocompromised** | | | | | | |
|  | **Pregnancy** | | | | | | |
|  | **If pregnancy: which week** | | | | | | |
|  | **No data on symptoms** | | | | | | |
|  | **Symptomatic /asymptomatic** | | | | | | |
|  | **Hospital admission** | | | | | | |
|  | **Time between first symptoms and presentation to hospital/physician** | | | | | | |
|  | **Duration of Hospital stay** | | | | | | |
|  | **Data on individual symptoms available** | | | | | | |
|  | **Duration of Symptoms (fever)** | | | | | | |
|  | **Fever** | | | | | | |
|  | **Highest temperature measured** | | | | | | |
|  | **Chills/rigor** | | | | | | |
|  | **Sweats** | | | | | | |
|  | **Malaise/fatigue** | | | | | | |
|  | **Rash** | | | | | | |
|  | **Presence of eschar, erythema migrans** | | | | | | |
|  | **Headache** | | | | | | |
|  | **Myalgia** | | | | | | |
|  | **Arthralgia** | | | | | | |
|  | **Lymphadenopathy** | | | | | | |
|  | **Nausea** | | | | | | |
|  | **Vomiting** | | | | | | |
|  | **Abdominal pain** | | | | | | |
|  | **Diarrhea** | | | | | | |
|  | **Anorexia** | | | | | | |
|  | **Hepatosplenomegaly** | | | | | | |
|  | **Cough** | | | | | | |
|  | **Shortness of breath** | | | | | | |
|  | **Confusion/ impaired consciousness** | | | | | | |
|  | **Meningeal symptoms** | | | | | | |
|  | **Neck stiffness** | | | | | | |
|  | **Neck pain** | | | | | | |
|  | **Photophobia** | | | | | | |
|  | **Other neurological signs and symptoms** | | | | | | |
|  | **Conjunctivitis** | | | | | | |
|  | **Dizziness** | | | | | | |
|  | **Vertigo** | | | | | | |
|  | **Sore throat** | | | | | | |
|  | **Chest pain** | | | | | | |
|  | **Weakness** | | | | | | |
|  | **Hypotension** | | | | | | |
|  | **Other cardiovascular signs and symptoms** | | | | | | |
|  | **Other symptoms** | | | | | | |
|  | **Duration between onset Fever and appropriate Treatment** | | | | | | |
|  | **Specify presumed vector of disease** | | | | | | |
|  | **Tick bite remembered** | | | | | | |
|  | **Tick species** | | | | | | |
|  | **Duration between bite and symptoms** | | | | | | |
|  | **No data on diagnostics** | | | | | | |
|  | **Serology** | | | | | | |
|  | **Days between acute and convalescent sample** | | | | | | |
|  | **PCR** | | | | | | |
|  | **Blood smear or buffy coat microscopy** | | | | | | |
|  | **Percentage of infected granulocytes** | | | | | | |
|  | **Morulae in which cell line** | | | | | | |
|  | **Culture** | | | | | | |
|  | **Biopsy** | | | | | | |
|  | **Level of diagnostic certainty** | | | | | | |
|  | **Time of first specific diagnostic test** | | | | | | |
|  | **Additional diagnostics** | | | | | | |
|  | **Anaplasma species** | | | | | | |
|  | **Coinfections** | | | | | | |
|  | **Diagnosis given initially** | | | | | | |
|  | **Data on laboratory values available** | | | | | | |
|  | **Leukopenia acc. author** | | | | | | |
|  | **Leucopenia: exact value** | | | | | | |
|  | **Specify Leucopenia** | | | | | | |
|  | **Thrombocytopenia acc. author** | | | | | | |
|  | **Thrombocytopenia: exact value** | | | | | | |
|  | **Anemia acc. author** | | | | | | |
|  | **Hemoglobin** | | | | | | |
|  | **Hematocrit** | | | | | | |
|  | **Elevated liver enzymes (at least one)** | | | | | | |
|  | **(AST) Aspartate Aminotransferase elevated** | | | | | | |
|  | **AST: exact value** | | | | | | |
|  | **(ALT) Alanine Aminotransferase elevated** | | | | | | |
|  | **ALT: exact value** | | | | | | |
|  | **(AP) Alkaline Phosphatase elevated** | | | | | | |
|  | **AP: Exact Value** | | | | | | |
|  | **Gamma-GT exact value** | | | | | | |
|  | **Total Bilirubin elevated** | | | | | | |
|  | **Bilirubin: exact value** | | | | | | |
|  | **CRP elevated** | | | | | | |
|  | **CRP highest** | | | | | | |
|  | **D-Dimer elevated** | | | | | | |
|  | **D-Dimers** | | | | | | |
|  | **BSR elevated** | | | | | | |
|  | **BSR: exact value** | | | | | | |
|  | **Procalcitonin** | | | | | | |
|  | **Elevated LDH** | | | | | | |
|  | **LDH: exact value** | | | | | | |
|  | **Blood urea nitrogen elevated** | | | | | | |
|  | **Blood urea nitrogen exact value** | | | | | | |
|  | **Creatinine elevated** | | | | | | |
|  | **Creatinine: exact value** | | | | | | |
|  | **Elevated CK** | | | | | | |
|  | **Creatine Kinase: exact value** | | | | | | |
|  | **Ferritin** | | | | | | |
|  | **Sodium** | | | | | | |
|  | **Albumin** | | | | | | |
|  | **CSF-findings** | | | | | | |
|  | **Other laboratory findings** | | | | | | |
|  | **No data on treatment** | | | | | | |
|  | **Received antibiotics** | | | | | | |
|  | **Appropriate antibiotics given** | | | | | | |
|  | **Time of appropriate antibiotics** | | | | | | |
|  | **Compound** | | | | | | |
|  | **Empirical antibiotic therapy** | | | | | | |
|  | **Time between presentation to hospital and specific therapy** | | | | | | |
|  | **Time until afebrile after start app. AB-Th** | | | | | | |
|  | **Duration of AB-Th** | | | | | | |
|  | **Dosage of AB-Th** | | | | | | |
|  | **Side Effects of treatment** | | | | | | |
|  | **Other treatment** | | | | | | |
|  | **No data on outcome** | | | | | | |
|  | **Complications** | | | | | | |
|  | **Specify complications** | | | | | | |
|  | **Outcome** | | | | | | |
|  | **Specify cause of death** | | | | | | |
|  | **Time from symptom onset to death** | | | | | | |
|  | **Specify sequelae** | | | | | | |
|  | **Info on last follow-up** | | | | | | |
|  | **Comment on relevant / interesting aspects** | | | | | | |
