# Supplementary material for: Human granulocytotropic anaplasmosis—A systematic review and analysis of the literature
Source: PLoS Negl Trop Dis. 2024 Aug 5;18(8):e0012313. doi: 10.1371/journal.pntd.0012313 (PMC11326711; doi:10.1371/journal.pntd.0012313)
Supplement: Attachment — Submitted filename: answerstoreviewers.docx [file pntd.0012313.s010.docx]

**Before we address the points raised by the two reviewers, we would like to thank them warmly for taking the time and obvious effort to provide us with a wealth of corrections, critical comments and constructive remarks! A cordial thanks for this highly appreciated support!**

**Methods**

Reviewer #2: Specific points:

Page 7, line 94: The citation regarding incidence of HGA in the US covered only 2008-2012. The peak of HGA cases in the US was until recently 2017, and now 2021. Why not update this with the data that is readily available on the CDC website?

**Response: Thank you very much for this helpful hint! We have updated the paragraph accordingly. Unfortunately, we have not found any more recent incidence data and have therefore continued to list the incidence data from the past reports.**

Page 7, line 96. Spelling error, should be “tick-borne”

**Response: The spelling error was corrected.**

Page 7, line 99. H. concinna has also been implicated as a potential vector in China.

**Response: *H. concinna* was added to the text.**

Page 8, Table 1. Same comment as above

**Response: *H. concinna* was added to the table.**

Page 8, HGA has been identified associated with organ transplantation (please see Transpl Infect Dis. 2019 Aug;21(4):e13129. doi: 10.1111/tid.13129)

**Response: (i) in response to the critic to not include statements or comparisons with HME elsewhere, we rephrased from “Besides infections transmitted by ticks, cases of HME and HGA acquired by blood-transfusion (both HME and HGA) and organ transplantation (so far HME only) have been reported.” to “Besides infections transmitted by ticks, cases of HGA acquired by blood-transfusion have been reported.” (ii) in response to the note that HGA has been identified associated with organ transplant and the quoted reference we also consider this phrasing correct as in the referenced case HGA was “associated” but not “transmitted” through the organ transplantation. This also is true for the other reviewed HGA cases with a history of organ transplantation.**

Page 8, line 120. Most existing data suggest that rash is rare and when present often reflects erythema migrans after B. burgdorferi coinfection (which is also infrequent).

**Response: We have changed the wording from "...sometimes a rash is present" to "...rarely a rash is present". Also, the section in the discussion dealing with rash in HGA has been updated to reflect the other point regarding rash raised below.**

Page 8, line 122. Similarly, although reported, a critical evaluation does not often support a clear diagnosis of meningoencephalitis because of lack of objective findings for that diagnosis.

**Response: We removed meningoencephalitis as suggested.**

Page 9, I was confused about why HME was being discussed, so consider elaborating about the purpose of the introductory comments. I didn’t understand this until I read the entire intro.

**Response: also in line with the comment of reviewer #1 we removed any unnecessary mentioning and comparison to HME.**

Page 9, line 129. While this had been true in the US, it has not been widely described this way for quite awhile, and this is reflected in the nomenclature used by the CDC, and in major texts and online resources (e.g. UpToDate).

**Response: We felt it necessary to address the issue of misnomers, especially for the understanding of readers who are unfamiliar with the topic and live outside the US**

Page 9, lines 138-139. True but in fact it is recommended directly by clinical suspicion alone.

**Response: to clarify, we rephrased from “…it is recommended to start empirical antimicrobial treatment before the results of the laboratory tests are available” to “…it is recommended to start empirical antimicrobial treatment immediately in case of clinical suspicion and regardless of diagnostic tests.”**

Page 9, lines 148-149. I don't understand this comment. The data on the 25-75% of patients for whom a blood smear will reveal morulae is from immunocompetent patients. Most now believe that rate is closer to 90-100% with an adequate blood smear evaluation. Thus, how could this be insensitive, and how would this justify the conclusion of a low bacterial burden, this is a relatively HIGH blood bacterial burden disease which is why PCR alone works so well.

**Response: we interpreted the wide ranges stated in the literature (20-80% Mandell chapter; 25-75% Ismail et al.) as “insensitive compared to other confirmatory tests…”.**

**We did not come across references suggesting 90-100%, but would be happy to receive and include such references.**

Page 11, Table 2. Column 3 (Disadvantages), Row “Serology”. Cross reactions with Rickettsia spp. is actually quite infrequent, it would be better to state cross reactivity with other Anaplasmataceae since such cross reactions with other rickettsial diseases is rare.
**Response: The text was edited as suggested.**

Reviewer #1: Specific points:
1. The meaning of "paraclinical" is unclear.

**Response: We apologize for this direct translation from German; in German, “paraclinical” is used to address “laboratory values/aspects”**. **We rephrased to clarify, respectively.**

2. The inappropriate term, liver function tests, is inaccurate. The mentioned tests are "liver injury tests".

**Response: We rephrased as suggested.**

3. HME was not reviewed systematically, and comparisons of HGA and HME are unnecessary and inappropriate in a systematic review.

**Response: we removed the unnecessary mentioning of and the comparison to HME as suggested.**

4. Table 2: What is meant by serum "scar"?

**Response: The term appears to be only existing in German (“Serumnarbe”) which means the persistence of detectable antibodies as a sign of past infection. We rephrased, respectively.**

5. Please provide a reference for the statement about serology: "decreased sensitivity after early administration of appropriate antibiotics".

**Response: This has been described in several sources (see sources in the table title), with data available for E. chaffeensis, whereas this has never been studied separately for A. phagocytophilum as far as we know. Since a reduced antibody response after early antibiotic therapy is known for ehrlichiosis and other rickettsiae, it can be assumed that this could be similar for anaplasmosis. The table text has been adjusted to reflect the fact that this is an assumption and not a proven fact.**

6. Awarding a certainty grade B for IFA or ELISA antibody titer of 64 is highly questionable.

**Response: We agree that a titer of 64 reflect good evidence of an acute infection. Nevertheless, this is the cut-off value for single titer serology that is widely used in the literature we reviewed. We have adapted the discussion on this topic to put more emphasis on this issue.**

7. Table 7: Reported co-infections are questionable, especially Bartonella, Rickettsia, Ehrlichia chaffeensis, Coxiella, Orientia, dengue virus, and chikungunya virus. How well documented are these as active co-infection versus seroprevalence?

**Response: We agree that some reported coinfections are questionable. We added the following comment to the table’s footer, respectively: * Note that the list of coinfections is based on the coinfections reported by the authors in the original publications, which, however, often cannot be checked for validity due to a lack of information on the corresponding diagnostics and therefore do not allow a conclusive assessment.**

**In addition, we added the following sentences to the discussion part: “The probability of correctly diagnosed co-infections must generally be viewed critically. If a pathogen has a regionally high prevalence and incidence, co-infection is plausible, even if the transmission route is different. However, if a pathogen is rare and the route of transmission not identic, the plausibility of co-infection is considerably lower. This is especially true if the level of diagnostic certainty of the applied tests is low.”**

**With regards to Bartonella we also added: “With regard to the reported co-infection cases of HGA with *Bartonella henselae*, it should be noted that although *Bartonella* spp. are not generally considered tick-borne pathogens, there is some evidence that they can be transmitted by ticks (References: https://jamanetwork.com/journals/jamaneurology/fullarticle/780359**

**https://doi.org/10.1111/j.1365-2915.2008.00713.x; https://doi.org/10.3390/microorganisms9050901; https://journals.asm.org/doi/10.1128/jcm.42.6.2799-2801.2004).”**

8. Table 8: How does microscopy distinguish between Ehrlichia ewingi and Anaplasma phagocytophilum, especially in states where Anaplasma is undetected?

**Response: Table 8 merely summarizes how the authors of the reviewed publications diagnosed HGA. The note that *E. ewingii* cannot be distinguished microscopically from *A. phagocytophilum* is of course absolutely correct and, thus we added “[of note: *A. phagocytophilum* cannot be differentiated from *Ehrlichia ewingii*, by microscopy as morulae of both species show a tropism for granulocytes]” to the footer of Table 8 and “…; limited specificity as morulae of *A. phagocytophilum* cannot be differentiated from morulae of *Ehrlichia ewingii* which also show a tropism for granulocytes” to clarify this point for the reader.**

9. Neologisms such as immunocompromization should be corrected to immunocompromised.

**Response: This was corrected as suggested.**

10. Clinical signs such as Bell's palsy and arthralgia are more likely due to a diagnosed or undiagnosed co-infection.

**Response: we fully agree and therefore added behind the note relativizing the association of HGA with rash (line >900): "The same is true for other symptoms highly suggestive of being caused by another pathogen (like e.g. in the case of Bell’s palsy or pronounced monoarthrisis where *B. burgdorferi* is more plausible) or symptoms which are rather untypical and, thus questionable in the context of HGA (e.g. cardiomyopathy/ heart failure, disseminated intravascular coagulation)."**

11. Lines 198: Please cite figure 8 not figure 3.

**Response: This was apparently misunderstood by the reviewer: figure 3 is referenced to clarify the origine of the cited number of patients. Figure 8 is concerning the laboratory findings.**

12 Figures 9 and 10 do not show "number of cases" as depicted in the y-axis.

**Response: We fully agree and corrected to “percentage of cases (%)”, respectively.**

13. Figure 9: What are the criteria used for diagnoses of cardiomyopathy, heart failure, and disseminated intravascular coagulation which seem to be very unlikely caused by Anaplasma.

**Response: As generally problematic when retrospectively analyzing 3^rd^ party data, one has to rely to a considerable degre on the source documents and the judgement of the authors. We absolutely agree that this is an acknowledged weakness and unavoidable issue limiting any systematic review. Nevertheless, with the now added points (see 10. above) we consider the issue sufficiently addressed.**

14. Lines 563-4: There are no antibiotics listed in Table 19.

**Response: The reference was corrected to Table 18.**

15. Line 632: The cases are primarily in the northeast not the northwest.

**Response: Corrected as suggested.**

16. Line 638: Are there any detections of Anaplasma phagocytophilum in vectors in South and Central America?

**Response: *A. phagocytophilum* has been found in some potential reservoir animals and vectors in South America and in potential reservoir animals in Central America (see DOI: 10.1186/s13071-023-05657-9, DOI: 10.3389/fvets.2018.00293, doi: 10.3389/fvets.2019.00164)**

17. Line 648: "Insensitive" is not the correct concept; "inadequate" would be more appropriate.

**Response: The wording was corrected as suggested.**

18. Line 671: Was the patient infected in Brazil or French Guiana?

**Response: He was infected in French Guiana as mentioned earlier in the text, while only the wild life isolates showing an isolate closely related to *Candidatus* Anaplasma sparouinense stemmed from Brazil. This was corrected.**

19. Line 741: Please correct the spelling of Anaplasma phagocytophilum.

**Response: Thanks! The spelling error was corrected.**

20. Lines of 788-790: Actually Lyme disease is currently spreading southward into eastern Tennessee and Western North Carolina.

**Response: Thanks for this hint!**

Reviewer #2: The analysis matched the plan. The results are generally clearly and completely presented, and the figures of good quality. Specific issues include:

Page 13, line 217. “immunosuppression” please define what this includes since the CDC reporting includes an array of predisposing conditions not classically felt to be immune compromising in the way that HIV, organ transplantation or immunosuppressive therapies are.

**Response: immunosuppression included (i) any medical treatment aiming at and leading to impaired immune defence (i.e. corticosteroids above substitution dosage, azathioprine, methotrexate, chemotherapy, biologics a.o.) and (ii) any clinical condition likely to impair immune defence (i.e. hematological malignancies and disorders, asplenia, HIV-infection with a below normal CD4-T-cell count, uncontrolled diabetes mellitus, end-stage kidney or liver disease).**

Page 13, lines 225-229. I am guessing that many of the individual case reports provided results from >1 approach named here. How were those classified? As is evident a grade A+ would be appropriate alone when another test was also done, but the lack of serconversion in such patients would argue for a possible false positive A+ method result. Additionally, those cases with >1 diagnostic from the categories of A and B+, might also be considered equivalent to A certainty. How these are handled is critical since the final analyses used should rely solely on the highest certainty testing, including that for coinfections, which as far as I can tell was not addressed.

**Response: We fully agree with the reviewer, but when setting the base for this review we had to decide on a methodology and did not consider such an elaborate integrated judgment. Next time we would surely consider and integrate such an approach.**

**We also want to note, that our aim was not to distil only the ultimate evidence from the literature (which is anyhow difficult with a retrospective data analysis of 3^rd^ party data) but to rather summarize the existing data in a more or less concise way. Always with the consideration that with large data sets, even individual erroneous and questionable data ultimately do not significantly distort the overall picture.**

**To answer the reviewer’s question and highlight the rather low numbers affected:**

**27 PCR-positive cases were negative by serology, whereby often only a single serology was performed. In 11 of these PCR-positive cases, morulae were detected.**

**45 cases had described morulae detection and a positive paired serology, 3 of these cases had a negative PCR (and for 20 of these cases no PCR was reported). These cases were counted in the A category.**

Page 13, line 230. CDC case definition of HGA. Please note that these diagnostic criteria are for surveillance purposes and not necessarily recommended for diagnosis. There is still ongoing debate about the application of diagnostics that should be recognized.

**Response: Thanks! Hint noted!**

Page 13, line 231. “IgM IFA” Please note that the CDC does NOT advocate the use of IgM serology for the diagnosis of HGA. Including this here might signify that this is an acceptable serodiagnostic approach when it is not.

**Response: We fully agree. We adjusted the wording regarding the validity of IgM in table 3 to “IgM detection lacks specificity to be useful as single means of diagnosis” to clarify this fact to the reader.**

Page 14, Table 3, Column 2 (Description), Row 7 (Serology – IgG). I personally do not agree with this application, although I know it appears on the CDC surveillance website; however, even there the CDC admits that these are surveillance criteria, not diagnostic. The issue here is best addressed in this publication: doi: 10.1128/JCM.40.7.2612-2615.2002

**Response: Thanks for the hint! We adjusted the wording in table 3 at several levels to hopefully now be more correct.**

Page 23, Table 7. To evaluate co-infections, this is a potentially misleading table since no grading system to asses certainty of a correct diagnosis was used for the coinfections. In my experience, this is a very significant problem that leads to the misimpression that co-infections are common. This document should strive to provide such data objectively rather than accepting the coinfection diagnoses in published literature. Additionally, the TBEV row is a bit confounded by the fact that TBEV does NOT occur in North America; thus using the denominator calculated by summing the numbers of all globally reported cases provides a misleading result and interpretation. Also, the B. burgdorferi s.l. + Bartonella henselae row is particularly troubling since B henselae is NOT tick-transmitted and there are NO CRID for this combination?? And finally, for the Ehrlichia chaffeensis row, is this even plausible?

**Response: We agree that some reported coinfections are questionable. We added the following comment to the table’s footer, respectively: * Note that the list of coinfections is based on the coinfections reported by the authors in the original publications, which, however, often cannot be checked for validity due to a lack of information on the corresponding diagnostics and therefore do not allow a conclusive assessment.**

**In addition, we added the following sentences to the discussion part: “The probability of correctly diagnosed co-infections must generally be viewed critically. If a pathogen has a regionally high prevalence and incidence, co-infection is plausible, even if the transmission route is different. However, if a pathogen is rare and the route of transmission not identic, the plausibility of co-infection is considerably lower. This is especially true if the level of diagnostic certainty of the applied tests is low.”**

**With regards to Bartonella we want to highlight that possible tick-transmission is well supported by the literature (see references below) which we added for completeness also to the discussion (“With regard to the reported co-infection cases of HGA with *Bartonella henselae*, it should be noted that although *Bartonella* spp. are not generally considered tick-borne pathogens, there is some evidence that they can be transmitted by ticks (References: https://jamanetwork.com/journals/jamaneurology/fullarticle/780359**

**https://doi.org/10.1111/j.1365-2915.2008.00713.x; https://doi.org/10.3390/microorganisms9050901; https://journals.asm.org/doi/10.1128/jcm.42.6.2799-2801.2004).”**

**The point that co-infections of HME and HGA are highly unlikely is well addressed and the 3 respective cases discussed in detail.**

Page 25, line 390. Do the analyses reflect the inclusion of ANY diagnostic criteria group or only the highest quality (A+)? Please describe this as relevant for each analysis, but preferably, select a single diagnostic group that provides high confidence in the diagnosis and use that for analysis. Anything less would introduce significant potentially troubling analyses and would lack confidence in the results and conclusions made.

**Response: To clarify this rightly raised point, we added to the method section: “We included data from all investigated HGA cases in the analyses, regardless of the level of evidence for a correct diagnosis. In order to determine whether the analyses performed might give a distorted picture corresponding to the differences in diagnostic accuracy, we carried out a subgroup analysis, provided that a sufficiently large number of cases and thus data volume was available, and compared the analysis of the A+ and A cases with the analysis of all cases.”**

**The analyses depicted in Figures 7,8,9 demonstrate that the A+/A cases show a pretty identical pattern compared to the analysis of all cases. This reinforces our assumption that larger data sets largely eliminate the problem of bias due to the possible inclusion of misclassified cases and that the results of our analyses are valid overall.**

Page 26, Table 10, hospitalization. It is interesting that the hospitalization rate is about twice as high in North America in these papers vs. what is accrued in case reports for the CDC. This indicates the bias of this approach, which is acknowledged in the Discussion. Please consider more emphasis on this bias in the abstract and conclusions.

**Response: We agree to emphasis the point of bias more and added “We consider the almost double as high hospitalization rate found in our analysis to accrue from reporting/publication bias and not reflect the true picture.” to the discussion section. At the end of the abstract we added “Overall, the data analyzed indicates a non-negligible bias in reporting/publication, so a certain degree of caution is required when generalizing the data.”.**

Page 27, lines 416-417. “vertical transmission” - you might clarify what this means, since it demonstrates the capacity for transplacental transmission in humans.

**Response: The point was specified in this sentence as well as in the discussion.**

Page 28, Table 11, patient no. 9, culprit blood produce – minor error, “RCB” should be “RBC”; also patient 11 had leukoreduced RBCs. I know and it’s in the paper.

**Response: Both points were corrected.**

Page 30, Table 12, patient 17, treatment category nomenclature. I did not review this manuscript, but if this is a quote, please use quotation marks; if not, please simply describe the antimicrobial treatment without judgement regarding "appropriateness".

**Response: We adapted the treatment category of patient 17 in table 12 as suggested, by adding the administered amoxicillin treatment of the patient.**

Page 39, lines 56-564. The substances considered "appropriate" are not listed in Table 19 as was indicated.

**Response: It should have said Table 18. This was corrected.**

Page 39, Table 18. Clearly this cannot be the entire list of antimicrobial agents used? Is this a representation of those drugs considered "appropriate" or effective?

**Response: Yes, the table is a representation of those drugs considered appropriate/effective. We renamed the table heading, respectively and added a clarifying list of the reported antimicrobials considered ineffective to the footer of the table.**

Page 45, Table 22. Patient no. 7, Complications column. “renal replacement therapy” What does this mean? Is this kidney transplant? If so, say that; if not known, please use an alternative phrase to indicate the renal therapy was not defined/identified.

**Response: Renal replacement therapy meant dialysis in all three cases. This was now specified to avoid confusion.**

--------------------

**Conclusions**

Reviewer #1: The interpretation of the data and accurate statements about the limitations and reservations about the interpretations are quite appropriate.

Reviewer #2: Conclusions are generally well-supported by the data, and limitations are discussed, but need some additional consideration especially with regard to the discrepancy between the overall conclusions discerned from major series publications and those that use CDC national data. This likely is the result of publication bias and although discussed, I think this needs considerably more emphasis. Also, the use of ALL data rather than that based on a diagnostic quality grading may be including data from cases erroneously classified as anaplasmosis. I would recommend an analysis of those cases that use only A or A+ diagnostic grade data and compare to the overall set or the set that is not A or A+ grade. The same situation is relevant for coinfections, which seem considerable higher than anticipated based on reputable publications that have directly sought this information, and is likely driven by publication bias for coinfections, some of which that were included that
are highly implausible and questionable, for which NONE of the coinfections was graded for quality. Additional individual concerns are:
Page 47, line 632. Places where majority of cases are reported in North America. First, this location refers to the U.S not North America, and most cases are reported from the Northeast and Upper Midwest, not the Northwest.

**Response: This inaccuracy was corrected.**

Page 47, line 638. About the cases from South America, especially Venezuela….for the authors awareness, I worked closely with the Venezuelan group considering this in platelets and found NO evidence of infection by any molecular method we used. They obviously found a way to publish without molecular data, but likely did not acknowledge the extensive negative data that had already been done. This is a reason to focus on the data within the trustworthy diagnostic group.

**Response: Thanks for the interesting insight!**

Page 49, lines 676-678. From my reading of this, there is no evidence that this Candidatus species even has the capacity to cause disease - the main topic of this systematic review.

**Response: We came to the very same conclusion (“it is unclear whether his symptoms in …were caused by Anaplasma”) but nevertheless found it worthwhile to mention the case as particularly this aspect broadens the perspective.**

Page 49, lines 680-681. in addition to the appropriate commentary about this, one must consider addressing the climate in the US and some European countries regarding the wide-spread belief among some active at co-infections are exceedingly common (>75-100% of infections diagnosed as some boutique "Lyme-literate" physician offices and laboratories advocate for this and often cosponsor misleading publications that appear to be valid. The 16% coinfection rate here is likely a significant overestimate as most studies directly examining this in a systematic and unbiased fashion find <10%.

**Response: we fully agree with the reviewer’s comment. To emphasis the aspected more we rephrased from “Nevertheless, coinfections are apparently not uncommon which supports considering testing for coendemic pathogens sharing identical tick vectors.” to “Although coinfections do occur, and testing for coendemic pathogens sharing identical tick vectors should be considered, the coinfection rate found in our analysis is very likely an overestimate as most studies directly examining this in a systematic and unbiased fashion find a coinfection rate of less than 10%”.**

Page 49, lines 687-690. Another key consideration that should be addressed is the diagnosis of coinfection in patients for which the vector is not a tick (e.g. B. henselae and O. tsutsugamushi), or for which the vector has little evidence of competence for acquisition or transmission of A. phagocytophilum (e.g. Dermacentor). It would be imprudent to leave readers with the impression that these are biologically plausible events.

**Response: With the already made adjustments to the respective discussion this aspect should now be sufficiently addressed.**

Page 50, lines 695-697. I'm not as certain that the data are entirely discrepant, as the outlier among these publications shows that those diagnosed with and treated appropriately for HGA (in the Belongia paper) has documentable impairments one year after their infection. As far as I know, this is the ONLY paper to address this issue in this way.

**Response: When reviewing the literature, we came to the stated conclusion: “The few studies which investigated the question whether HGA co-infections may be associated with more severe symptoms than HGA mono-infections focus on Lyme co-infection and came to conflicting conclusions [93-95]. It appears that these conflicting results accrue from, or are at least partly attributable to, different case definitions and selection/referral bias [96].” If the expert suggests rewording this section, we would be happy to do so, but in this case we would ask the reviewer for advice on how best to implement this.**

Page 50, lines 698-699. As I noted earlier, the proportion of cases that are consider coinfection globally do not account for this geographic limitation. It might be good to note that because it skews the results; similarly, the occurrence of Powassan coinfection only in the US, skews results (although to a much lower degree). I only note these features for full clarity such that readers understand the nuances.

**Response: We agree and hope that with adding the exemplary clarification regarding the geographic limitation of TBEV to Europe and Asia already made (footer table 7) the vigilant reader will extrapolate to other pathogens with limited geographic range. We do not really see a reasonable way to present this data in more differentiated way without loosing comprehensiveness. In the end this table is supposed to provide an overview on the rough pattern of the coinfections reported in the literature. Teaching the reader the epidemiology of the listed pathogens is beyond the scope of this review.**

Page 50, lines 703-704. I'm not certain why one would accept the implausible notion that this patient was bitten by two different tick species, one of which was not present in that region at that time, and for which there was NO serological reaction in paired sera. PCR tests are good, but not perfect and false positives from contamination do occur, as you appropriately note. The fact that this has not been corroborated better also makes the case for false positive PCR results, and the case for a better classification of diagnostics here.

**Response: We fully agree and consider this issue sufficiently discussed and summarized with “Since no vector is yet known to transmit both pathogens, the probability of a coinfection with both pathogens is overall very low.”**

Page 51, lines 731-732. I absolutely agree, but when both are present, both should be considered to exclude false positive results in one or the other.

**Response: Agree. We added “However, if availability permits, confirmation with both methods should be considered to rule out false positive results by one or the other method.”**

Page 51, line 733. I have long argued against this approach, and agree with a higher cutoff for this single sample method. The argument is that the CDC screens at a dilution of 1:32, and if positive titrates. Provided the concept that a titer could vary by 2 fold lower or 2 fold higher if repeated, indicates that the long held 4-fold rule be applied. Assuming that at some earlier point a case would have no antibodies and this would be reflected by a titer <=32 and thus would require a minimum titer of 128 (four fold higher than 32) to be considered a four-fold change.

**Response:** **We agree.**

Page 52, line 774. Immunodeficiency/suppression. As I suggested earlier, this category should be well defined since the CDC criteria include a range of pre-existing conditions that do not portend the same degree of immunosuppression as seen with organ transplants, immunosuppressive therapy or HIV, to name some. Could this be explained by that? Moreover, there is likely to be a reporting bias when such conditions are noted in truly immunosuppressive populations.

**Response: As addressed above and suggested, we added our applied definition of immunodeficiency/suppression to the footer of table 10: “Definition of immunodeficiency/-suppression: (i) any medical treatment aiming at and leading to impaired immune defence (i.e. corticosteroids above substitution dosage, azathioprine, methotrexate, chemotherapy, biologics a.o.) and (ii) any clinical condition likely to impair immune defence (i.e. hematological malignancies and disorders, asplenia, HIV-infection with a below normal CD4-T-cell count, uncontrolled diabetes mellitus, end-stage kidney or liver disease).” We agree that a reporting bias is very likely existing and, thus added the cautious relativizing note “However, whether this is a true effect or caused by reporting bias remains unclear.”**

Page 53, lines 788-791. Agree - but why is this specific commentary included here? It is a speculation and the data obtained here does nothing to provide support for the speculation.

**Response: Agreed, the commentary was removed.**

Page 53, lines 794-796. Please note that the final case in your list was also given leukoreduced products as well. The point of this is that since the early 2000s, all RBC products have been leukoreduced in the US, and the majority of the transfusion cases happened after that occurred.

**Response: We corrected the table containing this case and added a sentence in the discussion stating that more than half of the reviewed transfusion transmitted cases had received leukoreduced blood products.**

Page 54, lines 802-809. I am intimately familiar with this situation, and must note that SFTS and SFTSV was discovered after the JAMA publication. I was originally told that the original samples used to test in the JAMA cohort were exhausted, but learned years later that some were retained and tested, confirming that the cases were SFTV infection (now Dabie bandavirus). How the original samples tested positive for A. phagocytophilum, I've never learned, and I do not believe that a erratum or retraction of the JAMA paper was ever attempted. It was a disaster.

**Response: Thank you very much for this interesting insight! We found it interesting to collate the information about the events that we found in the literature.**

Page 54, lines 810-814. In general, I agree; however it is well documented that Anaplasma marginale can be easily transmitted via blood contamination and fomites, and given the very high bacterial loads that can occur in HGA, I do not encourage out and out dismissal of this possibility.

**Response: We rephrased this section as follows: “This transmission route has not yet been proven for *A. phagocytophilum*, but is well documented for *A. marginale*. However, given the not insignificant risk of tick exposure among hunters and butchers, transmission through an undetected tick bite in these cases could be an alternative explanation.”**

Page 55, line 844 “asymptomatic”. I think that the use of asymptomatic is not entirely correct here, but perhaps one should consider "subclinical" to reflect that those individuals that develop very mild symptoms and signs are unlikely to seek medical help and would not be otherwise recognized despite mild presentations. See the first case of HGA in Europe for an example (doi: 10.1128/jcm.35.6.1556-1559.)

**Response: Agreed, we adapted as suggested.**

Page 55, line 849. Rashes. I'd recommend to take care with this statement as the range of rash presentations you provide is broad and has no specific relationship with the disease. As I indicated before, some are as a result of concurrent Lyme disease, a distinct rash lesion, and the others are not known, but perhaps related to petechiae with thrombocytopenia or drug-induced rashes. Until a definitive pathophysiologic reason for the rash is proposed, this is like many other symptoms, nonspecific, and infrequent enough to be of little help.

**Response: Agreed, we further relativized by rephrasing “Considering that a rash may be rather rare in HGA, some authors emphasize the importance of considering other or additional tick-borne infections in patients presenting with rash after having sustained a tick-bite.”**

Page 56, lines 871-876. This is a critically important point and especially relevant given the problem illustrated by the cases of nosocomial "HGA" in China that turned out to be SFTV. Of note, several publications regarding Asian (Chinese) HGA have case fatality rates considerably higher than elsewhere, and this should raise a potential red flag, even given what appears to be appropriate diagnostic test results (PCR and seroconversion). I suspect the only certainty can be gained "in future studies" by cultivation of the bacterium in distinct geographic regions from patients with the distinct "paraclinical" presentations.

**Response:** **We fully agree.**

Page 58, lines 903-905. I've rarely observed CNS disease with A phagocytophilum infection, so recommend that these few reports are clinically consistent with those diagnoses, and not simply clinical impressions.

**Response: We fully agree. In the end the issue will remain unresolved: on the one hand very rare manifestations can never be excluded, neither can the possibility of undetected coinfections be excluded… or the reported data were subjective and other authors would have reported them differently…**

Page 58, lines 910-911. I have always had a problem with this publication and was involved with it from the beginning. You will note that there is NO image of the infected HL-60 cells and I was always concerned about PCR contamination. The lack of PCR for B. burgdorferi is typical for testing US CSF, and the lack a serologic response are of no great surprise in the first 14 days is also not usual.

**Response: Thank you also for sharing these thoughts!**

Page 58, lines 912-914. This is a bit speculative, especially given the lack of reporting of clinical bleeding or hemorrhage, except in very severe cases. Coagulation parameters alone do not define coagulopathy, it also requires clinical manifestations.

**Response: A very valid point… We have decided to delete the sentence on coagulopathy without replacement.**

Page 58, line 920. Case fatality is only one measure to consider; HLH and MAS with HGA has a greater severity than in its absence.

**Response: We fully agree with this assessment, but a comparison of severity is unfortunately not supported by the available data in these few cases.**

Page 58, lines 923-925. This is a reasonable point, but misses part of the issue. HLH as defined in the citation includes ALL forms, genetic and acquired (no established genetic predisposition). In general, acquired HLH has a better prognosis than genetic-associated HLH, and thus this comparison is not entirely fair.

**Response: This is true. We now only compared it to the mortality in other infection-associated HLH. We now rephrased from: “Since the overall reported case fatality rate of HLH is 50-80% [146], HGA-associated HLH appears to have a comparatively good outcome when treated with appropriate antibiotics.” to “HLH associated with HGA therefore appears to have a similar mortality compared to infection-associated HLH in general, where case fatality rates of around 20% are reported.”**

Page 60, lines 953-954. SFTSV?

**Response: Yes, we rephrased to clarify.**

Page 60, lines 958-960. SFTSV was first identified in the NEJM paper in 2011. Both of these studies involved patients studied prior to that time. Additional similar publications have not appeared from China. It is appropriate to point this out, but it is also reasonable provide the caveat I note.

**Response: Thanks for pointing this out. We rephrased to clarify.**

Page 60, line 962. Agree - but your suggestion is based on the concept that there was an extensive amount of critical clinical data know about HGA at that time, but here wasn't. In fact, the first several patients identified with HGA (then called the human granulcyctic ehrlichia agent) both had severe disease, including hemorrhage and significant hepatitis among a range other features. I don't think it's appropriate to cast judgement on what happened other than to note that this was the sequence of events and likely misidentified cases of HGA. I'm not certain that the concept of more virulent strains exist there needs any note, because the data to support that, based on human cases is highly questionable and there is NO corollary in the veterinary world for such virulent strains.

**Response: As addressed above we rephrased and we also removed the sentence regarding a putatively more virulent strain.**

Page 61, line 977. In fact, this reference which was the existing guidelines from the IDSA state exactly that.

**Response: We rephrased from “The tendency to opt for 10-14 days is probably due to the fact that experts want possible Lyme co-infection to be covered.” To “ The tendency to opt for 10-14 days is due to the fact that this duration of treatment also covers possible Lyme co-infections.”**

Page 61m lines 996-998. in fact, NO clinical trials have ever been conducted to determine in vivo efficacy for any drug for A. phagocytophilum.

**Response: Thanks for this very valid point! We added “No prospective controlled treatment study has yet been conducted to determine the antimicrobial efficacy in HGA.” as first sentence to the treatment paragraph, respectively.**

Page 63, 3rd Key learning point. The exceedingly rare occurrence of secondary HGA infection despite it being the 2nd most common tick-borne disease in North America says something.

**Response: We are not sure whether we understand the reviewer’s comment correctly as the 3^rd^ key learning point (i.e. “Although usually mild and self-limiting, HGA may cause severe and even life-threatening complications (e.g. acute renal failure, ARDS, multi organ failure a.o.”) is apparently not addressed. What does the reviewer address?**

Page 64, Key Learning points continued, 3rd bullet. It would be worth noting the proportion of patients with severe complications if you're able to provide that. The fact that over 50% required hospitalization is important, but of course has some bias in this sort of study.

**Response:** **We added the rates of the most common severe complications (acute renal failure 9.8%, ARDS 6.3%, multi organ failure 7.5%) to the 3^rd^ bullet point, as suggested.**

Reference 14. you might want to note the specific chapter in the textbook

**Response: Added as suggested.**

S8 Table 25 the endnote references didn’t work here

**Response: This was corrected.**

S6 the values for creatinine should use English words

**Response: Sorry! Corrected.**

S4 publications. The excluded publications lack year designations and appropriate volume and page numbers

**Response: The references list was edited, respectively.**

--------------------

<b>Editorial and Data Presentation Modifications?</b></br><br/>
Use this section for editorial suggestions as well as relatively minor modifications of existing data that would enhance clarity. If the only modifications needed are minor and/or editorial, you may wish to recommend “Minor Revision” or “Accept”.

Reviewer #1: Stated above in the results box.
Reviewer #2: (No Response)

--------------------
 **Summary and General Comments**

Reviewer #1: This manuscript would be a valuable contribution of knowledge if the issues raised are addressed effectively.

Reviewer #2: This comprehensive systematic review and analysis of the literature with regard to clinical aspects of human granulocytic (granulocytotropic) anaplasmosis is an outstanding contribution to the literature that allows an in depth look at and discovery of similarities and differences in how the human disease has been observed, diagnosed, reported and treated on a global scale. The authors have careful devised an appropriate screen to capture as many relevant publications with patient oriented data stratified by a remarkable wide range of features that provide a deep dive into the human disease. The strengths include the use of the systematic review method, as complies with PRISMA guidelines, a comprehensive compilation of an extraordinarily large dataset from which multiple clinical datapoints are extracted and evaluated over multiple regions and over time. The result is a compilation of aspects of the disease that share common features and distinct differences,
providing opportunities to discern knowledge gaps and weaknesses that will require attention as investigations proceed in the future. There are indeed weaknesses, some of which can be readily addressed, others that likely cannot be addressed, and others where the data simply isn’t strong enough to make solid conclusions. This includes the use of all cases rather than those for which diagnostic certainty (grade A-A+) would be most appropriate, and a better consideration of how coinfections were curated given the lack of any diagnostic quality considerations for those infections, and the inclusion of a range of highly implausible coinfection cases. However, the strengths far outweigh the weaknesses in this review, and the work will provide an encyclopedic tabulation of what is know or not known about HGA since its discovery in 1990.

**Response: Finally, we would like to reiterate the point rightly made that we have included all cases in the analyses and have not just concentrated on cases with diagnostic certainty levels A+ and A. We fully agree that focusing on A+ and A cases only would have been the most clean approach. However, already early on we concluded that it will be highly unlikely to distil ultimate evidences from retrospectively analyzing 3rd party data from the sort of literature reviewed (primarily case reports and series; which is considerably different from any meta-analysis of study data). In addition, we wanted to apply the same methodology for HGA and HME (we dearly hope the two reviewers will also provide the review of this second manuscript!) and realized, that if we would only consider A+ and A cases the number of cases would largely be insufficient to provide a good overall picture. However, this is what we primarily aimed for: summarizing the existing data in a more or less concise way and trying to obtain a glimpse of the whole picture, possibly finding some correlations which could then guide future research questions.**

**That our assumption that with a large data set even individual erroneous and questionable data ultimately do not significantly distort the overall picture appears to be correct considering the pretty matching pattern of A+/A cases when compared to the pattern of all cases depicted in Figure 7, 8 and 9.**
